# Machine Learning-Based Self-Induced Scratch Intensity Detection Using Feature Optimization and Multi-Channel Electromyogram Signals for Prevention of Lichenification

**DOI:** 10.3390/bioengineering13070787

**Published:** 2026-07-08

**Authors:** Muhammad Omar Cheema, Alina Akhlaq, Zia Mohy Ud Din, Abdullah Al Aishan, Hedi Ammar Guesmi, Jahan Zeb Gul

**Affiliations:** 1 Department of Biomedical Engineering, Air University, Islamabad 44000, Pakistan; omar.cheema@au.edu.pk (M.O.C.); drzia@au.edu.pk (Z.M.U.D.); 2 Department of Biomedical Engineering, HITEC University, Taxila 47070, Pakistan; alina.akhlaq@hitecuni.edu.pk; 3 Vice Dean for Academic Affairs and Development, King Khalid University, Abha 61421, Saudi Arabia; aalaishan@kku.edu.sa; 4 Department of Electrical Engineering, College of Engineering, Qassim University, Buraydah 52571, Saudi Arabia; 5 Department of Electronic Engineering, Maynooth University, W23 A3HY Maynooth, Ireland; jahanzeb.gul@mu.ie

**Keywords:** chronic pruritus, electromyography, scratching intensity detection, feature extraction, recursive feature elimination, biomedical signal classification

## Abstract

Chronic pruritus in patients with dermatological conditions causes physical discomfort, skin breakdown, sleep disturbance, and overall decline in quality of life. Persistent scratching can lead to a condition known as lichenification, which further deteriorates the skin. Lichenified skin is more susceptible to infections, which further complicates the treatment and prolongs the recovery time. This highlights the importance of accurately detecting and quantifying scratching behavior for effective management and intervention. All the existing technologies to monitor scratching involve the use of external sensor modalities such as an accelerometer and a gyroscope, followed by camera monitoring. However, such scratch detection methods are limited in their reliability in monitoring scratching intensity. This study aims to acquire muscle activity to accurately detect, quantify, and provide feedback on self-induced scratching intensity. Through a meticulous understanding of the synergies of hand muscles, three out of seven forearm muscles were identified that generate consistent and distinctive signals during scratching motions. These signals were acquired, preprocessed, segmented, and analyzed using both time- and frequency-domain features. The extracted 45 features of 3-Channel EMG signals were first optimized using recursive feature elimination and validated by cross-validation accuracies of the recursive feature elimination technique, with the highest optimized accuracy of 0.8628 attained when EMG of a complete combination of muscles is used. This study shows promising results in facilitating enhanced diagnosis and management of scratching intensities. The model may support future wearable devices for generating alerts and providing advanced therapy.

## 1. Introduction

Itch or pruritus may be the result of many different factors, such as dermatological, neurological, and systemic diseases [1,2]. It is among the most irritating symptoms in patients, which appear due to allergic cutaneous illnesses like atopic dermatitis and eczema, infections, and an adverse drug reaction [3]. Itching has a serious negative impact on the quality of life, causing insomnia and even depression [3,4]. Research has shown that chronic pruritus (more than six weeks) has an incidence of approximately 10% of the world population, and 91% of victims of atopic dermatitis have the condition [3,4,5,6]. The Centers for Disease Control and Prevention estimates 1.7 million skin infections are reported every year in the United States [7]. In 2023, recent statistics illustrate that there remains a health concern and that there have been recent spikes to 5.8 million new cases annually [8]. This growth highlights the importance of the need to develop powerful monitoring and prevention systems.

In the clinical picture, itch causes a strong urge to scratch the skin, which can lead to lichenification with thickened and leathery skin [9,10]. This is among the possible complications of uncontrolled and persistent pruritus, which results in excoriation (scratching) and predisposes secondary bacterial infections. When pruritus is managed properly and treated, the risk of complications can be minimized which is instrumental in supporting better outcomes [6]. Traditional measures of pruritus are mainly grounded on clinical outcome assessments and patient-reported outcome assessments [11,12], which are subjective data and give little knowledge on symptom variation beyond clinical conditions. This may result in a misperception of the severity of the disease. Hence, additional indicators and objective measurements are required to assess the efficiency of treatment and underlying factors.

The development of wearable sensor devices has supported objective health measurements both in outpatient and inpatient settings. Human scratching is commonly quantified by wrist-worn actigraphs, including ActiTrac and DigiTrac. In both cases, Ebata et al. and Hon et al. utilized accelerometers that can detect the movement in two and three axial planes, respectively [13,14,15]. But techniques based on accelerometer data find it difficult to accurately detect fine scratching motions constrained to fingers [16]. Alternatively, there are those approaches, where the sound of body-conducted scratching is detected, to document and measure the human scratching behavior, which is then usually followed by video recording [16,17,18,19]. This solution has the limitation of possible distraction by the surrounding sounds and is therefore not practical as a clinical solution, and difficult to automate [18]. A combination of inertial measurement unit and electric potential sensor data was suggested to measure scratching by Jocys et al. [20]. The deficiencies of this approach consist of the fact that several sensors should be attached to several body parts, which would, in practice, not be feasible. Other earlier applications, including Itchtector and Itch Tracker, use smartwatches and their acceleration sensors, demonstrating similar performance to actigraphy when detecting scratching actions [21,22]. But these applications will not differentiate arm and wrist movements.

In recent years, alternative methods to detect scratching with machine learning algorithms have been proposed [18,19,20,21,22,23,24,25]. Digital health technologies allow researchers to collect and analyze measurable data regarding many facets of health, including scratching behavior, which has already demonstrated good results. These technologies have the advantage of objective data capture [26]. As technology improves, there is a growing drive to invent new methods of detecting bio-signals, especially with new commercial wearables. Wearable medical technologies could transform healthcare by continuously monitoring. EMG is another one of the most common biopotentials used with human–machine interfaces, through which muscle contractions can be measured by recording their electrical activity [27]. The movement in the forearm muscles can be monitored using this technique to determine scratching activity. The EMG analysis of the signals will help us to understand more about the muscle activity pattern related to scratching. This could be done by recording surface electromyography using a noninvasive method in order to ensure that the force of the contracting muscles beneath the skin is monitored [28]. A thorough study was conducted to identify the superficial forearm muscles responsible for scratching movements. The study showed that in total, there are seven primary muscles that directly contribute to finger movements of flexion and extension [29]. Out of these seven muscles, three superficial muscles can be selected because of their signal quality and ease of access for sEMG electrode placement.

The BIOPAC MP 36 is a well-known research-grade physiological signal acquisition system and has been extensively applied in biomedical research as it is highly reliable, precise, and applicable to an extensive array of biomedical signals. It is a widely used research-grade system for physiological signal acquisition due to the possibility of recording high-quality, reliable, and reproducible data on a variety of modalities, such as EMG, EOG, ECG, EEG, and EHG [30,31,32,33,34]. Its ability to record both low- and high-frequency biomedical signals makes it essential to ensure that new biomedical devices and machine learning systems are validated. Its multi-channel recording capabilities, coupled with in-built noise reduction and ability to support sophisticated analysis software, make it reliable in characterizing the signals accurately, and provide a baseline upon which to compare wearable and experimental systems of acquisition. The BIOPAC MP 36 is significant because it is considered a reliable reference tool in research, and thus, the acquired signals are of high quality. Artificial intelligence algorithms can be used for the classification of scratch intensity [35]. A comparison between different modalities is presented in Table 1.

Hence, amongst physiological signals, EMG may be a better alternative for the detection of scratching and classification of intensities with the help of machine learning models because it represents the electrical activity of muscles directly responsible for scratching. Machine learning algorithms combined with EMG technology make it more effective to detect and analyze scratching behavior as well as to classify the scratch intensity as low, medium, or high. Irrespective of the advances made in the field of wearable actigraphy, there is still a considerable research gap: the failure to provide the objective definition of benign touch and the different levels of harmful scratching intensity. General limb motion is usually confused with localized scratching by indirect sensors. This is a proof-of-concept sEMG-based scratch intensity discrimination framework combining physiological signal analysis with machine learning classification and feature optimization. This will shift us away from motion-based detection and towards a more clinically relevant tool that will identify which force thresholds are clinically relevant when it comes to causing skin barrier degradation and subsequent lichenification.

In this study, the feasibility of the sEMG signals to detect and classify the intensity level of self-induced scratching is investigated. The proposed framework explores the muscle activation patterns captured from various forearm muscles to see how well they can be used to distinguish between the various levels of scratching. The relationship between the multi-channel EMG signals and the intensity classes are modeled using a machine learning approach, which allows for quantitative assessment of discriminative muscle activity. Furthermore, the contribution to the study of the single muscle and muscle combinations and optimized feature and channel configurations to determine the most informative representations of scratching behavior is investigated. The features are optimized and the classifiers are evaluated to achieve robust and reliable intensity classification performance. The study systematically investigates multi-channel sEMG signals for objective detection of scratching intensity by optimized computational modeling.

## 2. Materials and Methods

### 2.1. Participants

The EMG signals were acquired from 50 right-handed healthy participants. The inclusion criteria required participants with no prior history of neurological, muscular, or dermatological conditions affecting skin sensitivity or muscle activity. This study was conducted in accordance with the Declaration of Helsinki and was also approved by the Ethical Review Board of Air University, Islamabad [36]. Before participating in the experiment, each participant was given a comprehensive verbal explanation of the paradigm and protocols involved, including the associated risks. Subsequently, each participant signed an informed consent before proceeding with the experiment. The targeted age group was based on data from the National Health and Nutrition Examination Survey [2]. According to this, psoriasis and AD are more prevalent in individuals over the age of 20, with no difference in prevalence between men and women. EMG was acquired from both genders. In total, 50 regional subjects, 25 males and 25 females, aged 18–27 years, were involved in the study. All the subjects were students and staff from Air University. Essential physiological parameters and patients’ demographics were recorded before EMG acquisition and are documented in Table 2.

### 2.2. Experimental Paradigm

**Figure 1:**
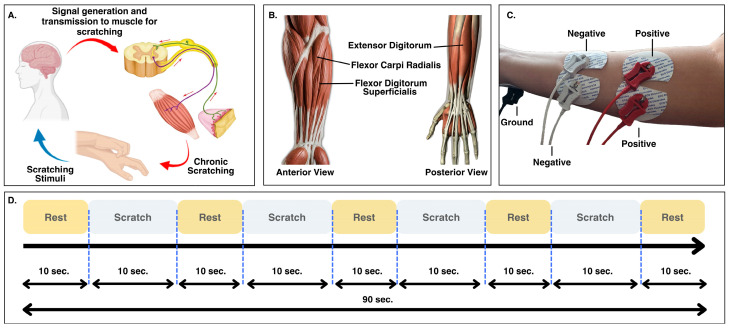
(**A**) EMG generation when scratching is initiated. (**B**) Illustration of the ventral (palmar) and dorsal aspects of the right forearm muscles. (**C**) Placement of recording electrodes to measure the scratching movement in the muscle. Two electrodes were placed longitudinally on each muscle to measure contractions of the extensor digitorum, flexor carpi radialis, and flexor digitorum superficialis. The electrodes with leads in a red color are positive, in a white color are negative, and in black are ground. (**D**) The resting phase shows no muscle activity, minimal muscle involvement is shown in low-intensity scratching, moderate muscle activity is seen in medium-intensity scratching, and high-intensity scratching is indicated by a strong amplitude of the EMG signal. Varied EMG amplitudes are visible in dummy or non-scratching movements.

Preparatory measures were carried out before data acquisition for every participant. The subjects were asked to remove any jewelry or wristwatches they were wearing to acquire noise-free EMG. Before electrode placement, the skin was thoroughly cleaned using 70% isopropyl alcohol swabs to ensure optimal signal quality. The mechanism of EMG signal generation is shown in Figure 1A. For each participant, the scratching activity of varying intensities was performed for five repetitions per intensity, with each intensity lasting 10 s, followed by 10 s of rest, as shown in Figure 1C. Verbal instructions were provided to prompt the initiation and cessation of the activity. To minimize signal interference, participants were instructed to maintain relaxed hand muscles throughout the recording session, as EMG signals may fluctuate with changes in hand orientation. Each participant was comfortably seated on a chair with a straight back and relaxed shoulders and arms. Prompts were provided to guide participants in increasing the scratching intensity using PsychoPy 2023.2.3 software (Open Science Tools Ltd., Nottingham, UK). PsychoPy 2023.2.3 (Open Science Tools Ltd., Nottingham, UK) is an open-source cross-platform software package for experimental psychology, neuropsychology, and neuroscience for creating accurate studies for research. The software provides a dual interface; researchers can carry out experiments visually on a graphical Builder or create custom programs using the Coder interface. This particular release is focused on the extremely precise presentation of auditory and visual stimuli, with millisecond accuracy when collecting data locally in the lab. This helped the subject perform the activity according to the prompts displayed on the screen for a designated period.

**Figure 2:**
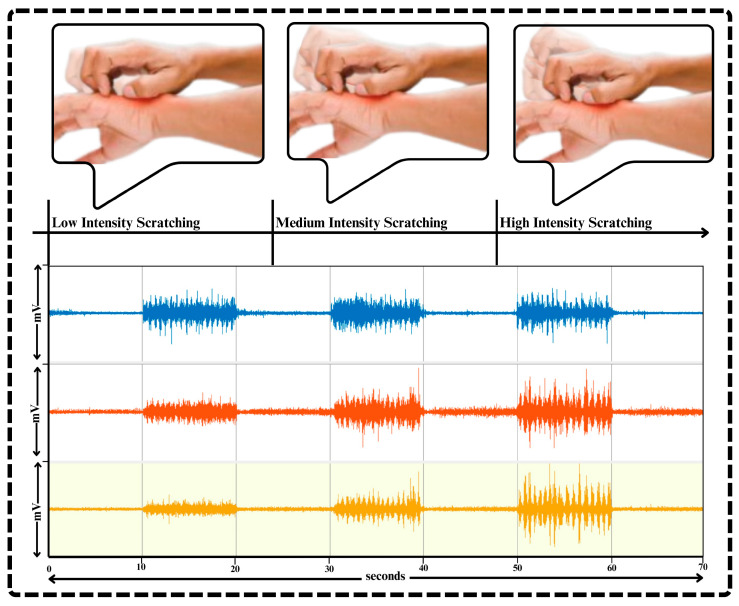
Paradigm for EMG signal acquisition: The paradigm includes five phases, i.e., rest, dummy scratching, low intensity scratching, medium-intensity scratching, and high-intensity scratching.

Data acquisition involved the recording of four distinct scratching motions: dummy, low intensity, medium intensity, and high intensity, alongside the fifth rest class as illustrated in Figure 2. The dummy scratching involved mimicking the action of scratching in the air. This is important for differentiating real and fake scratching in daily life, especially while the subject is sleeping. Hence, there are 5 different segments in the designed paradigm named rest, dummy scratching, low-intensity scratching, medium-intensity scratching, and high-intensity scratching. Using the PsychoPy 2023.2.3 software (Open Science Tools Ltd., Nottingham, UK), during the low-intensity scratching, the subject was guided to scratch lightly, during the medium-intensity scratching, the subject was guided to scratch with moderate intensity, and finally, during high-intensity scratching, the subject was guided to scratch with high intensity. During all segments, the subject used the dominant right hand. This paradigm, with respect to time in seconds, was standardized for all 50 subjects, which means that the subject has to perform the required task within the assigned time.

To ensure robust data for machine learning-based classification, five trials were performed for each movement for a duration of 90 s. After each trial, the participants were given a rest interval to minimize muscle fatigue. By adhering to this data collection protocol, a comprehensive dataset was compiled, representing the muscle activation patterns.

### 2.3. Experimental Setup

The key muscles chosen were the extensor digitorum, flexor carpi radialis, and flexor digitorum superficialis muscles, as shown in Figure 1B. The electrode placement was made such that firstly the muscles are identified for each subject and the Ag/AgCl part of the electrode is exactly placed on the designated part of that muscle. For negative (white lead), it is placed on the belly of the muscle. For positive (red lead), it is placed on the insertion of the muscle. For ground (black lead), it is placed on the bony structure where there is no motion to avoid the motion artifact in the EMG signals. Hence, crosstalk amongst the muscles can be minimized effectively.

The BIOPAC MP 36 data acquisition system was used to acquire the desired EMG signals by utilizing its 3 channels. BIOPAC Systems, Inc. (Goleta, CA, USA) is recognized for its advanced capabilities in real-time biological signal measurement and recording from the human body [37]. The acquired data were subsequently recorded by employing the AcqKnowledge software (BIOPAC Student Lab 4.1.1). The EMG signal was recorded at a sampling rate of 2 kHz (2000 samples per second) and then subjected to a filtering process. Initially, a 50 Hz notch filter was applied to eliminate power-line interference, followed by a band-pass Butterworth with lower and upper cut-off frequencies of 30 and 1000 Hz, respectively, to refine signal quality. The filters are predefined in BIOPAC Student Lab software with a quality factor of 1. The amplitude of the sEMG signal is then recorded to analyze the intensity of the muscle activity engaged in a particular movement.

### 2.4. Feature Extraction

**Figure 3:**
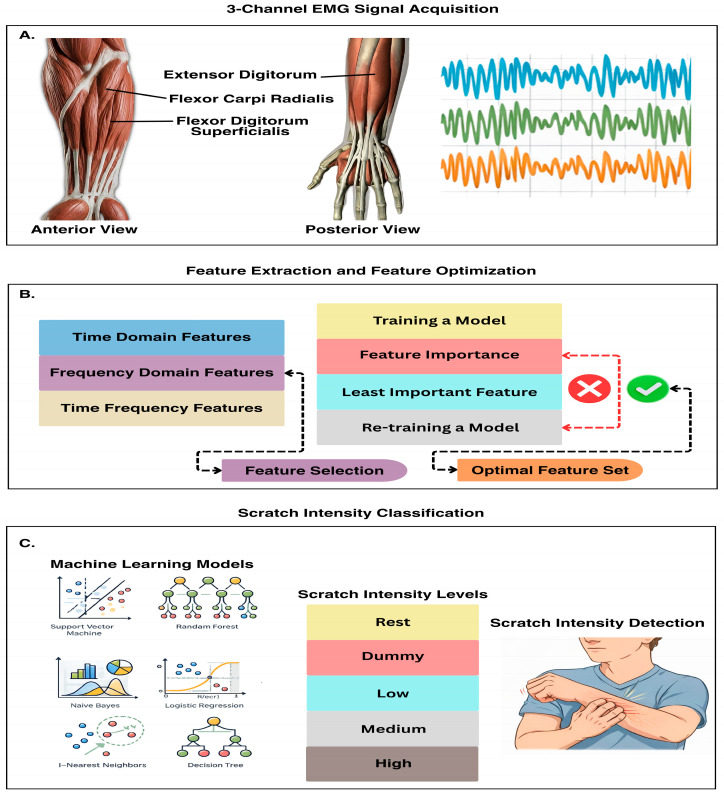
Overview of the proposed framework for scratch intensity detection using multi-channel EMG signals. (**A**) Placement of electrodes and acquisition of three-channel EMG signals from forearm muscles, including extensor digitorum, flexor carpi radialis, and flexor digitorum superficialis, along with representative signal waveforms. (**B**) Feature extraction (time-domain, frequency-domain, time–frequency, and nonlinear features) followed by recursive feature elimination (RFE), where features are iteratively ranked using model training and feature importance, least important features are removed, and the model is retrained to obtain an optimal feature subset. (**C**) Classification of scratch intensity using multiple machine learning models, categorizing activity into rest, dummy, and low-, medium-, and high-intensity levels for effective scratch behavior detection and prevention of lichenification.

Raw EMG contains various noises and artifacts that cause decreased efficiency for classification. To enhance the classifier’s performance, various EMG features were extracted to improve accuracy and effectiveness. This is achieved by transforming the input signal into a set of representative signal features, which enables the extraction of relevant information by filtering out unwanted components and interferences. The EMG feature sets are categorized into three main groups: time domain, frequency domain, and time–frequency or time–scale representation [38]. Despite the availability of multiple feature domains, time-domain features are predominantly favored due to their superior classification performance and their lower computational cost compared to alternative domains. They offer an efficient approach to feature extraction, as they do not require additional transformations and are computed directly from raw EMG time series data [39]. In total, 10 time domain features were extracted, also targeting the Hudgins feature set [40]. The 3-channel EMG from extensor digitorum, flexor digitorum superficialis, and flexor carpi radialis muscles were used for processing and feature extraction. The features were extracted after EMG rectification and enveloping, as shown in Figure 3.

A total of 15 features were extracted from each channel of EMG, including both the time- and frequency-domain features. Hence, the total features for all 3 channels of EMG were 45. The features include mean, median, standard deviation, variance, covariance, kurtosis, skewness, root mean square, square integral, average energy, temporal moment, Willison amplitude, zero crossing, mean frequency, and median frequency. Detailed mathematical description of these features is mentioned in the Supplementary Materials.

### 2.5. Feature Selection, Optimization, and Machine Learning Classification

The proposed methodology, as shown in Figure 3, also includes the optimization of features using recursive feature elimination. It is a wrapper-type feature selection method. RFE operates by trying to find a subset of features beginning with all features in the training set and then trying to remove features until the required number is achieved. It does this by training the given machine learning algorithm, which is in the middle of the model, ranking features by importance, rejecting the least important features, and retraining the model. This step is continued until only a desired number of features is left. The primary logic is that the most relevant features will produce the largest effects on the target variable and will therefore be more helpful in predicting the target. In each iteration, it uses a model, typically a linear regression or support vector machine, to estimate the relative importance of each feature, and the features with the least importance are dropped.

Once the extracted features are optimized, they are used to train the machine learning classifiers for 5 different classes, i.e., rest, dummy scratch, low-intensity scratch, medium-intensity scratch, and high-intensity scratch. The performance metrics are evaluated, including the test accuracy, precision, recall, F1 score, and 5-fold cross-validation accuracy. This process took a step-wise computational path in order to test and measure feature selection and classification of multi-channel EMG signal data. The dataset, in Excel format, was preprocessed first to locate the label and file name columns and deduce channel-based feature groups. To select features, a grid-search-based recursive feature elimination, combining with a Random Forest base estimator, was applied to all potential sizes of a feature subset, performing a stratified five-fold cross-validation. Random Forest has been used as the base estimator with recursive feature elimination (RFE) since it is suitable when working with data that is of high dimensionality and non-homogeneous, as in the case of multi-channel EMG signals in biomedical applications. Random Forest, being an ensemble learning algorithm that relies on bagging and decision trees, is useful at modeling complicated nonlinear interactions between features and is also resistant to noise, variability, and overfitting, all of which are prevalent in EMG recordings. It provides an embedded feature ranking mechanism, which is based upon measures like Gini impurity reduction or permutation importance, and offers a statistically sound basis to iterative feature dropping in the RFE framework. In addition, Random Forest does not assume independent features and or a specific data distribution, and it is resistant to multicollinearity and scaling variance across time-domain, frequency-domain, and statistical EMG descriptors. This combination is what guarantees that the feature subsets that are chosen are discriminative and have a higher reliability in future multi-classification of scratch intensities. The best subset of features in each channel arrangement (single, combined, and all channels) was selected according to the maximum cross-validation accuracy, and the chosen features were used in further modeling.

The configuration for recursive feature elimination is represented in Table 3. Through the algorithm, a fixed random seed of 42 was set to ensure reproducibility. The extraction of features provided 45 features of three EMG channels including the time-domain and frequency-domain descriptors. Recursive feature elimination (RFE) was conducted based on a Random Forest classifier of 200 estimators and class balancing and was applied to 5-fold stratified cross-validation across all possible sizes of feature subsets. Multiplex machine learning models were utilized with default hyperparameters and their performance was measured both with stratified train–test splitting (80/20) and cross-validation. Oversampling, undersampling, SMOTE, ADASYN and SMOTE-ENN were used to deal with class imbalance. Standard scaling was used to normalize all features before the training of the models.

The classification phase used a wide variety of machine learning models, such as k-Nearest Neighbors, Linear Discriminant Analysis, Naive Bayes, Decision Tree, Random Forest, Support Vector Machine, Gradient Boosting, Multi-layer Perceptrons, AdaBoost, Extra Trees, XGBoost, LightGBM, and CatBoost. In order to deal with possible class imbalance, various resampling methods were incorporated into the pipeline, such as random oversampling, random undersampling, SMOTE, ADASYN, and SMOTE-ENN, in addition to the original unbalanced data. All classifiers were trained and tested under these balancing plans on stratified five-fold cross-validation on training data, then independently tested on held-out test data. Accuracy, precision, recall, and F1-score were used as measures of performance, both as weighted averages and by class. The measure of overfitting was the difference between training and test accuracy. Classifier and balancing method comparisons were visualized using cross-validation curves, confusion matrices, and radar plots. Evidence-based interpretation of all intermediate and final results, such as selected features, classification metrics, and accuracy-overfitting summaries, was exported into formatted Excel files and graphical plots. This methodology allowed a strict comparison of feature selection strategies, balancing techniques, and classifiers to optimize the predictive performance in the provided dataset.

## 3. Results

**Figure 4:**
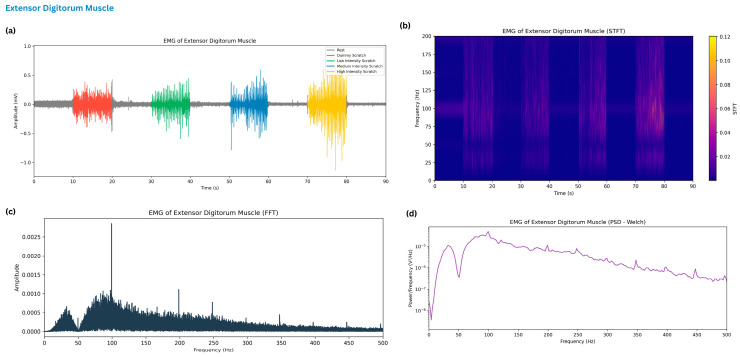
(**a**) EMG acquired from extensor digitorum muscle, (**b**) Short-Time Fourier Transform, (**c**) Fourier Transform, and (**d**) power density spectrum of the acquired 3-channel EMG signals according to the paradigm, including rest, dummy scratch, low intensity scratch, medium-intensity scratch, and high-intensity scratch.

**Figure 5:**
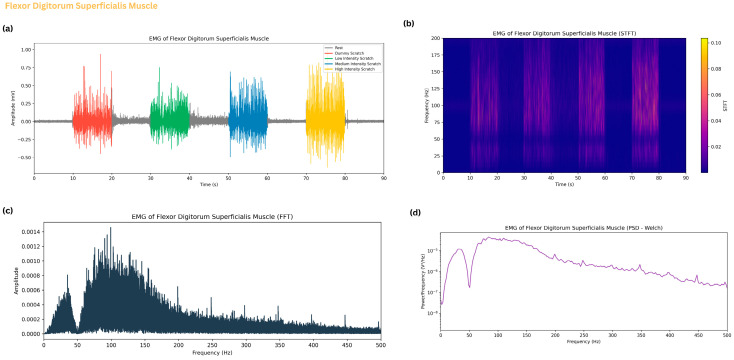
(**a**) EMG acquired from flexor digitorum superficialis muscle, (**b**) Short-Time Fourier Transform, (**c**) Fourier Transform, and (**d**) power density spectrum of the acquired 3-channel EMG signals according to the paradigm, including rest, dummy scratch, low intensity scratch, medium-intensity scratch, and high-intensity scratch.

**Figure 6:**
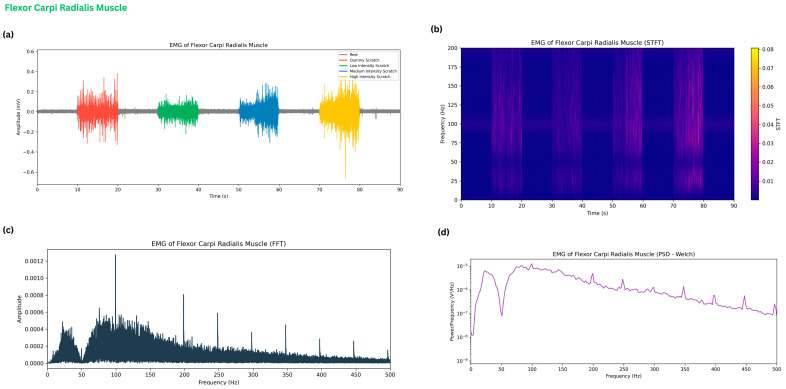
(**a**) EMG acquired from flexor carpi radialis muscle, (**b**) Short-Time Fourier Transform, (**c**) Fourier Transform, and (**d**) power density spectrum of the acquired 3-channel EMG signals according to the paradigm, including rest, dummy scratch, low intensity scratch, medium-intensity scratch, and high-intensity scratch.

Figure 4, Figure 5 and Figure 6 show the multi-channel electromyography pattern of the muscles extensor digitorum, flexor digitorum superficialis, and flexor carpi radialis during a controlled experimental paradigm consisting of five distinct conditions: rest, dummy scratch, low-intensity scratch, medium-intensity scratch, and high-intensity scratch. The plots in the first row are time-domain EMG signals (a), the amplitude change in which distinctly indicates the intensity of various tasks; the more intense the task, the more intense the level of contraction and, accordingly, the more intense the EMG activity. The second row shows time–frequency analysis by the Short-Time Fourier Transform (b), which shows the nonstationary spectral variation in muscle activation. Distinct bursts of activity can be observed during the execution of tasks, especially when the intensity of the task is more demanding, with more robust motor unit recruitment. The frequency-domain representation, as represented by the Fast Fourier Transform (c), is presented in the third row and indicates the prevalent frequency content of muscle activity under the conditions. The fourth row shows the power spectral density as estimated by the Welch method (d), which provides a strong measure of the signal power distribution with respect to frequency bands. Taken together, these analyses offer a global description of EMG activity of the three muscles, both in terms of the temporal dynamics of the activity, spectral composition, and power distribution.

Table 4 represent the impact of recursive feature elimination (RFE) on cross-validation accuracy when used on the original dataset with no augmentation, i.e., without using any conventional dataset balancing technique like random oversampling, random undersampling, synthetic minority oversampling technique (SMOTE), adaptive synthetic sampling (ADASYN), and synthetic minority oversampling edited nearest neighbor technique (SMOTE ENN). Classification accuracy increased with the number of features per muscle when the muscles were looked at separately, but then the performance leveled off. On the extensor digitorum muscle (CH1), twelve out of fifteen features were identified to be ideal, resulting in a cross-validation rate of 0.7429. In the case of the flexor digitorum superficialis muscle (CH2), all fifteen features were selected, resulting in a maximum accuracy of 0.74. The flexor carpi radialis muscle (CH3) only needed ten features to achieve a maximum accuracy of 0.76. When the features of two muscles were optimized and combined, there was a noticeable improvement. The combination of extensor digitorum and flexor digitorum superficialis muscles (CH1 + CH2) accuracy was 0.8057 with twenty-nine features, whereas extensor digitorum and flexor carpi radialis (CH1 + CH3) accuracy was 0.8171 with twenty-seven features. The best accuracy between the two-muscle pairs was achieved in flexor digitorum superficialis and flexor carpi radialis (CH2 + CH3), where the accuracy was 0.8229 and twenty-six features were selected. Whereas, when all the three muscles were combined for machine learning classification, it resulted in the maximum cross-validation accuracy of 0.8629 being obtained with forty-four features chosen among forty-five. The recursive feature elimination (RFE) process only kept 44 out of 45 features in the final multi-channel model.

**Figure 7:**
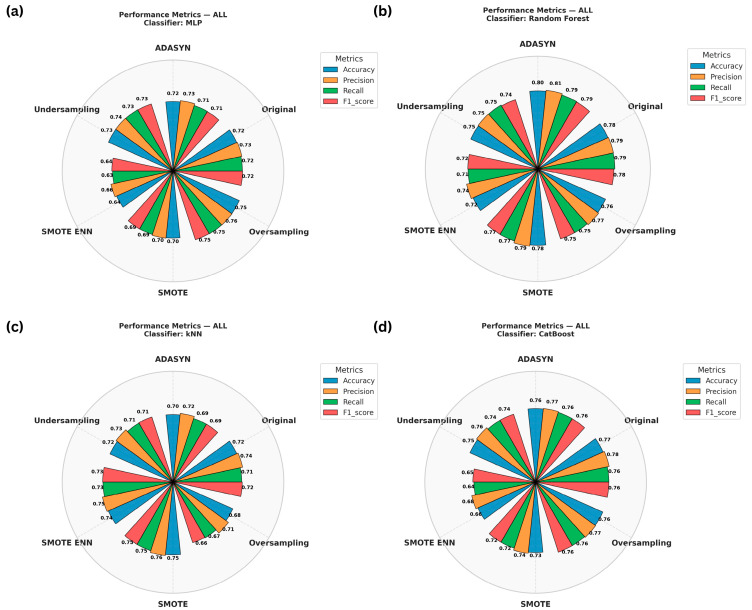
Radial histogram plots (**a**–**d**) representing performance metrics, i.e., accuracy, precision, recall, and F-1 score for different machine learning classifiers, including features extracted from EMG of all three muscles, while different data balancing techniques are used.

The results from Figure 7a–d show accuracy, precision, recall, and F1 score when different sampling techniques were used and all three muscles were taken. The other results when individual and muscle pairs were used, are provided in the Supplementary Materials.

**Figure 8:**
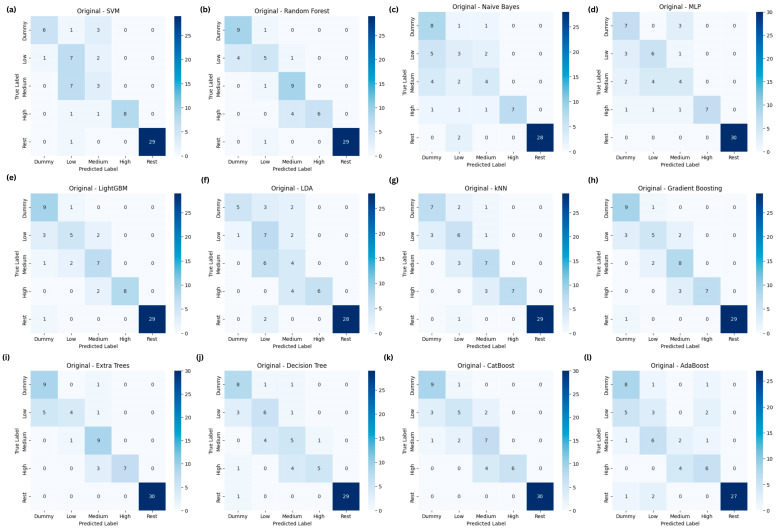
Confusion matrices (**a**–**l**) representing true and predicted labels when the original dataset is used without any balancing technique.

Figure 8a–l represent twelve confusion matrices, each corresponding to a different machine learning classification model applied to the original dataset without any balancing technique. The confusion matrices show how well each algorithm predicts categorical labels (dummy, low, medium, high, rest) compared to their true values.

Table 5 shows that ensembles based on trees (Random Forest, Extra Trees, Gradient Boosting, LightGBM, CatBoost) are always balanced in terms of performance across classes. As an example, the maximum F1-scores of dummy (0.82), medium (0.72), and perfect rest were created by Extra Trees. The performance of kNN and MLP also remain consistent, especially on rest and high classes. Linear models (LDA, SVM, Naive Bayes) do reasonably well, separating well on rest and high.

## 4. Discussion

This study shows that the use of surface electromyography (sEMG) is a promising alternative to traditional methods like accelerometers, gyroscopes, acoustic sensors and video monitoring for the detection and quantification of scratching behavior. The proposed framework was able to directly capture neuromuscular activity which occurred during scratching by recording the electrical activity of the extensor digitorum, flexor carpi radialis and flexor digitorum superficialis muscles. The results demonstrated that each muscle channel gave discriminative information, but multiple muscle channels gave a more comprehensive and reliable feature space for the classification of different kinds of scratches under different intensities. This demonstrates the need for synergistic muscle response for distinguishing scratching.

The optimized features were both time- and frequency-domain features, such as variance, root mean square, Willison amplitude, mean frequency and median frequency. Consistent with this, both the amplitude and frequency modulations are retained, suggesting that the scratching behavior has temporal and spectral variations rather than one type of variation pertained. The recursive feature elimination boosted the classification efficiency by eliminating noise and retaining the most informative features. Moreover, the application of ensemble learning techniques through a stratified 5-fold cross-validation reduced overfitting and achieved good performance for each of the scratching classes, showing that features extracted were representative of meaningful neuromuscular patterns and not only specific to the dataset.

The results align with the findings from the literature, which have shown that indirect sensing modalities are not good for detecting fine motor activity. While acoustic and video-based systems are sensitive to noise levels and privacy concerns and can be limited by their use in long term monitoring, accelerometer and gyroscope-based systems are prone to confusion from subtle finger movements and general movement of the limbs. Compared with this, the proposed EMG-based approach is directly capturing the muscle activation patterns related to scratching, resulting in increased specificity and sensitivity. Another advantage of the proposed framework is the capability to discriminate between low-, medium- and high-intensity scratch detection, which further highlights the clinical relevance of the proposed approach beyond the binary detection of a scratch.

Multiple muscle channels were combined for classification, and improvement in classification performance was observed, indicating that different muscles of the forelimb provide complementary information in the scratching activity. Moderate classification rate was obtained by each muscle, but the multi-channel configuration yielded improved cross-validation performance for all trials suggesting that synergistic activation of muscle groups may more accurately reflect the intensity of the scratching behavior. In addition, a high percentage of the extracted features was kept in the recursive feature elimination process, indicating that both temporal and spectral EMG descriptors provided valuable information for classification. The slight difference in cross-validation and test on independent data also suggests better performance by the model. Furthermore, the comparison of the balancing techniques showed that most classifiers achieved a similar accuracy even with simple balancing procedures, indicating the robustness of the extracted multi-channel EMG feature space for scratch intensity detection.

One other aspect of this study that has been important is the use of a relatively simple acquisition system consisting of three channels as opposed to earlier multimodal acquisition systems that had several sensors throughout the body. This simplified hardware configuration helps to make future wearable applications more feasible. Further, the machine learning pipeline allowed objective tuning of feature importance and tuning of classification boundaries without suffering a loss of performance, handling all the features without sacrificing compute efficiency. The results suggest that the proposed framework could be applicable to portable and real-time monitoring systems, aimed at managing chronic pruritus.

There are several limitations that should be recognized, however, even with the positive outcomes. This work is proof of concept with healthy subjects in a controlled laboratory setting with self-induced scratching paradigms. The conditions allowed the collection of high-quality EMG signals but may not be appropriate for the natural scratching behavior that may occur in natural environments. Further, only healthy young adults were included in the study, and the results need to be validated in patients with chronic pruritus or dermatological diseases. The period of recording was also brief, and the continuous recording of long-term monitoring, especially during sleep, could yield further clinically relevant information. Furthermore, the BIOPAC MP36 system proved effective for the acquisition of signals, but further research is needed to find miniature, low-power, and wireless wearable systems to use in the real world every day.

The clinical validation in dermatological patients, the monitoring in a naturalistic follow-up and the integration of wearable hardware should be investigated in future studies. Machine learning and deep learning methods could be further enhanced to boost classification accuracy and personalization. Furthermore, when used together with other physiological signals like EDA or HRV, EMG can improve the comprehension of the itch–scratch cycle. Haptic or electrical stimulation may also be integrated with therapeutic feedback to help decrease scratching behavior and prevent the skin damage that occurs from chronic pruritus and lichenification.

The objective scratch intensity detection and monitoring by direct neuromuscular measurement is shown to be a promising approach. The proposed framework is more specific and clinically relevant than the indirect motion-based methods, as it does not rely on motion-based sensing. This method could be used for better treatment of chronic itch and prevention of complications like lichenification with further clinical use and wearable application.

## 5. Conclusions

Existing scratch detection devices are not sufficiently capable of detecting complex hand movement and the intensity level associated with itching and scratching. An EMG signal contains important, useful information regarding the neuromuscular system that can be studied by analyzing these signals. This approach reduces the reliance on movement-based features, which makes it more effective in distinguishing the movement and the different scratch intensities. It also minimizes errors by eliminating confounding factors like external body movements. In the future, clinical trials can be conducted for validation on subjects with scratching issues, and a therapeutic system can be integrated for prevention as well as treatment of lichenification.

## Figures and Tables

**Table 1:** Comparison between different modalities to detect scratching.

Feature	EMG (Surface EMG)	Accelerometer (Actigraphy)	Acoustic (Sound)	Video/Camera
Data Source	Electrical activity of muscles (e.g., forearm).	Physical movement/acceleration of the limb.	Body-conducted sounds of scratching.	Visual recording of patient behavior.
Accuracy	High; detects fine motor activity and muscle synergy.	Moderate; struggles with fine finger movements.	Variable; can capture specific friction sounds.	High; but requires manual review.
Intensity Tracking	Able to classify instructed low, medium, and high force.	Poor; often confuses general limb motion with scratching.	Limited data on force/pressure.	Subjective visual assessment of vigor.
Environmental Interference	Low; monitors signals beneath the skin.	High; movement from walking or arm waving creates noise.	High; easily distracted by surrounding ambient noise.	Lighting and occlusion can block view.
Feasibility	High, non-invasive and requires a wearable device.	High; usually integrated into a wearable device.	Low; requires sensors positioned to record body sound.	Low; privacy concerns and stationary setup.
Clinical Utility	May help estimate scratching intensity	Useful for general activity but lacks specificity for itch.	Difficult to automate and scale.	Mostly used to validate other sensors.

**Table 2:** Summary of participant demographics, physiological parameters, and database composition.

Parameter	Description
Age Group	22 ± 2.5
No. of Participants	50
Oxygen Saturation	>94%
BMI	20–25
Dominant Hand	Right-handed
No. of Channels	03
No. of Repetitions	05
Scratching Intensities	Low, medium, high
Sampling Rate (Hz)	2000

**Table 3:** Parameters in recursive feature elimination method.

Parameter	Value
Estimator	RandomForestClassifier
n_estimators	200
class_weight	Balanced
Feature Selection	Exhaustive (1 to N)
Selection Criterion	Highest CV accuracy
Tie-break Rule	Smallest number of features

**Table 4:** Channel-wise feature optimization using the recursive feature elimination method.

Muscle Name (s)	Channel (s)	No. of Total Features	No. of Features Selected	RFE Best CV Accuracy	Selected Features
Extensor Digitorum Muscle	CH1	15	12	0.742857	StdDev, Variance, Covariance, Kurtosis, Skewness RMS, Square Integral, Average Energy Temporal Moment, Willison Amplitude, Zero Crossing Mean Frequency
Flexor Digitorum Superficialis Muscle	CH2	15	15	0.74	Mean, Median, StdDev, Variance, Covariance Kurtosis, Skewness, RMS, Square Integral, Average Energy Temporal Moment, Willison Amplitude, Zero Crossing Mean Frequency, Median Frequency
Flexor Carpi Radialis Muscle	CH3	15	10	0.76	Median, StdDev, Variance, Covariance, Kurtosis, Skewness RMS, Square Integral, Average Energy Mean Frequency
Extensor Digitorum Muscle + Flexor Digitorum Superficialis Muscle	CH1 + CH2	30	29	0.805714	CH1: Mean, Median, StdDev, Variance, Covariance, Kurtosis, Skewness, RMS, Square Integral, Average Energy, Temporal Moment, Willison Amplitude, Zero Crossing, Mean Frequency, Median Frequency CH2: Mean, Median, StdDev, Variance, Covariance, Kurtosis, Skewness, RMS, Square Integral, Average Energy, Temporal Moment, Willison Amplitude, Zero Crossing, Mean Frequency
Extensor Digitorum Muscle + Flexor Carpi Radialis Muscle	CH1 + CH3	30	27	0.817143	CH1: Median, StdDev, Variance, Covariance, Kurtosis, Skewness, RMS, Square Integral, Average Energy, Temporal Moment, Willison Amplitude, Zero Crossing, Mean Frequency CH3: Mean, Median, StdDev, Variance, Covariance, Kurtosis, Skewness, RMS, Square Integral, Average Energy, Temporal Moment, Willison Amplitude, Zero Crossing, Mean Frequency
Flexor Digitorum Superficialis Muscle + Flexor Carpi Radialis Muscle	CH2 + CH3	30	26	0.822857	CH2: Mean, StdDev, Variance, Covariance, Kurtosis, Skewness, RMS, Square Integral, Average Energy, Temporal Moment, Willison Amplitude, Mean Frequency CH3: Mean, Median, StdDev, Variance, Covariance, Kurtosis, Skewness, RMS, Square Integral, Average Energy, Willison Amplitude, Zero Crossing, Mean Frequency, Median Frequency
Extensor Digitorum Muscle + Flexor Digitorum Superficialis Muscle + Flexor Carpi Radialis Muscle	ALL	45	44	0.862857	CH1: Mean, Median, StdDev, Variance, Covariance, Kurtosis, Skewness, RMS, Square Integral, Average Energy, Temporal Moment, Willison Amplitude, Zero Crossing, Mean Frequency, Median Frequency CH2: Mean, Median, StdDev, Variance, Covariance, Kurtosis, Skewness, RMS, Square Integral, Average Energy, Temporal Moment, Willison Amplitude, Zero Crossing, Mean Frequency CH3: Mean, Median, StdDev, Variance, Covariance, Kurtosis, Skewness, RMS, Square Integral, Average Energy, Temporal Moment, Willison Amplitude, Zero Crossing, Mean Frequency, Median Frequency

**Table 5:** Performance metrics including precision, recall, and F-1 Score for individual classes when the original dataset is used without any balancing technique.

Classifier	Class	Precision	Recall	F-1 Score
k-Nearest Neighbors	Dummy	0.7	0.7	0.7
	Low	0.5	0.6	0.545455
	Medium	0.583333	0.7	0.636364
	High	1	0.7	0.823529
	Rest	1	0.966667	0.983051
Linear Discriminant Analysis	Dummy	0.833333	0.5	0.625
	Low	0.388889	0.7	0.5
	Medium	0.333333	0.4	0.363636
	High	1	0.6	0.75
	Rest	1	0.933333	0.965517
Naïve Bayes	Dummy	0.444444	0.8	0.571429
	Low	0.333333	0.3	0.315789
	Medium	0.5	0.4	0.444444
	High	1	0.7	0.823529
	Rest	1	0.933333	0.965517
Decision Tree	Dummy	0.666667	0.8	0.727273
	Low	0.461538	0.6	0.521739
	Medium	0.5	0.6	0.545455
	High	1	0.5	0.666667
	Rest	1	0.933333	0.965517
Random Forest	Dummy	0.666667	0.8	0.727273
	Low	0.571429	0.4	0.470588
	Medium	0.642857	0.9	0.75
	High	0.875	0.7	0.777778
	Rest	1	0.966667	0.983051
Support Vector Machine	Dummy	0.857143	0.6	0.705882
	Low	0.411765	0.7	0.518519
	Medium	0.333333	0.3	0.315789
	High	1	0.8	0.888889
	Rest	1	0.966667	0.983051
Gradient Boosting	Dummy	0.692308	0.9	0.782609
	Low	0.625	0.5	0.555556
	Medium	0.615385	0.8	0.695652
	High	1	0.7	0.823529
	Rest	1	0.966667	0.983051
Multilayer Perceptron	Dummy	0.636364	0.7	0.666667
	Low	0.461538	0.6	0.521739
	Medium	0.555556	0.5	0.526316
	High	1	0.7	0.823529
	Rest	1	1	1
AdaBoost	Dummy	0.533333	0.8	0.64
	Low	0.25	0.3	0.272727
	Medium	0.333333	0.2	0.25
	High	0.6	0.6	0.6
	Rest	1	0.9	0.947368
Extra Trees	Dummy	0.75	0.9	0.818182
	Low	0.8	0.4	0.533333
	Medium	0.6	0.9	0.72
	High	0.875	0.7	0.777778
	Rest	1	1	1
LightGBM	Dummy	0.642857	0.9	0.75
	Low	0.625	0.5	0.555556
	Medium	0.636364	0.7	0.666667
	High	1	0.8	0.888889
	Rest	1	0.966667	0.983051
CatBoost	Dummy	0.692308	0.9	0.782609
	Low	0.625	0.5	0.555556
	Medium	0.538462	0.7	0.608696
	High	1	0.6	0.75
	Dummy	0.692308	0.9	0.782609

## Data Availability

The data supporting the findings of this study are not publicly available due to patient privacy and institutional restrictions. However, anonymized data and analysis scripts can be obtained from the corresponding author upon reasonable request.

## Supplementary Materials

The following supporting information can be downloaded at: https://www.mdpi.com/article/10.3390/bioengineering13070787/s1, Figures: Results obtained when individual and pairs of muscles were taken; File: Details of Features Extraction. The supplementary figures and file are provided for the results section and materials and methods section.
