# Supplementary material for: Machine Learning-Based Self-Induced Scratch Intensity Detection Using Feature Optimization and Multi-Channel Electromyogram Signals for Prevention of Lichenification
Source: Bioengineering (Basel). 2026 Jul 8;13(7):787. doi: 10.3390/bioengineering13070787 (PMC13405920; doi:10.3390/bioengineering13070787)
Supplement: Supplementary file 1 [file bioengineering-13-00787-s001.zip › Features Formulas/Feature Formulas Supplementary Material.pdf]

Feature Extraction

The features are described as,

Mean: It is the average amplitude of the EMG signal that shows the central tendency of muscle activity in the time domain. It also reflects the baseline level of muscle contraction. It can be represented by an equation as

$$\mu = \frac{1}{N}\sum x[n] \qquad \text{(S1)}$$

Where μ is the mean value of the EMG signal, N is the total number of samples, and $x[n]$ is the amplitude of the EMG signal at the name n.

Median: It is the middle value of the EMG signal distribution. It is helpful in EMG analysis where sporadic spikes may appear.

$$Median(x) = middle\ value\ of\ sorted\ \ x[n] \qquad \text{(S2)}$$

Where $Median$ is the middle value of the ordered EMG amplitudes and $x[n]$ is the amplitude of the EMG signal at sample n.

Standard Deviation: It is the variability of the EMG signal around the mean value. A high value of standard deviation indicates larger fluctuations in muscle activity. It can be represented by an equation as

$$\sigma = \sqrt{\left(\frac{1}{\mathrm{N}}\right)\sum \left(x[n] - \mu\right)^2} \qquad \text{(S3)}$$

Where σ is the standard deviation of the EMG signal, μ is the mean value of the signal, $x[n]$ is the amplitude of the EMG signal, and N is the number of samples.

Variance: It is the squared value of the deviation of the EMG signal from its mean value. It represents the muscle activation intensity.

$$\sigma^2 = \left(\frac{1}{\mathrm{N}}\right)\sum \left(x[n] - \mu\right)^2 \qquad \text{(S4)}$$

Where σ^2 is the variance of the EMG signal, μ is the mean value of the signal, $x[n]$ is the amplitude of the EMG signal, and N is the number of samples.

Covariance: It represents the similarity or dissimilarity of variance between two EMG signals. It is helpful in the quantification of muscle coordination.

$$Cov(X,Y) = \left(\frac{1}{\mathrm{N}}\right)\sum \left(x[n] - \mu_X\right)\left(y[n] - \mu_Y\right) \qquad \text{(S5)}$$

Where $Cov(X,Y)$ is the covariance between signals X and Y, μ_X is the mean value of signal X, and μ_Y is the mean value of signal Y, $x[n]$ and $y[n]$ are amplitudes of signals X and Y, and N is the total number of samples.

Kurtosis: It represents the peakedness of the amplitude of the EMG signal. A higher value of kurtosis shows a more frequent occurrence of extreme values.

$$Kurt(x) = \frac{\left(\frac{1}{\mathrm{N}}\right)\sum (x[n] - \mu)^4}{\sigma^4} \tag{S6}$$

Where $Kurt(x)$ is the Kurtosis of the EMG signal, μ is the mean value of the signal, $x[n]$ is the amplitude of the EMG signal, σ is the standard deviation of the EMG signal, and N is the number of samples.

Skewness: It represents the asymmetry of the EMG signal distribution. It can be either positive or negative, depending on the patterns of the tail when plotted.

$$Skew(x) = \frac{\left(\frac{1}{\mathrm{N}}\right)\sum (x[n] - \mu)^3}{\sigma^3} \tag{S7}$$

Where $Skew(x)$ is the Skewness of the EMG signal, μ is the mean value of the signal, $x[n]$ is the amplitude of the EMG signal, σ is the standard deviation of the EMG signal, and N is the number of samples.

Root Mean Square: It represents the effective value of the EMG signal, which corresponds to the energy of muscle contraction.

$$\mathrm{RMS} = \sqrt{\left(\frac{1}{\mathrm{N}}\right)\sum (x[n])^2} \tag{S8}$$

Where RMS is the root mean square of the EMG signal, $x[n]$ is the amplitude of the EMG signal, and N is the number of samples.

Square Integral: It is the sum of squared amplitudes across the segment of an EMG signal. It represents a cumulative measure of the power of the EMG signal.

$$SI = \sum x[n]^2 \tag{S9}$$

Where SI is the square integral of the EMG signal and $x[n]$ is the amplitude of the EMG signal.

Average Energy: It normalizes the power of the EMG signal per sample and provides a relative measure of activity intensity.

$$E_{avg} = \frac{1}{N}\sum x[n]^2 \tag{S10}$$

Where E_{avg} is the average energy, $x[n]$ is the amplitude of the EMG signal, and N is the number of samples.

Temporal Moment: It shows the distribution of EMG signal energy with respect to time. Low values of temporal moment represent the mean and variance of the amplitude distribution, and high values represent the signal shape and temporal structure. It is important in identifying the muscle fatigue patterns.

$$M_m = \frac{1}{N}\sum (t_n - \mu_t)^{m\, x[n]} \tag{S11}$$

Where M_m is the temporal moment of order m, t_n is the time index of the sample n, μ_t is the mean of time indices, $x[n]$ is the amplitude of the EMG signal, and N is the number of samples.

Willison Amplitude: It shows the number of times the absolute difference between the consecutive samples exceeds a predefined threshold. It is sensitive to changes in the muscle activity and reflects the complexity of the EMG signal.

$$WAMP = \sum f(|x[n+1] - x[n]| \geq \theta) \tag{S12}$$

Where $WAMP$ is the Willison amplitude, $f()$ is the step function (1 if the condition is satisfied, else 0), θ is the threshold value, and $x[n]$ is the amplitude of the EMG signal.

Zero Crossing: It counts the number of times the EMG signal changes sign, representing the oscillatory activity within the muscle. It is also related to the dominant frequency content of the EMG signal.

$$ZC = \sum g(x[n], x[n+1]) \tag{S13}$$

Where ZC is zero crossing count, $g(x[n], x[n+1])$ is the indicator function = 1 if sign change occurs, else 0.

Mean Frequency: It is the average frequency value of the EMG power spectrum. It is weighted by spectral amplitude and provides the information regarding the spectral centroid.

$$MNF = \frac{\sum f_k P_{fk}}{\sum P_{fk}} \tag{S14}$$

Where MNF is the mean frequency, f_k is the frequency bin, and P_{fk} is the power spectral density at frequency f_k.

Median Frequency: It is the frequency that divides the power spectrum into two equal halves.

$$\sum P_{fk}, f \leq f_{med} = \frac{1}{2}\sum P_{fk} \tag{S15}$$

Where f_{med} is the median frequency, P_f is the power spectral density, and the condition is that power below the median frequency = half of the total spectral power.
