# Supplementary figures and images for: Machine Learning-Based Self-Induced Scratch Intensity Detection Using Feature Optimization and Multi-Channel Electromyogram Signals for Prevention of Lichenification (part 2 of 2)
Source: Bioengineering (Basel). 2026 Jul 8;13(7):787. doi: 10.3390/bioengineering13070787 (PMC13405920; doi:10.3390/bioengineering13070787)

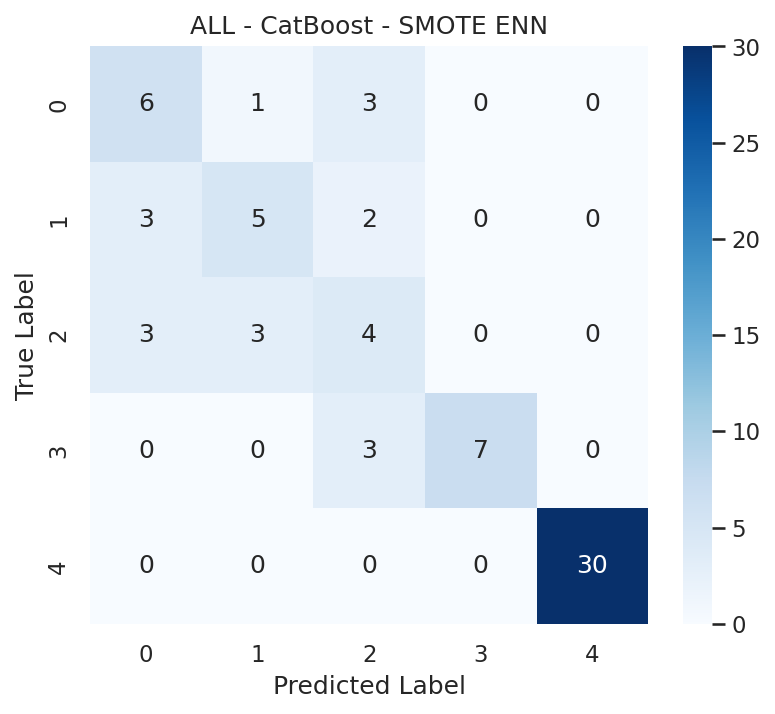

Supplement: Supplementary file 1 [file bioengineering-13-00787-s001.zip › Supplementary Material - Performance Metrics/cm_ALL_CatBoost_SMOTE ENN.png]

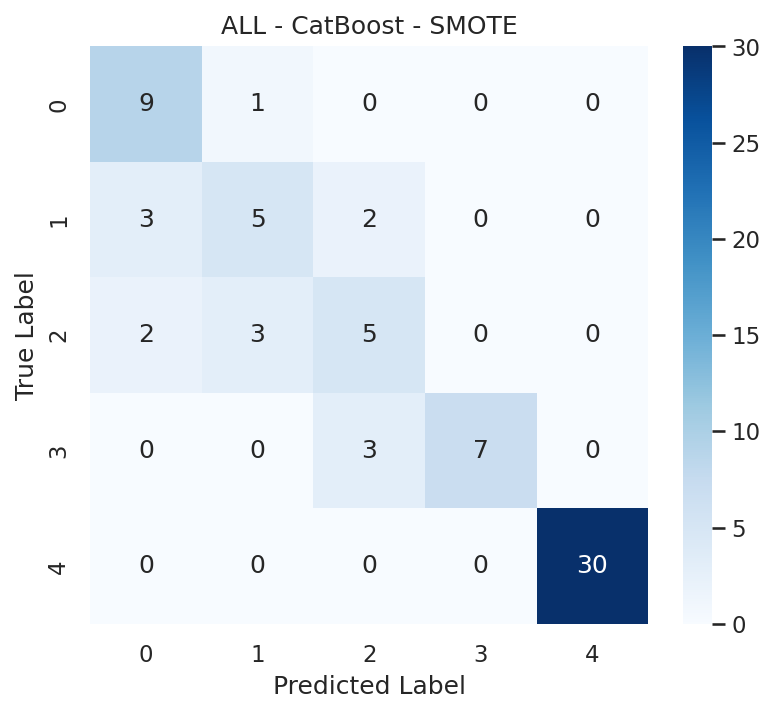

Supplement: Supplementary file 1 [file bioengineering-13-00787-s001.zip › Supplementary Material - Performance Metrics/cm_ALL_CatBoost_SMOTE.png]

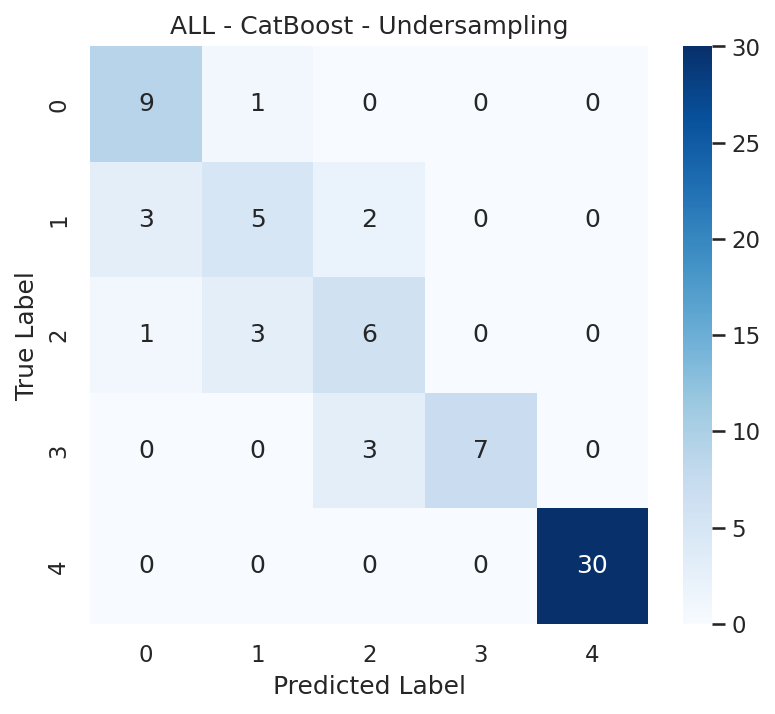

Supplement: Supplementary file 1 [file bioengineering-13-00787-s001.zip › Supplementary Material - Performance Metrics/cm_ALL_CatBoost_Undersampling.png]

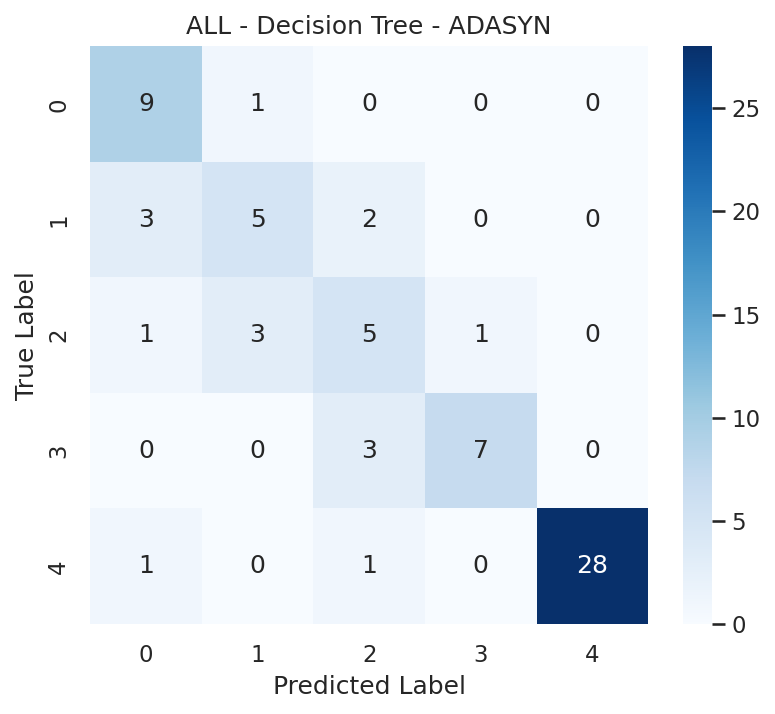

Supplement: Supplementary file 1 [file bioengineering-13-00787-s001.zip › Supplementary Material - Performance Metrics/cm_ALL_Decision Tree_ADASYN.png]

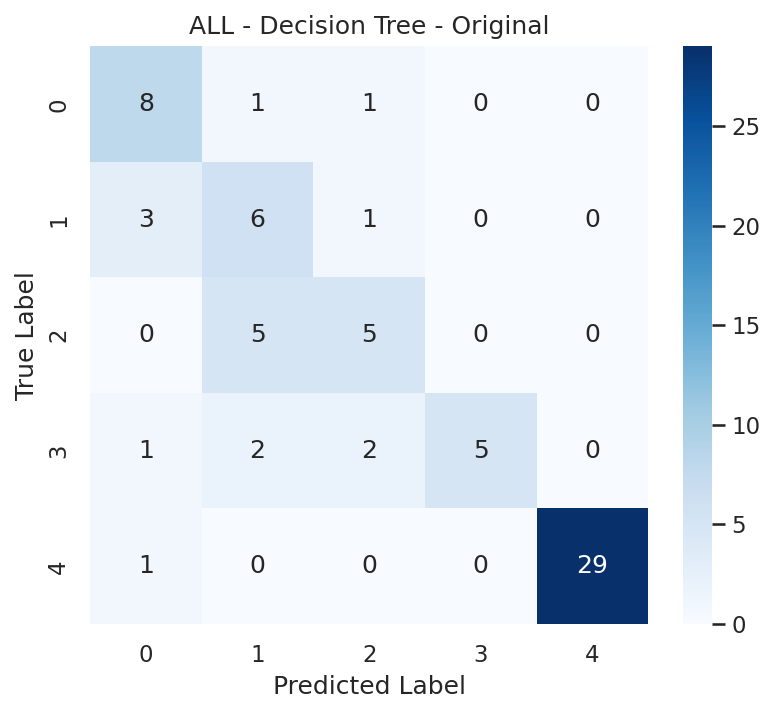

Supplement: Supplementary file 1 [file bioengineering-13-00787-s001.zip › Supplementary Material - Performance Metrics/cm_ALL_Decision Tree_Original.png]

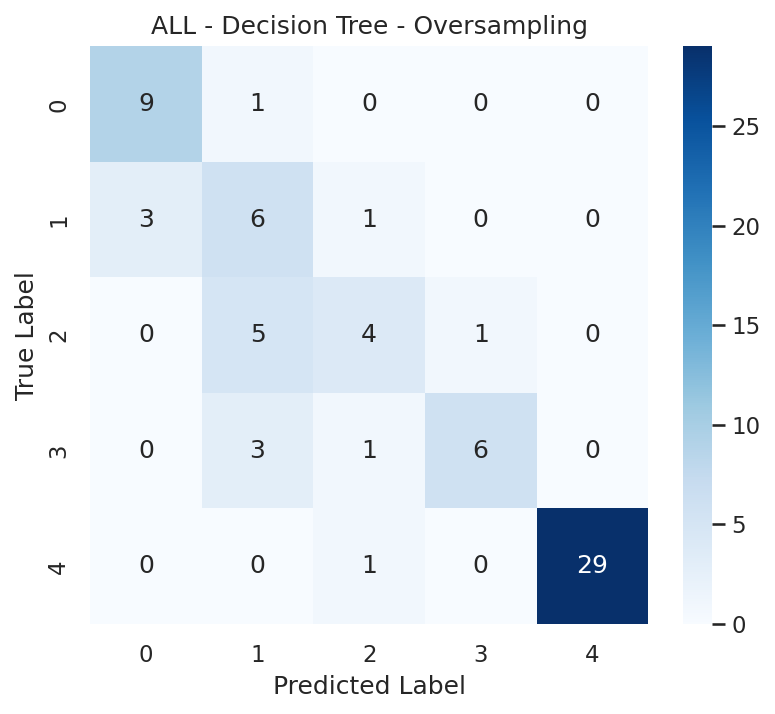

Supplement: Supplementary file 1 [file bioengineering-13-00787-s001.zip › Supplementary Material - Performance Metrics/cm_ALL_Decision Tree_Oversampling.png]

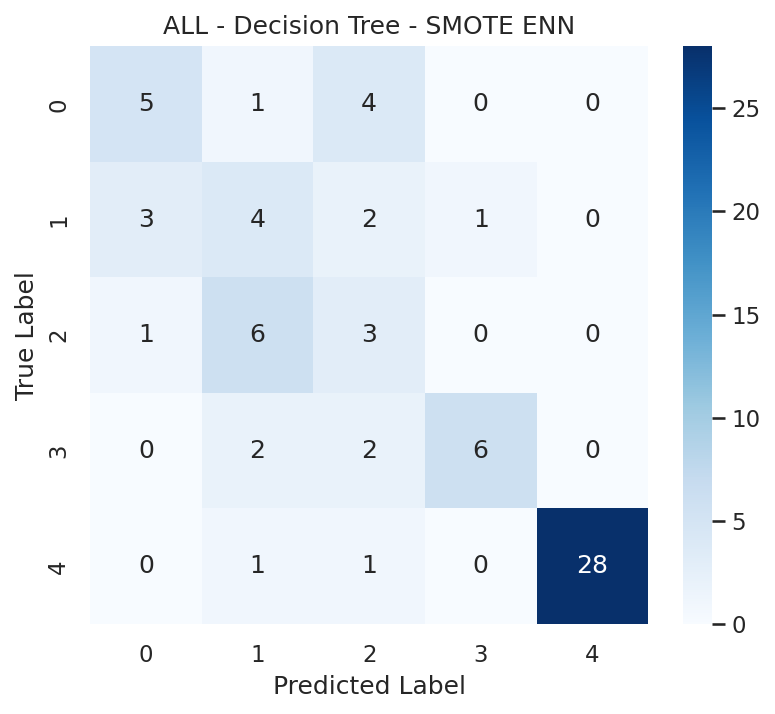

Supplement: Supplementary file 1 [file bioengineering-13-00787-s001.zip › Supplementary Material - Performance Metrics/cm_ALL_Decision Tree_SMOTE ENN.png]

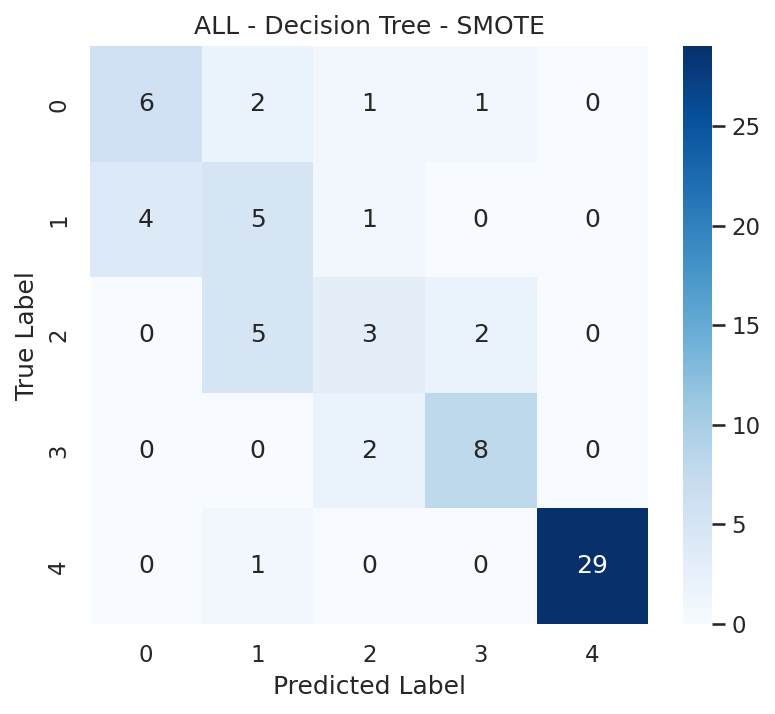

Supplement: Supplementary file 1 [file bioengineering-13-00787-s001.zip › Supplementary Material - Performance Metrics/cm_ALL_Decision Tree_SMOTE.png]

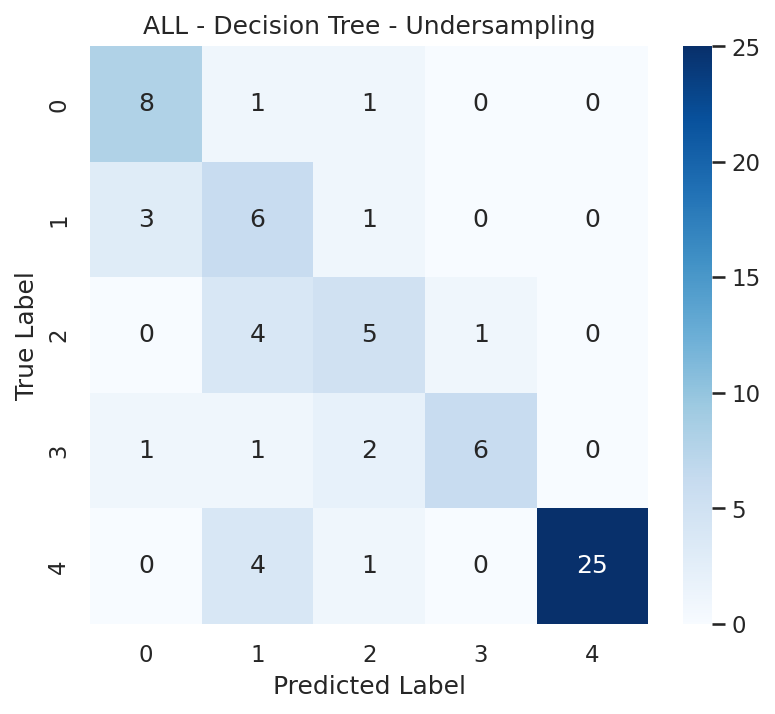

Supplement: Supplementary file 1 [file bioengineering-13-00787-s001.zip › Supplementary Material - Performance Metrics/cm_ALL_Decision Tree_Undersampling.png]

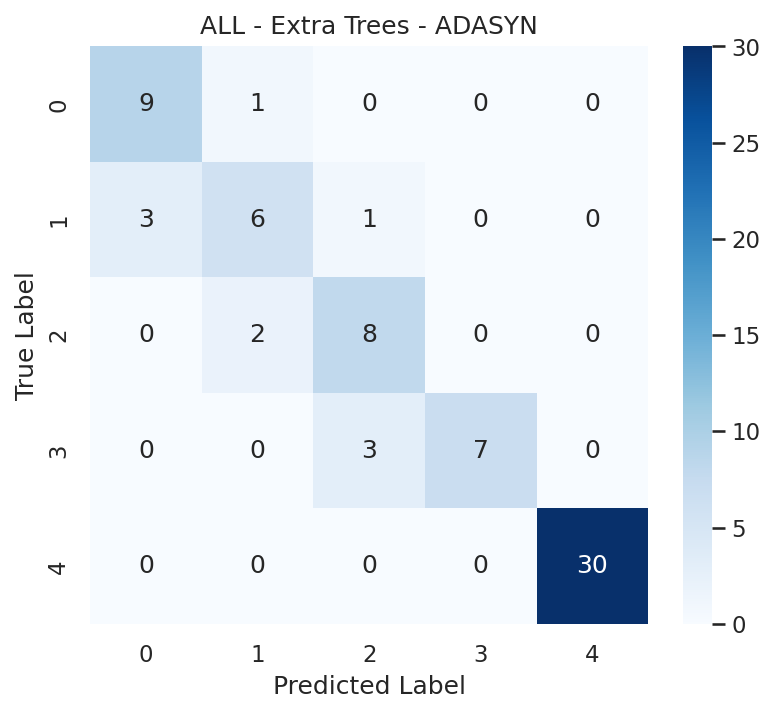

Supplement: Supplementary file 1 [file bioengineering-13-00787-s001.zip › Supplementary Material - Performance Metrics/cm_ALL_Extra Trees_ADASYN.png]

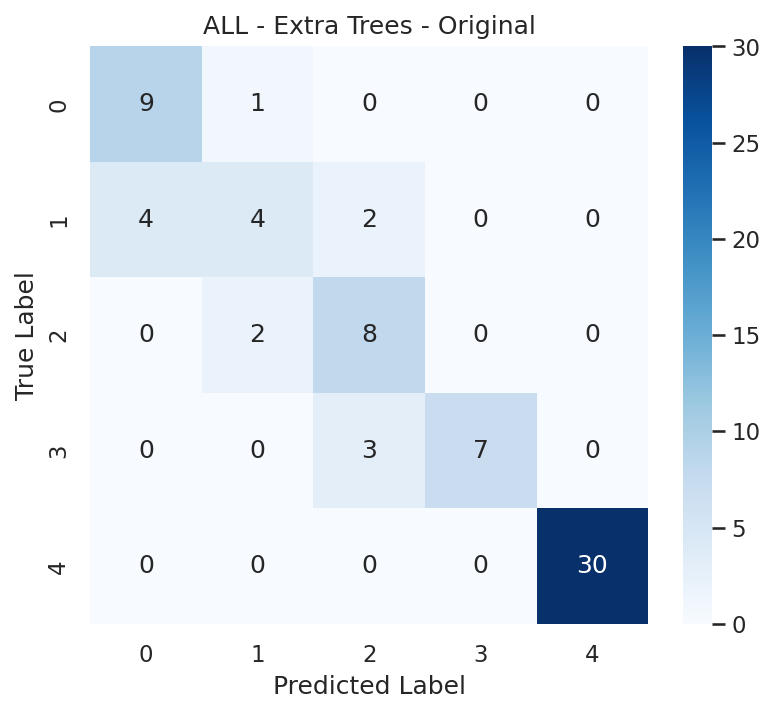

Supplement: Supplementary file 1 [file bioengineering-13-00787-s001.zip › Supplementary Material - Performance Metrics/cm_ALL_Extra Trees_Original.png]

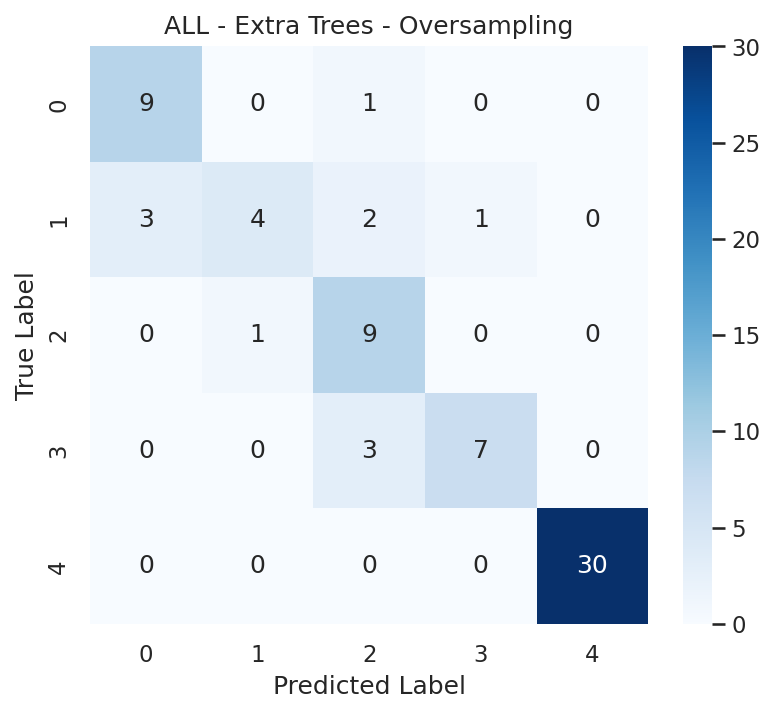

Supplement: Supplementary file 1 [file bioengineering-13-00787-s001.zip › Supplementary Material - Performance Metrics/cm_ALL_Extra Trees_Oversampling.png]

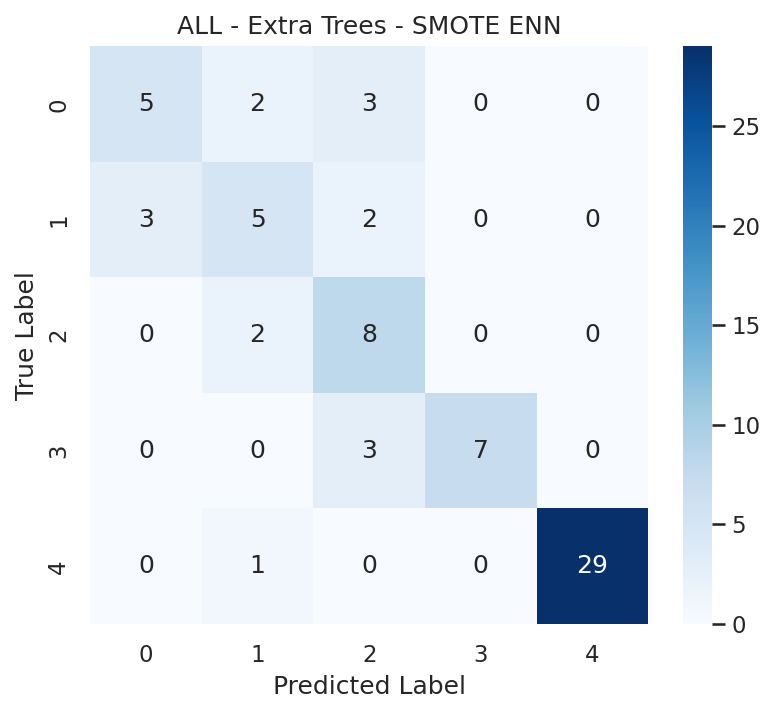

Supplement: Supplementary file 1 [file bioengineering-13-00787-s001.zip › Supplementary Material - Performance Metrics/cm_ALL_Extra Trees_SMOTE ENN.png]

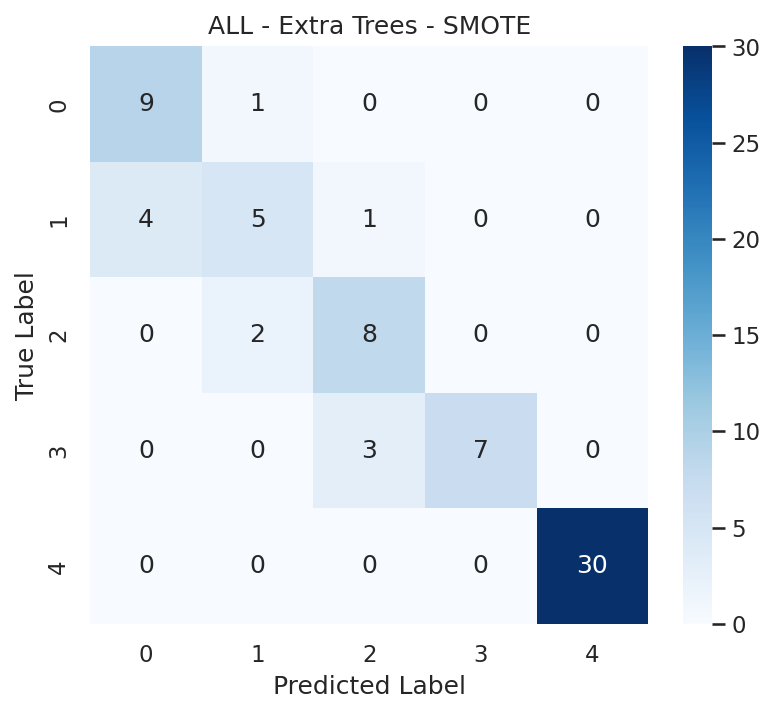

Supplement: Supplementary file 1 [file bioengineering-13-00787-s001.zip › Supplementary Material - Performance Metrics/cm_ALL_Extra Trees_SMOTE.png]

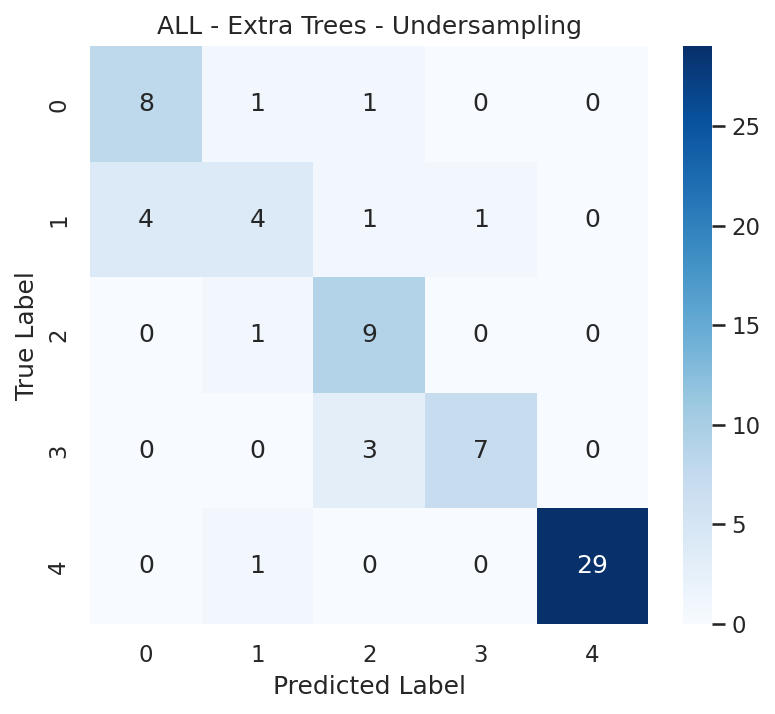

Supplement: Supplementary file 1 [file bioengineering-13-00787-s001.zip › Supplementary Material - Performance Metrics/cm_ALL_Extra Trees_Undersampling.png]

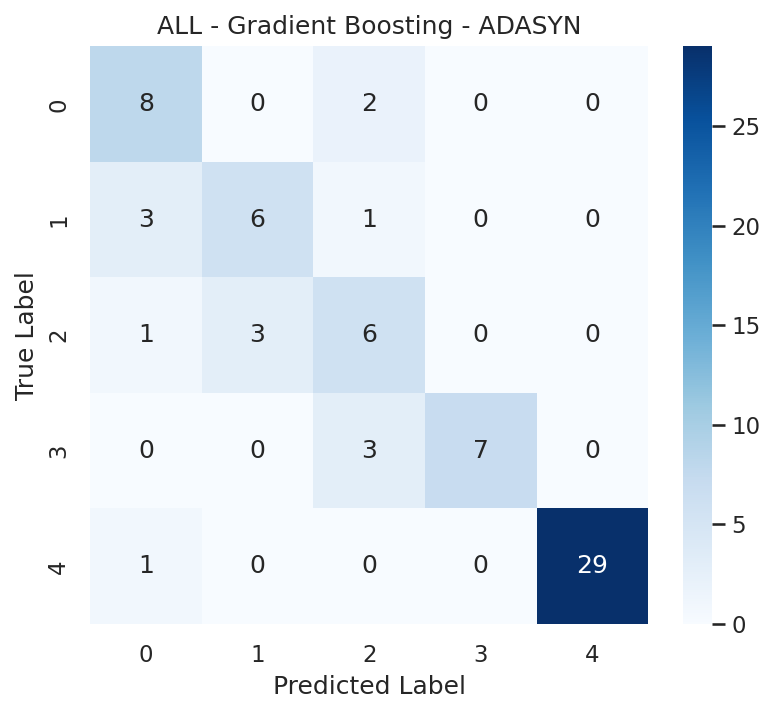

Supplement: Supplementary file 1 [file bioengineering-13-00787-s001.zip › Supplementary Material - Performance Metrics/cm_ALL_Gradient Boosting_ADASYN.png]

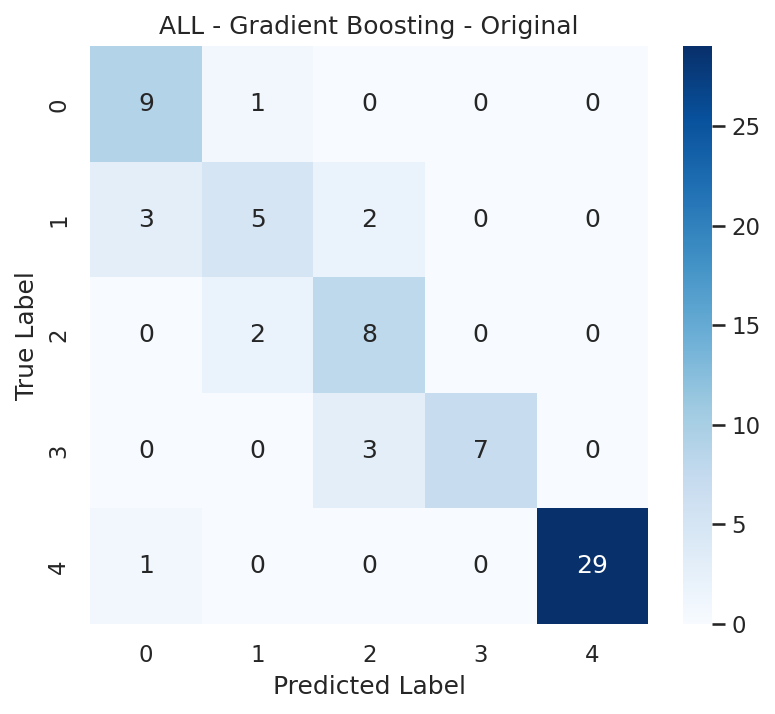

Supplement: Supplementary file 1 [file bioengineering-13-00787-s001.zip › Supplementary Material - Performance Metrics/cm_ALL_Gradient Boosting_Original.png]

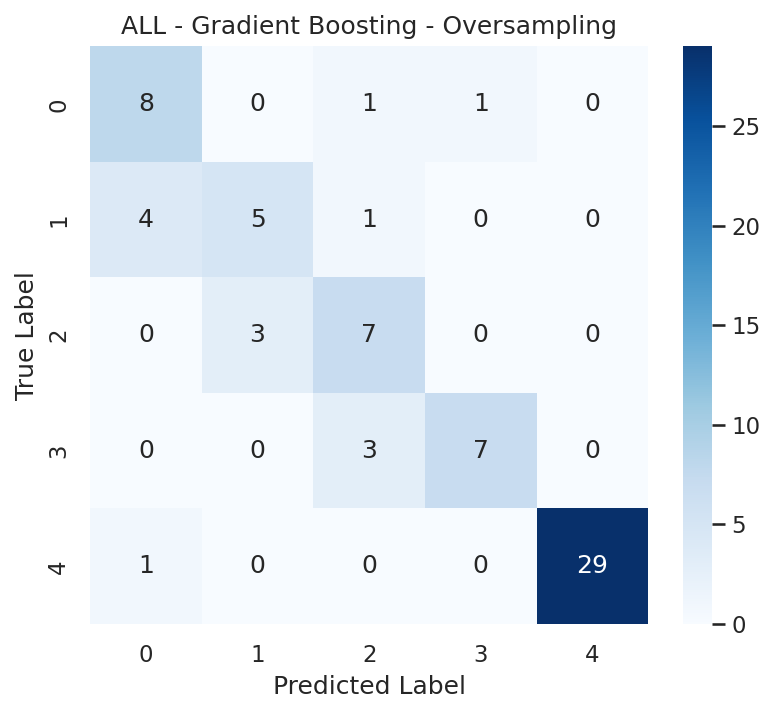

Supplement: Supplementary file 1 [file bioengineering-13-00787-s001.zip › Supplementary Material - Performance Metrics/cm_ALL_Gradient Boosting_Oversampling.png]

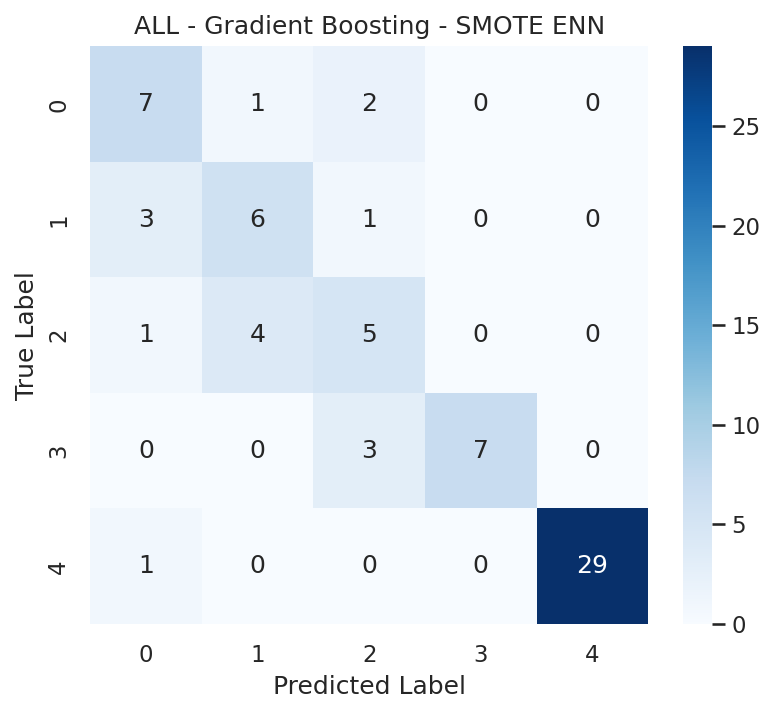

Supplement: Supplementary file 1 [file bioengineering-13-00787-s001.zip › Supplementary Material - Performance Metrics/cm_ALL_Gradient Boosting_SMOTE ENN.png]

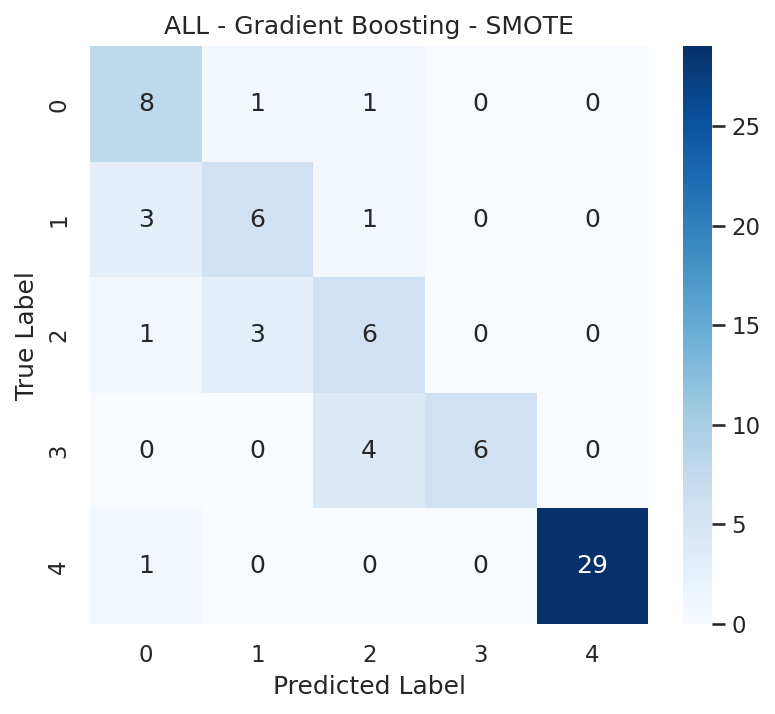

Supplement: Supplementary file 1 [file bioengineering-13-00787-s001.zip › Supplementary Material - Performance Metrics/cm_ALL_Gradient Boosting_SMOTE.png]

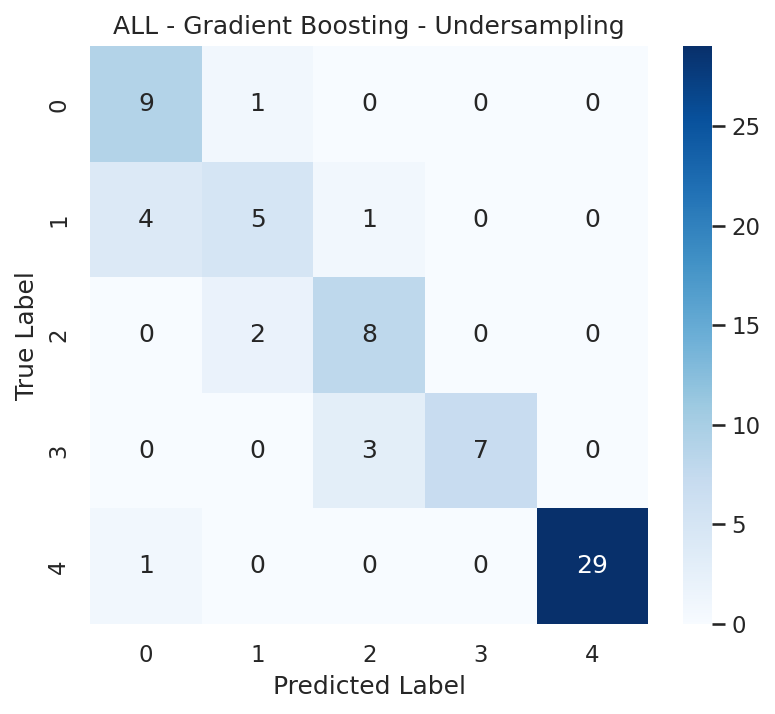

Supplement: Supplementary file 1 [file bioengineering-13-00787-s001.zip › Supplementary Material - Performance Metrics/cm_ALL_Gradient Boosting_Undersampling.png]

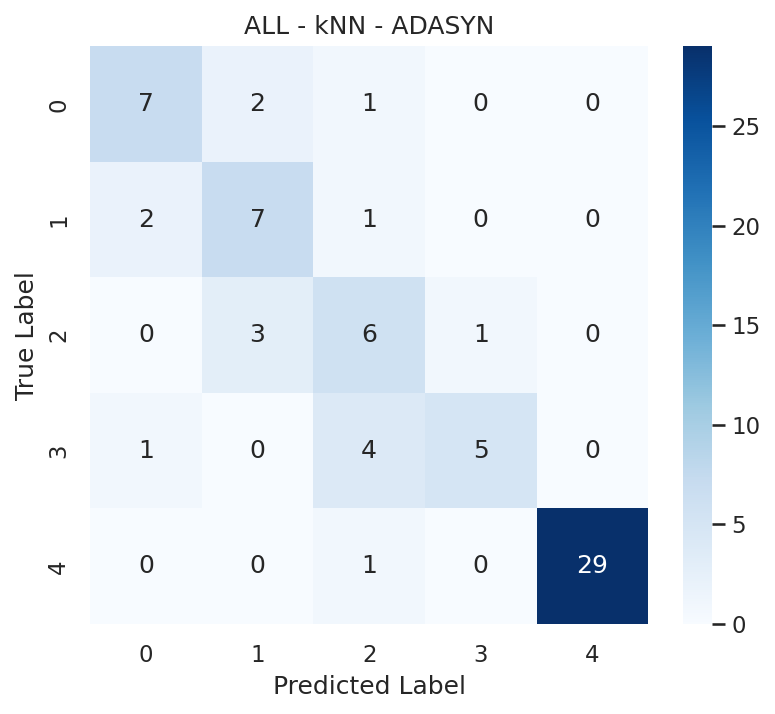

Supplement: Supplementary file 1 [file bioengineering-13-00787-s001.zip › Supplementary Material - Performance Metrics/cm_ALL_kNN_ADASYN.png]

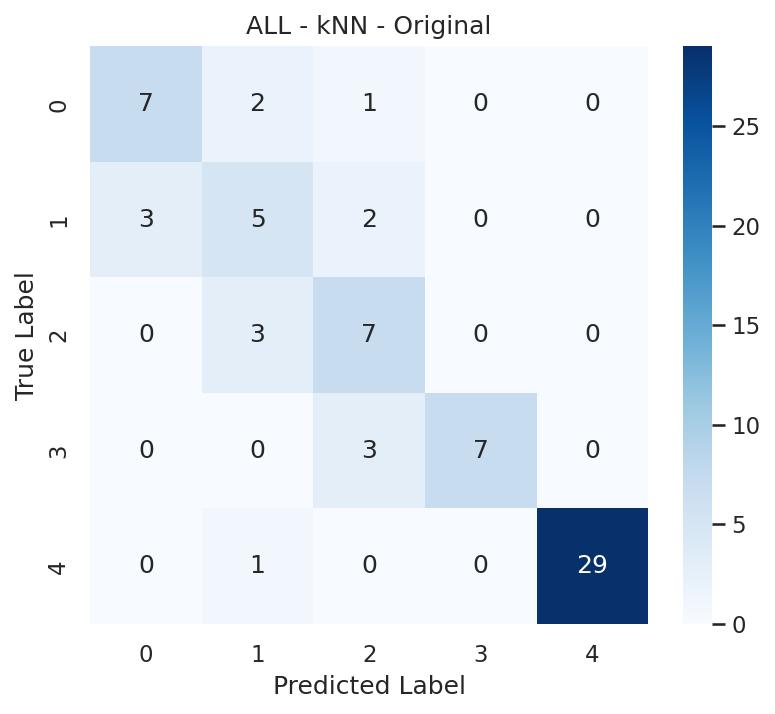

Supplement: Supplementary file 1 [file bioengineering-13-00787-s001.zip › Supplementary Material - Performance Metrics/cm_ALL_kNN_Original.png]

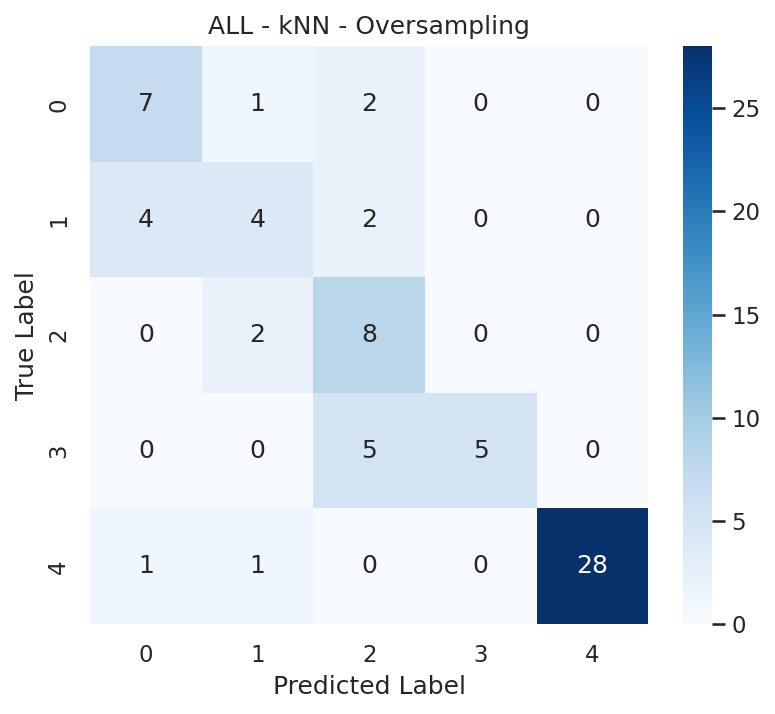

Supplement: Supplementary file 1 [file bioengineering-13-00787-s001.zip › Supplementary Material - Performance Metrics/cm_ALL_kNN_Oversampling.png]

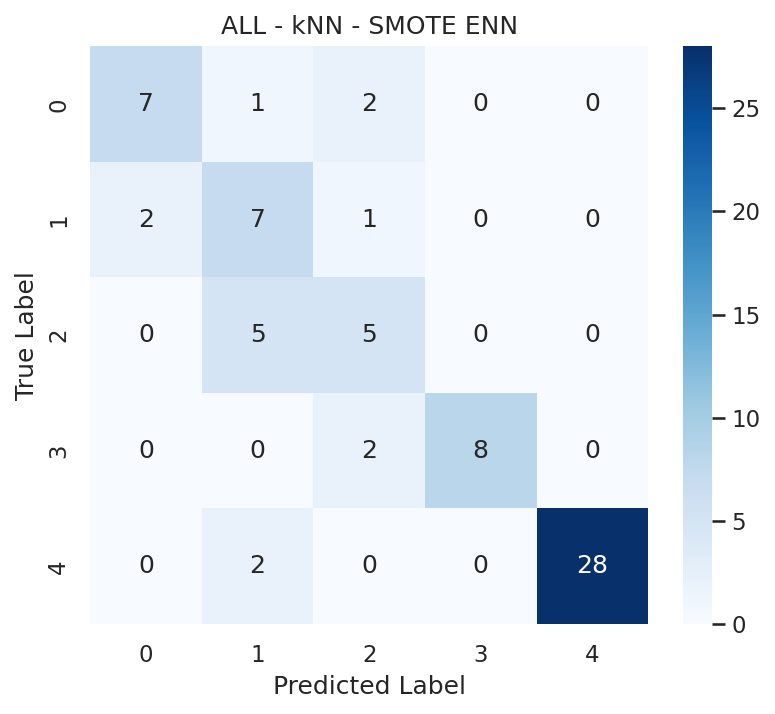

Supplement: Supplementary file 1 [file bioengineering-13-00787-s001.zip › Supplementary Material - Performance Metrics/cm_ALL_kNN_SMOTE ENN.png]

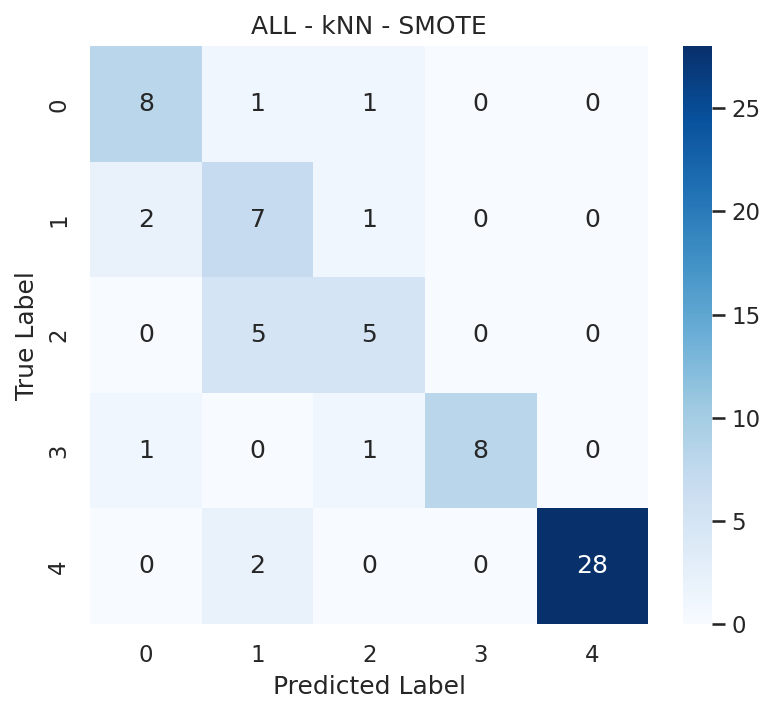

Supplement: Supplementary file 1 [file bioengineering-13-00787-s001.zip › Supplementary Material - Performance Metrics/cm_ALL_kNN_SMOTE.png]

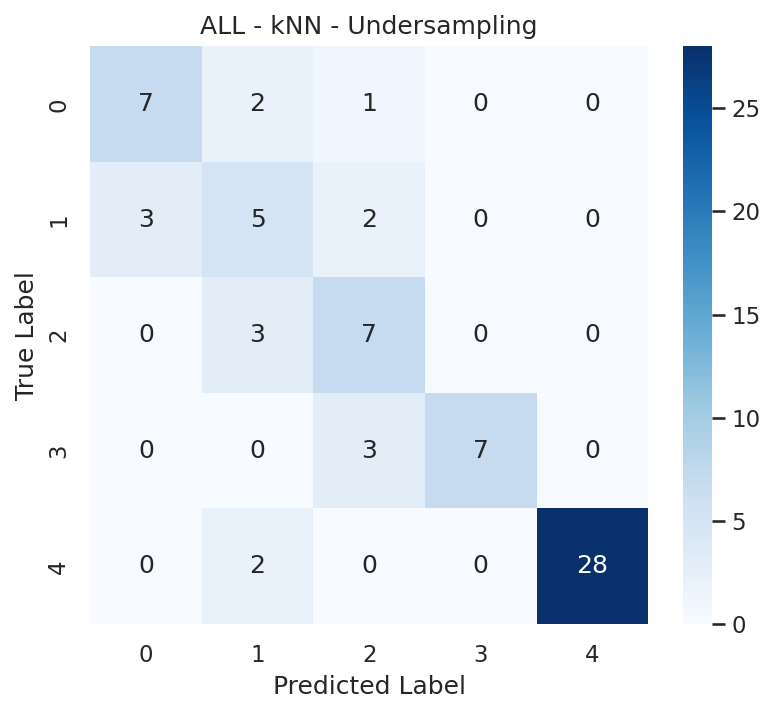

Supplement: Supplementary file 1 [file bioengineering-13-00787-s001.zip › Supplementary Material - Performance Metrics/cm_ALL_kNN_Undersampling.png]

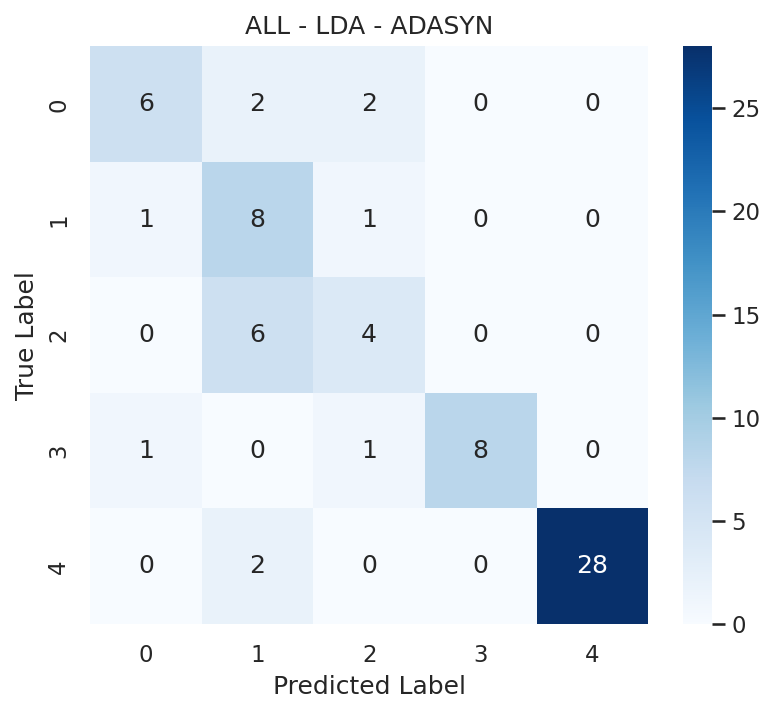

Supplement: Supplementary file 1 [file bioengineering-13-00787-s001.zip › Supplementary Material - Performance Metrics/cm_ALL_LDA_ADASYN.png]

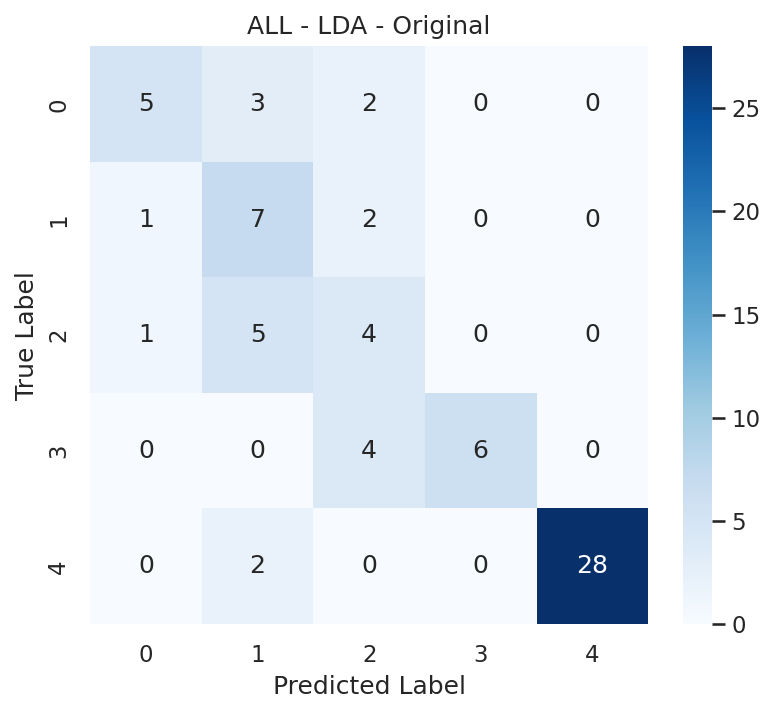

Supplement: Supplementary file 1 [file bioengineering-13-00787-s001.zip › Supplementary Material - Performance Metrics/cm_ALL_LDA_Original.png]

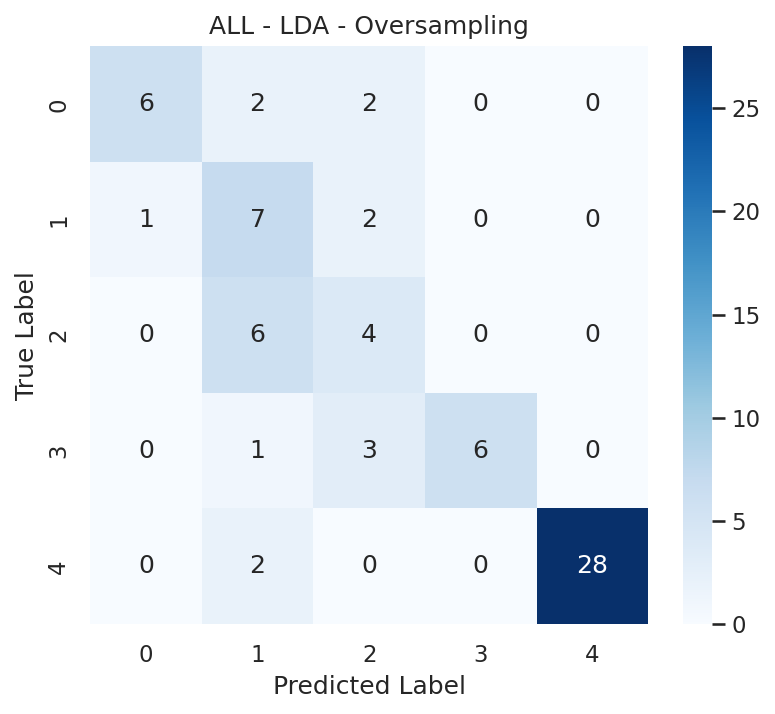

Supplement: Supplementary file 1 [file bioengineering-13-00787-s001.zip › Supplementary Material - Performance Metrics/cm_ALL_LDA_Oversampling.png]

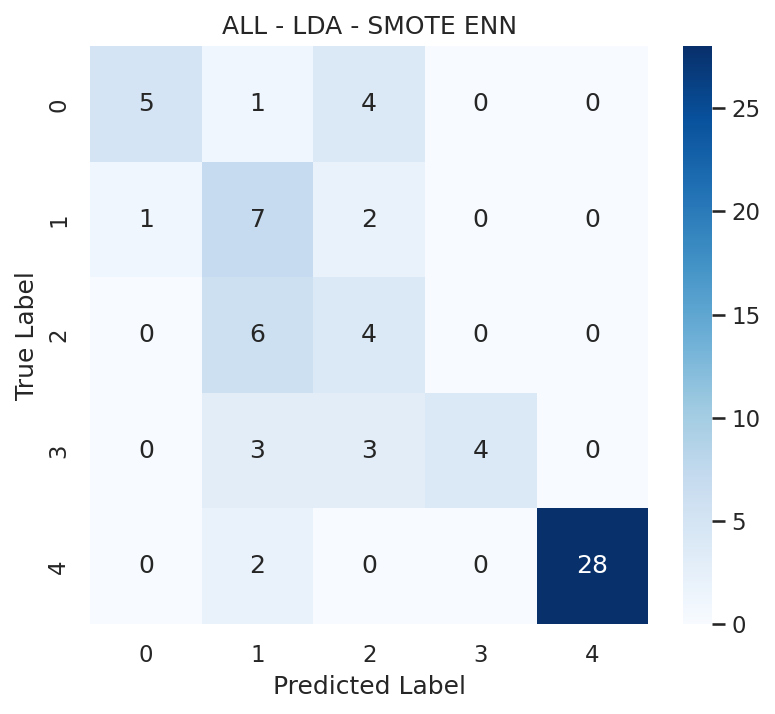

Supplement: Supplementary file 1 [file bioengineering-13-00787-s001.zip › Supplementary Material - Performance Metrics/cm_ALL_LDA_SMOTE ENN.png]

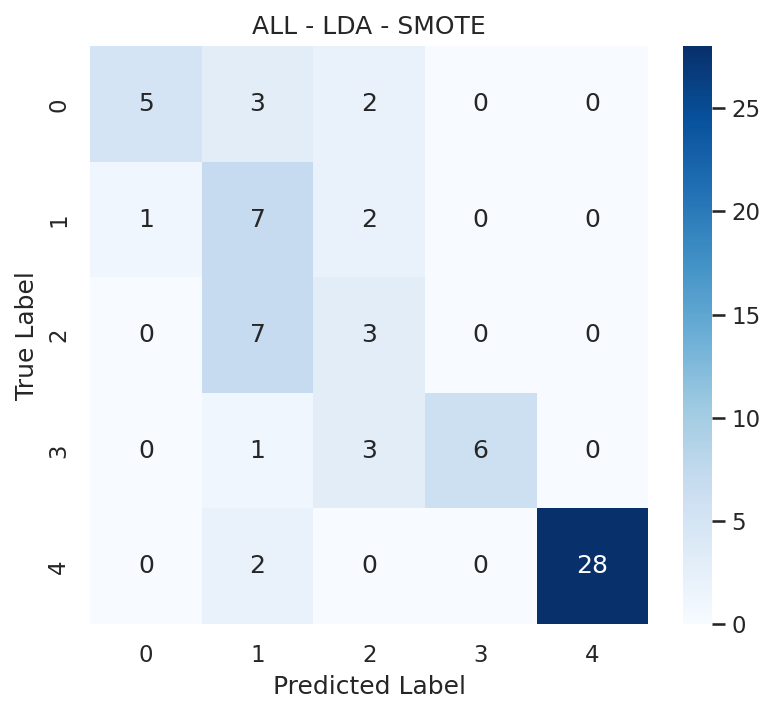

Supplement: Supplementary file 1 [file bioengineering-13-00787-s001.zip › Supplementary Material - Performance Metrics/cm_ALL_LDA_SMOTE.png]

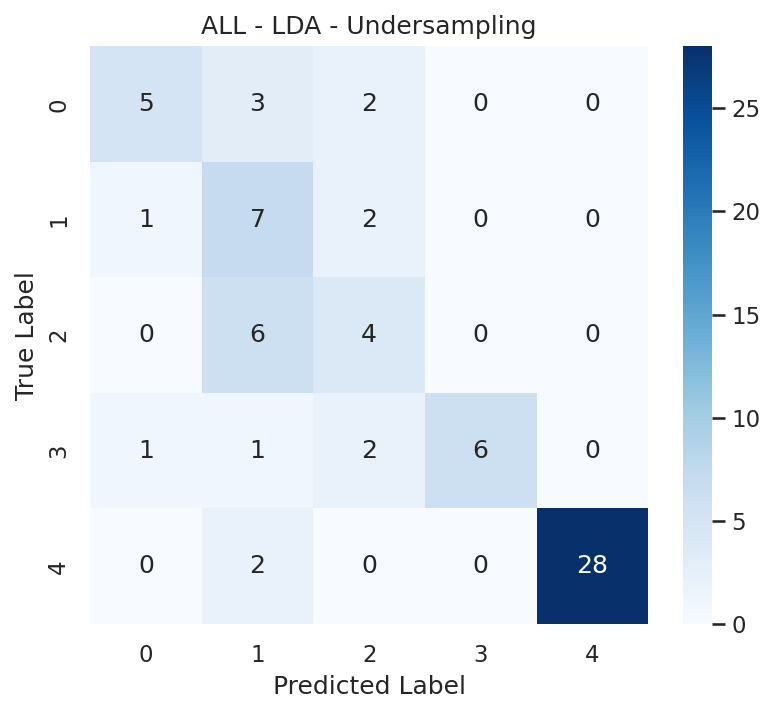

Supplement: Supplementary file 1 [file bioengineering-13-00787-s001.zip › Supplementary Material - Performance Metrics/cm_ALL_LDA_Undersampling.png]

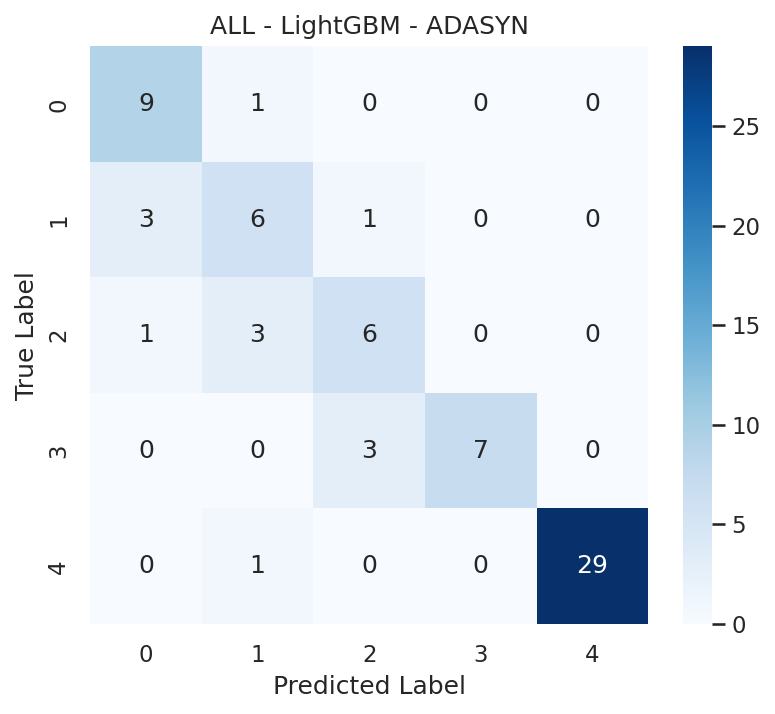

Supplement: Supplementary file 1 [file bioengineering-13-00787-s001.zip › Supplementary Material - Performance Metrics/cm_ALL_LightGBM_ADASYN.png]

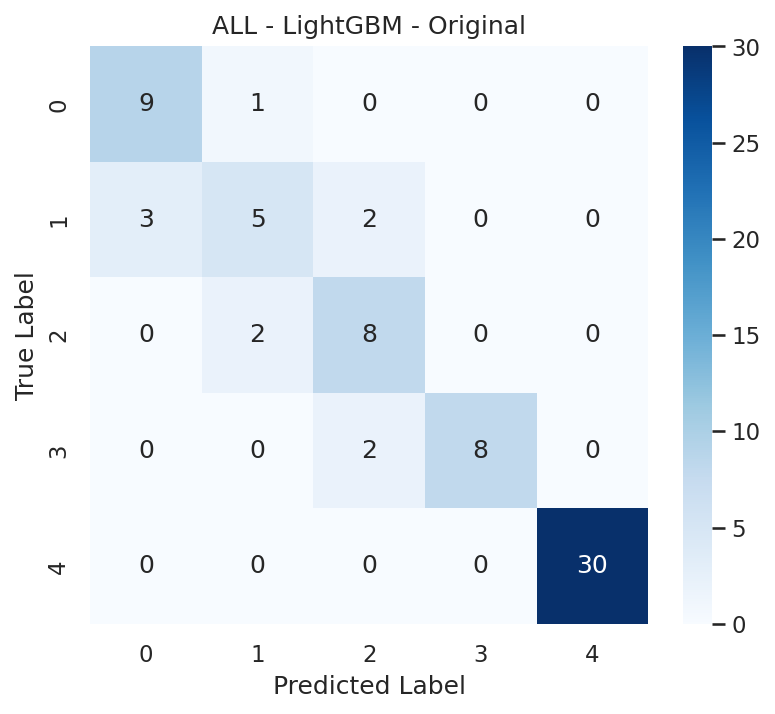

Supplement: Supplementary file 1 [file bioengineering-13-00787-s001.zip › Supplementary Material - Performance Metrics/cm_ALL_LightGBM_Original.png]

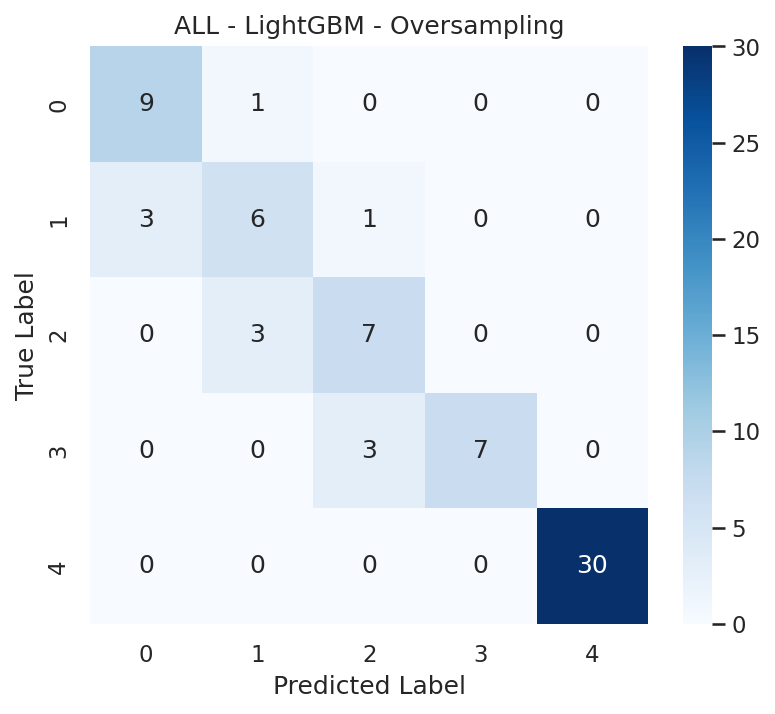

Supplement: Supplementary file 1 [file bioengineering-13-00787-s001.zip › Supplementary Material - Performance Metrics/cm_ALL_LightGBM_Oversampling.png]

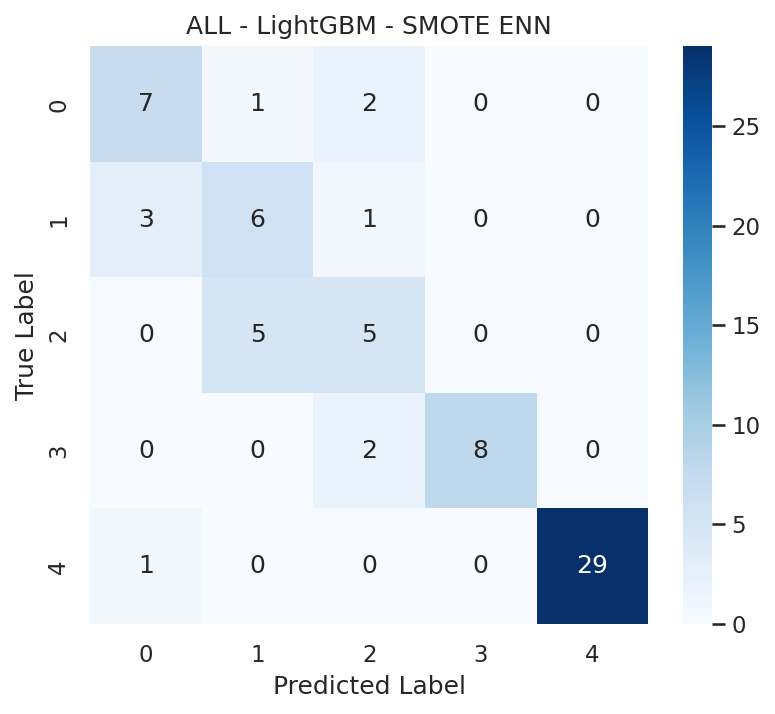

Supplement: Supplementary file 1 [file bioengineering-13-00787-s001.zip › Supplementary Material - Performance Metrics/cm_ALL_LightGBM_SMOTE ENN.png]

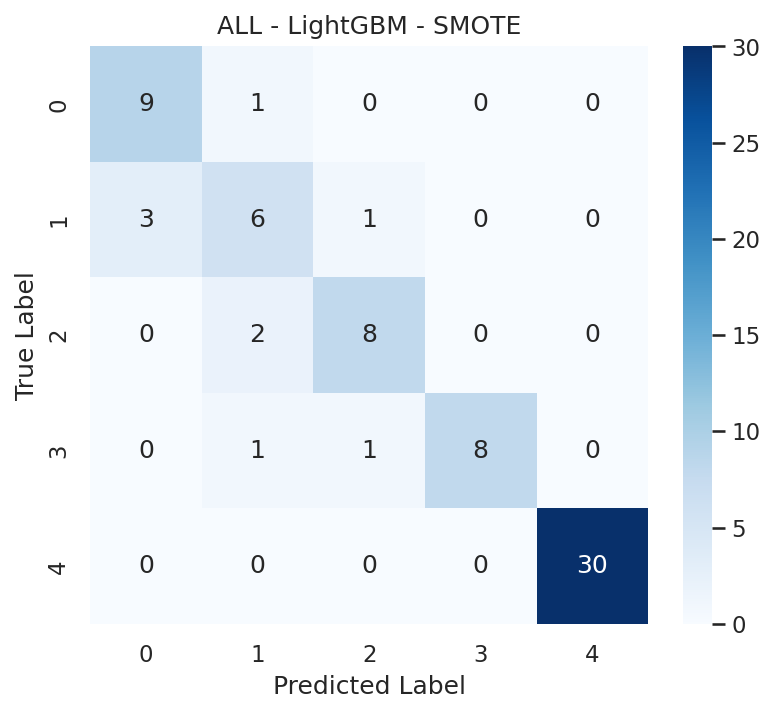

Supplement: Supplementary file 1 [file bioengineering-13-00787-s001.zip › Supplementary Material - Performance Metrics/cm_ALL_LightGBM_SMOTE.png]

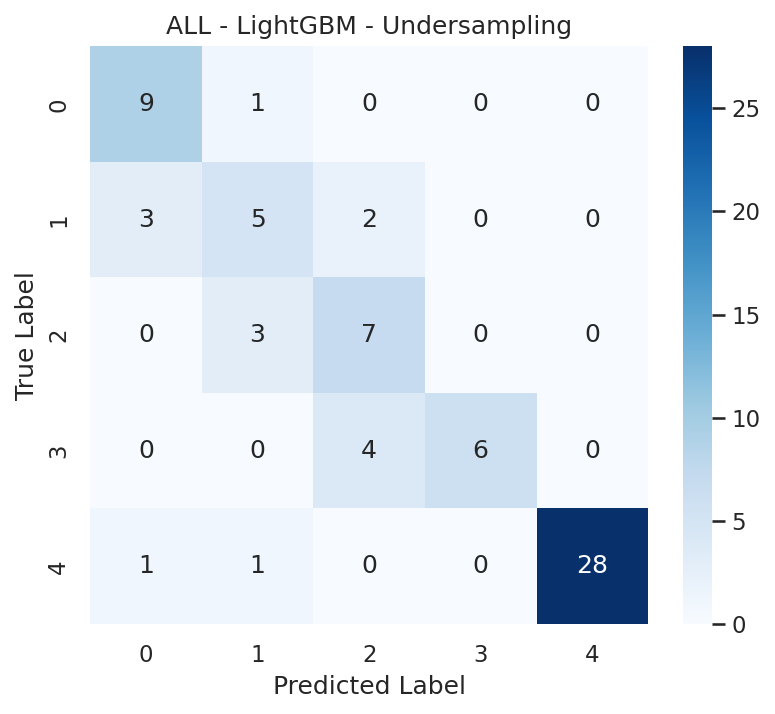

Supplement: Supplementary file 1 [file bioengineering-13-00787-s001.zip › Supplementary Material - Performance Metrics/cm_ALL_LightGBM_Undersampling.png]

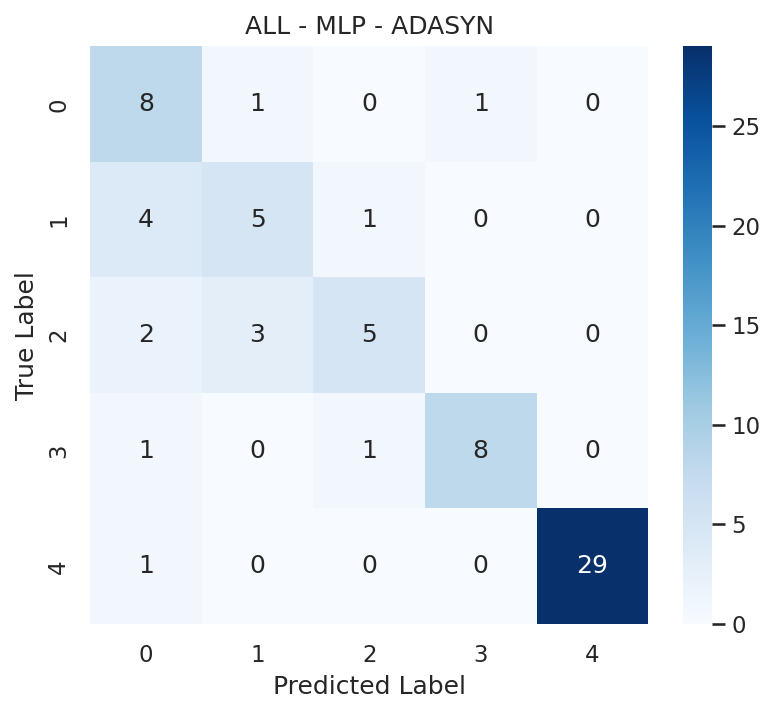

Supplement: Supplementary file 1 [file bioengineering-13-00787-s001.zip › Supplementary Material - Performance Metrics/cm_ALL_MLP_ADASYN.png]

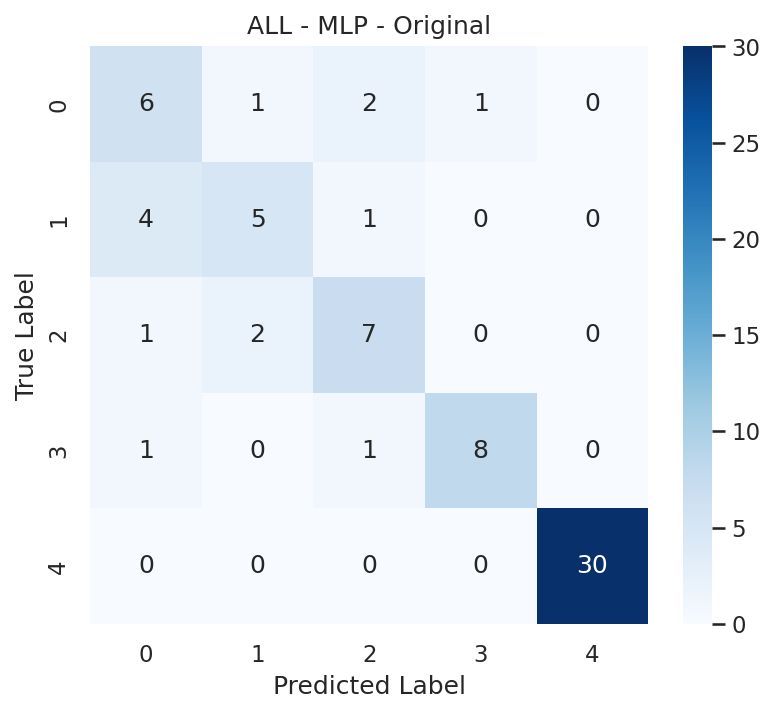

Supplement: Supplementary file 1 [file bioengineering-13-00787-s001.zip › Supplementary Material - Performance Metrics/cm_ALL_MLP_Original.png]

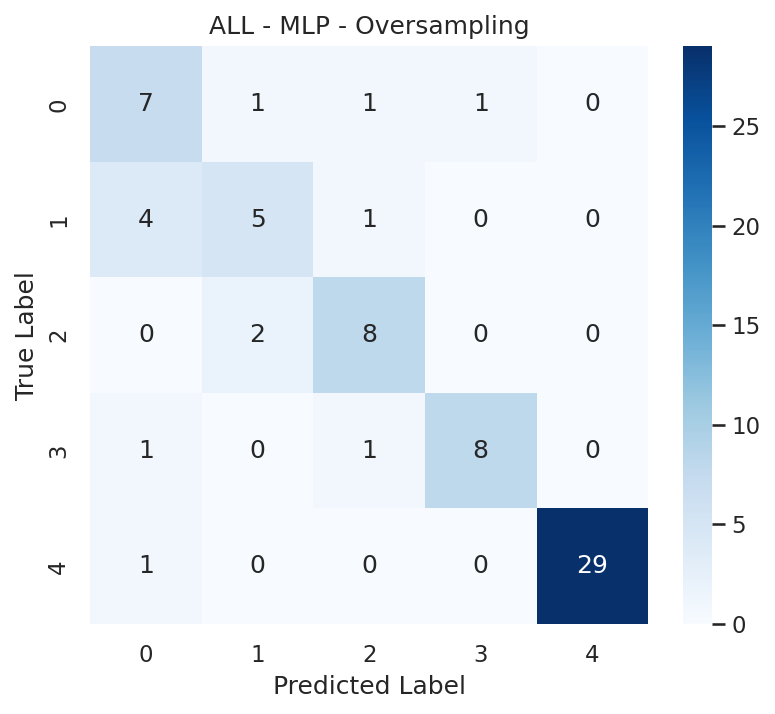

Supplement: Supplementary file 1 [file bioengineering-13-00787-s001.zip › Supplementary Material - Performance Metrics/cm_ALL_MLP_Oversampling.png]

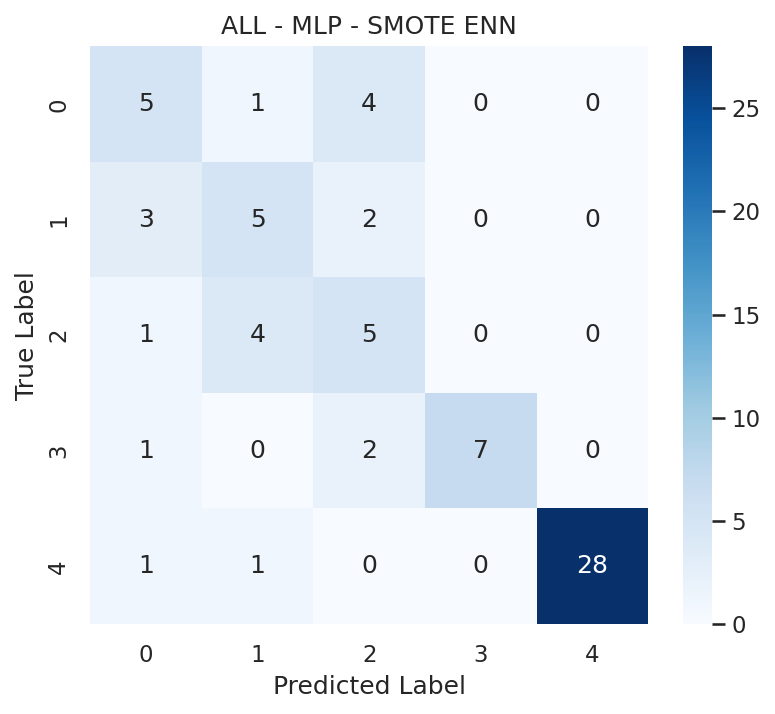

Supplement: Supplementary file 1 [file bioengineering-13-00787-s001.zip › Supplementary Material - Performance Metrics/cm_ALL_MLP_SMOTE ENN.png]

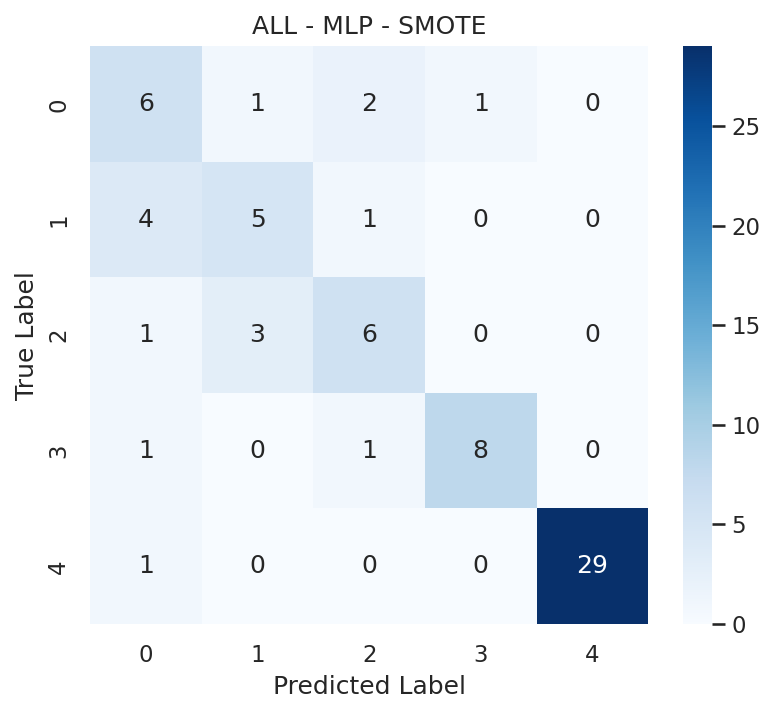

Supplement: Supplementary file 1 [file bioengineering-13-00787-s001.zip › Supplementary Material - Performance Metrics/cm_ALL_MLP_SMOTE.png]

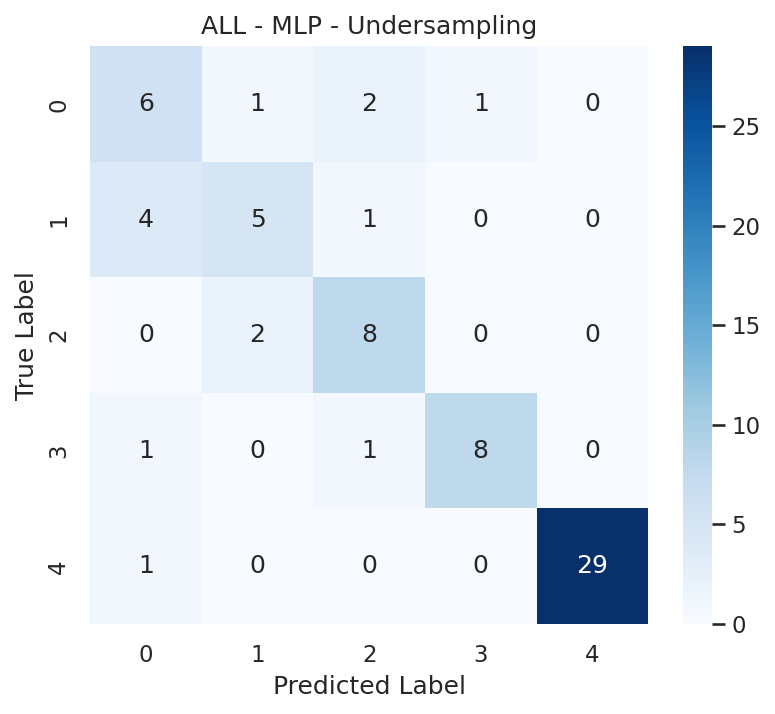

Supplement: Supplementary file 1 [file bioengineering-13-00787-s001.zip › Supplementary Material - Performance Metrics/cm_ALL_MLP_Undersampling.png]

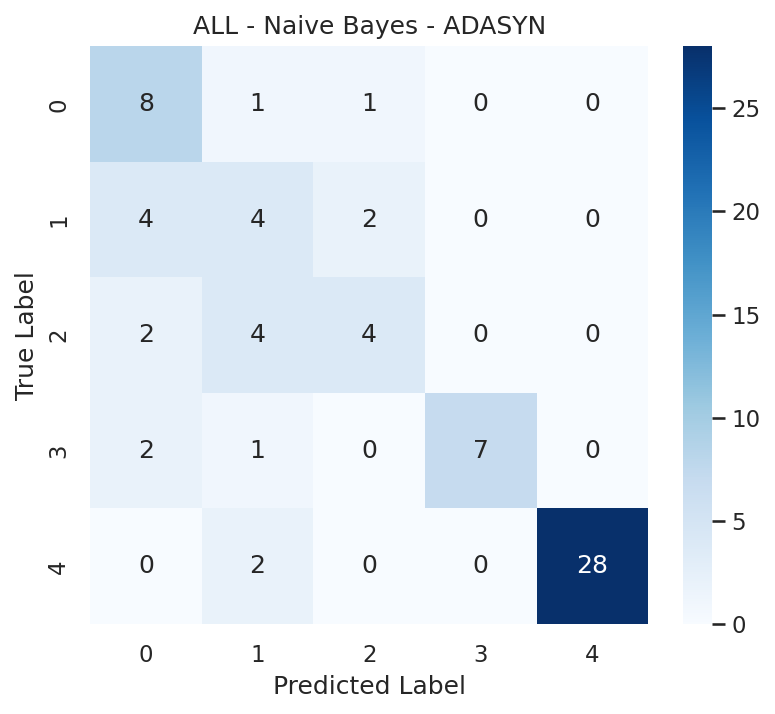

Supplement: Supplementary file 1 [file bioengineering-13-00787-s001.zip › Supplementary Material - Performance Metrics/cm_ALL_Naive Bayes_ADASYN.png]

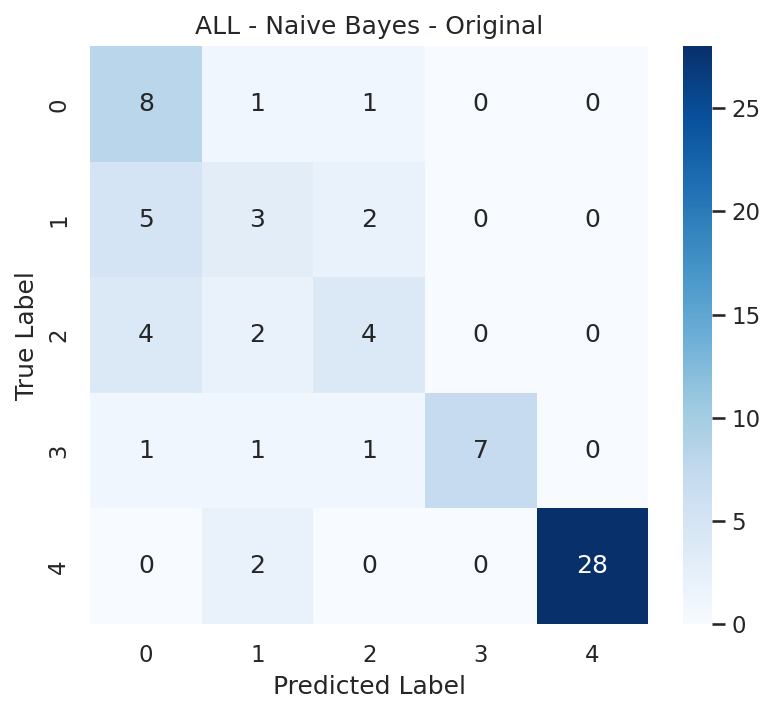

Supplement: Supplementary file 1 [file bioengineering-13-00787-s001.zip › Supplementary Material - Performance Metrics/cm_ALL_Naive Bayes_Original.png]

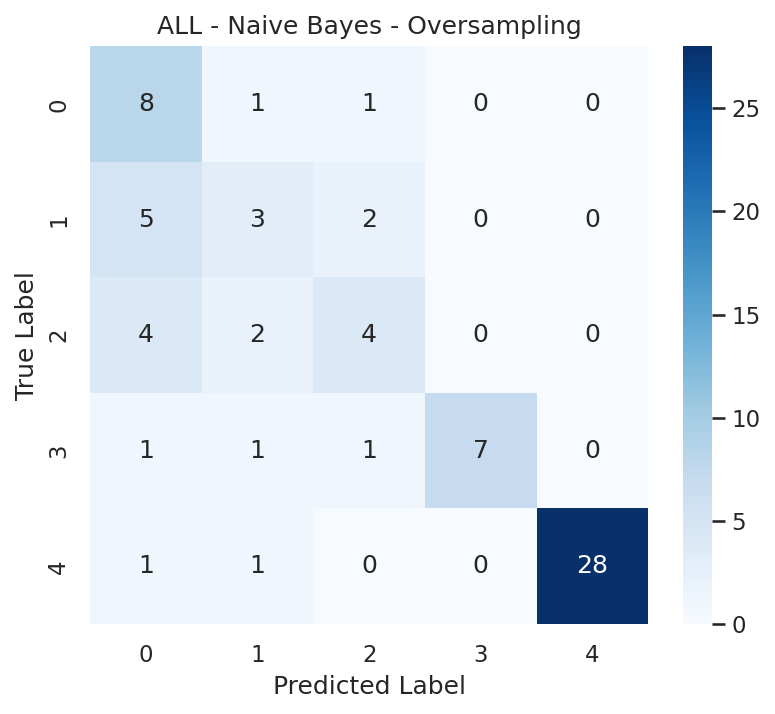

Supplement: Supplementary file 1 [file bioengineering-13-00787-s001.zip › Supplementary Material - Performance Metrics/cm_ALL_Naive Bayes_Oversampling.png]

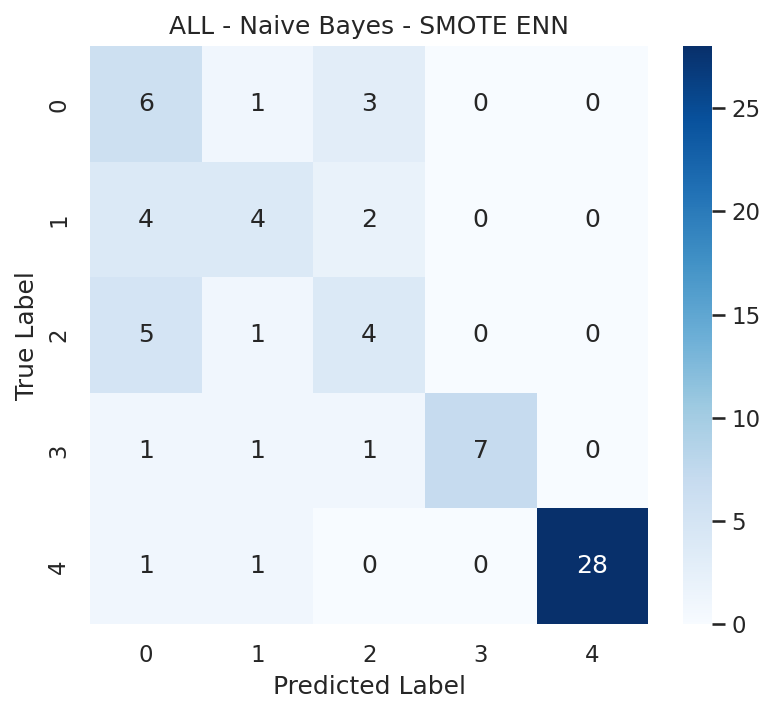

Supplement: Supplementary file 1 [file bioengineering-13-00787-s001.zip › Supplementary Material - Performance Metrics/cm_ALL_Naive Bayes_SMOTE ENN.png]

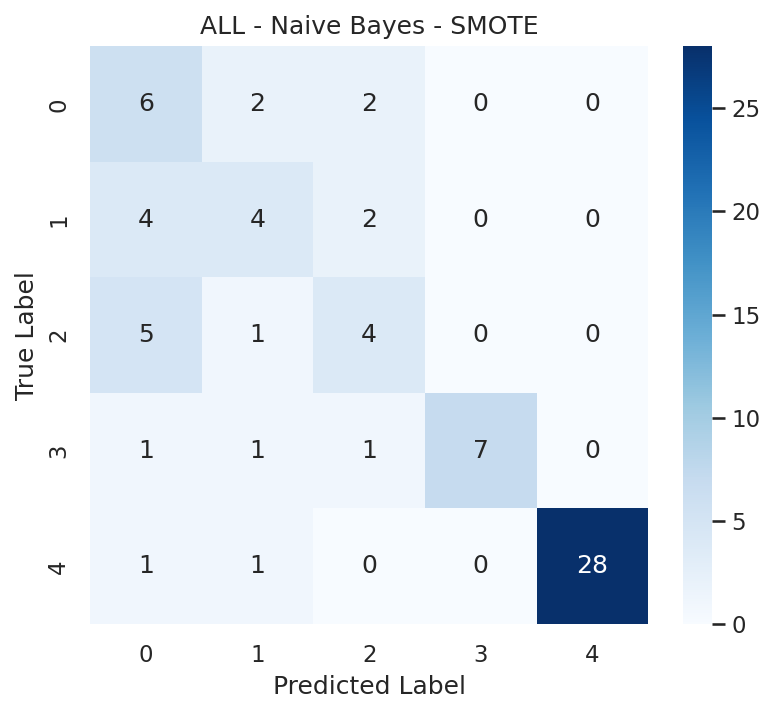

Supplement: Supplementary file 1 [file bioengineering-13-00787-s001.zip › Supplementary Material - Performance Metrics/cm_ALL_Naive Bayes_SMOTE.png]

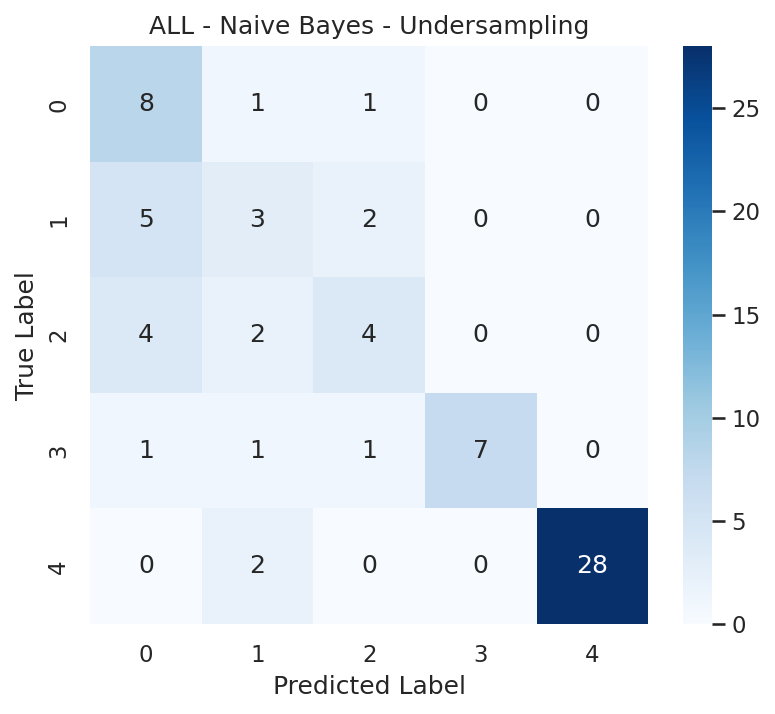

Supplement: Supplementary file 1 [file bioengineering-13-00787-s001.zip › Supplementary Material - Performance Metrics/cm_ALL_Naive Bayes_Undersampling.png]

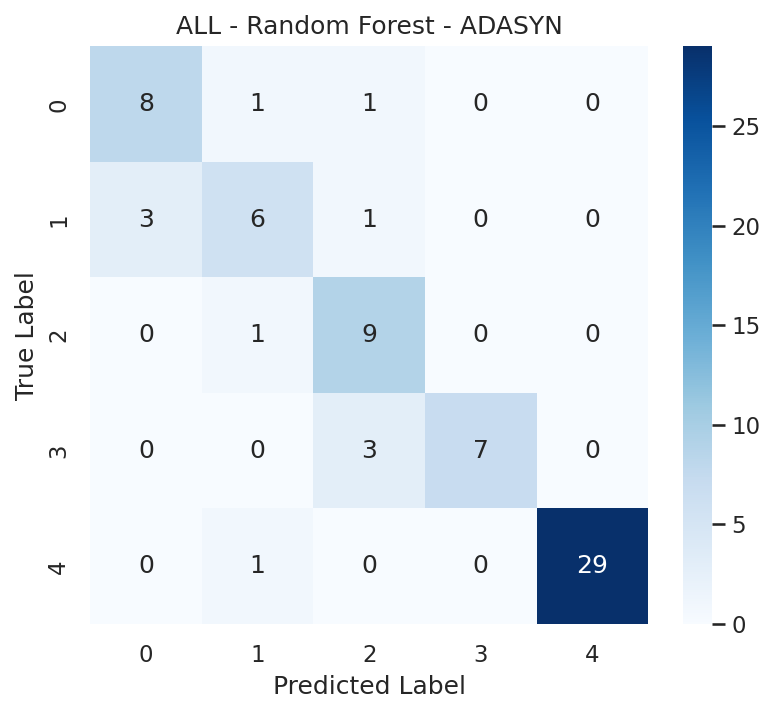

Supplement: Supplementary file 1 [file bioengineering-13-00787-s001.zip › Supplementary Material - Performance Metrics/cm_ALL_Random Forest_ADASYN.png]

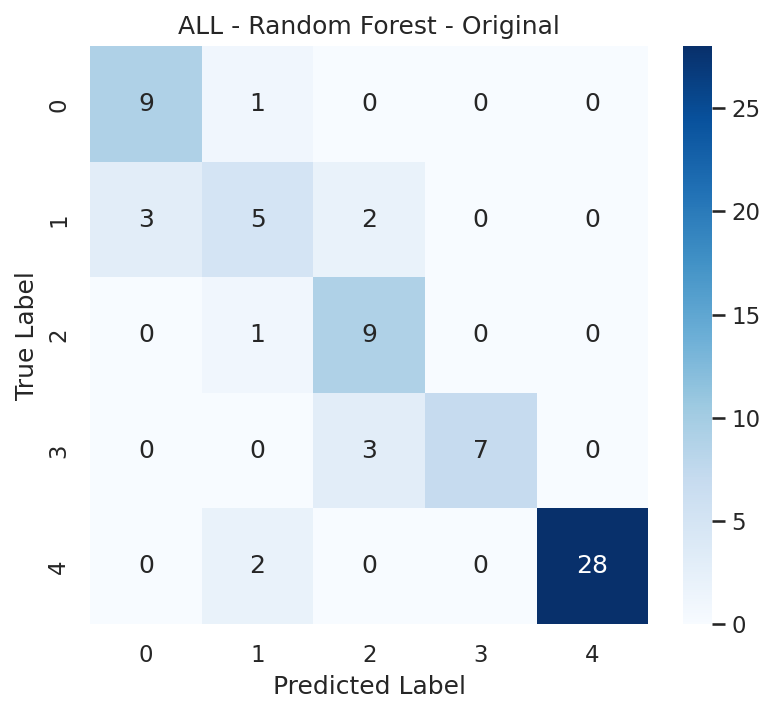

Supplement: Supplementary file 1 [file bioengineering-13-00787-s001.zip › Supplementary Material - Performance Metrics/cm_ALL_Random Forest_Original.png]

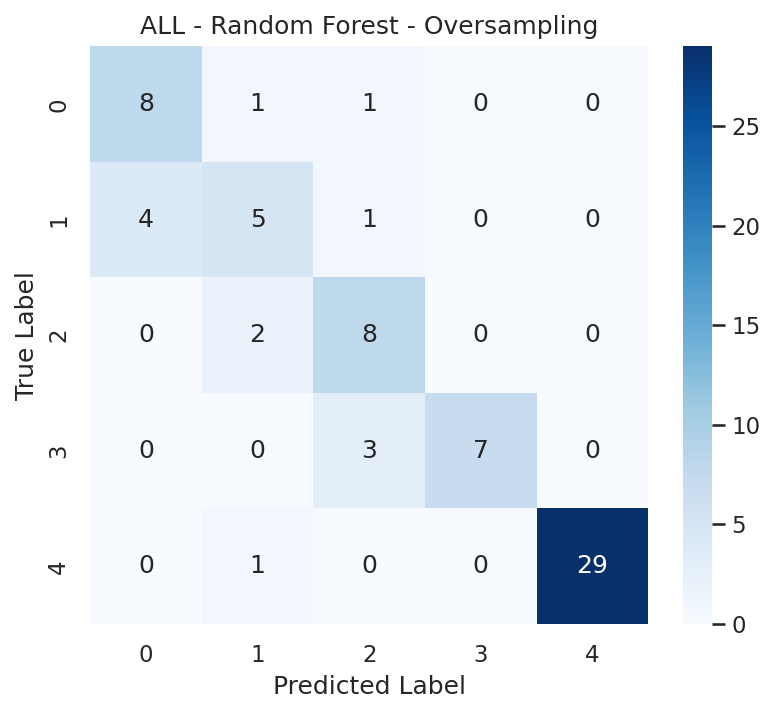

Supplement: Supplementary file 1 [file bioengineering-13-00787-s001.zip › Supplementary Material - Performance Metrics/cm_ALL_Random Forest_Oversampling.png]

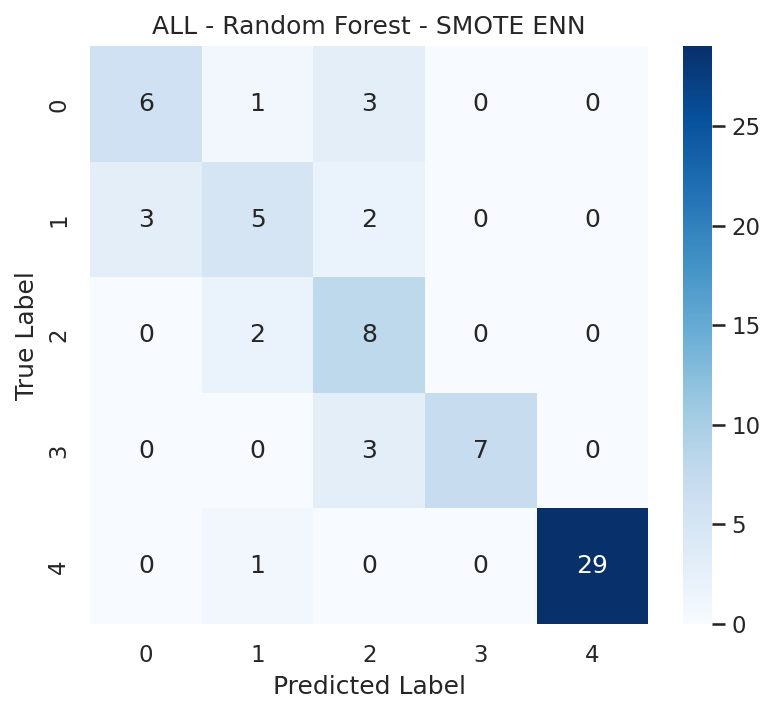

Supplement: Supplementary file 1 [file bioengineering-13-00787-s001.zip › Supplementary Material - Performance Metrics/cm_ALL_Random Forest_SMOTE ENN.png]

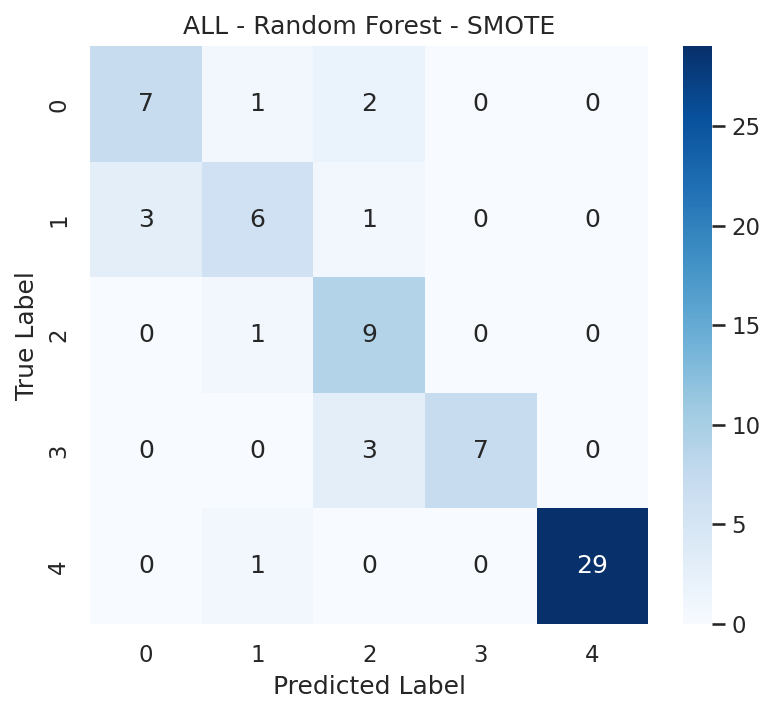

Supplement: Supplementary file 1 [file bioengineering-13-00787-s001.zip › Supplementary Material - Performance Metrics/cm_ALL_Random Forest_SMOTE.png]

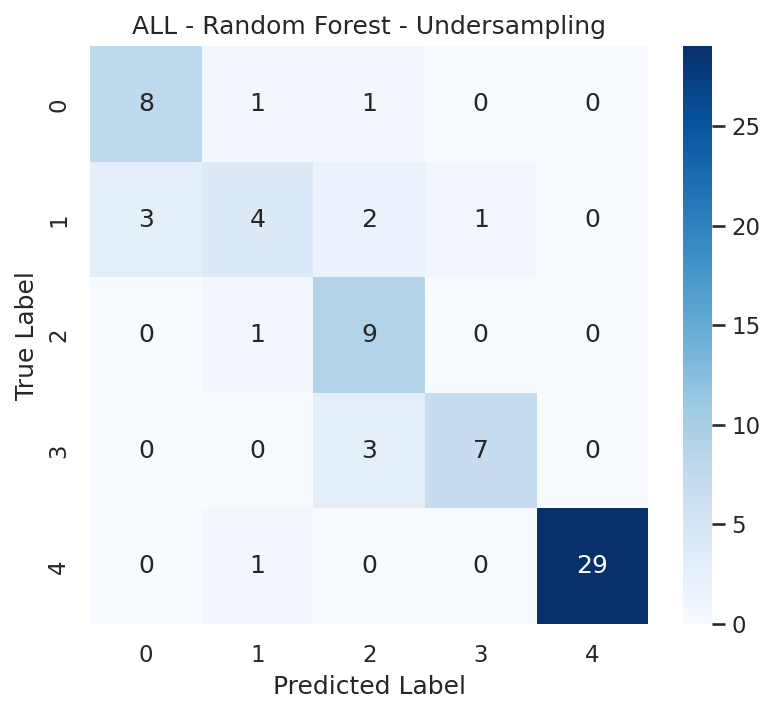

Supplement: Supplementary file 1 [file bioengineering-13-00787-s001.zip › Supplementary Material - Performance Metrics/cm_ALL_Random Forest_Undersampling.png]

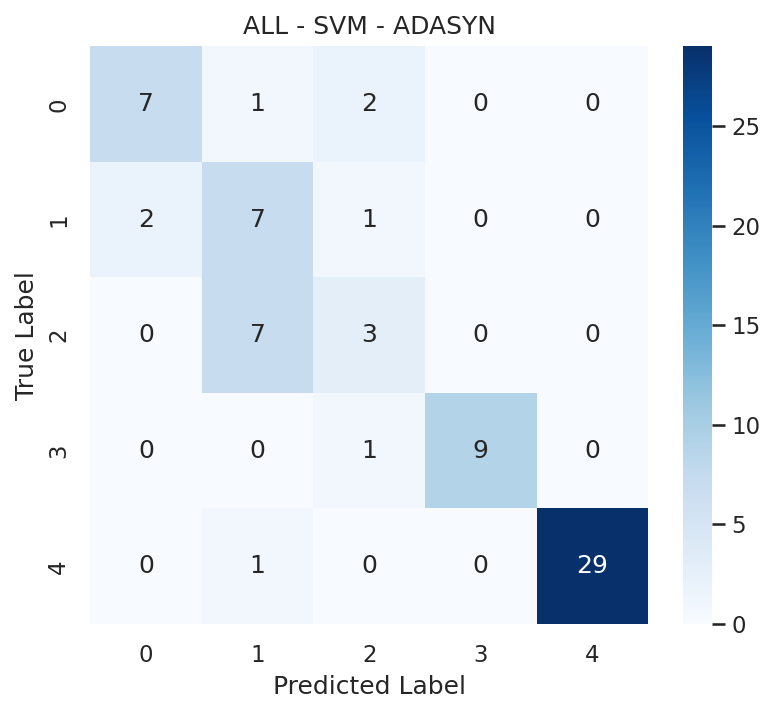

Supplement: Supplementary file 1 [file bioengineering-13-00787-s001.zip › Supplementary Material - Performance Metrics/cm_ALL_SVM_ADASYN.png]

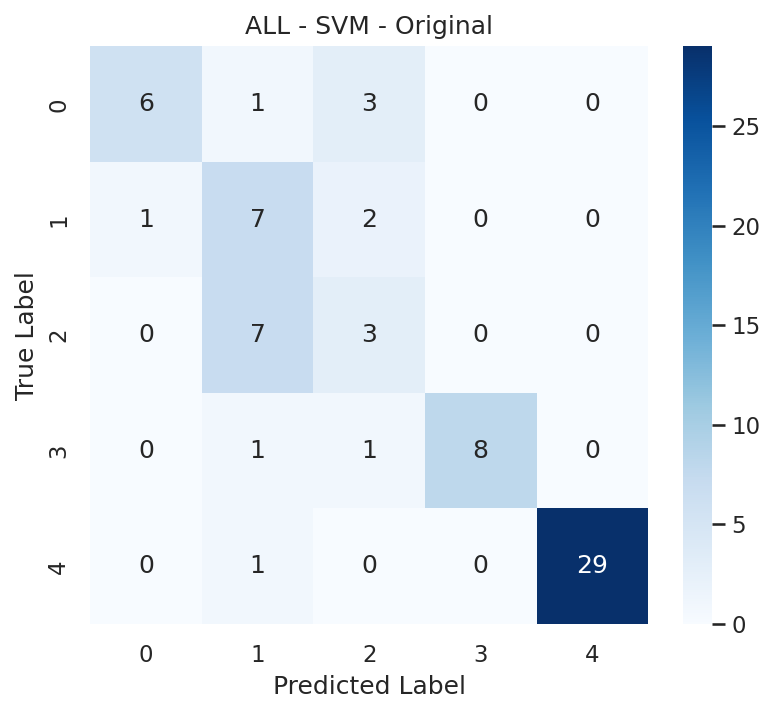

Supplement: Supplementary file 1 [file bioengineering-13-00787-s001.zip › Supplementary Material - Performance Metrics/cm_ALL_SVM_Original.png]

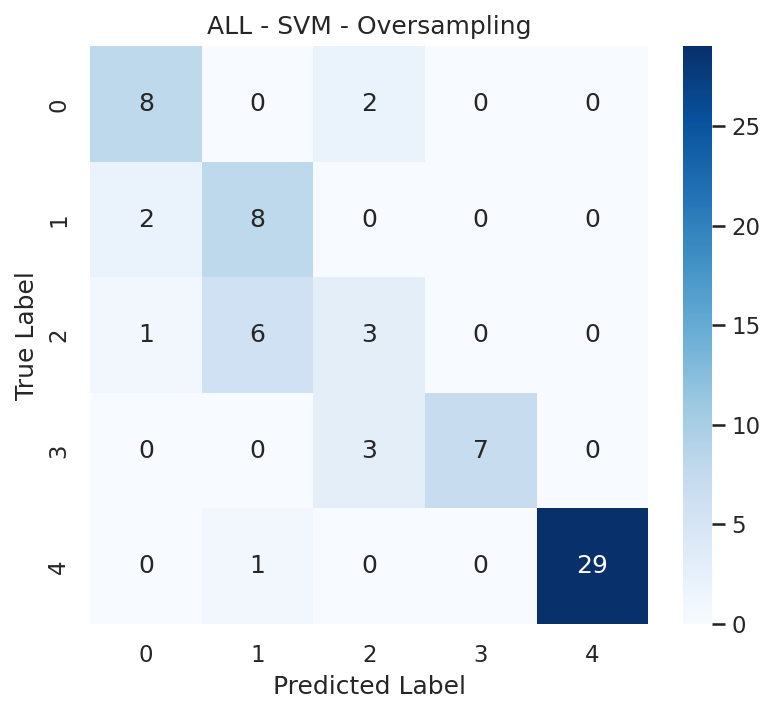

Supplement: Supplementary file 1 [file bioengineering-13-00787-s001.zip › Supplementary Material - Performance Metrics/cm_ALL_SVM_Oversampling.png]

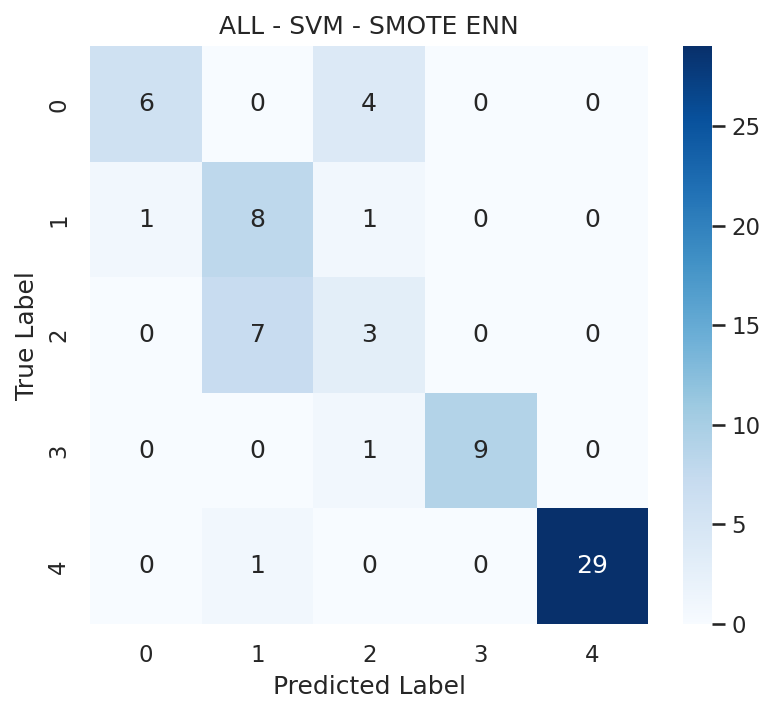

Supplement: Supplementary file 1 [file bioengineering-13-00787-s001.zip › Supplementary Material - Performance Metrics/cm_ALL_SVM_SMOTE ENN.png]

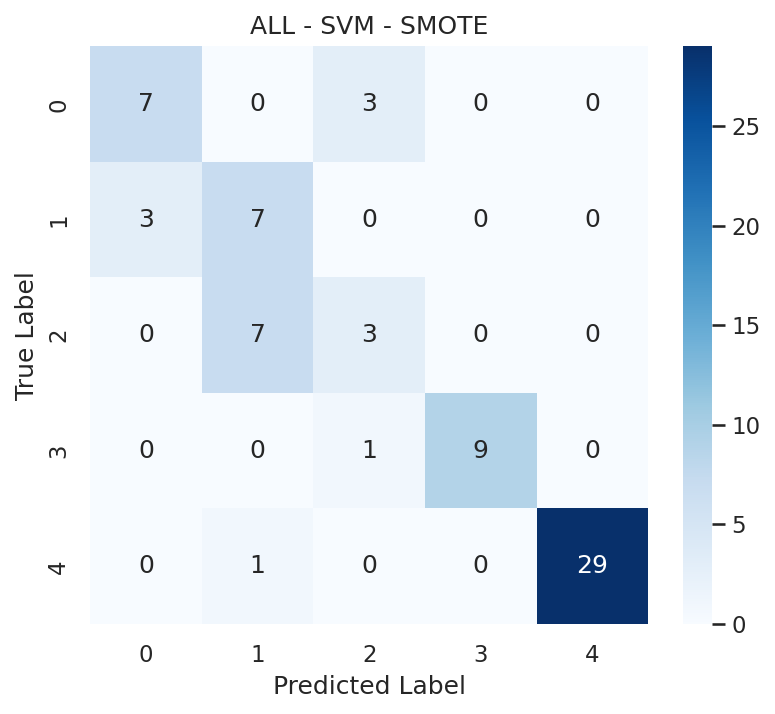

Supplement: Supplementary file 1 [file bioengineering-13-00787-s001.zip › Supplementary Material - Performance Metrics/cm_ALL_SVM_SMOTE.png]

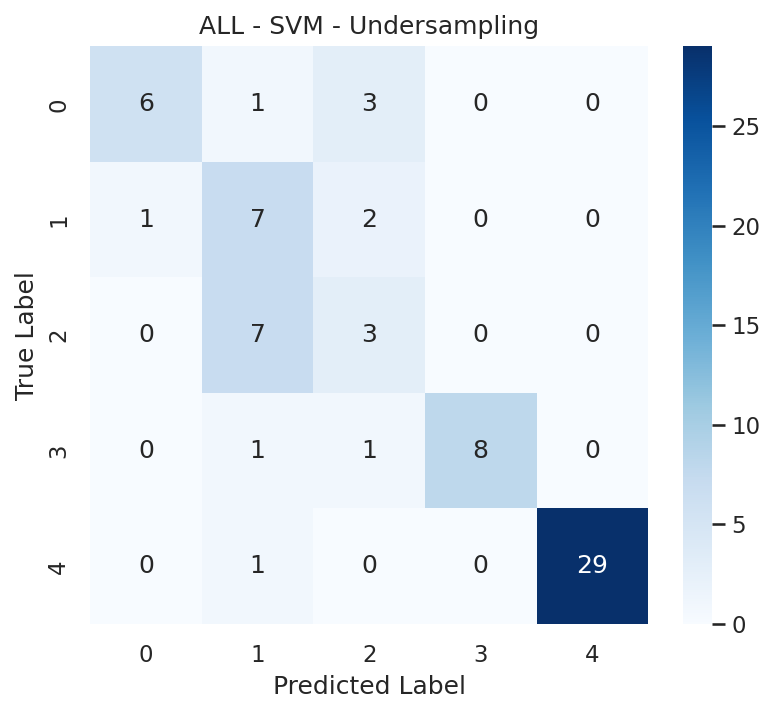

Supplement: Supplementary file 1 [file bioengineering-13-00787-s001.zip › Supplementary Material - Performance Metrics/cm_ALL_SVM_Undersampling.png]

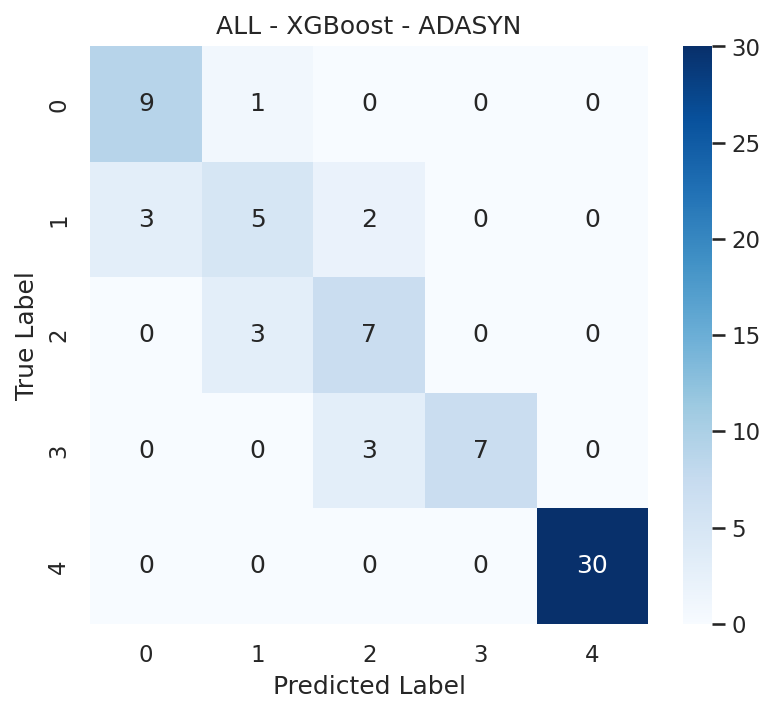

Supplement: Supplementary file 1 [file bioengineering-13-00787-s001.zip › Supplementary Material - Performance Metrics/cm_ALL_XGBoost_ADASYN.png]

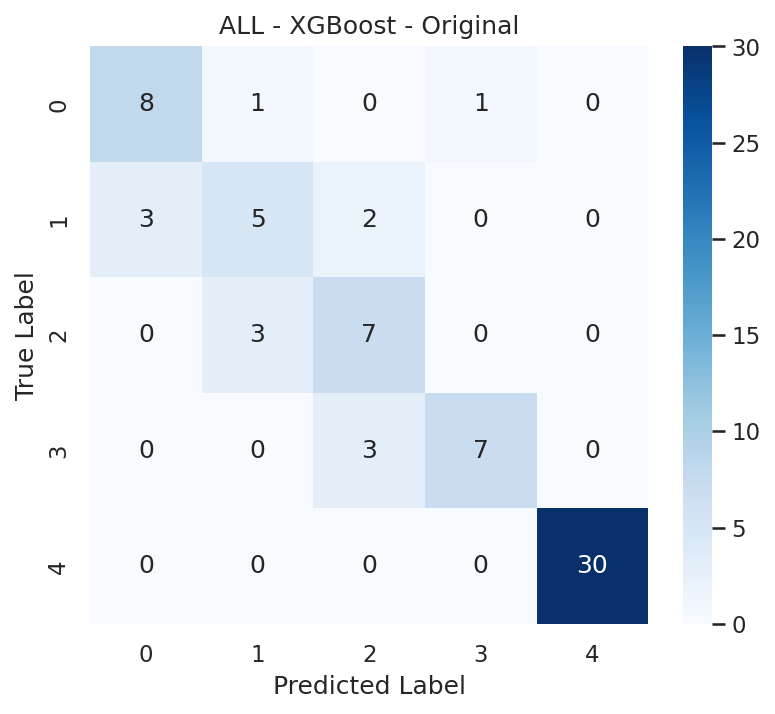

Supplement: Supplementary file 1 [file bioengineering-13-00787-s001.zip › Supplementary Material - Performance Metrics/cm_ALL_XGBoost_Original.png]

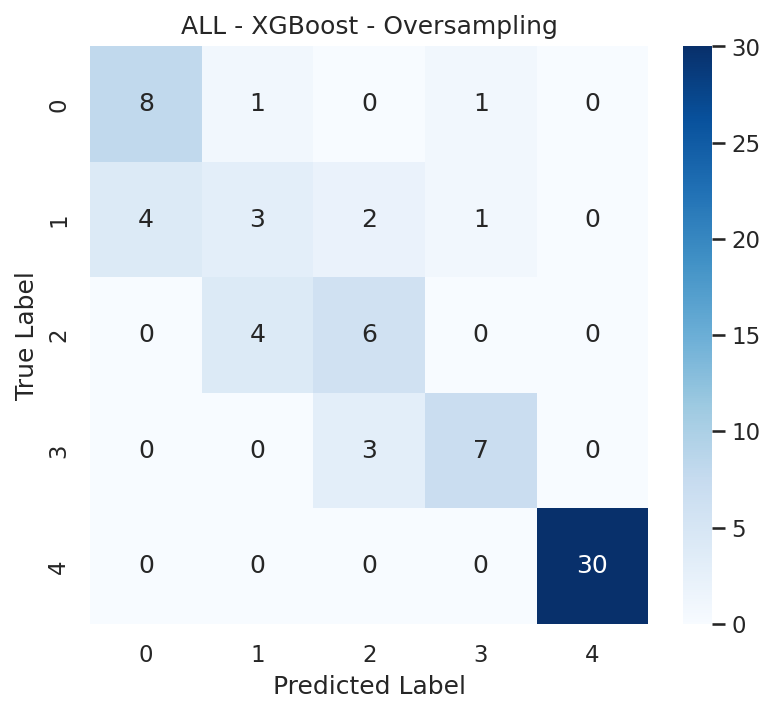

Supplement: Supplementary file 1 [file bioengineering-13-00787-s001.zip › Supplementary Material - Performance Metrics/cm_ALL_XGBoost_Oversampling.png]

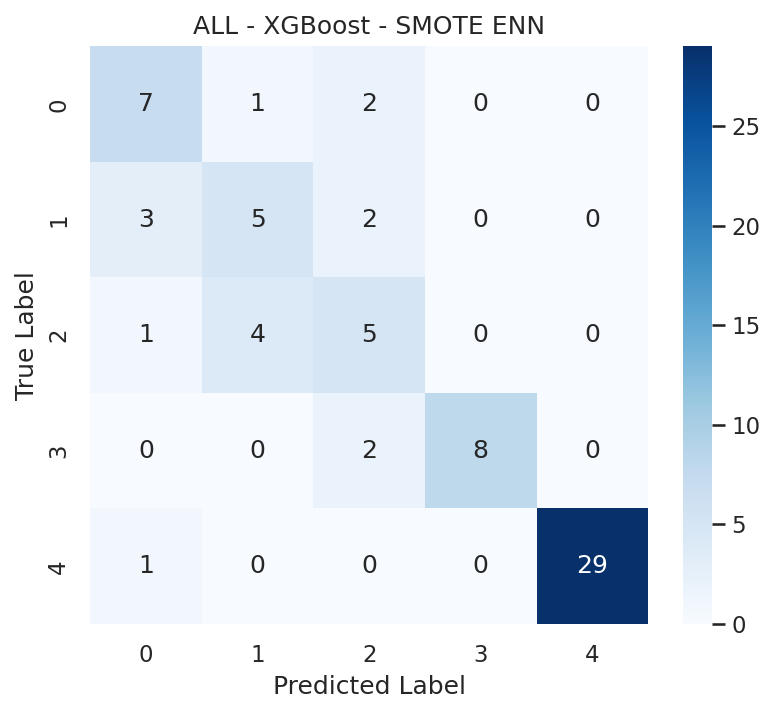

Supplement: Supplementary file 1 [file bioengineering-13-00787-s001.zip › Supplementary Material - Performance Metrics/cm_ALL_XGBoost_SMOTE ENN.png]

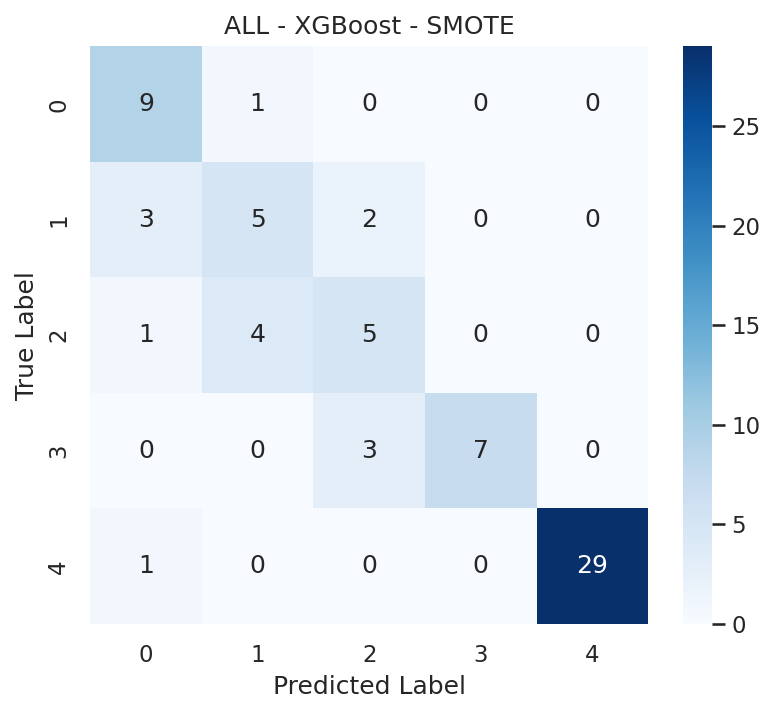

Supplement: Supplementary file 1 [file bioengineering-13-00787-s001.zip › Supplementary Material - Performance Metrics/cm_ALL_XGBoost_SMOTE.png]

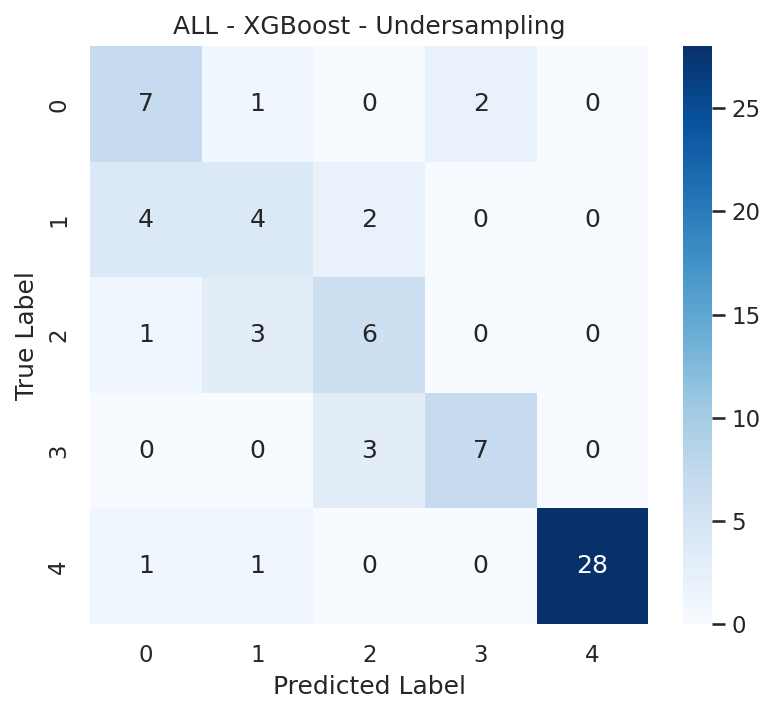

Supplement: Supplementary file 1 [file bioengineering-13-00787-s001.zip › Supplementary Material - Performance Metrics/cm_ALL_XGBoost_Undersampling.png]

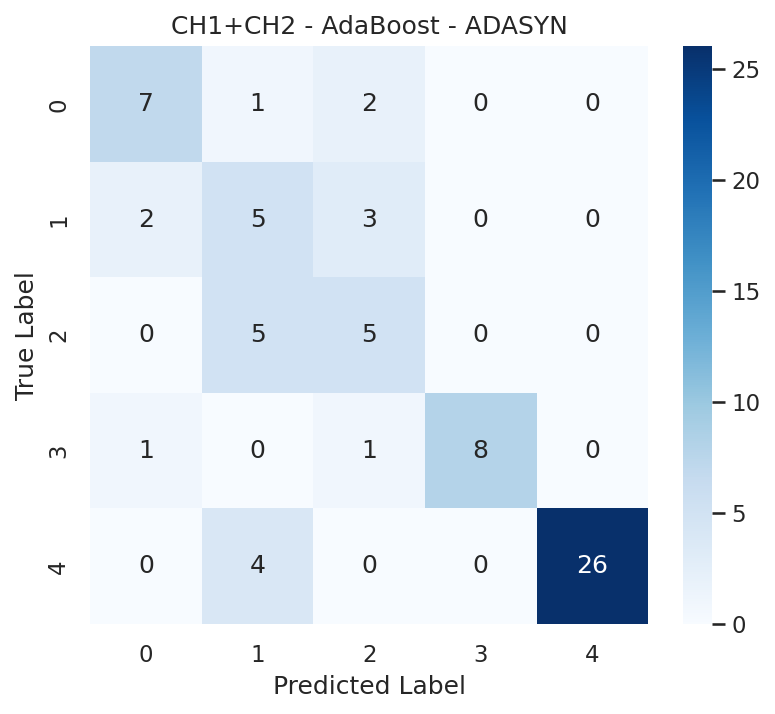

Supplement: Supplementary file 1 [file bioengineering-13-00787-s001.zip › Supplementary Material - Performance Metrics/cm_CH1+CH2_AdaBoost_ADASYN.png]

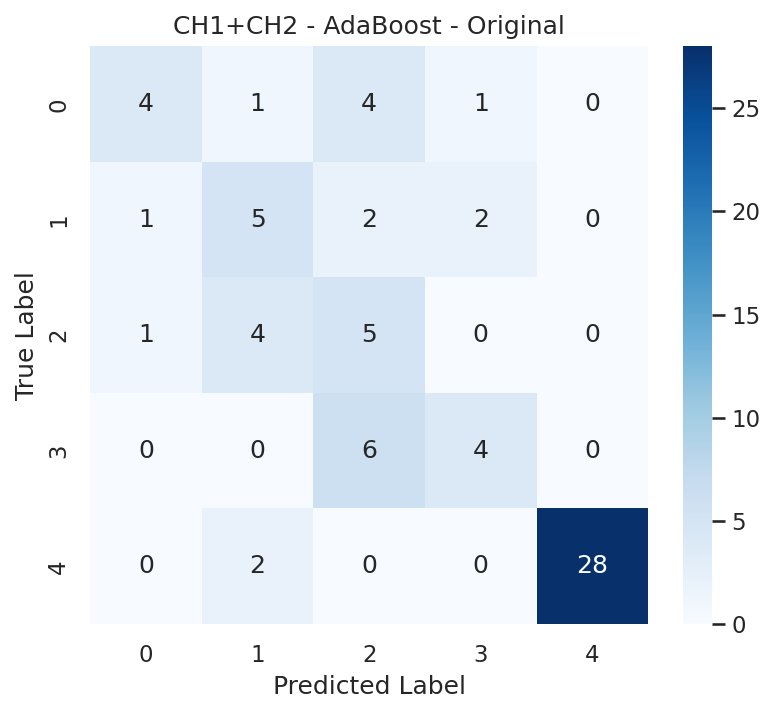

Supplement: Supplementary file 1 [file bioengineering-13-00787-s001.zip › Supplementary Material - Performance Metrics/cm_CH1+CH2_AdaBoost_Original.png]

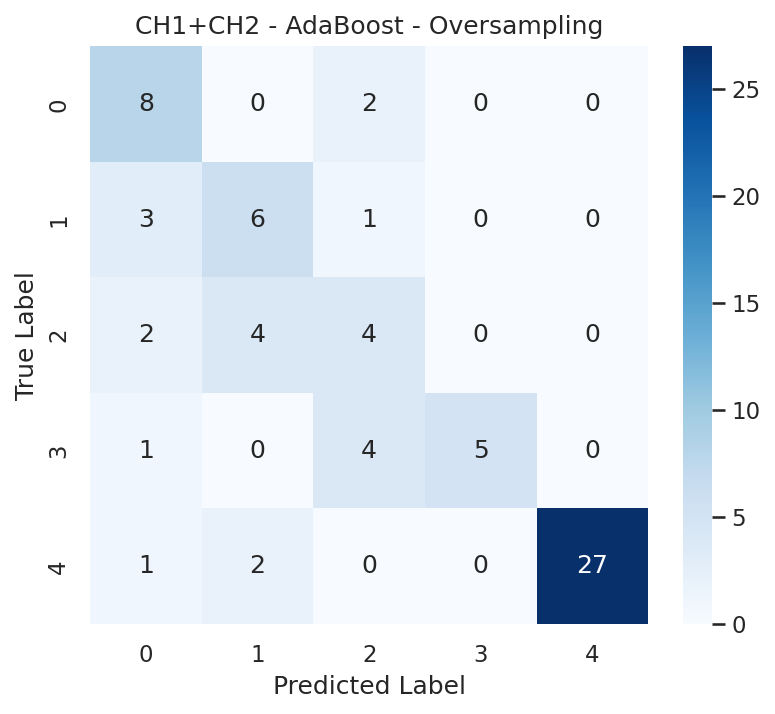

Supplement: Supplementary file 1 [file bioengineering-13-00787-s001.zip › Supplementary Material - Performance Metrics/cm_CH1+CH2_AdaBoost_Oversampling.png]

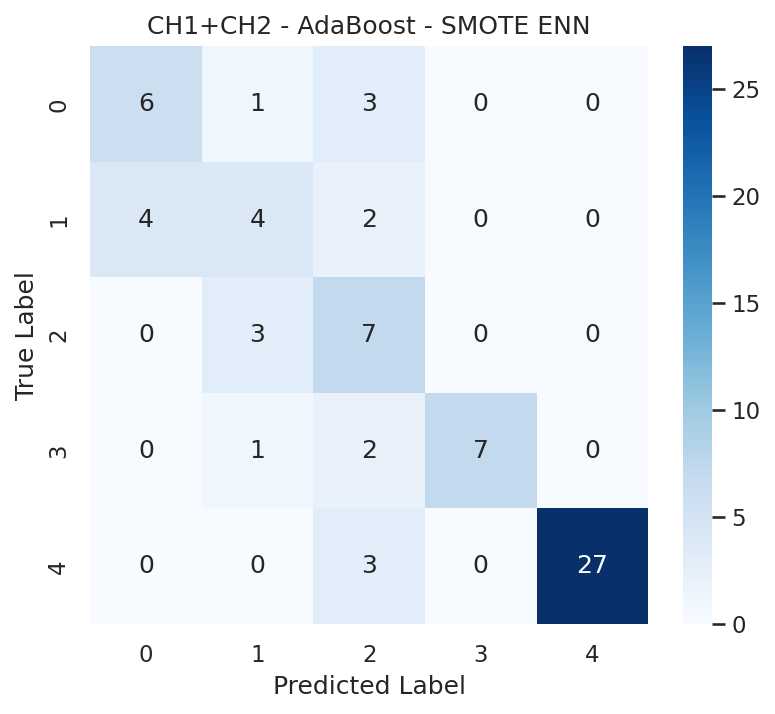

Supplement: Supplementary file 1 [file bioengineering-13-00787-s001.zip › Supplementary Material - Performance Metrics/cm_CH1+CH2_AdaBoost_SMOTE ENN.png]

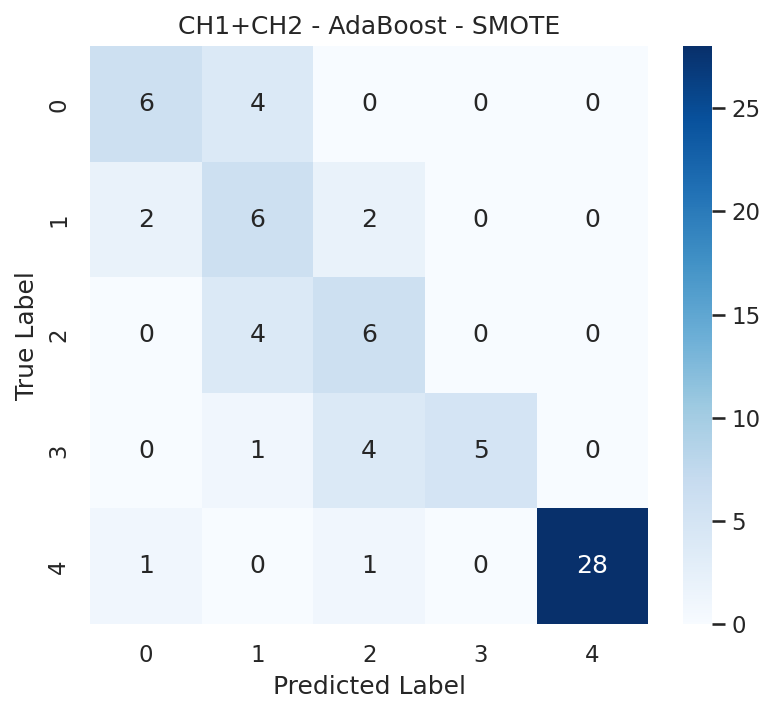

Supplement: Supplementary file 1 [file bioengineering-13-00787-s001.zip › Supplementary Material - Performance Metrics/cm_CH1+CH2_AdaBoost_SMOTE.png]

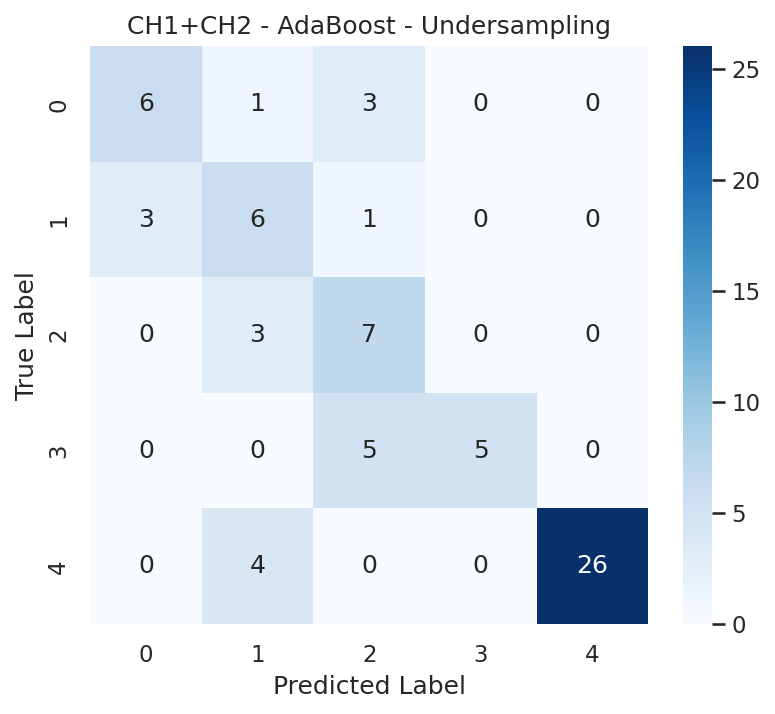

Supplement: Supplementary file 1 [file bioengineering-13-00787-s001.zip › Supplementary Material - Performance Metrics/cm_CH1+CH2_AdaBoost_Undersampling.png]

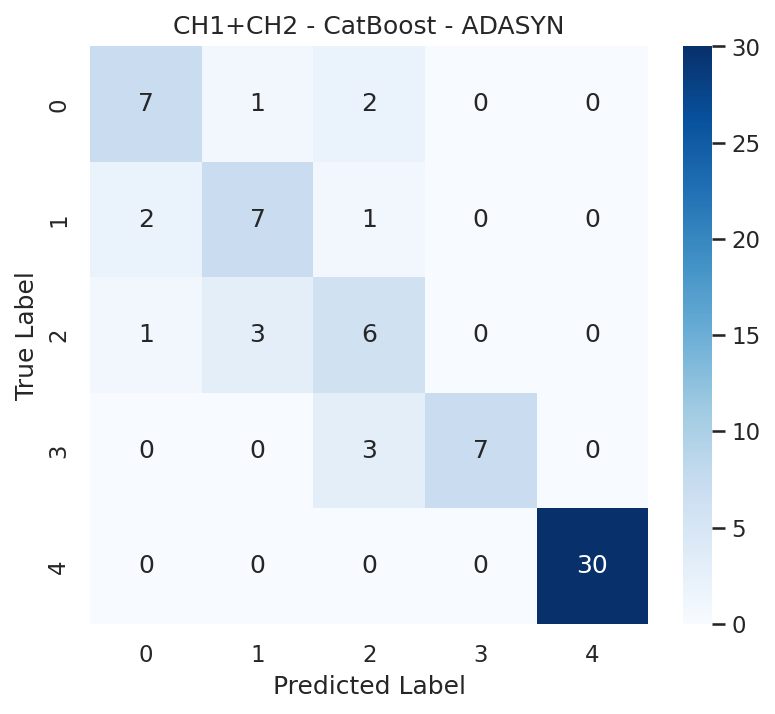

Supplement: Supplementary file 1 [file bioengineering-13-00787-s001.zip › Supplementary Material - Performance Metrics/cm_CH1+CH2_CatBoost_ADASYN.png]

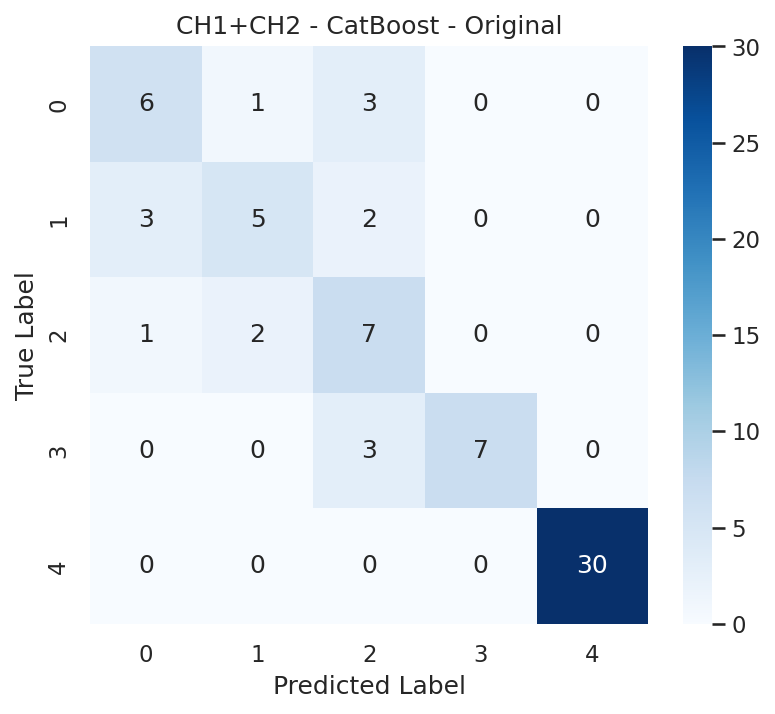

Supplement: Supplementary file 1 [file bioengineering-13-00787-s001.zip › Supplementary Material - Performance Metrics/cm_CH1+CH2_CatBoost_Original.png]

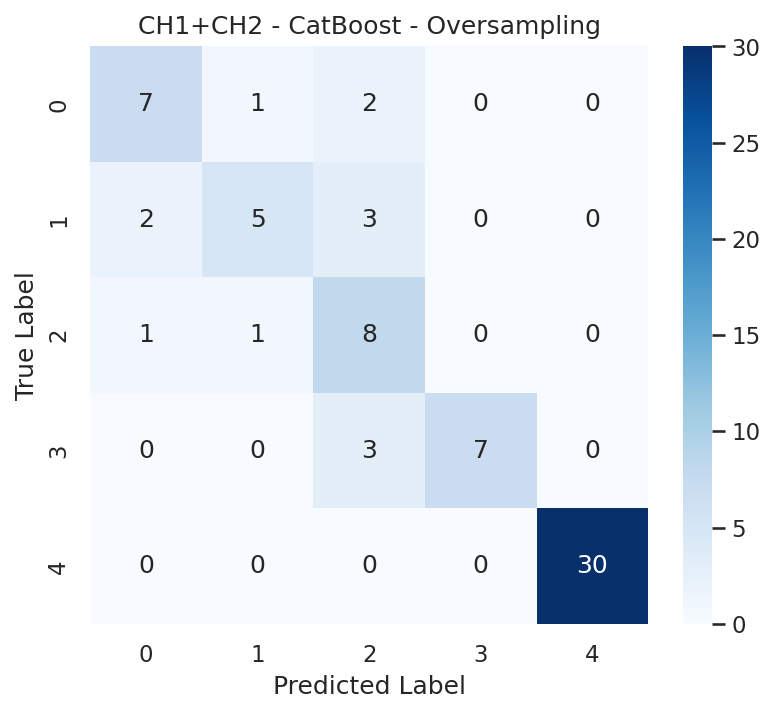

Supplement: Supplementary file 1 [file bioengineering-13-00787-s001.zip › Supplementary Material - Performance Metrics/cm_CH1+CH2_CatBoost_Oversampling.png]

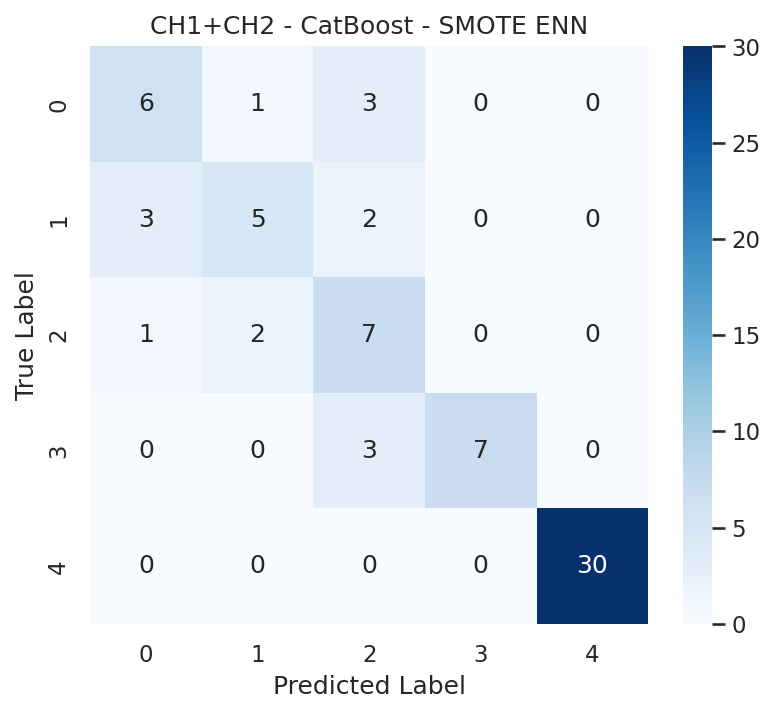

Supplement: Supplementary file 1 [file bioengineering-13-00787-s001.zip › Supplementary Material - Performance Metrics/cm_CH1+CH2_CatBoost_SMOTE ENN.png]

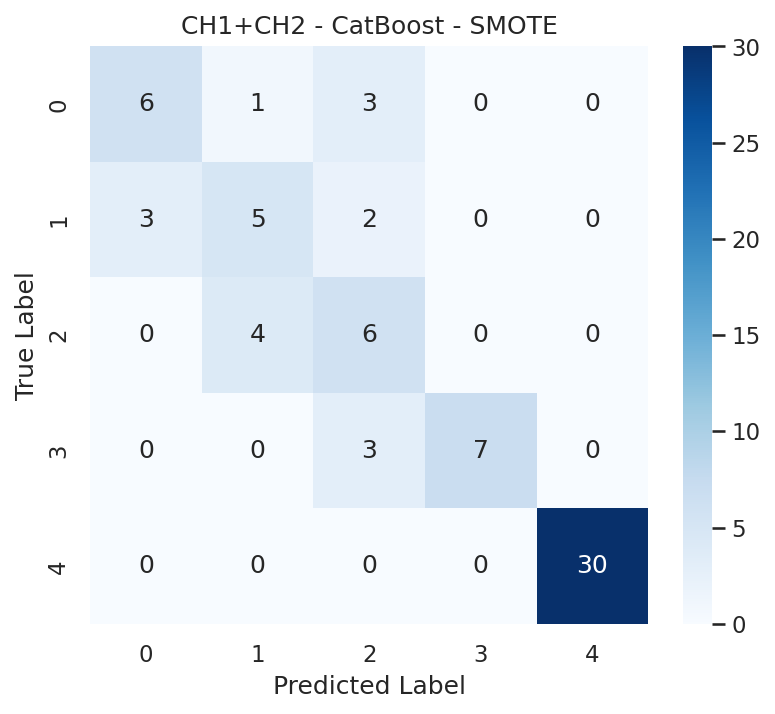

Supplement: Supplementary file 1 [file bioengineering-13-00787-s001.zip › Supplementary Material - Performance Metrics/cm_CH1+CH2_CatBoost_SMOTE.png]

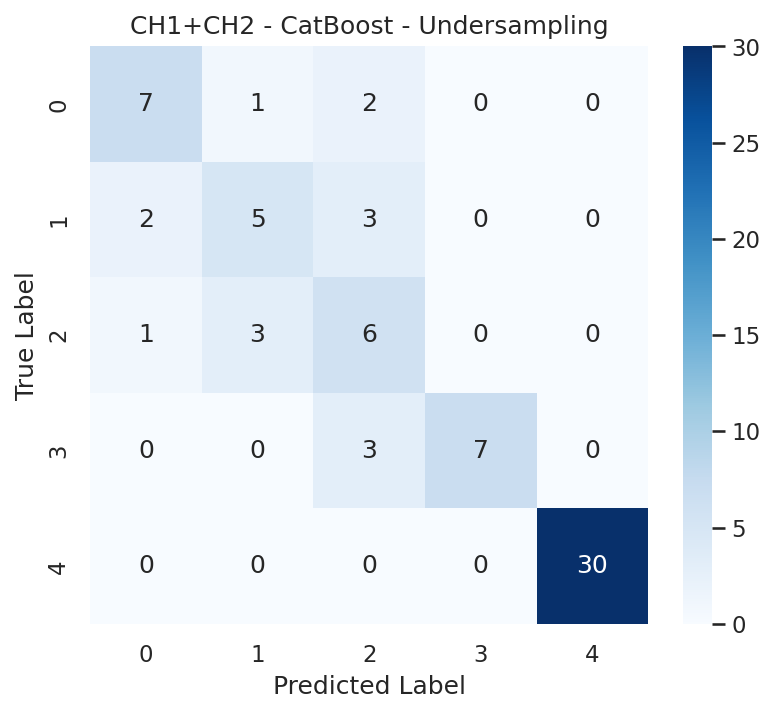

Supplement: Supplementary file 1 [file bioengineering-13-00787-s001.zip › Supplementary Material - Performance Metrics/cm_CH1+CH2_CatBoost_Undersampling.png]

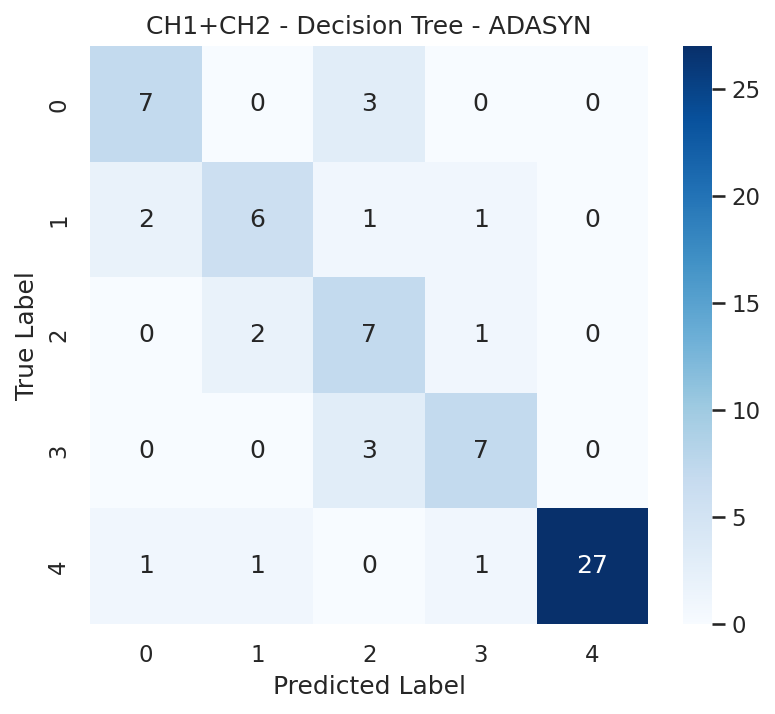

Supplement: Supplementary file 1 [file bioengineering-13-00787-s001.zip › Supplementary Material - Performance Metrics/cm_CH1+CH2_Decision Tree_ADASYN.png]

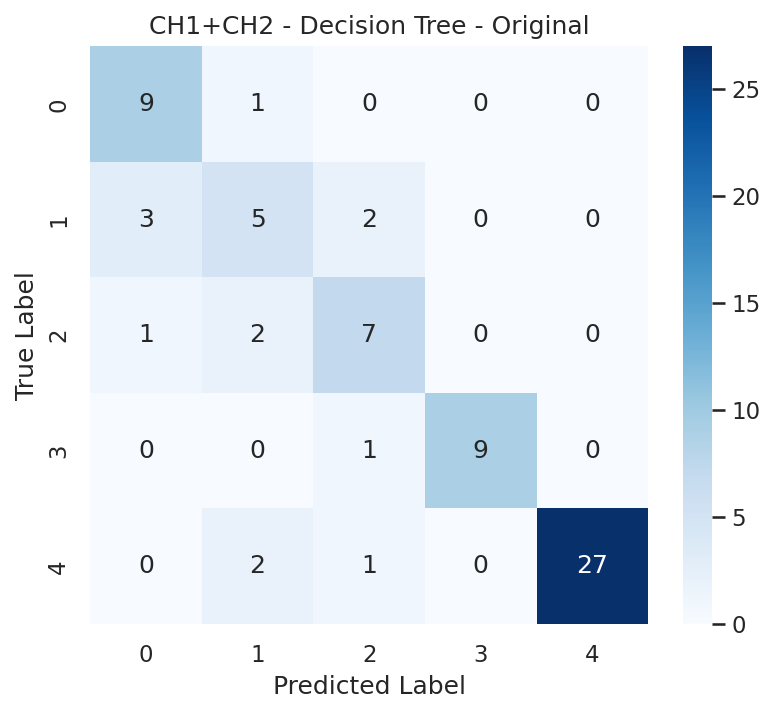

Supplement: Supplementary file 1 [file bioengineering-13-00787-s001.zip › Supplementary Material - Performance Metrics/cm_CH1+CH2_Decision Tree_Original.png]

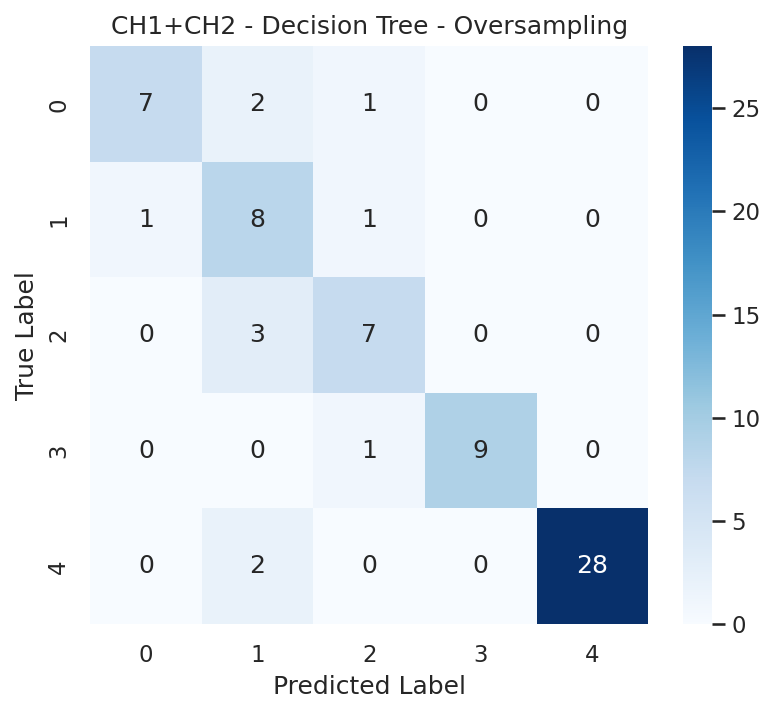

Supplement: Supplementary file 1 [file bioengineering-13-00787-s001.zip › Supplementary Material - Performance Metrics/cm_CH1+CH2_Decision Tree_Oversampling.png]

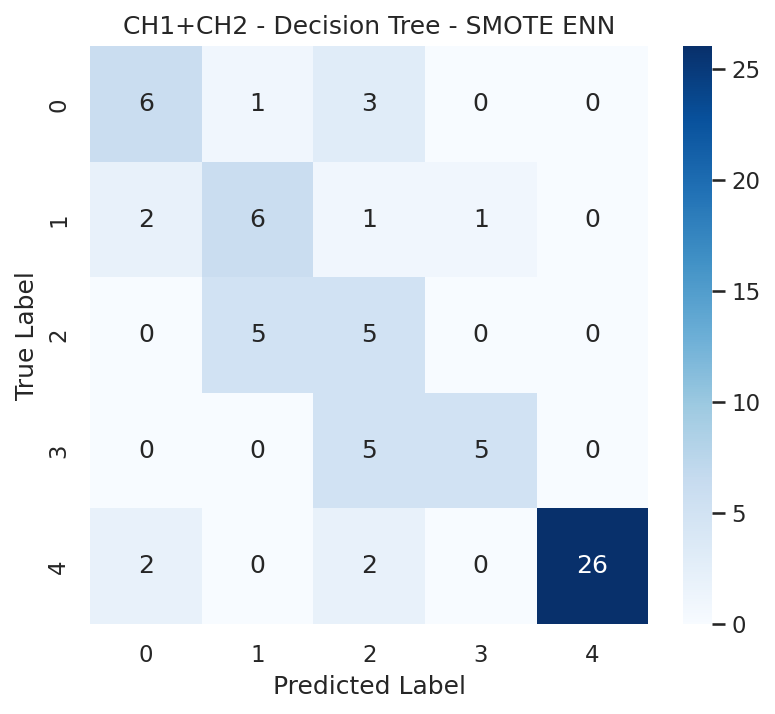

Supplement: Supplementary file 1 [file bioengineering-13-00787-s001.zip › Supplementary Material - Performance Metrics/cm_CH1+CH2_Decision Tree_SMOTE ENN.png]

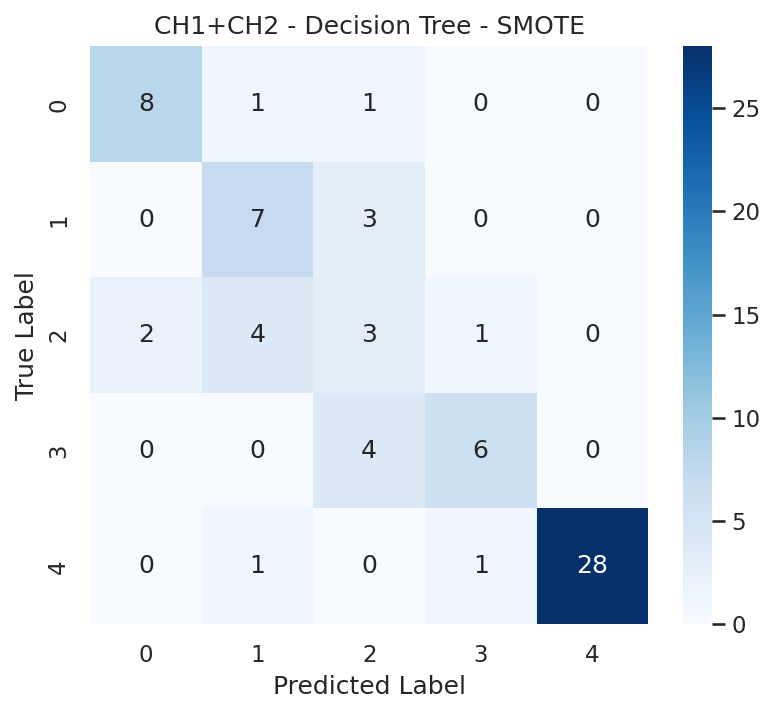

Supplement: Supplementary file 1 [file bioengineering-13-00787-s001.zip › Supplementary Material - Performance Metrics/cm_CH1+CH2_Decision Tree_SMOTE.png]

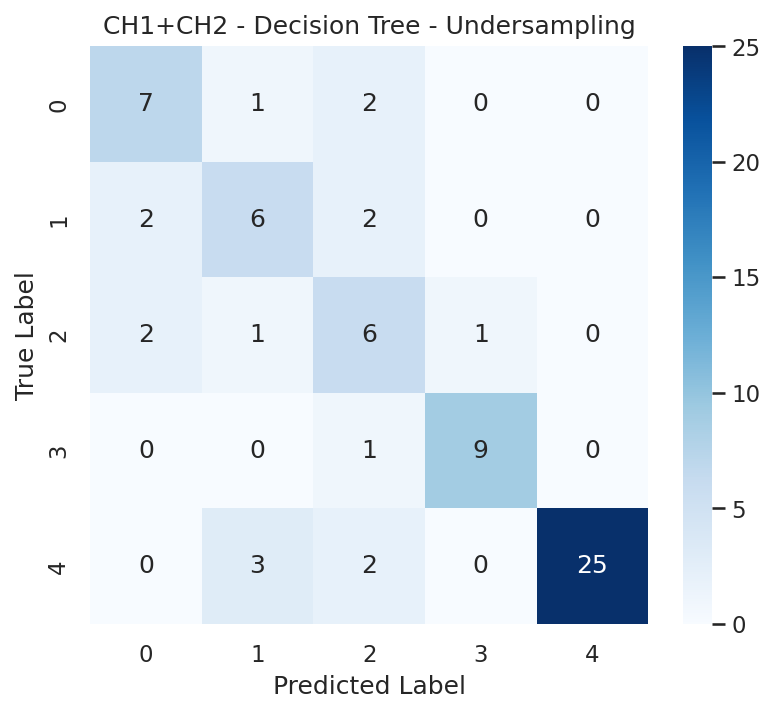

Supplement: Supplementary file 1 [file bioengineering-13-00787-s001.zip › Supplementary Material - Performance Metrics/cm_CH1+CH2_Decision Tree_Undersampling.png]

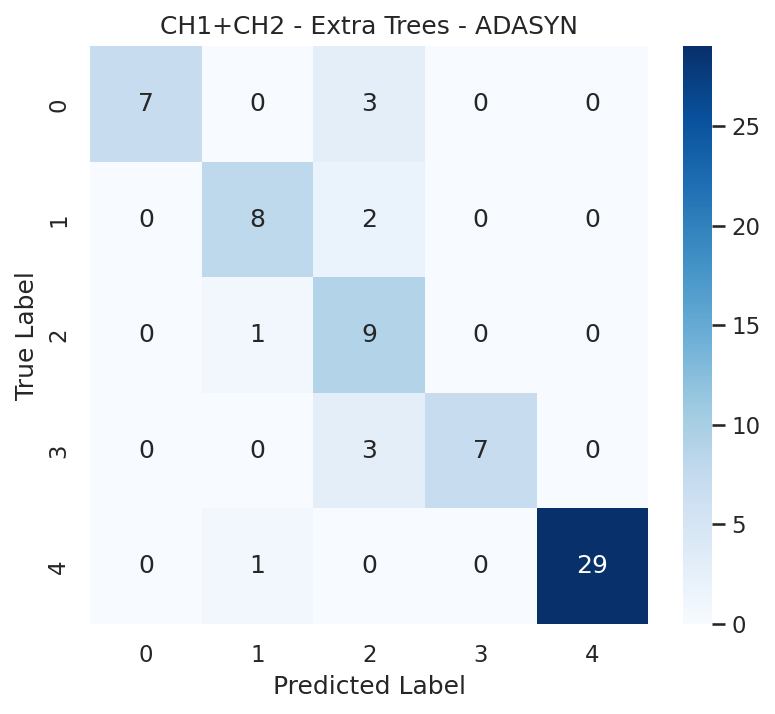

Supplement: Supplementary file 1 [file bioengineering-13-00787-s001.zip › Supplementary Material - Performance Metrics/cm_CH1+CH2_Extra Trees_ADASYN.png]

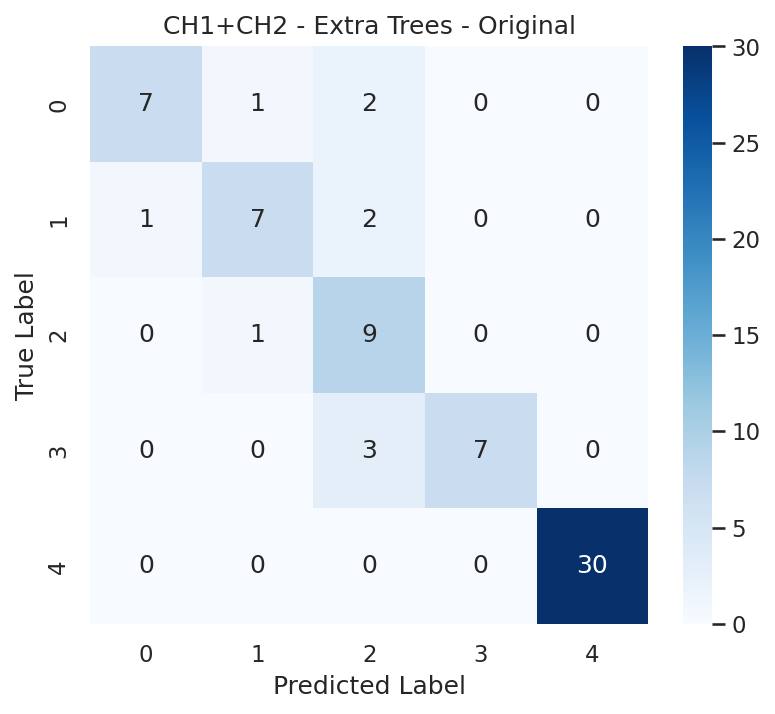

Supplement: Supplementary file 1 [file bioengineering-13-00787-s001.zip › Supplementary Material - Performance Metrics/cm_CH1+CH2_Extra Trees_Original.png]

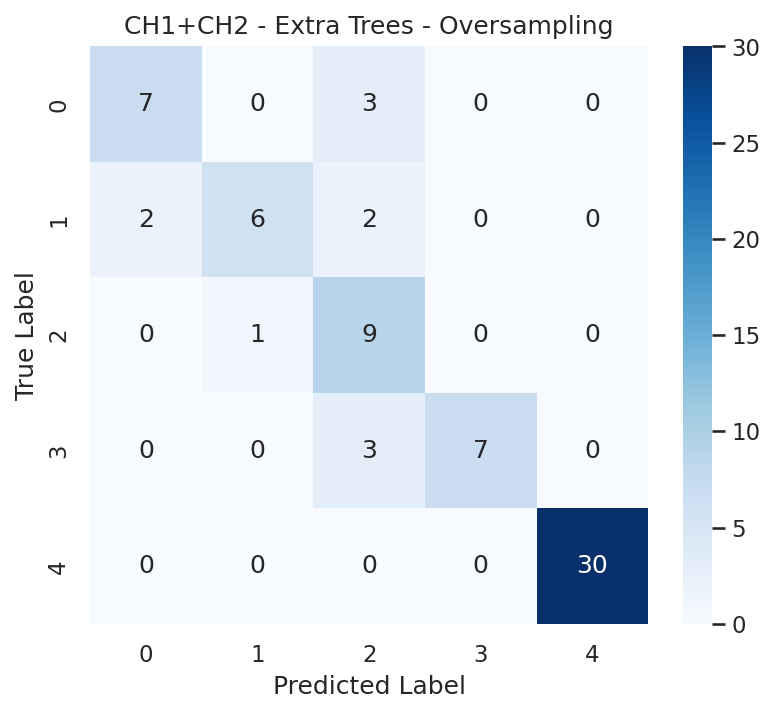

Supplement: Supplementary file 1 [file bioengineering-13-00787-s001.zip › Supplementary Material - Performance Metrics/cm_CH1+CH2_Extra Trees_Oversampling.png]

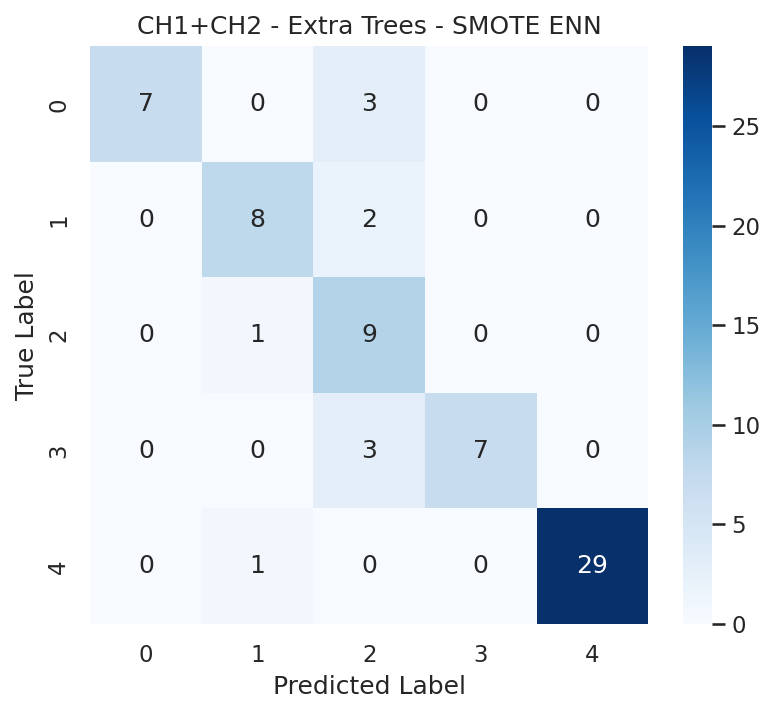

Supplement: Supplementary file 1 [file bioengineering-13-00787-s001.zip › Supplementary Material - Performance Metrics/cm_CH1+CH2_Extra Trees_SMOTE ENN.png]

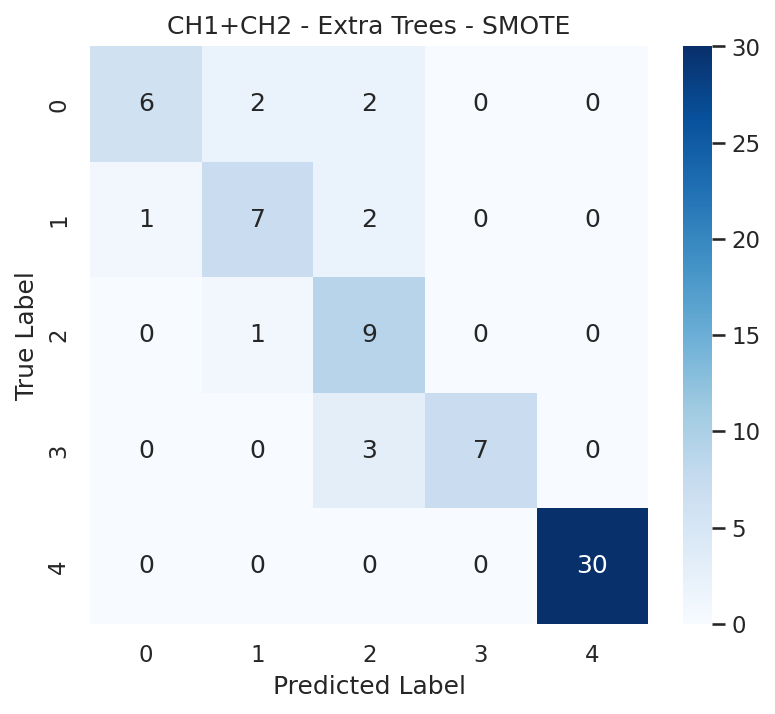

Supplement: Supplementary file 1 [file bioengineering-13-00787-s001.zip › Supplementary Material - Performance Metrics/cm_CH1+CH2_Extra Trees_SMOTE.png]

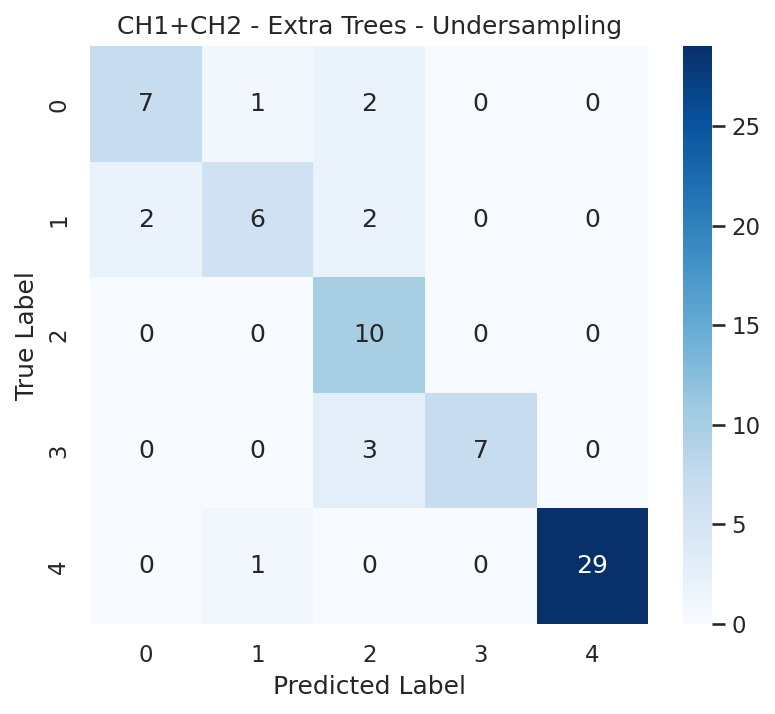

Supplement: Supplementary file 1 [file bioengineering-13-00787-s001.zip › Supplementary Material - Performance Metrics/cm_CH1+CH2_Extra Trees_Undersampling.png]

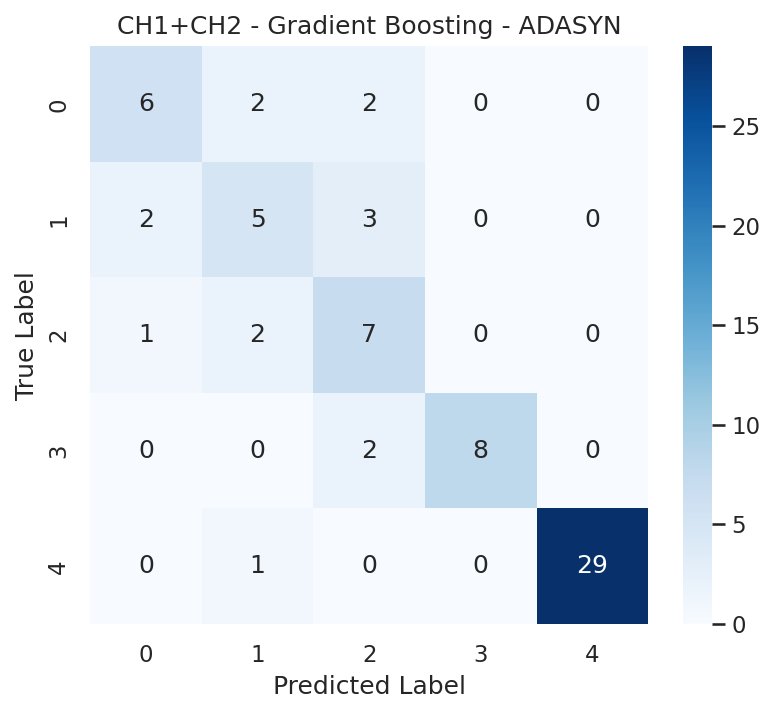

Supplement: Supplementary file 1 [file bioengineering-13-00787-s001.zip › Supplementary Material - Performance Metrics/cm_CH1+CH2_Gradient Boosting_ADASYN.png]

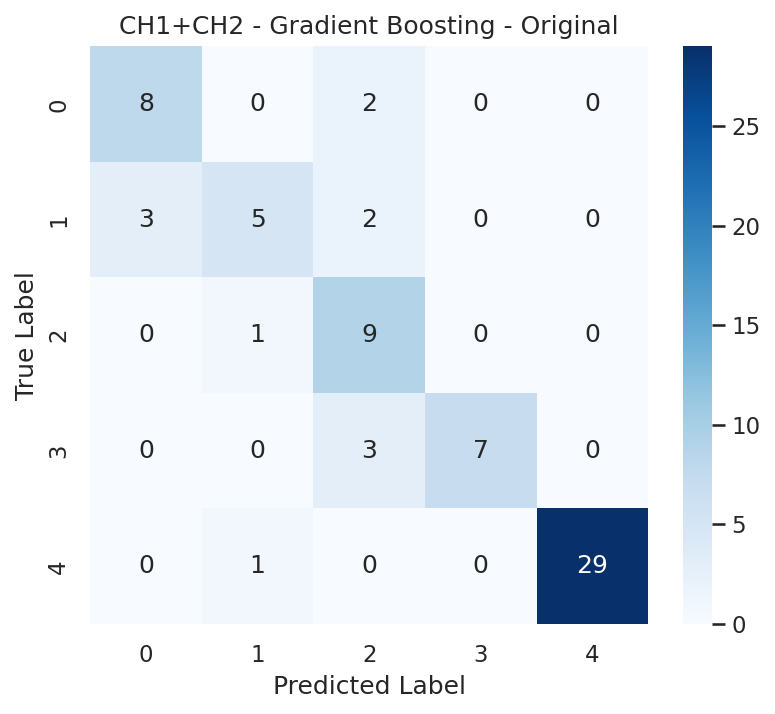

Supplement: Supplementary file 1 [file bioengineering-13-00787-s001.zip › Supplementary Material - Performance Metrics/cm_CH1+CH2_Gradient Boosting_Original.png]

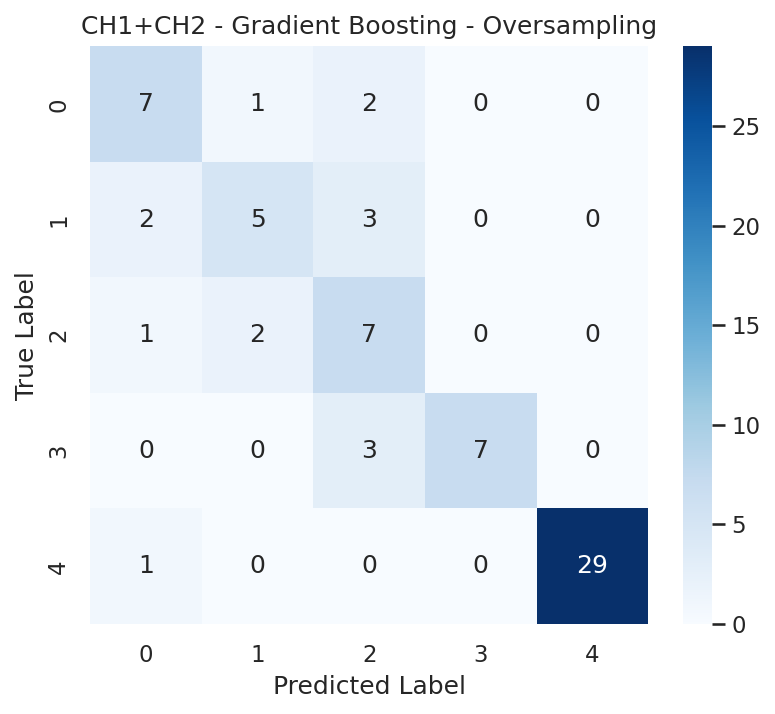

Supplement: Supplementary file 1 [file bioengineering-13-00787-s001.zip › Supplementary Material - Performance Metrics/cm_CH1+CH2_Gradient Boosting_Oversampling.png]

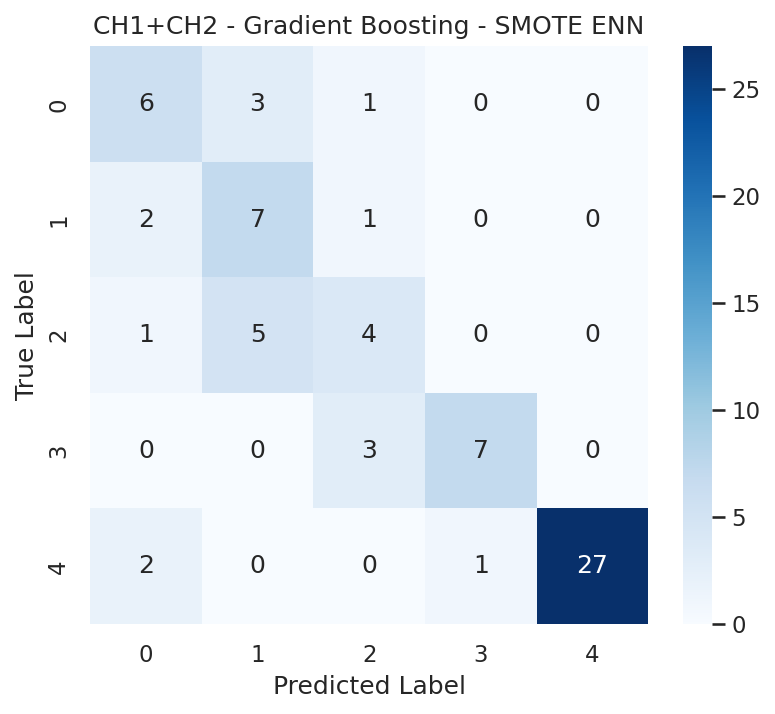

Supplement: Supplementary file 1 [file bioengineering-13-00787-s001.zip › Supplementary Material - Performance Metrics/cm_CH1+CH2_Gradient Boosting_SMOTE ENN.png]

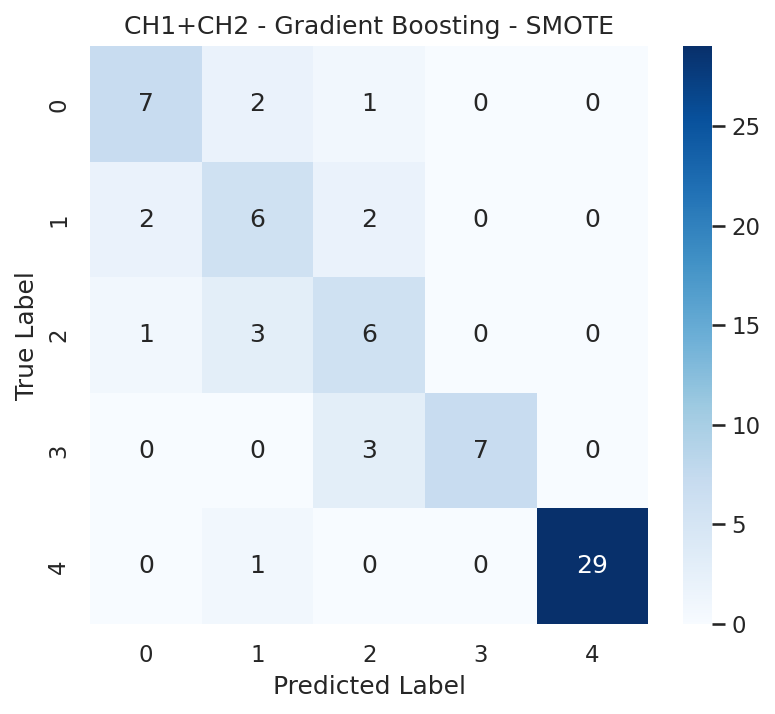

Supplement: Supplementary file 1 [file bioengineering-13-00787-s001.zip › Supplementary Material - Performance Metrics/cm_CH1+CH2_Gradient Boosting_SMOTE.png]

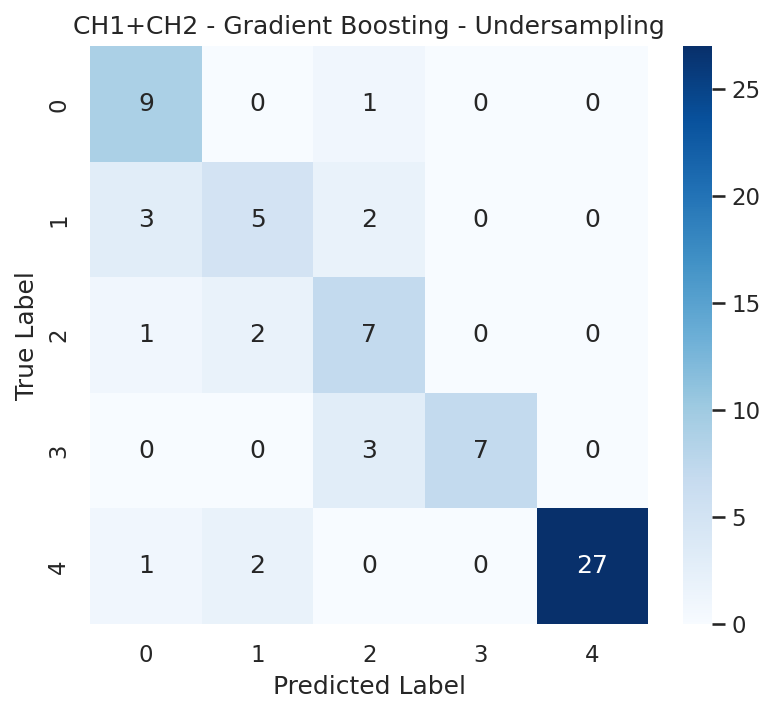

Supplement: Supplementary file 1 [file bioengineering-13-00787-s001.zip › Supplementary Material - Performance Metrics/cm_CH1+CH2_Gradient Boosting_Undersampling.png]
