# Supplementary figures and images for: Machine Learning-Based Self-Induced Scratch Intensity Detection Using Feature Optimization and Multi-Channel Electromyogram Signals for Prevention of Lichenification (part 1 of 2)
Source: Bioengineering (Basel). 2026 Jul 8;13(7):787. doi: 10.3390/bioengineering13070787 (PMC13405920; doi:10.3390/bioengineering13070787)

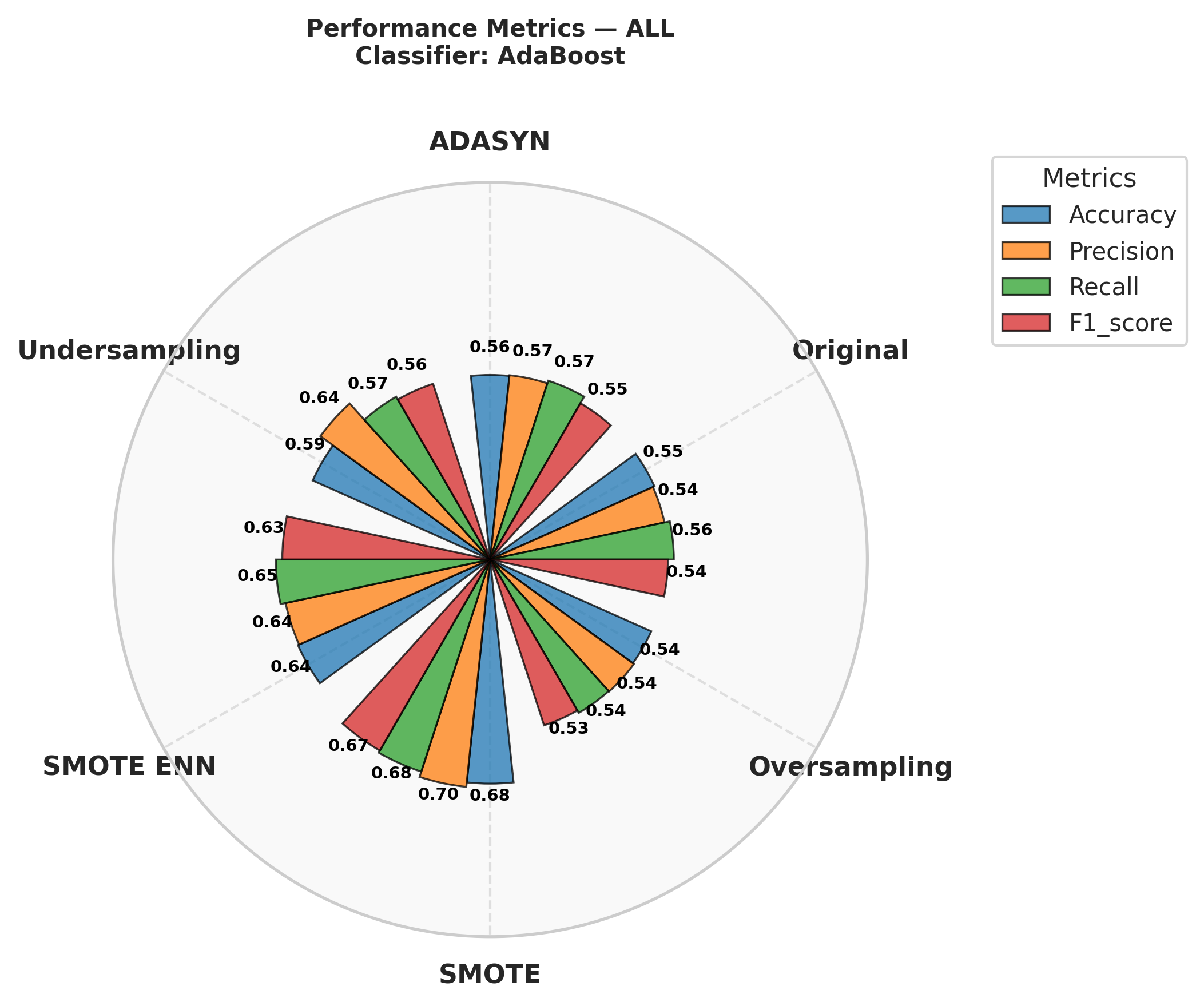

Supplement: Supplementary file 1 [file bioengineering-13-00787-s001.zip › Supplementary Material - Performance Metrics/ALL_AdaBoost_polar.png]

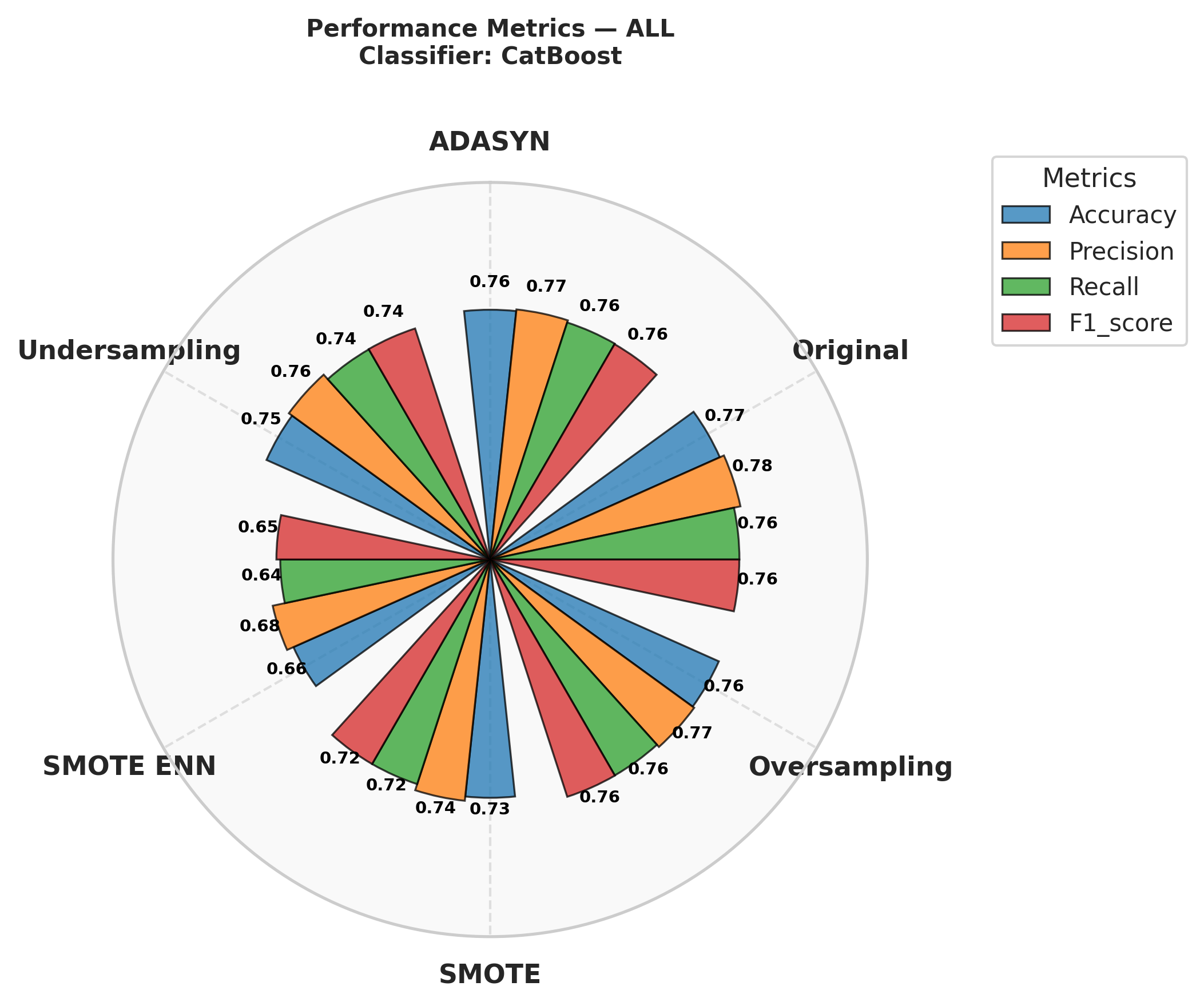

Supplement: Supplementary file 1 [file bioengineering-13-00787-s001.zip › Supplementary Material - Performance Metrics/ALL_CatBoost_polar.png]

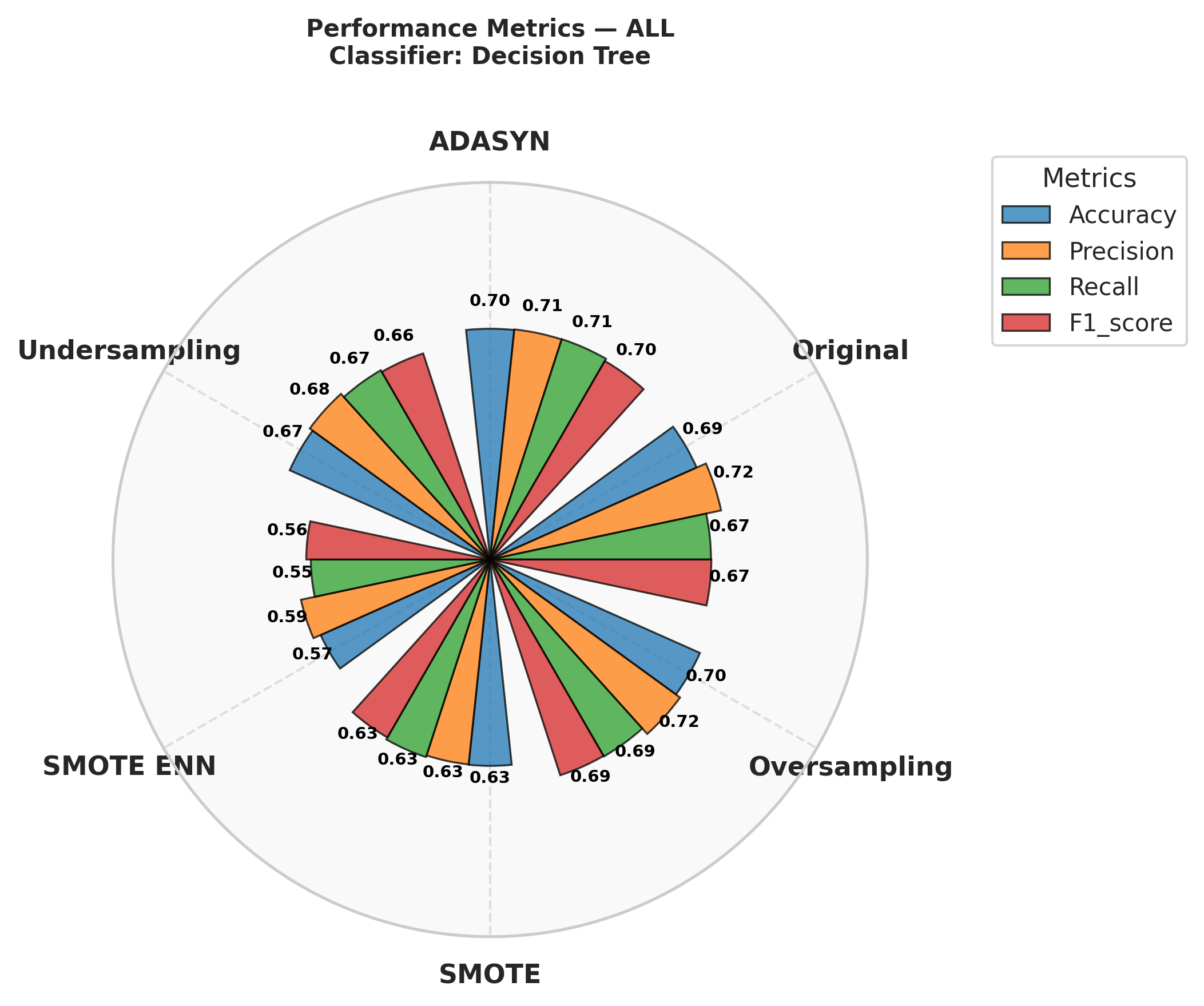

Supplement: Supplementary file 1 [file bioengineering-13-00787-s001.zip › Supplementary Material - Performance Metrics/ALL_Decision Tree_polar.png]

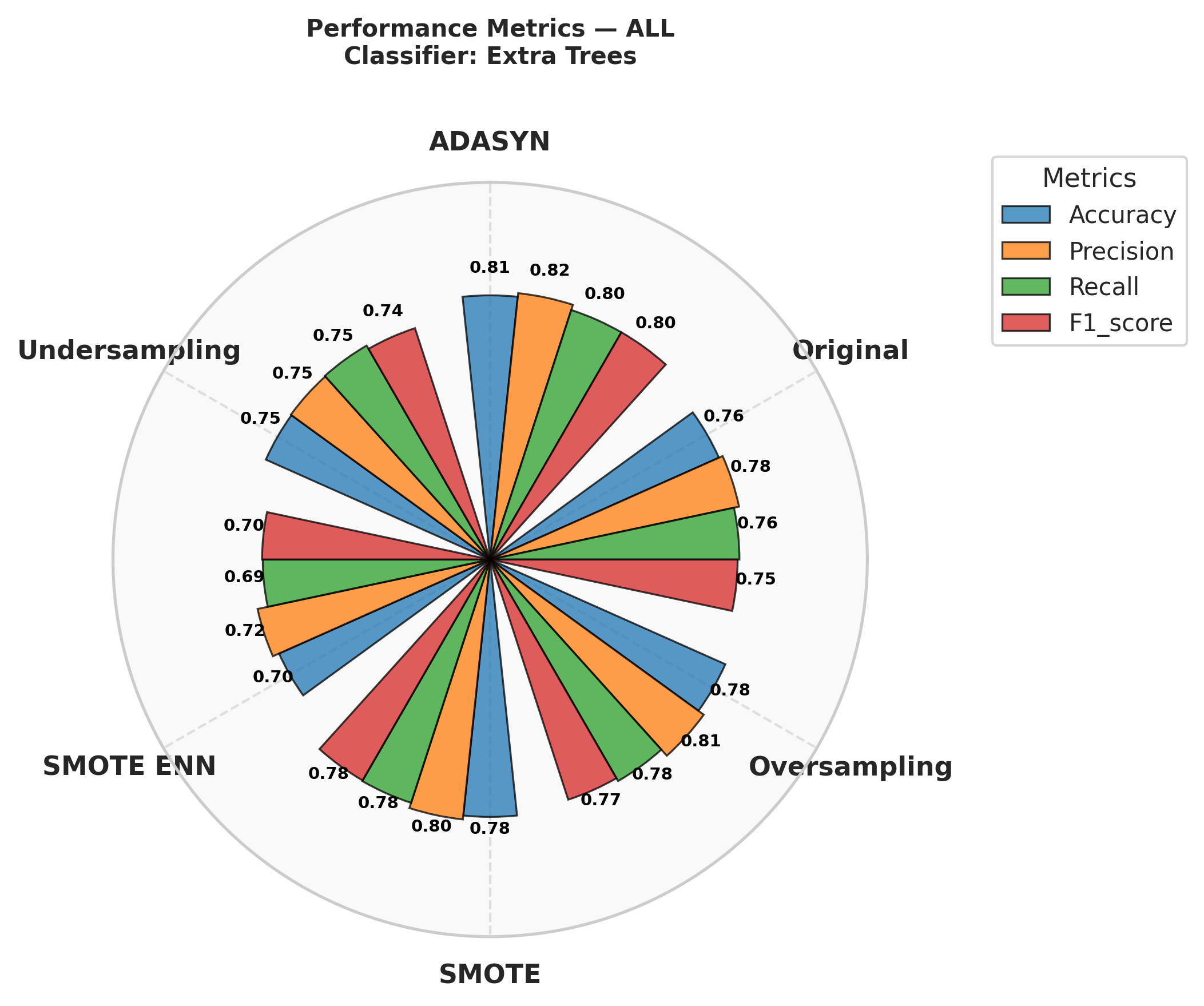

Supplement: Supplementary file 1 [file bioengineering-13-00787-s001.zip › Supplementary Material - Performance Metrics/ALL_Extra Trees_polar.png]

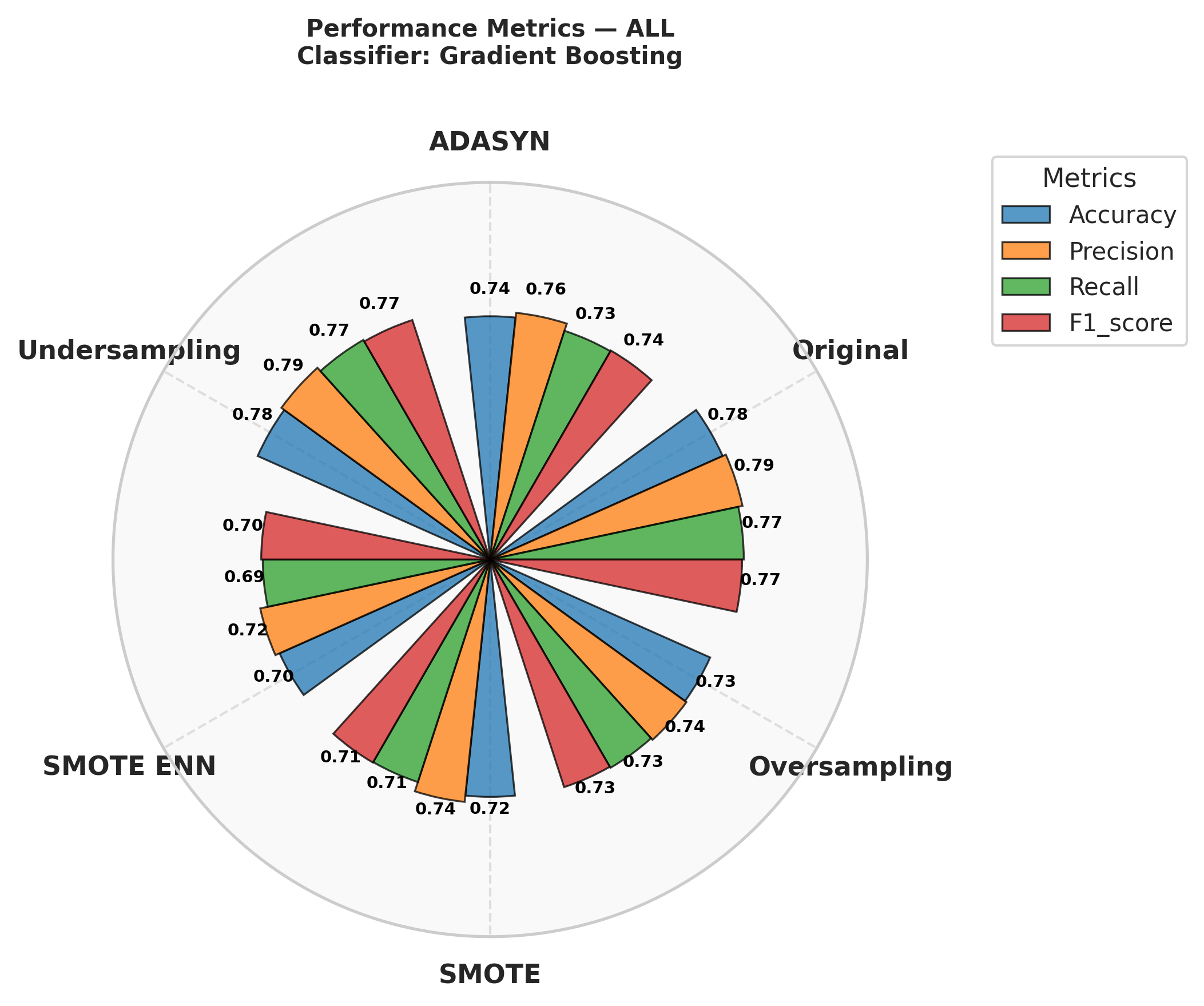

Supplement: Supplementary file 1 [file bioengineering-13-00787-s001.zip › Supplementary Material - Performance Metrics/ALL_Gradient Boosting_polar.png]

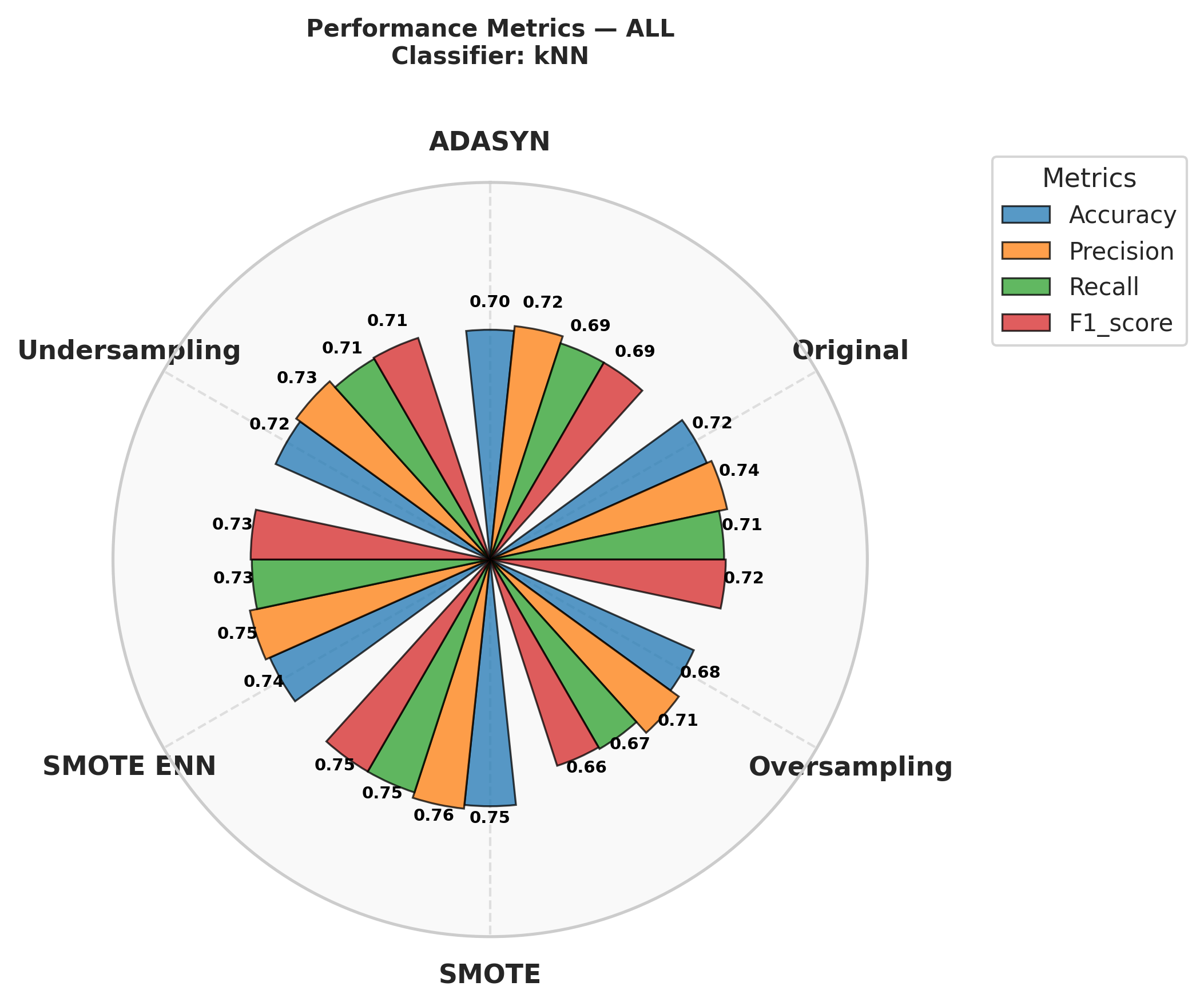

Supplement: Supplementary file 1 [file bioengineering-13-00787-s001.zip › Supplementary Material - Performance Metrics/ALL_kNN_polar.png]

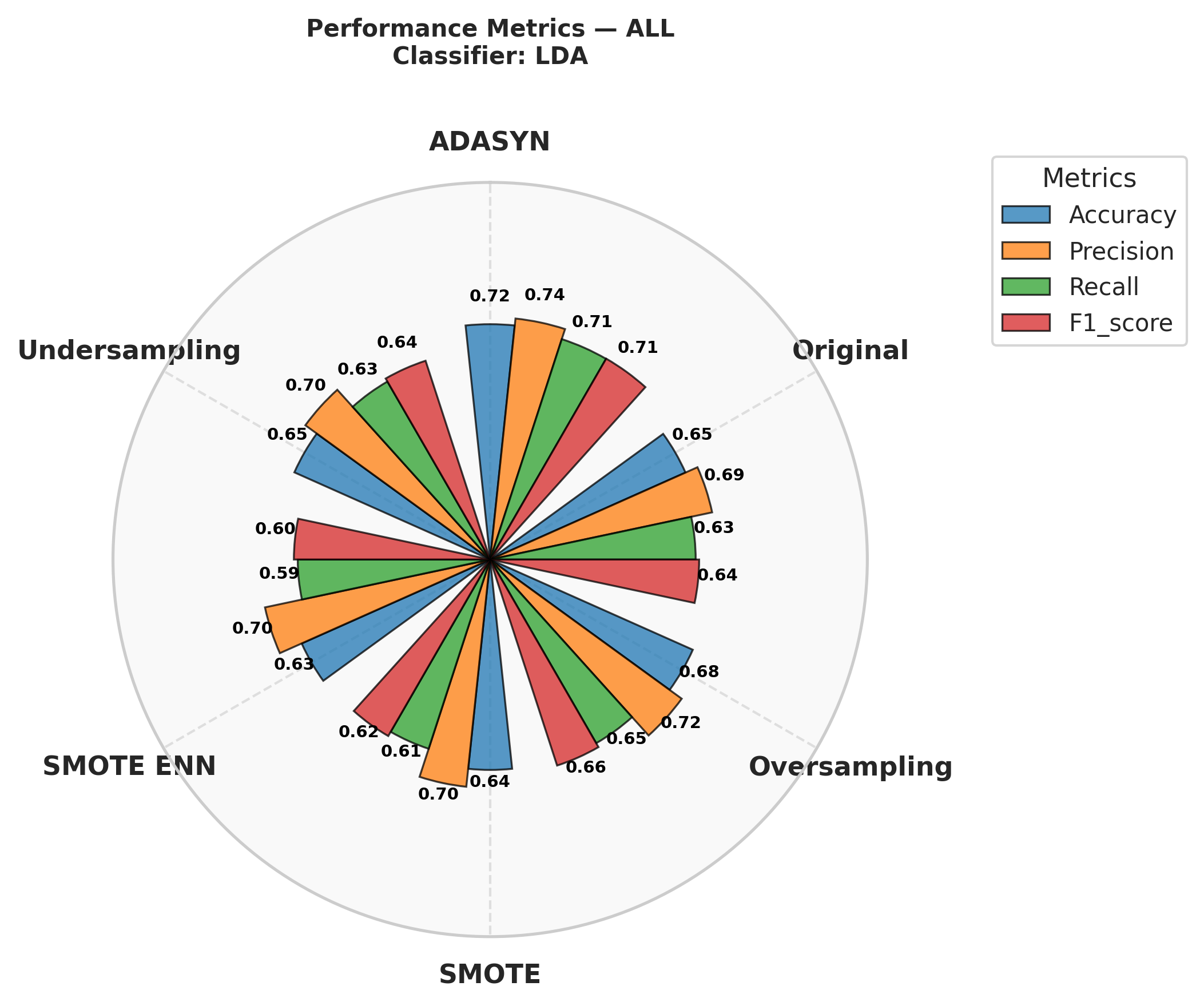

Supplement: Supplementary file 1 [file bioengineering-13-00787-s001.zip › Supplementary Material - Performance Metrics/ALL_LDA_polar.png]

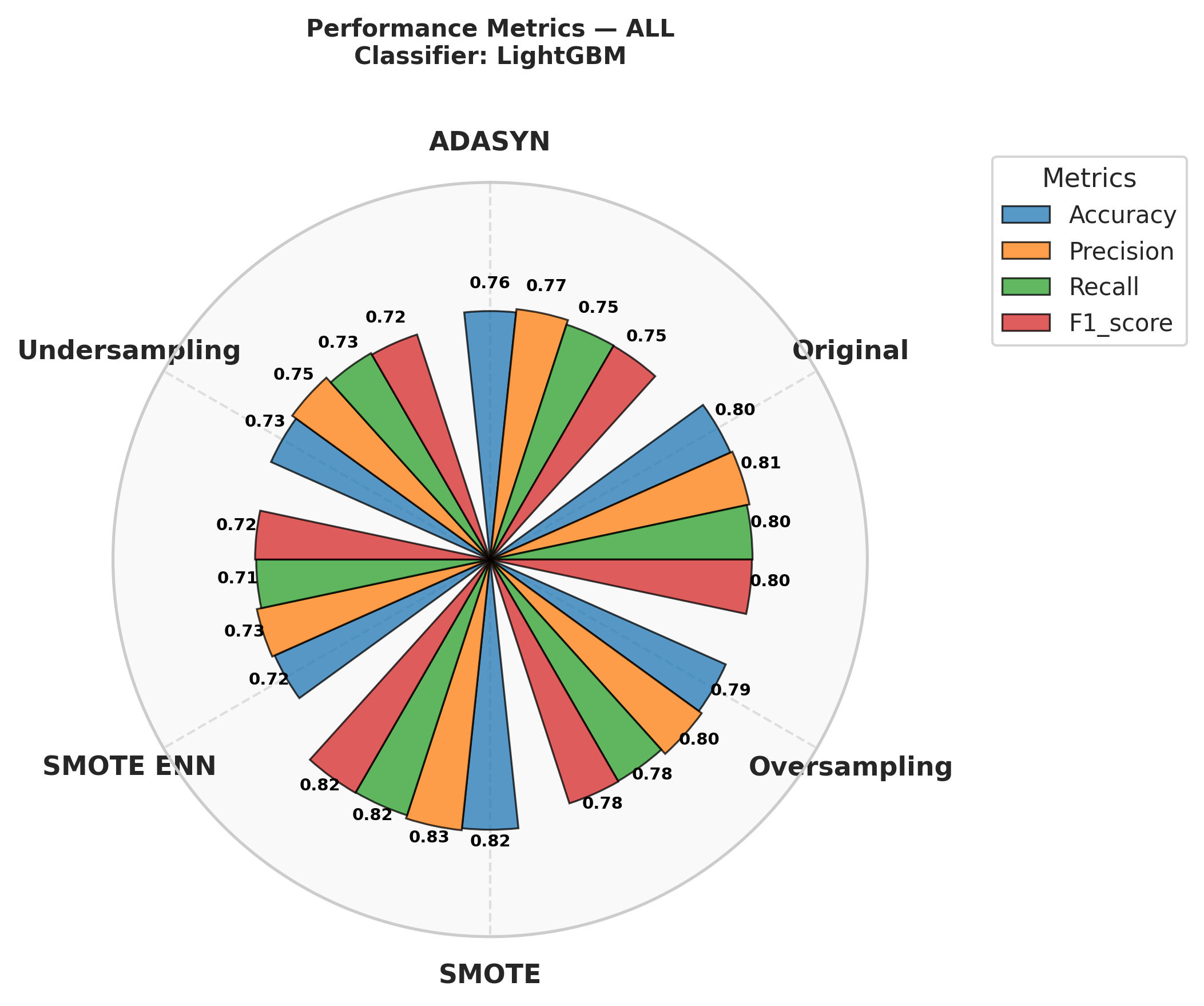

Supplement: Supplementary file 1 [file bioengineering-13-00787-s001.zip › Supplementary Material - Performance Metrics/ALL_LightGBM_polar.png]

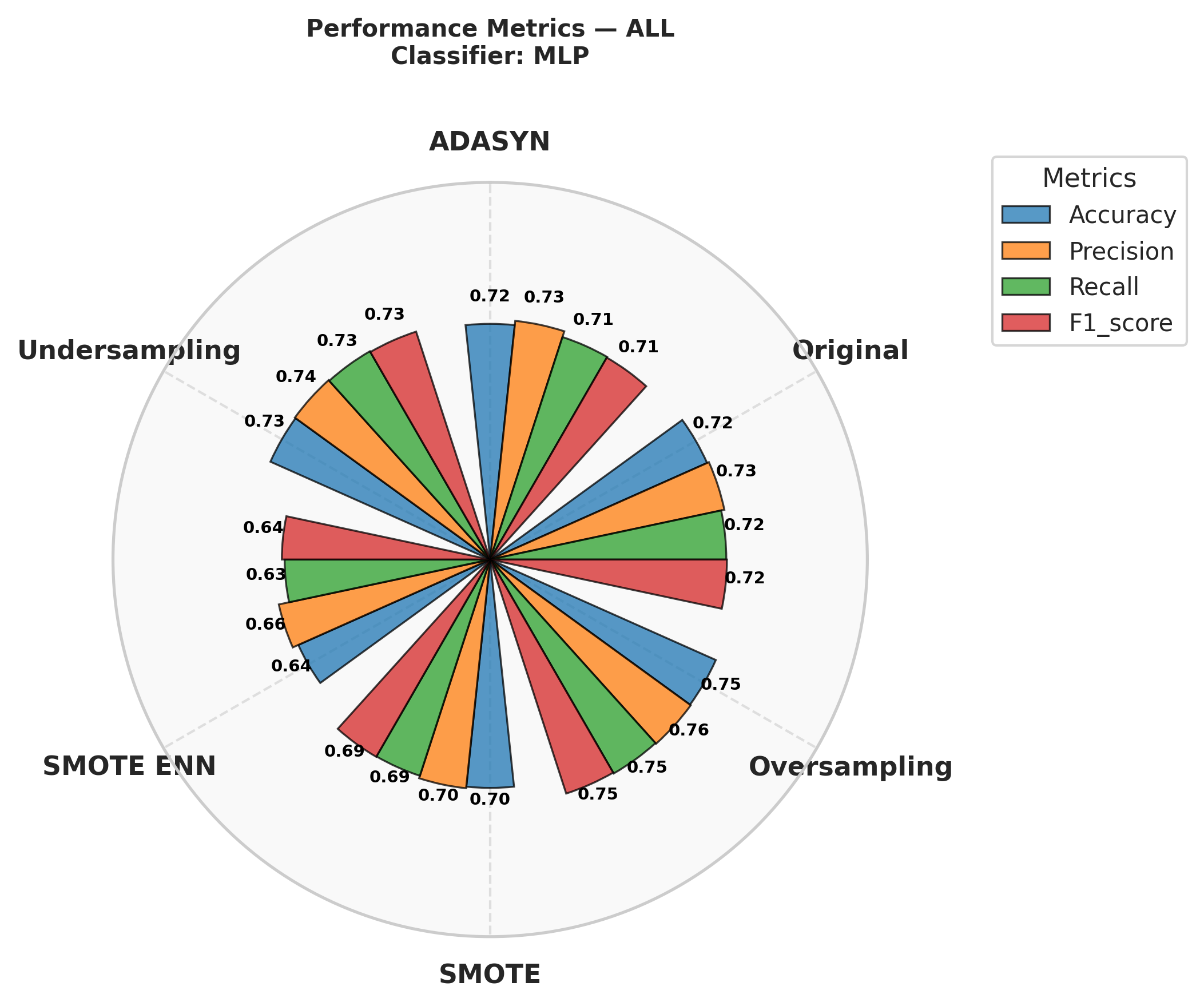

Supplement: Supplementary file 1 [file bioengineering-13-00787-s001.zip › Supplementary Material - Performance Metrics/ALL_MLP_polar.png]

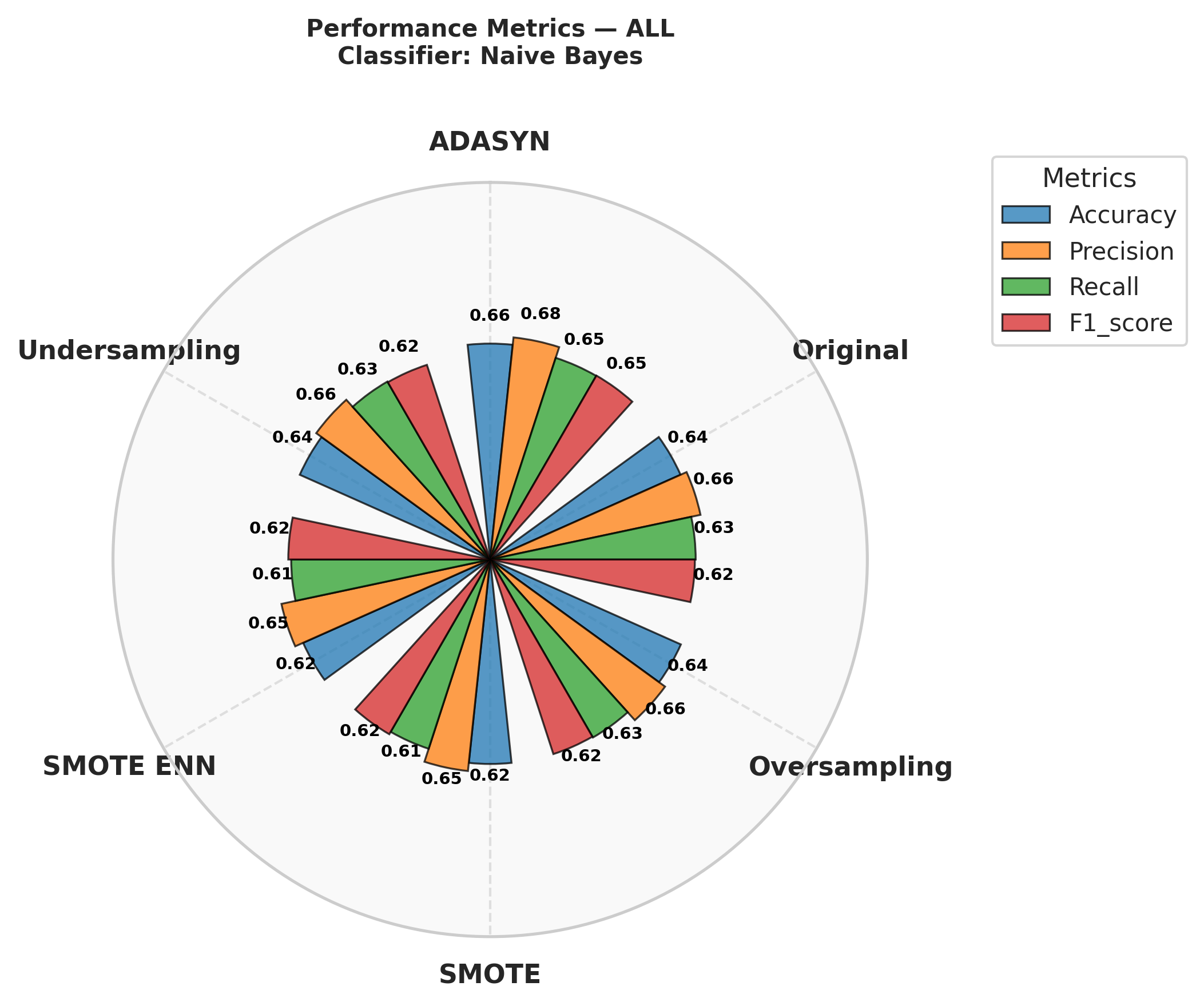

Supplement: Supplementary file 1 [file bioengineering-13-00787-s001.zip › Supplementary Material - Performance Metrics/ALL_Naive Bayes_polar.png]

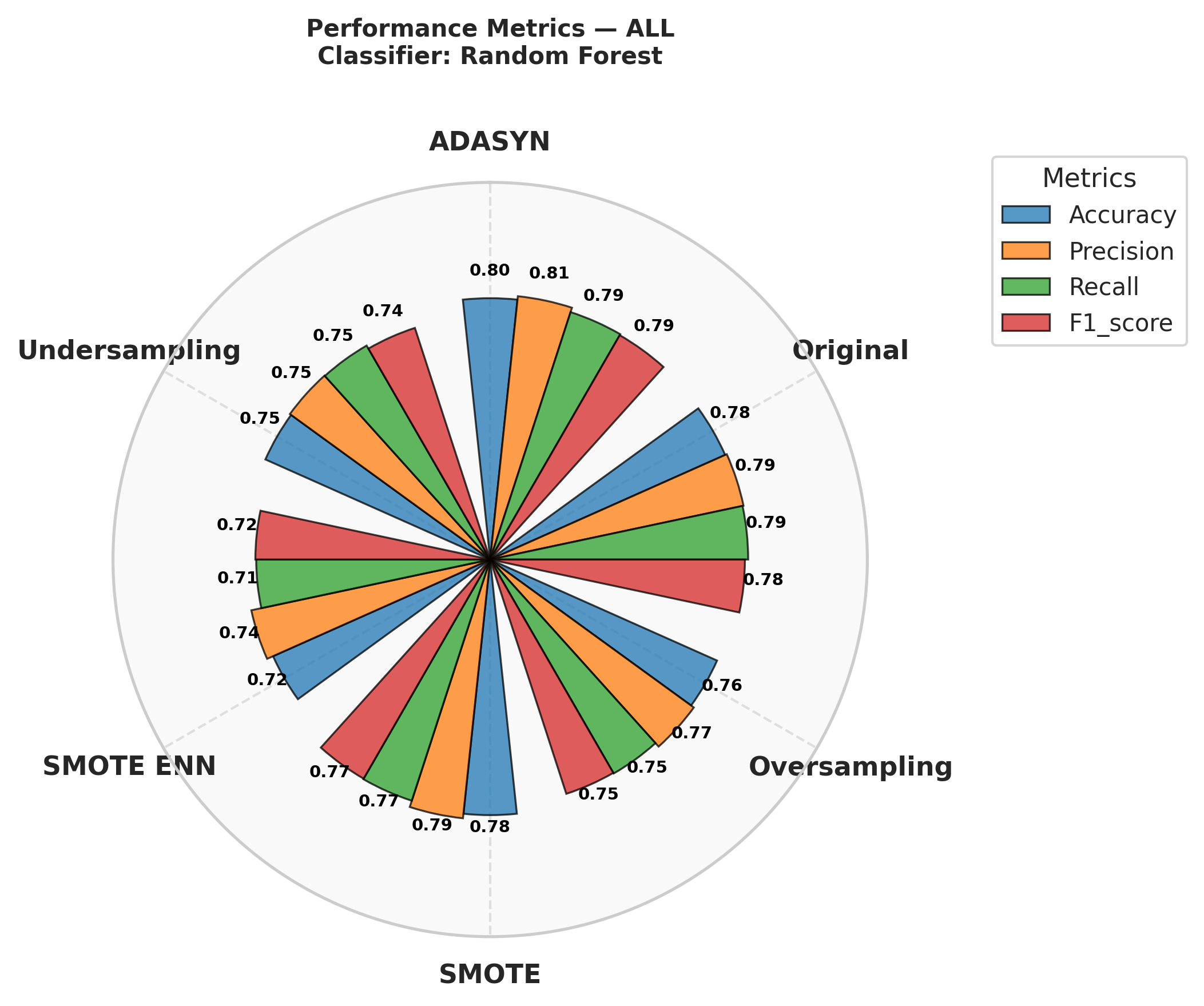

Supplement: Supplementary file 1 [file bioengineering-13-00787-s001.zip › Supplementary Material - Performance Metrics/ALL_Random Forest_polar.png]

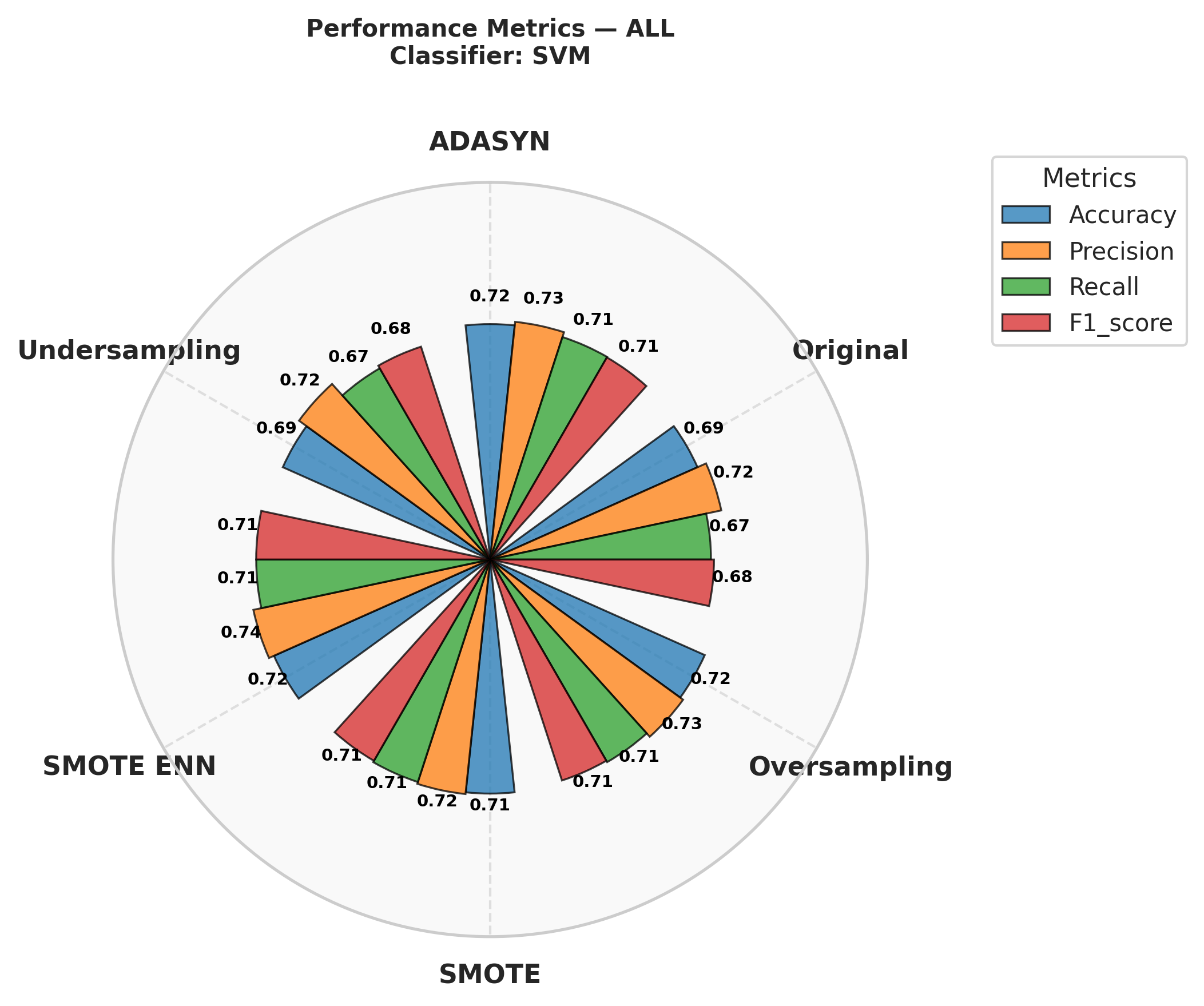

Supplement: Supplementary file 1 [file bioengineering-13-00787-s001.zip › Supplementary Material - Performance Metrics/ALL_SVM_polar.png]

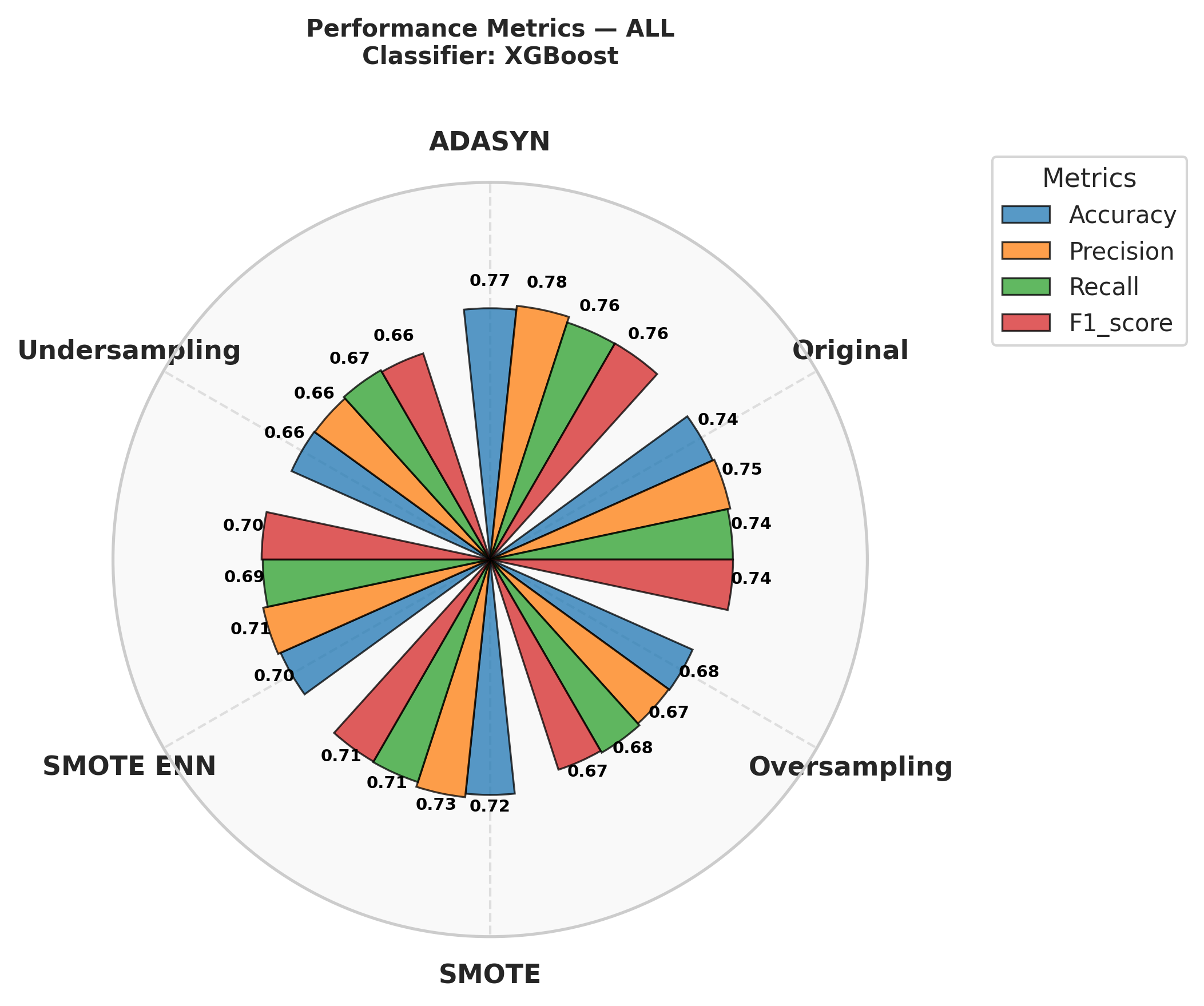

Supplement: Supplementary file 1 [file bioengineering-13-00787-s001.zip › Supplementary Material - Performance Metrics/ALL_XGBoost_polar.png]

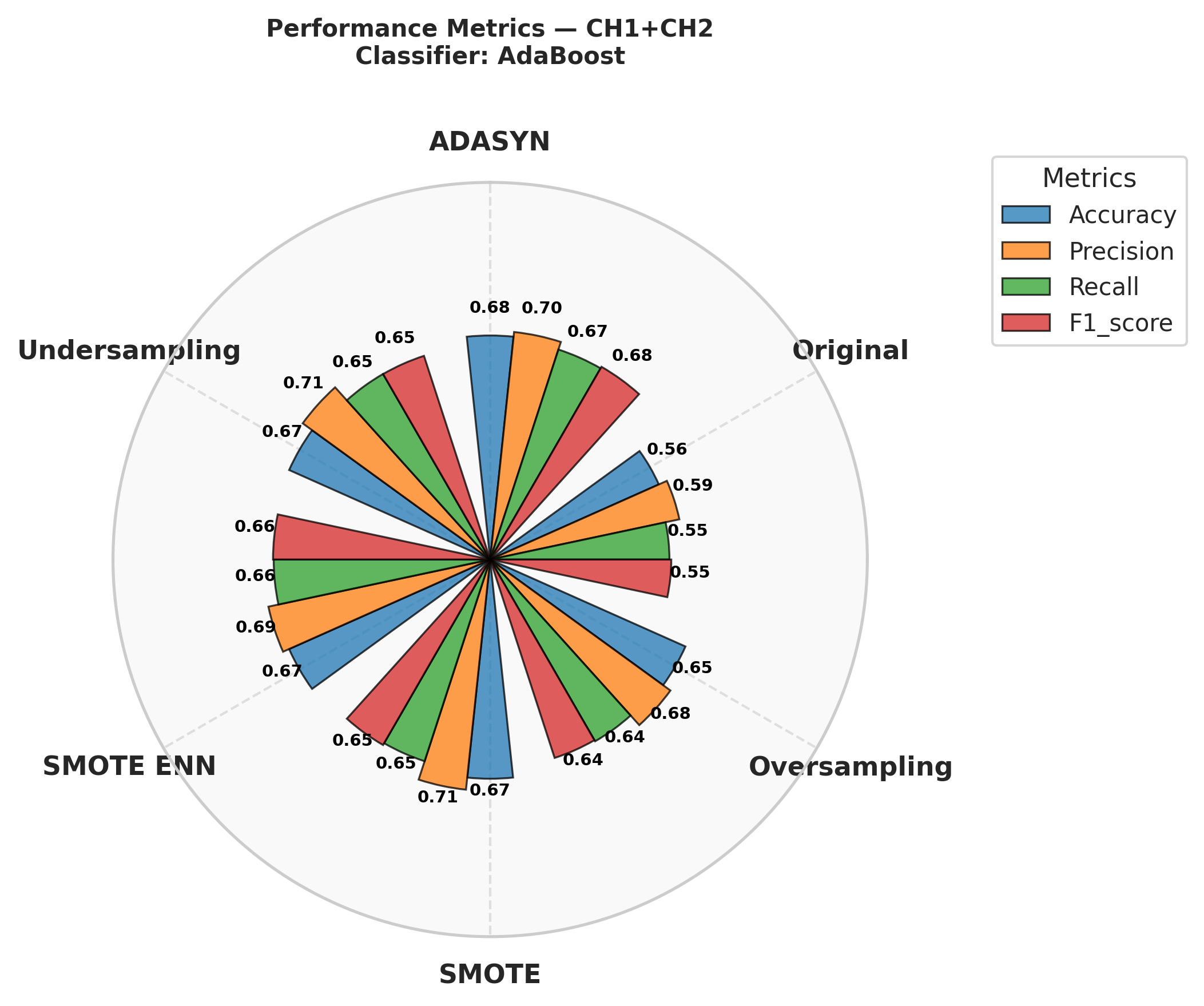

Supplement: Supplementary file 1 [file bioengineering-13-00787-s001.zip › Supplementary Material - Performance Metrics/CH1+CH2_AdaBoost_polar.png]

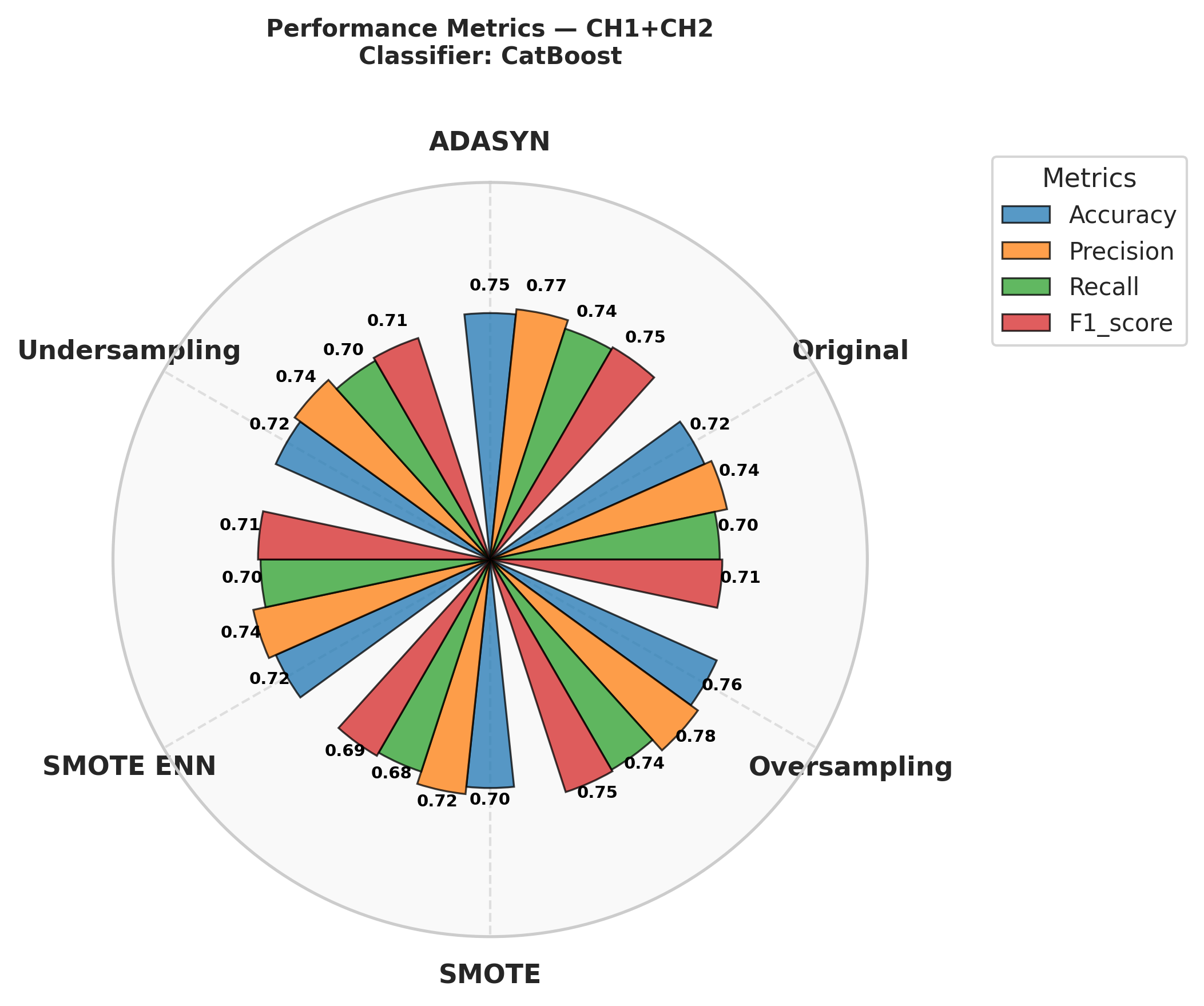

Supplement: Supplementary file 1 [file bioengineering-13-00787-s001.zip › Supplementary Material - Performance Metrics/CH1+CH2_CatBoost_polar.png]

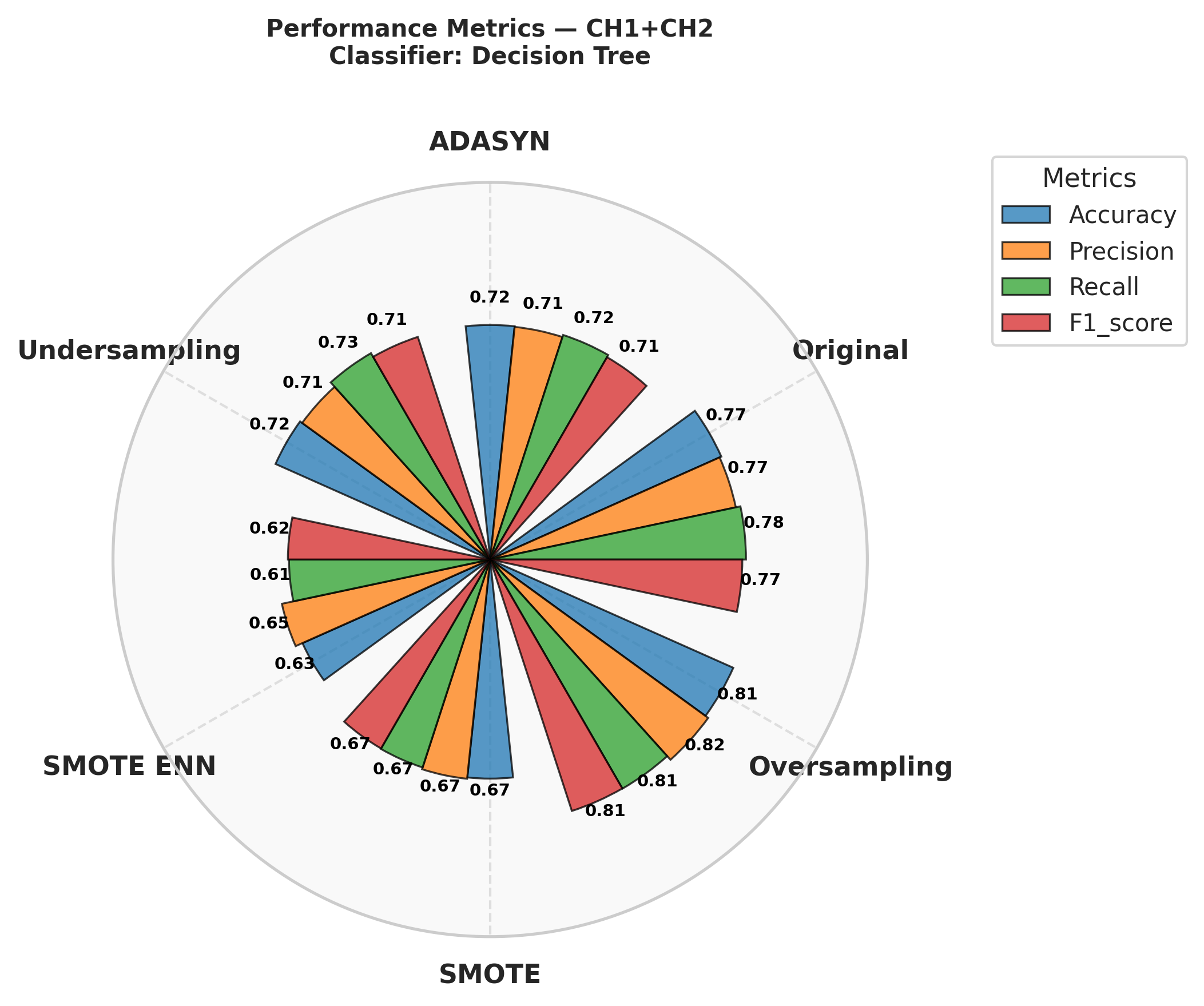

Supplement: Supplementary file 1 [file bioengineering-13-00787-s001.zip › Supplementary Material - Performance Metrics/CH1+CH2_Decision Tree_polar.png]

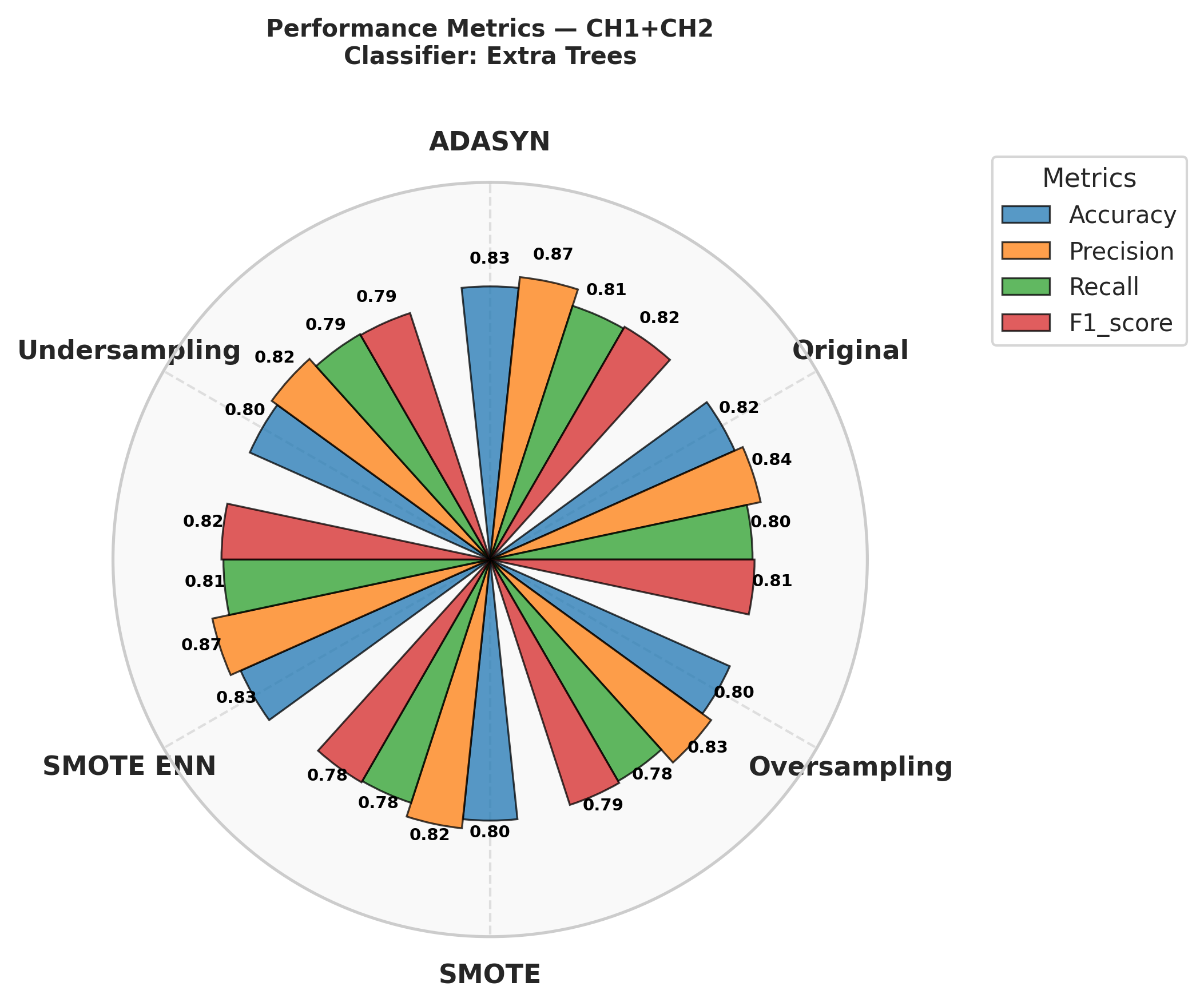

Supplement: Supplementary file 1 [file bioengineering-13-00787-s001.zip › Supplementary Material - Performance Metrics/CH1+CH2_Extra Trees_polar.png]

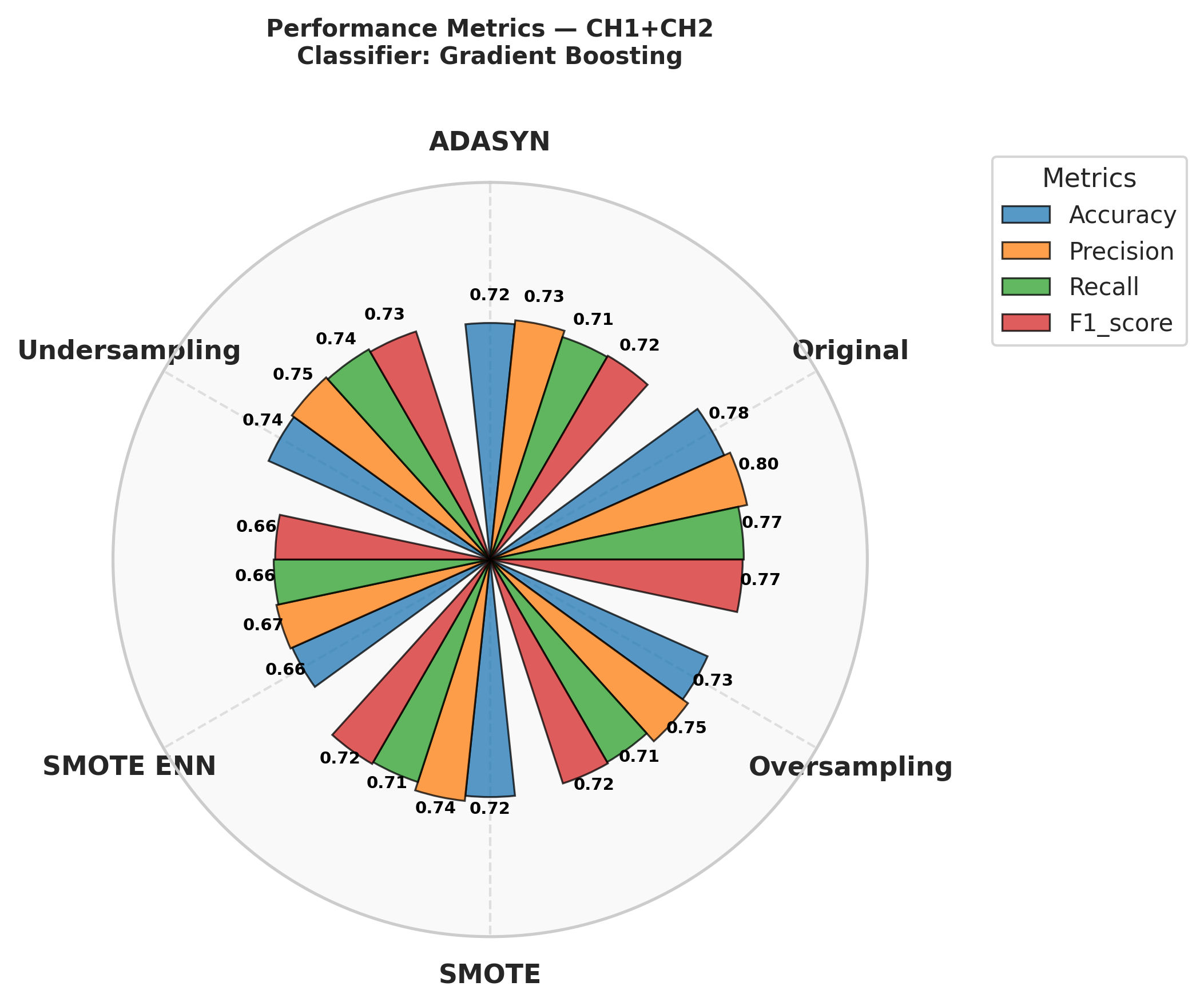

Supplement: Supplementary file 1 [file bioengineering-13-00787-s001.zip › Supplementary Material - Performance Metrics/CH1+CH2_Gradient Boosting_polar.png]

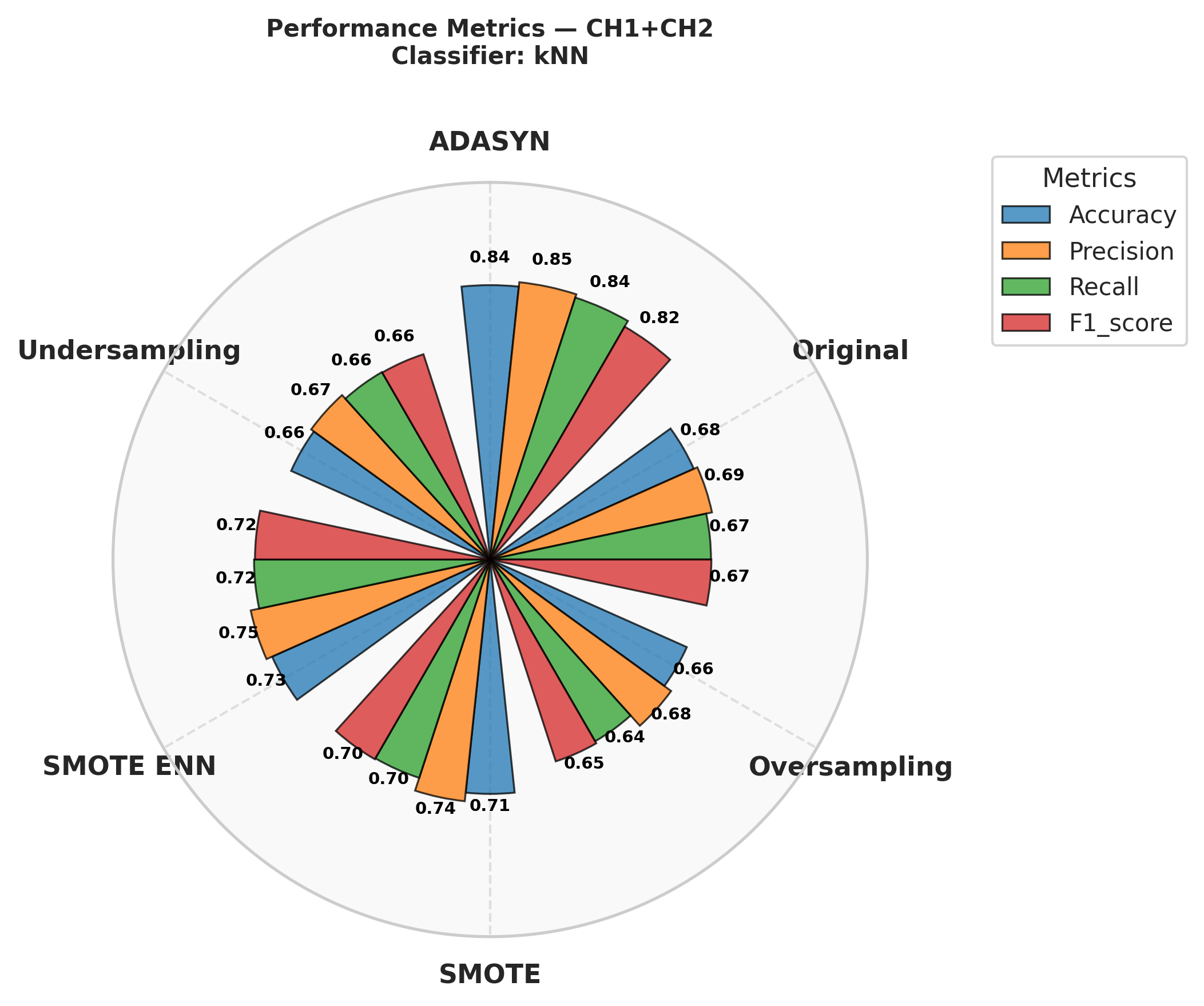

Supplement: Supplementary file 1 [file bioengineering-13-00787-s001.zip › Supplementary Material - Performance Metrics/CH1+CH2_kNN_polar.png]

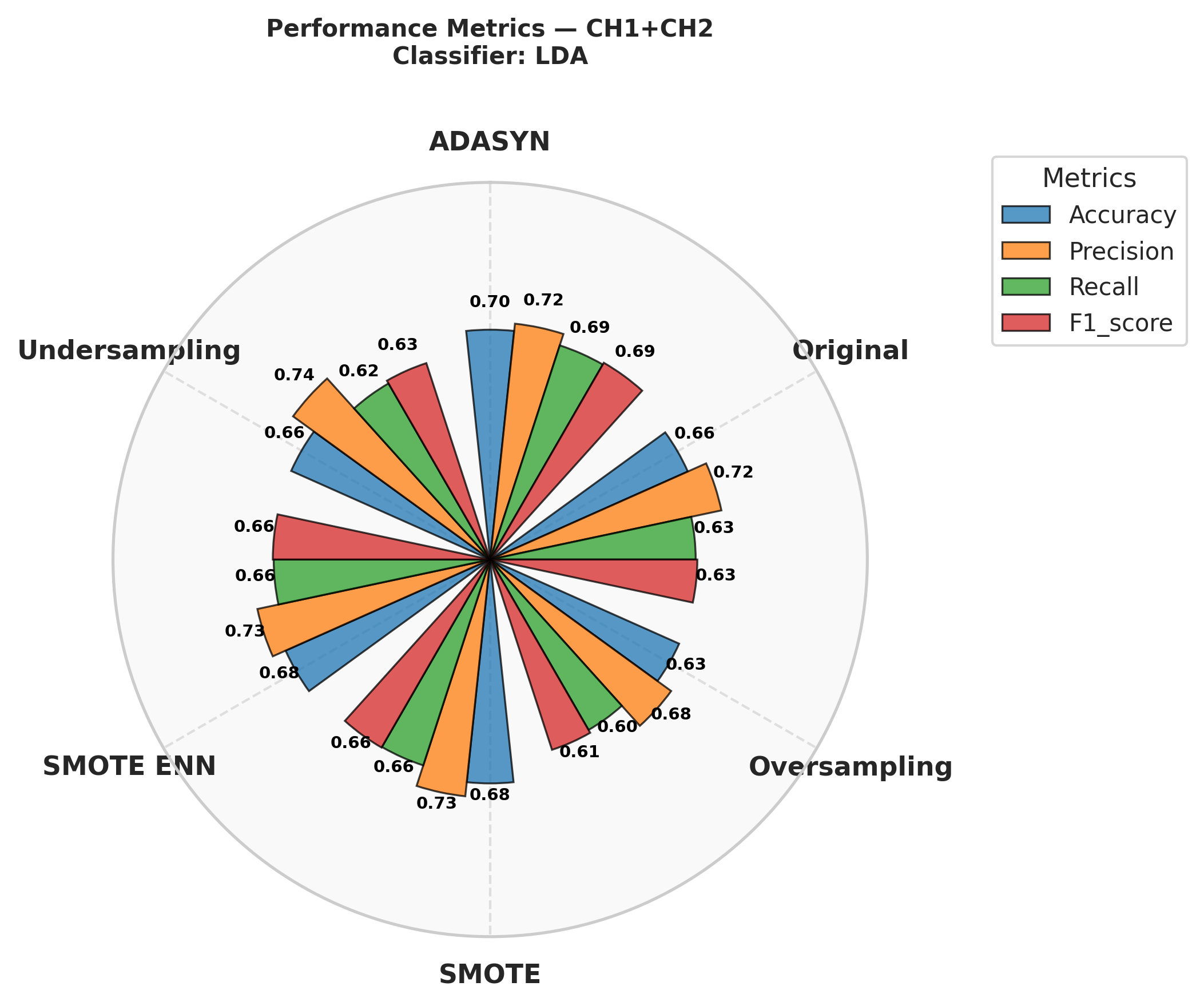

Supplement: Supplementary file 1 [file bioengineering-13-00787-s001.zip › Supplementary Material - Performance Metrics/CH1+CH2_LDA_polar.png]

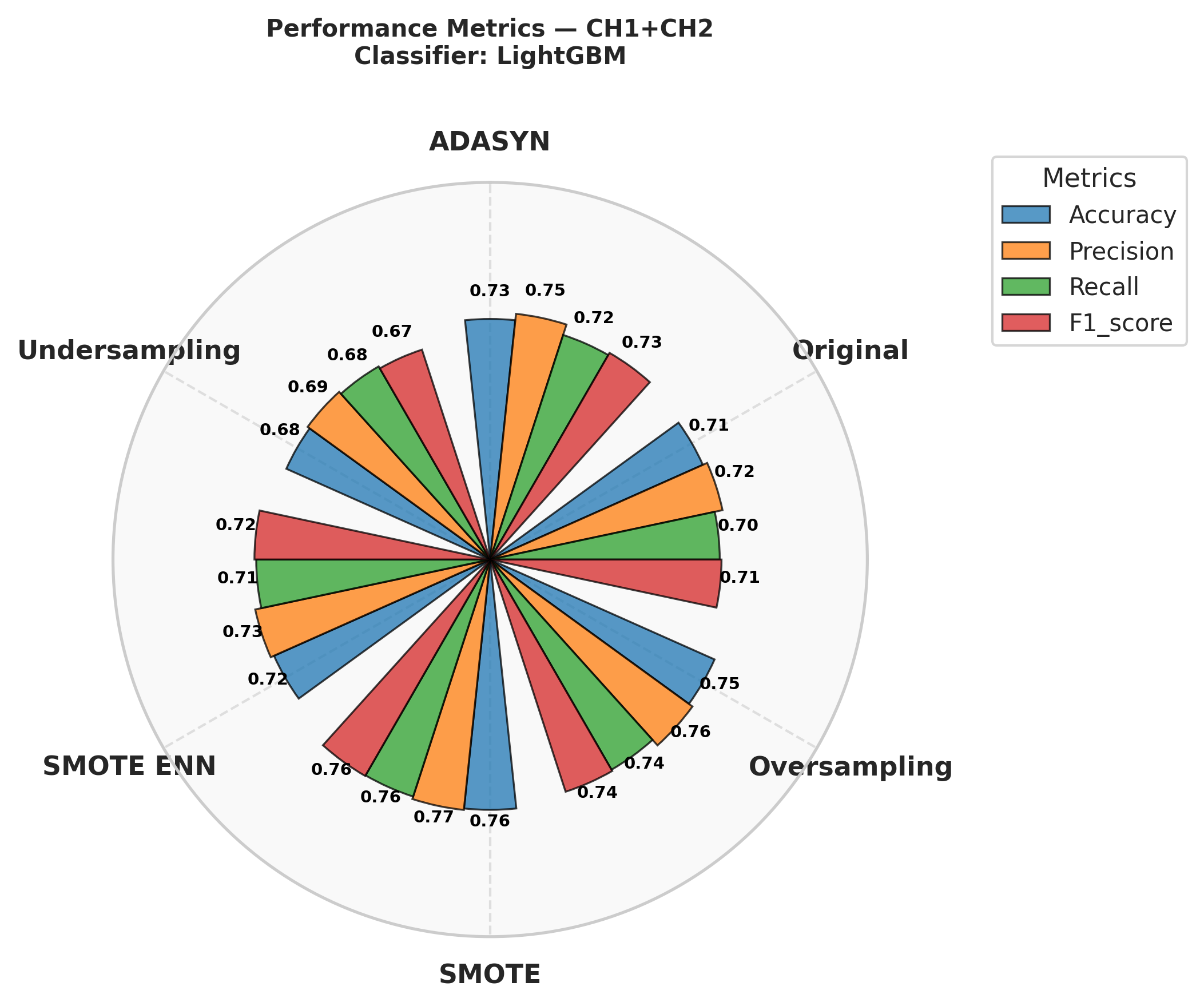

Supplement: Supplementary file 1 [file bioengineering-13-00787-s001.zip › Supplementary Material - Performance Metrics/CH1+CH2_LightGBM_polar.png]

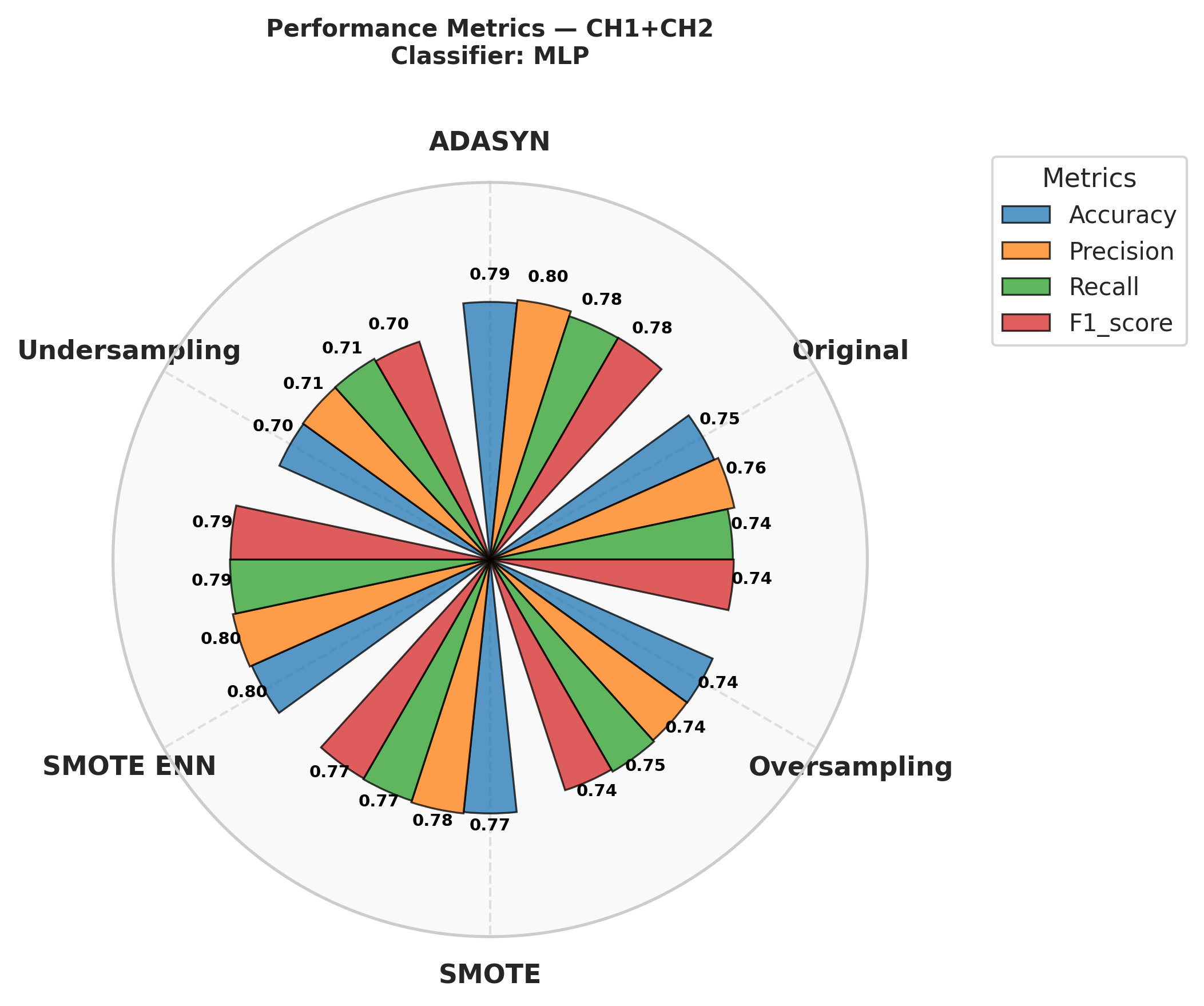

Supplement: Supplementary file 1 [file bioengineering-13-00787-s001.zip › Supplementary Material - Performance Metrics/CH1+CH2_MLP_polar.png]

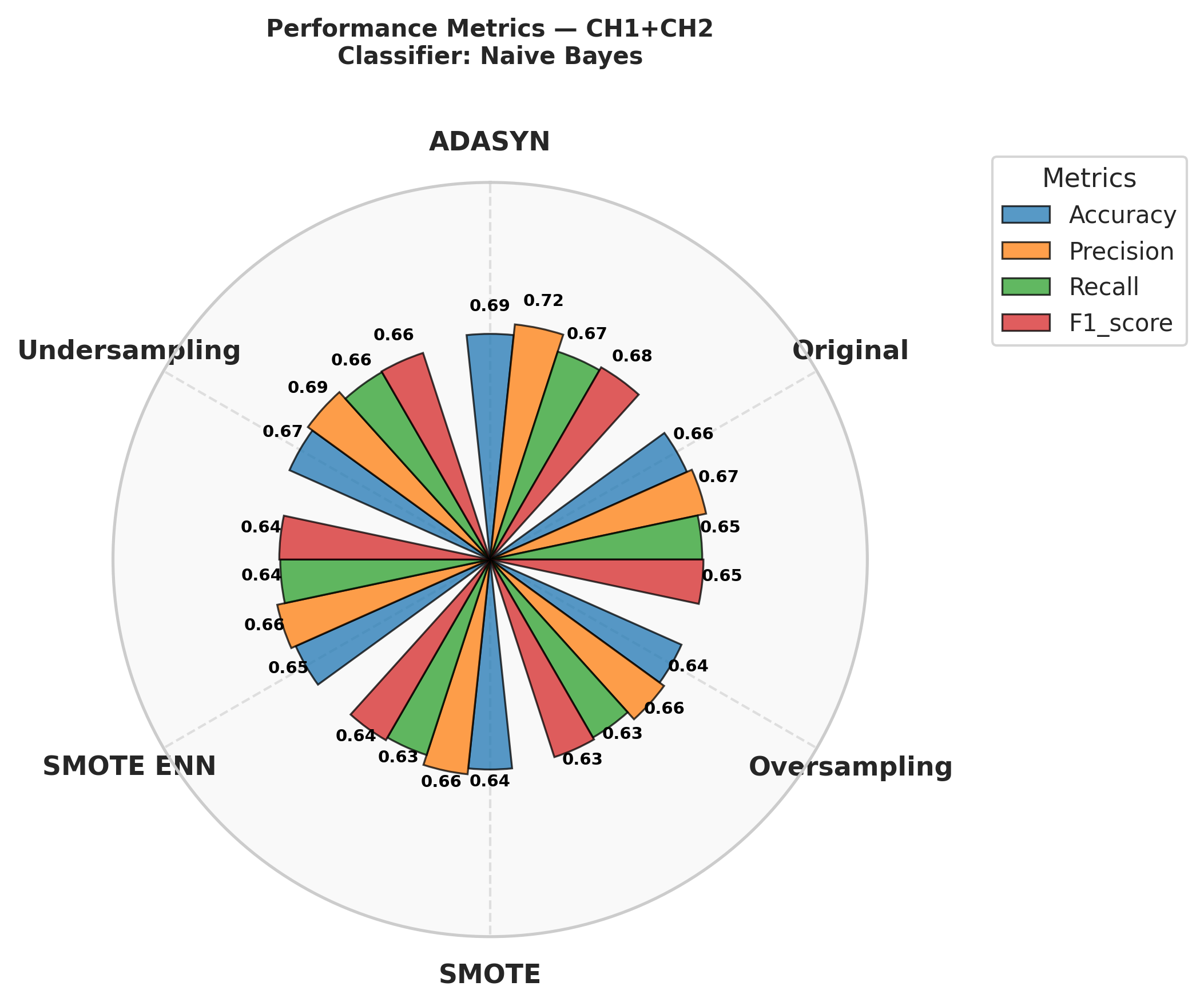

Supplement: Supplementary file 1 [file bioengineering-13-00787-s001.zip › Supplementary Material - Performance Metrics/CH1+CH2_Naive Bayes_polar.png]

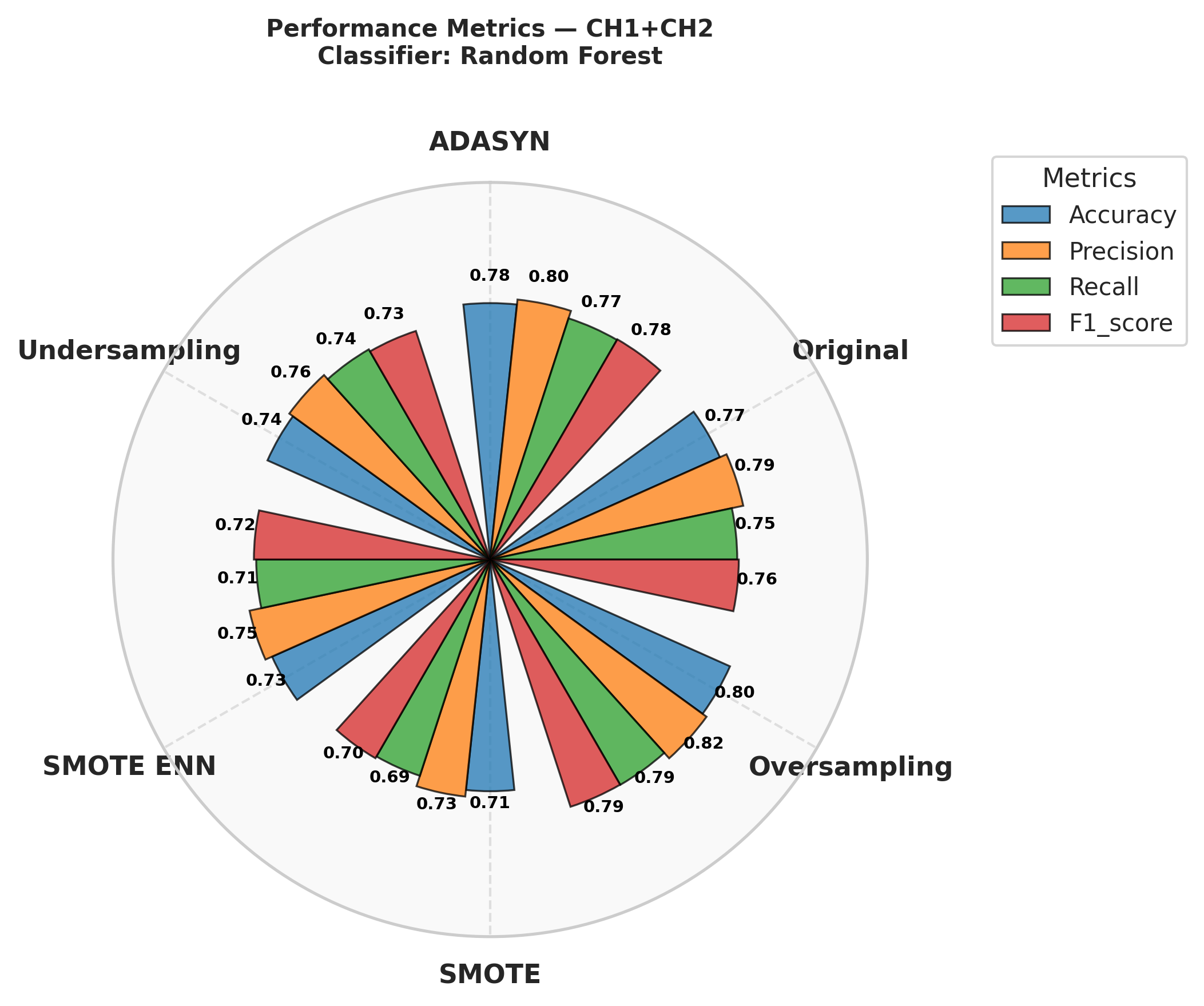

Supplement: Supplementary file 1 [file bioengineering-13-00787-s001.zip › Supplementary Material - Performance Metrics/CH1+CH2_Random Forest_polar.png]

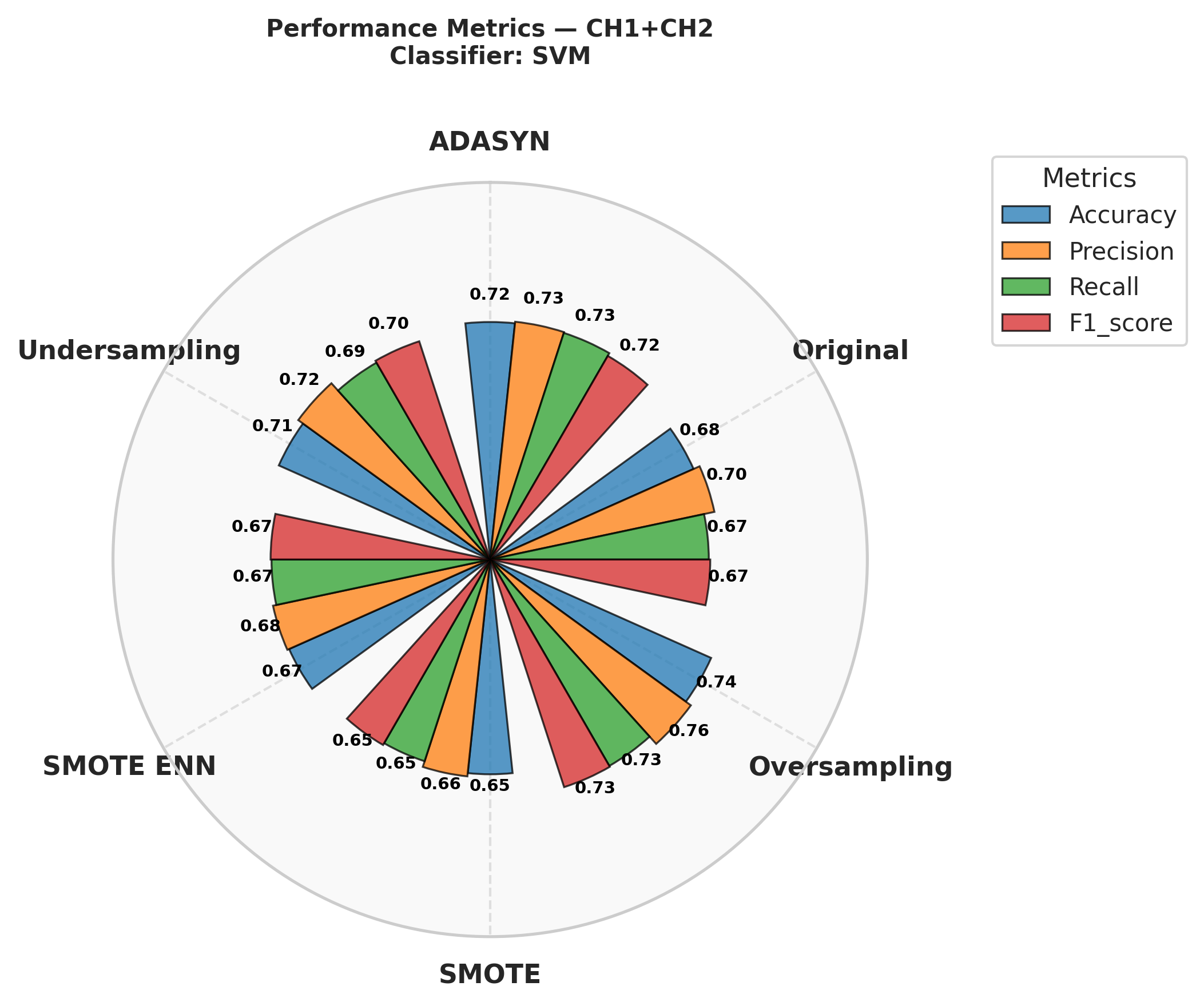

Supplement: Supplementary file 1 [file bioengineering-13-00787-s001.zip › Supplementary Material - Performance Metrics/CH1+CH2_SVM_polar.png]

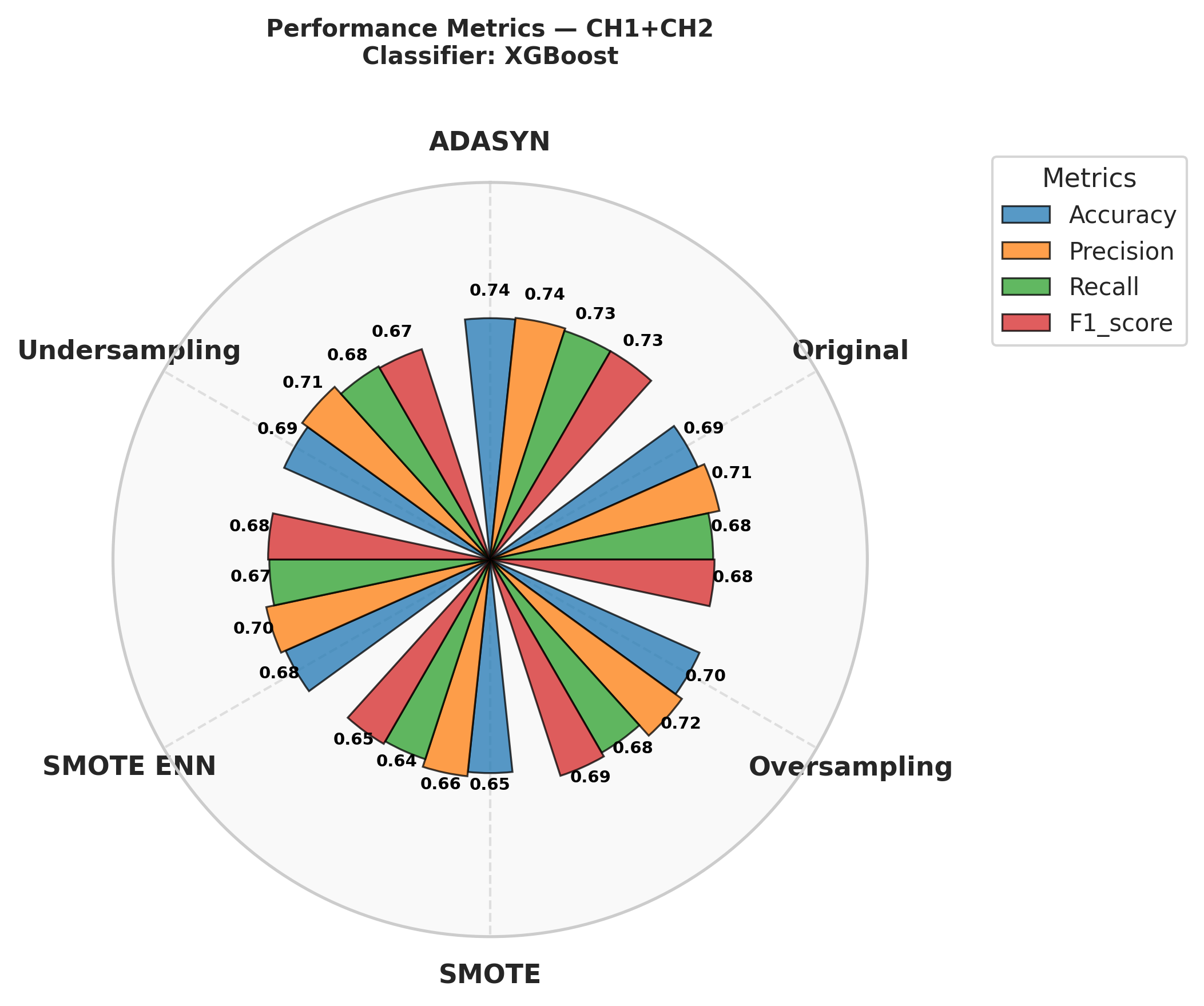

Supplement: Supplementary file 1 [file bioengineering-13-00787-s001.zip › Supplementary Material - Performance Metrics/CH1+CH2_XGBoost_polar.png]

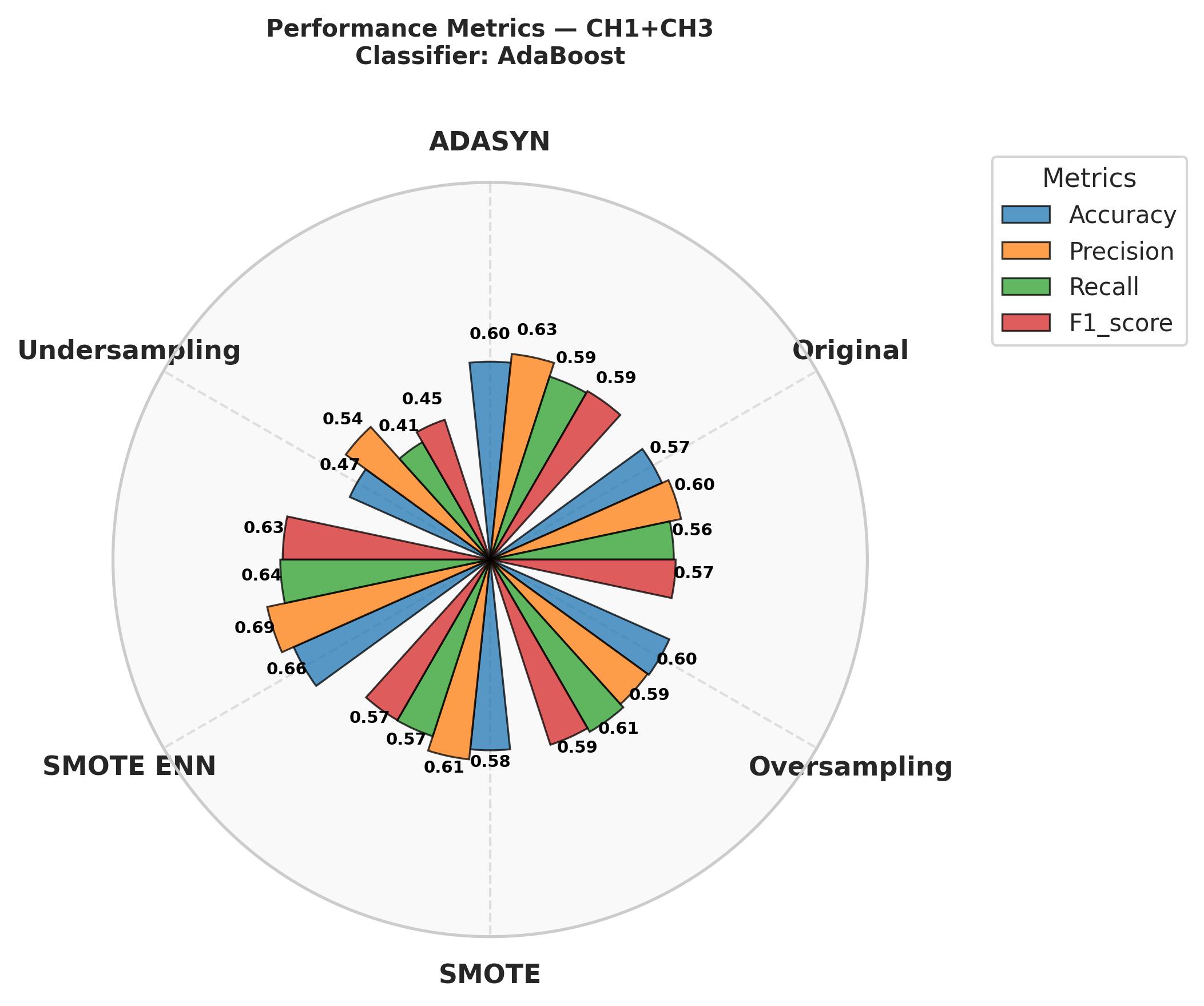

Supplement: Supplementary file 1 [file bioengineering-13-00787-s001.zip › Supplementary Material - Performance Metrics/CH1+CH3_AdaBoost_polar.png]

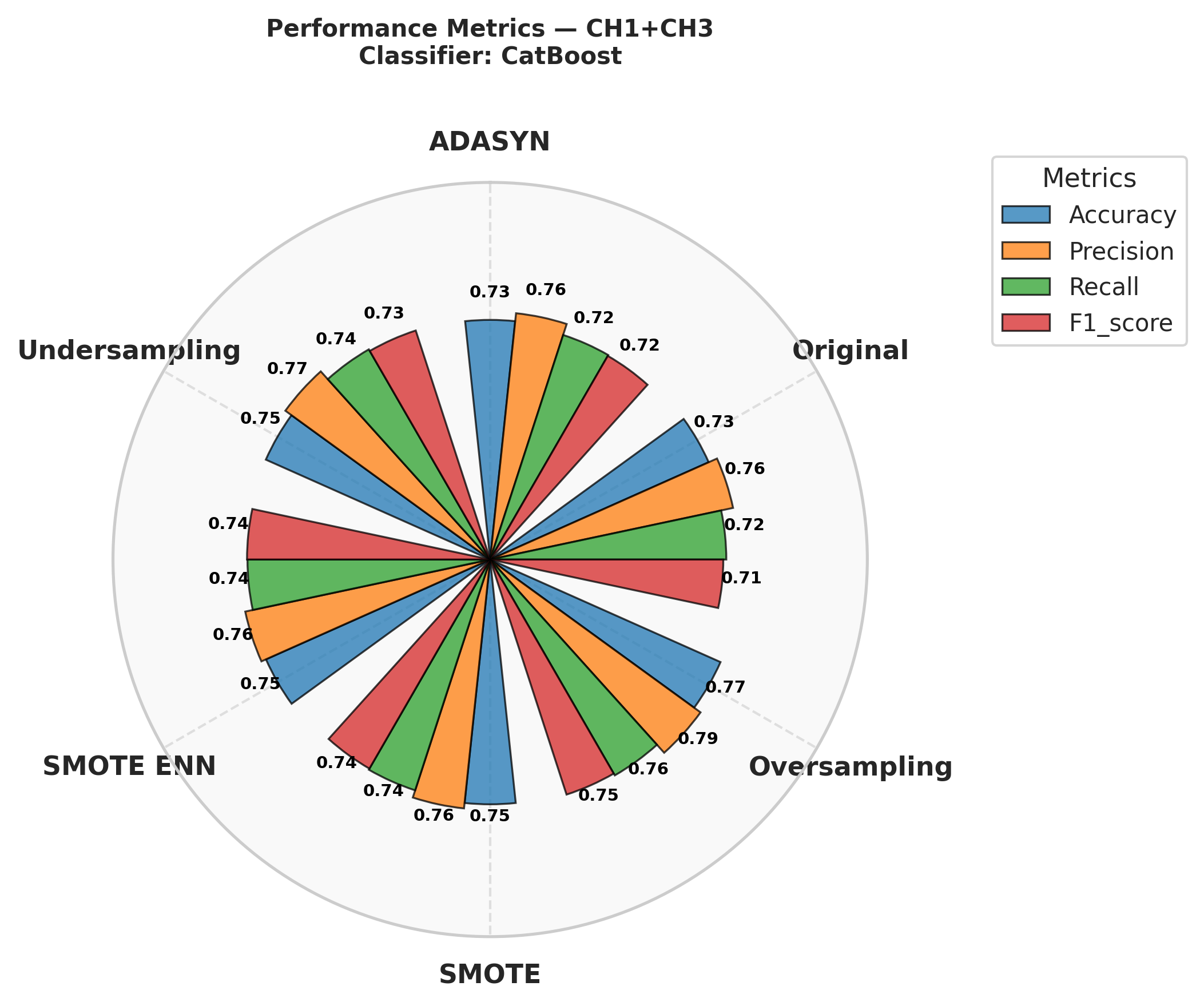

Supplement: Supplementary file 1 [file bioengineering-13-00787-s001.zip › Supplementary Material - Performance Metrics/CH1+CH3_CatBoost_polar.png]

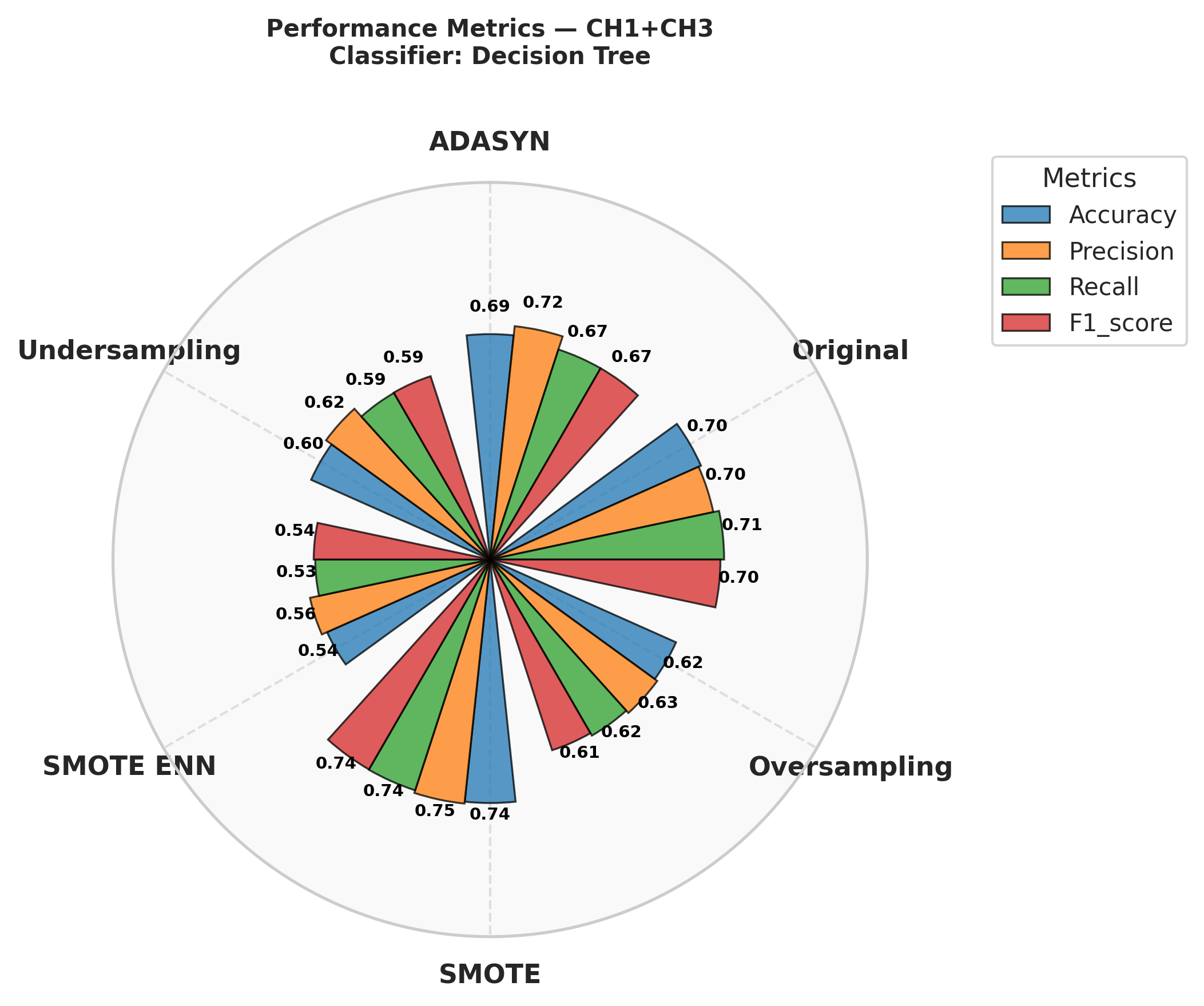

Supplement: Supplementary file 1 [file bioengineering-13-00787-s001.zip › Supplementary Material - Performance Metrics/CH1+CH3_Decision Tree_polar.png]

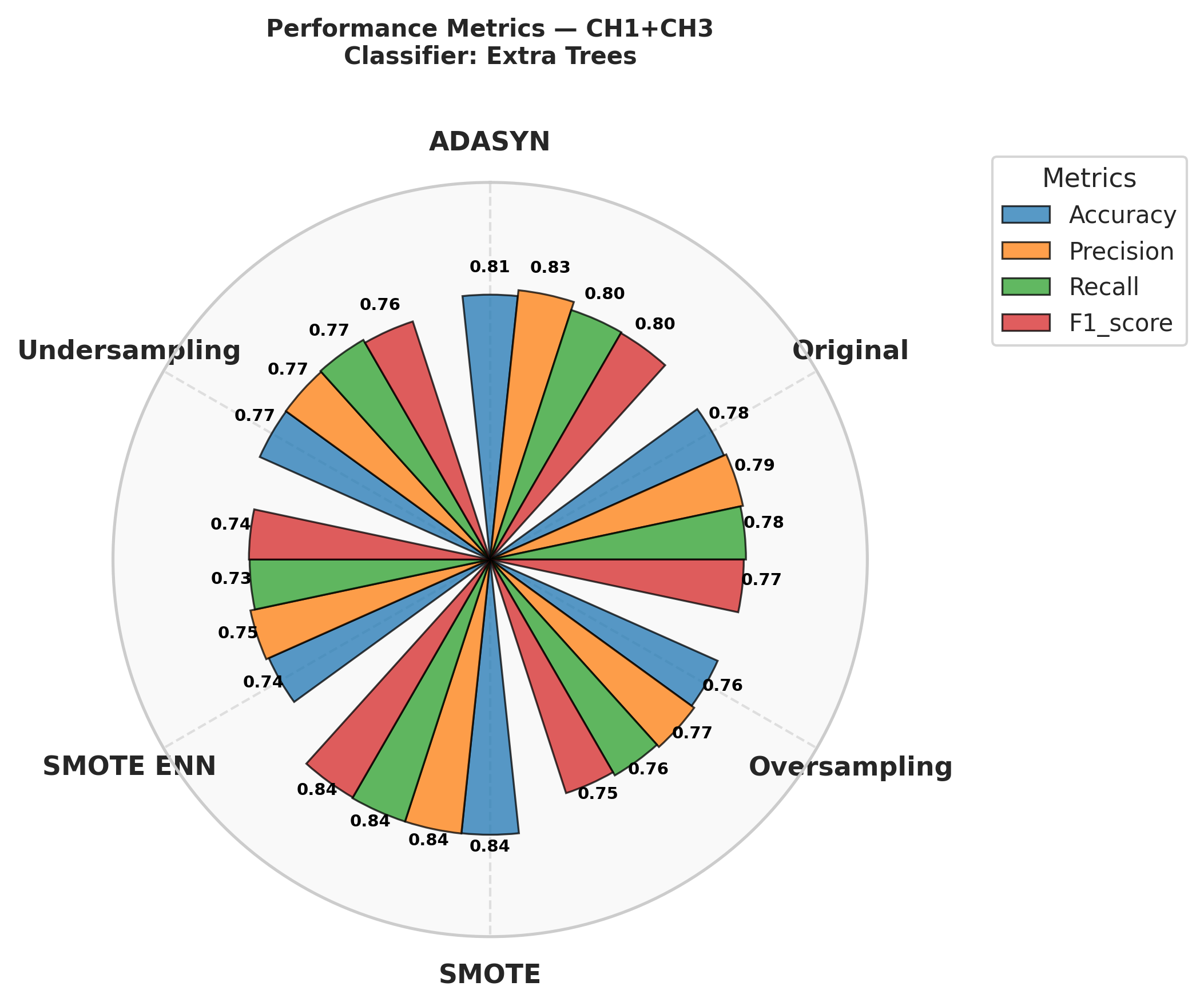

Supplement: Supplementary file 1 [file bioengineering-13-00787-s001.zip › Supplementary Material - Performance Metrics/CH1+CH3_Extra Trees_polar.png]

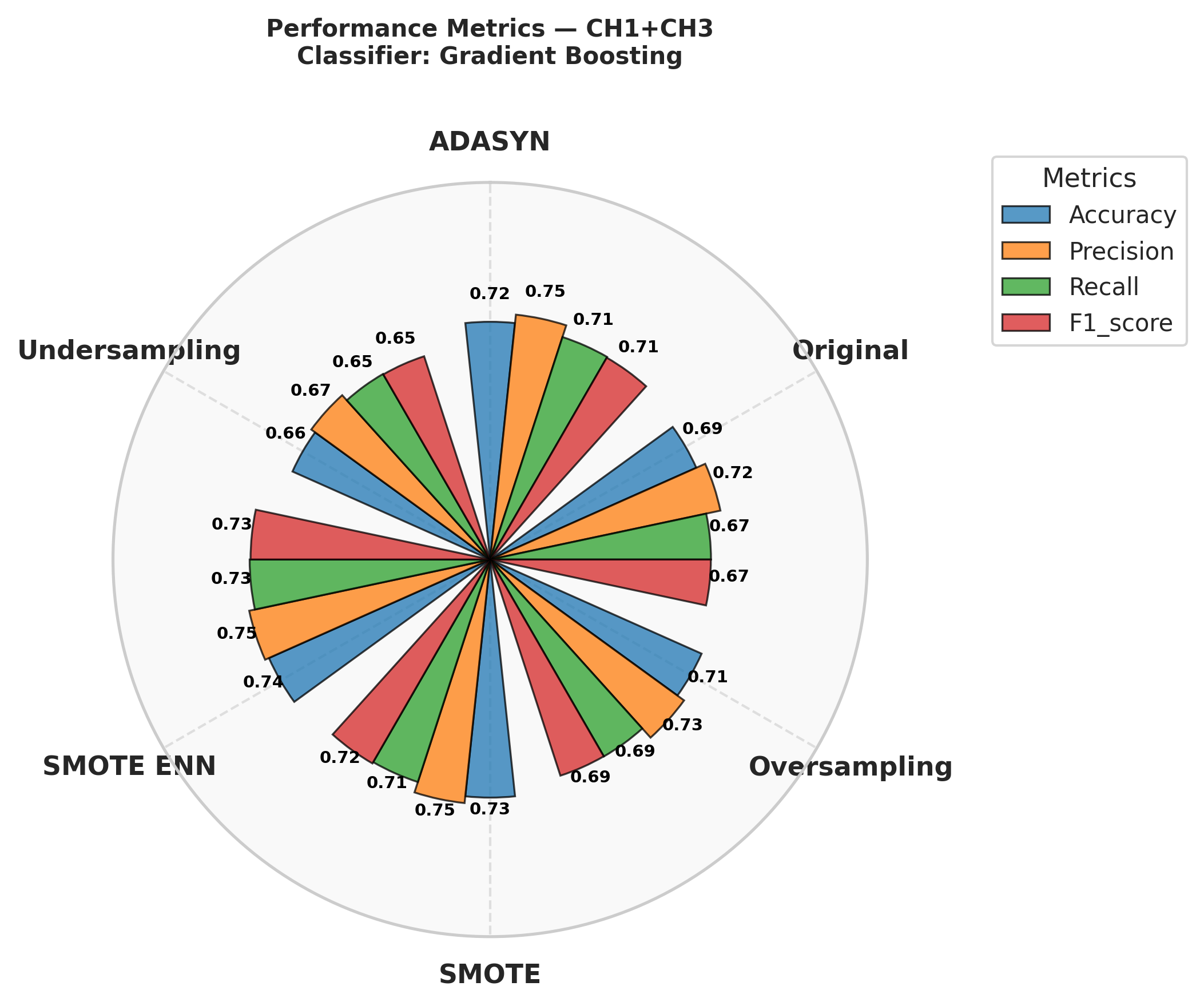

Supplement: Supplementary file 1 [file bioengineering-13-00787-s001.zip › Supplementary Material - Performance Metrics/CH1+CH3_Gradient Boosting_polar.png]

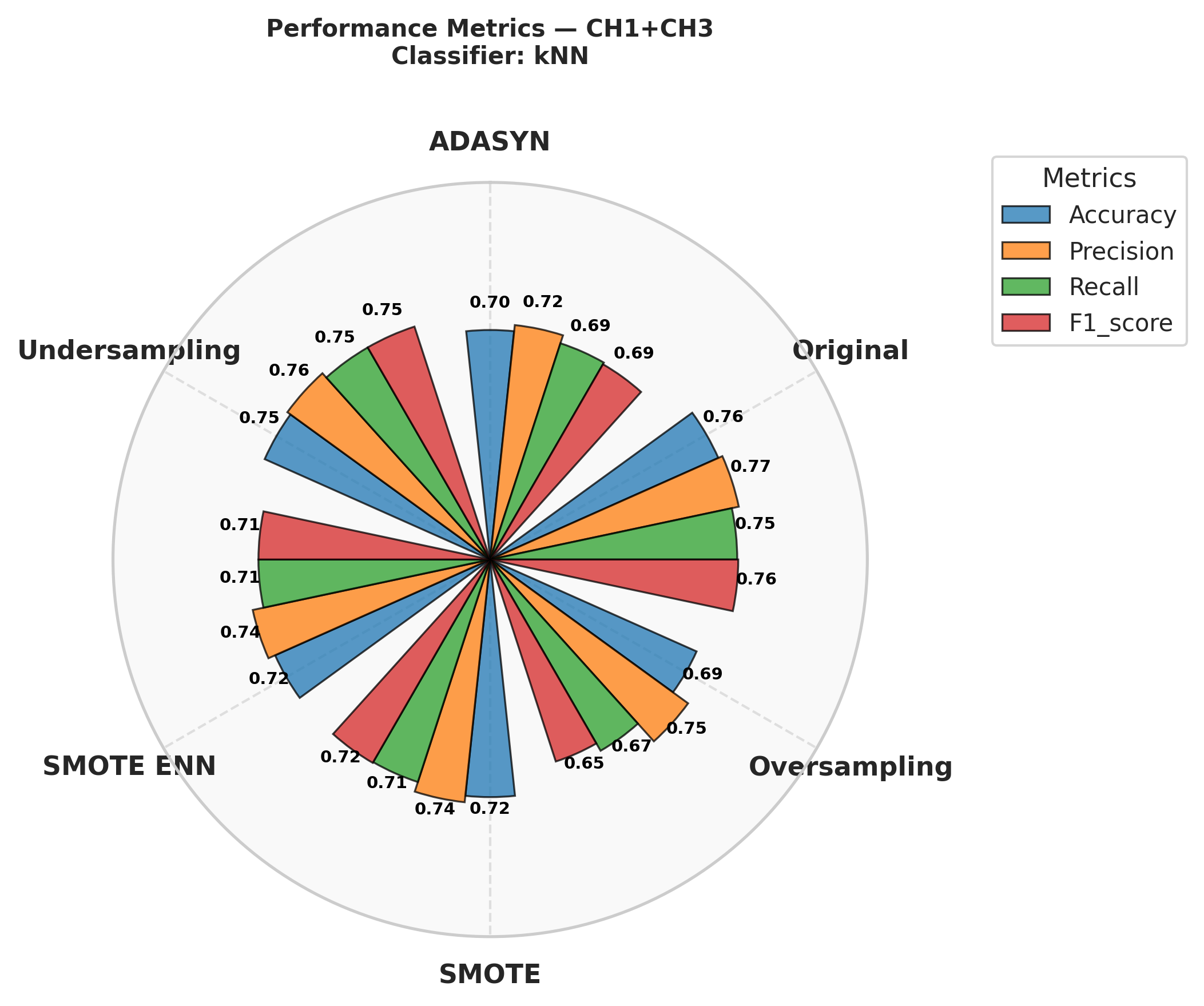

Supplement: Supplementary file 1 [file bioengineering-13-00787-s001.zip › Supplementary Material - Performance Metrics/CH1+CH3_kNN_polar.png]

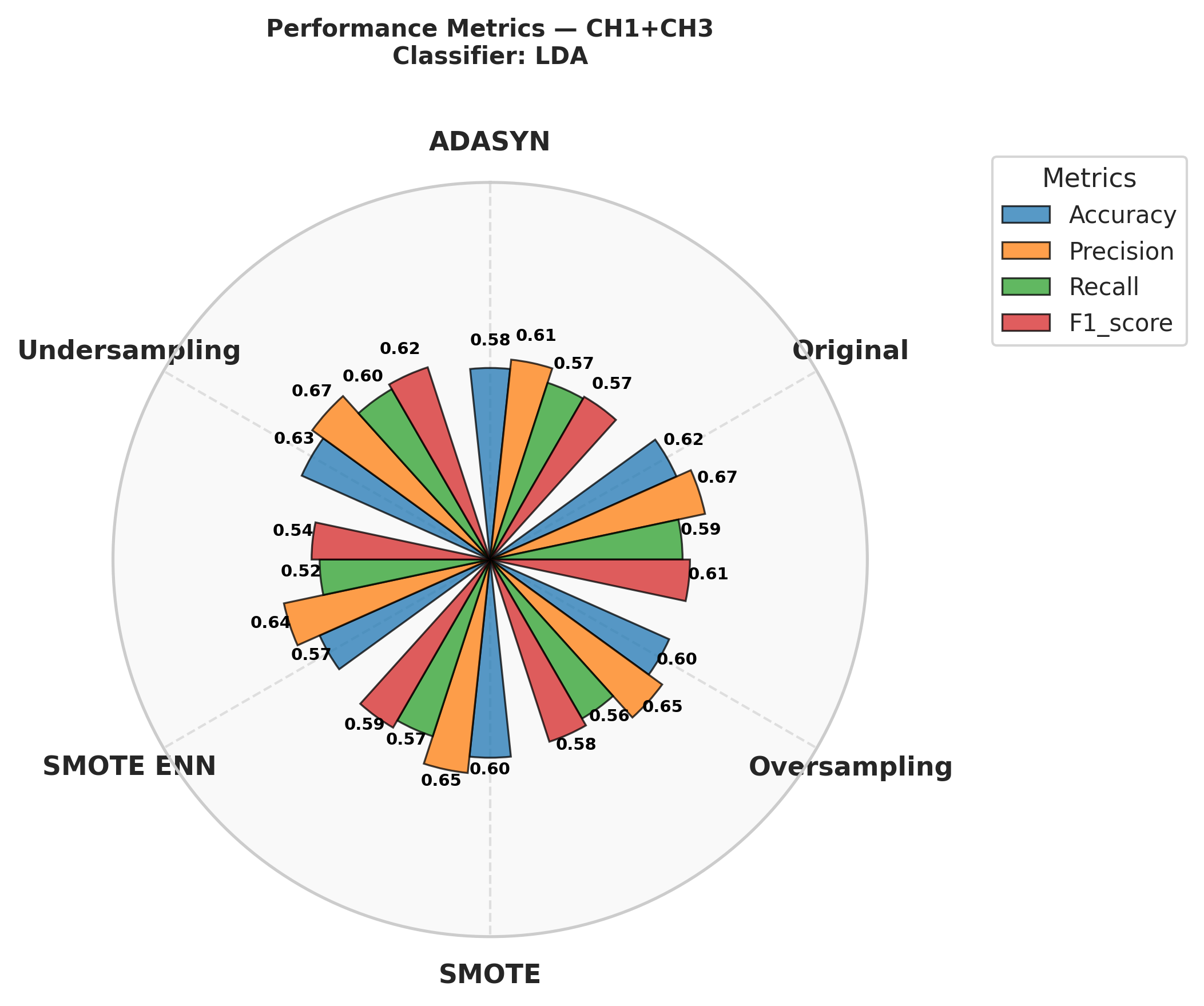

Supplement: Supplementary file 1 [file bioengineering-13-00787-s001.zip › Supplementary Material - Performance Metrics/CH1+CH3_LDA_polar.png]

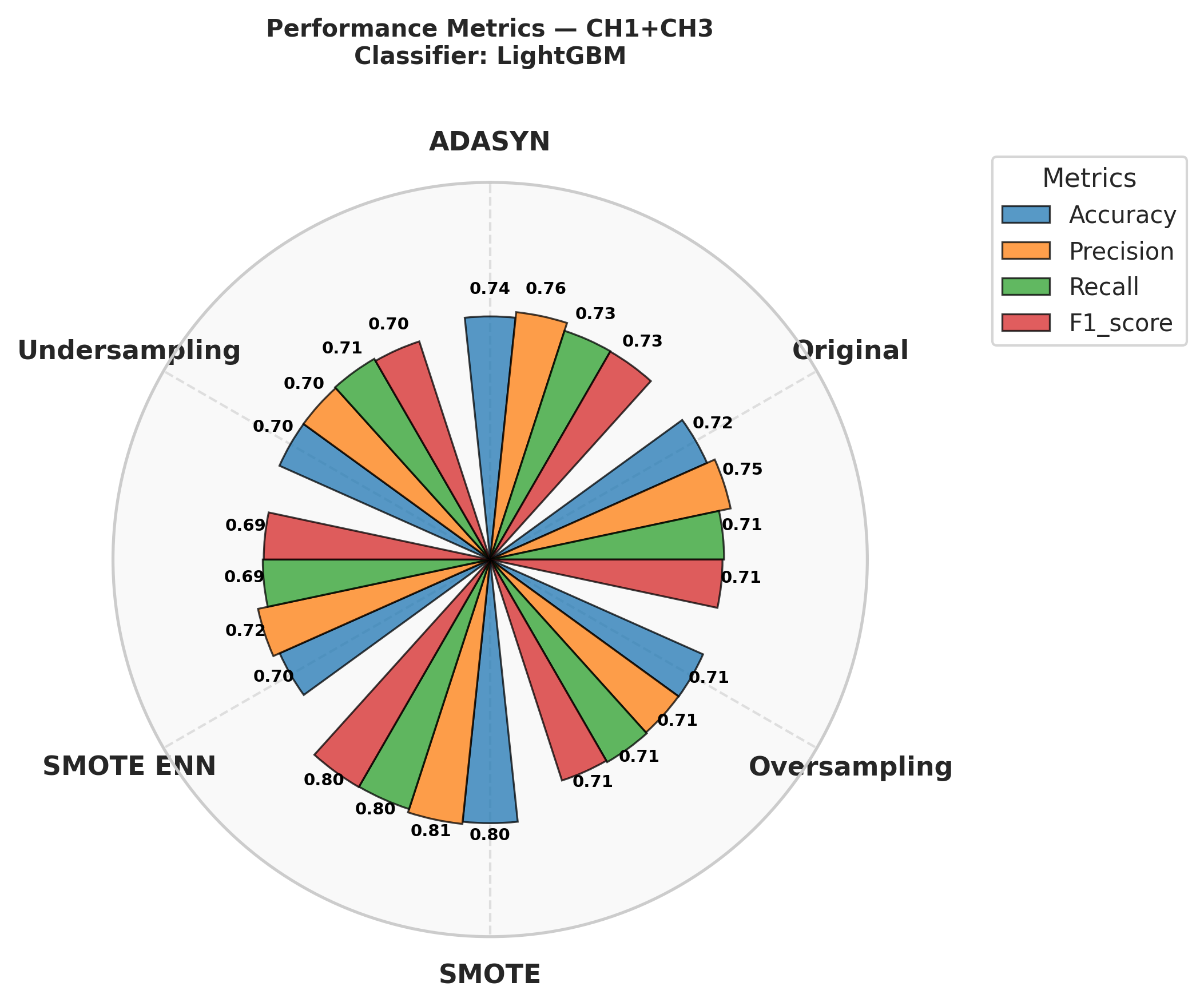

Supplement: Supplementary file 1 [file bioengineering-13-00787-s001.zip › Supplementary Material - Performance Metrics/CH1+CH3_LightGBM_polar.png]

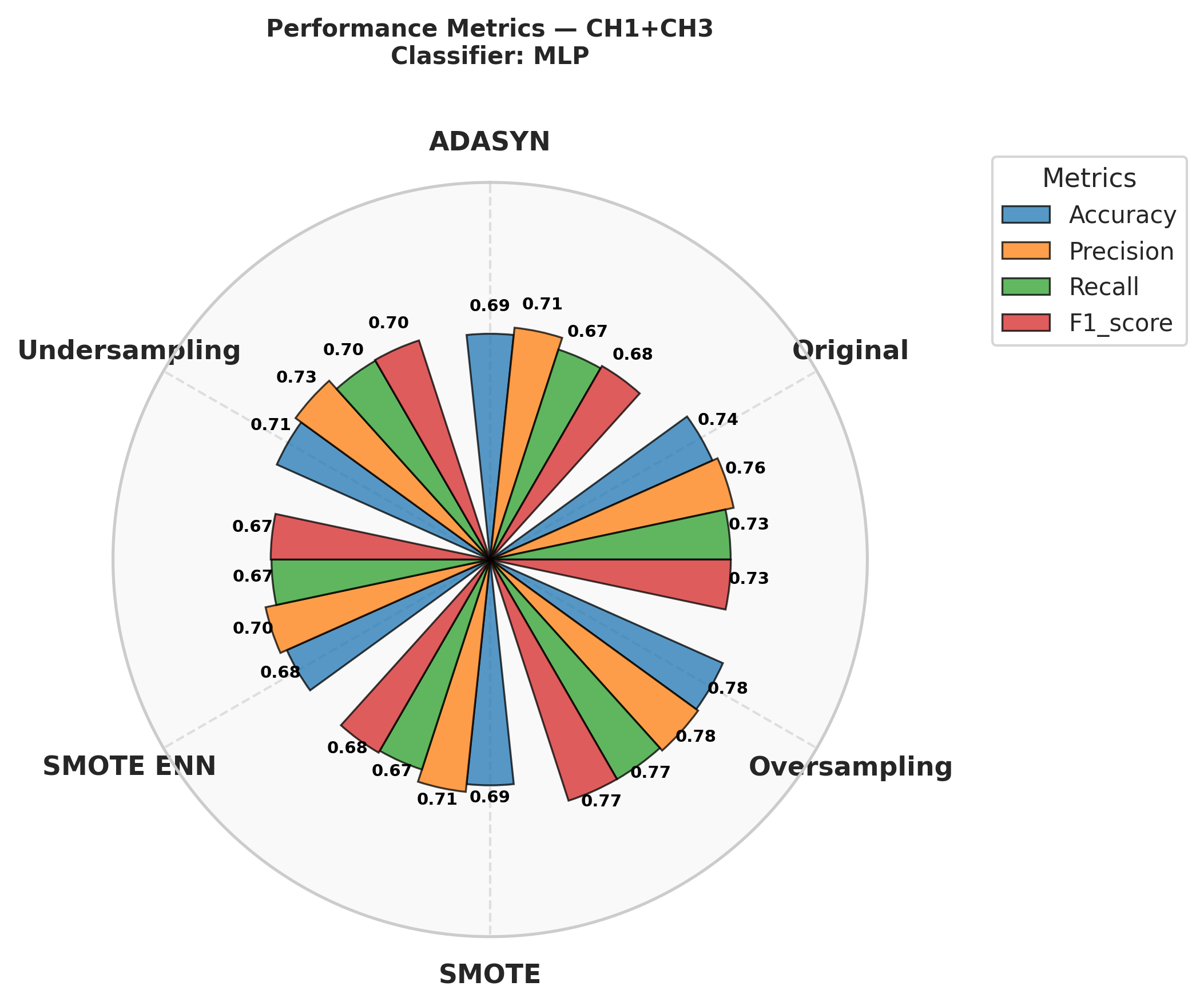

Supplement: Supplementary file 1 [file bioengineering-13-00787-s001.zip › Supplementary Material - Performance Metrics/CH1+CH3_MLP_polar.png]

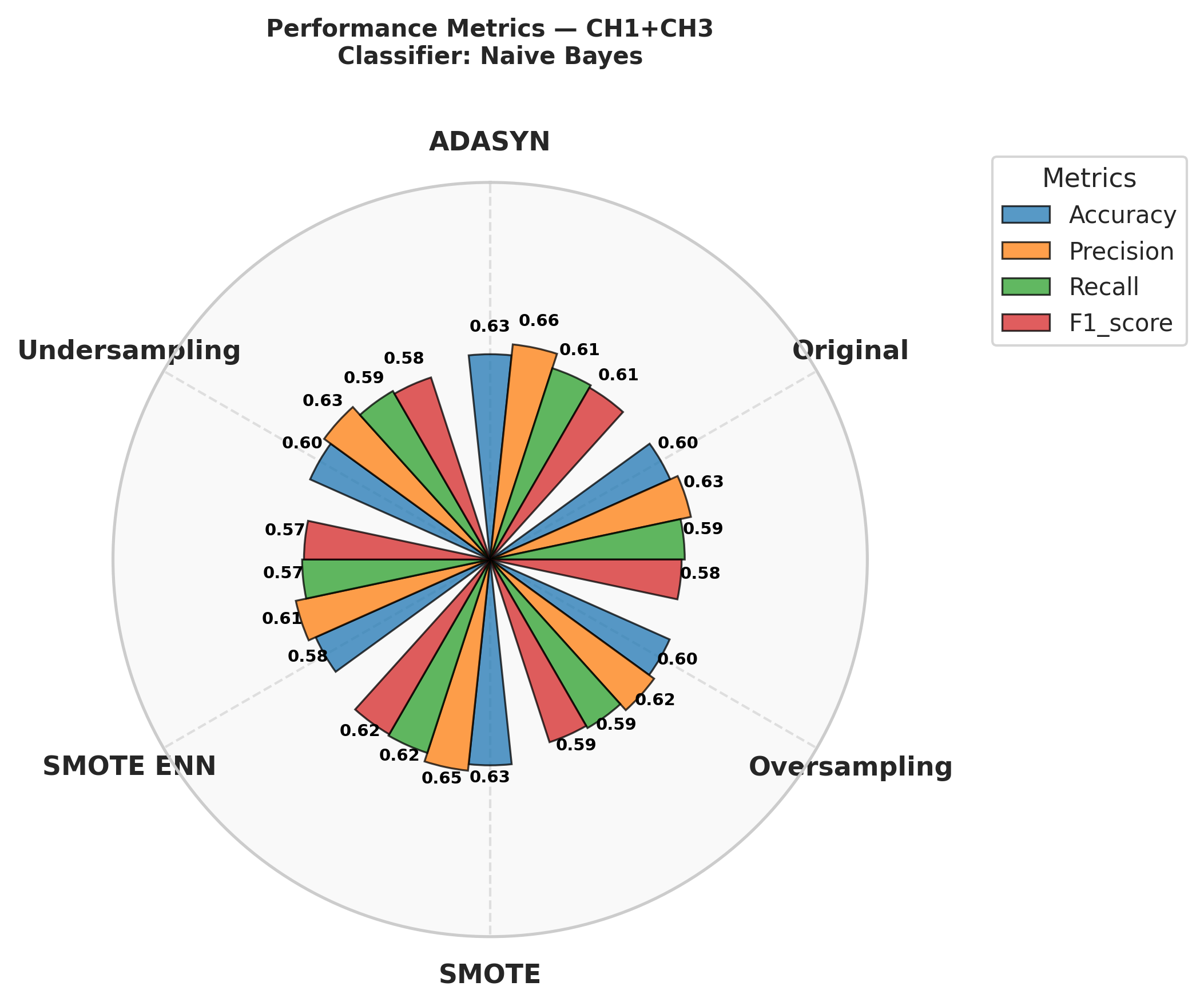

Supplement: Supplementary file 1 [file bioengineering-13-00787-s001.zip › Supplementary Material - Performance Metrics/CH1+CH3_Naive Bayes_polar.png]

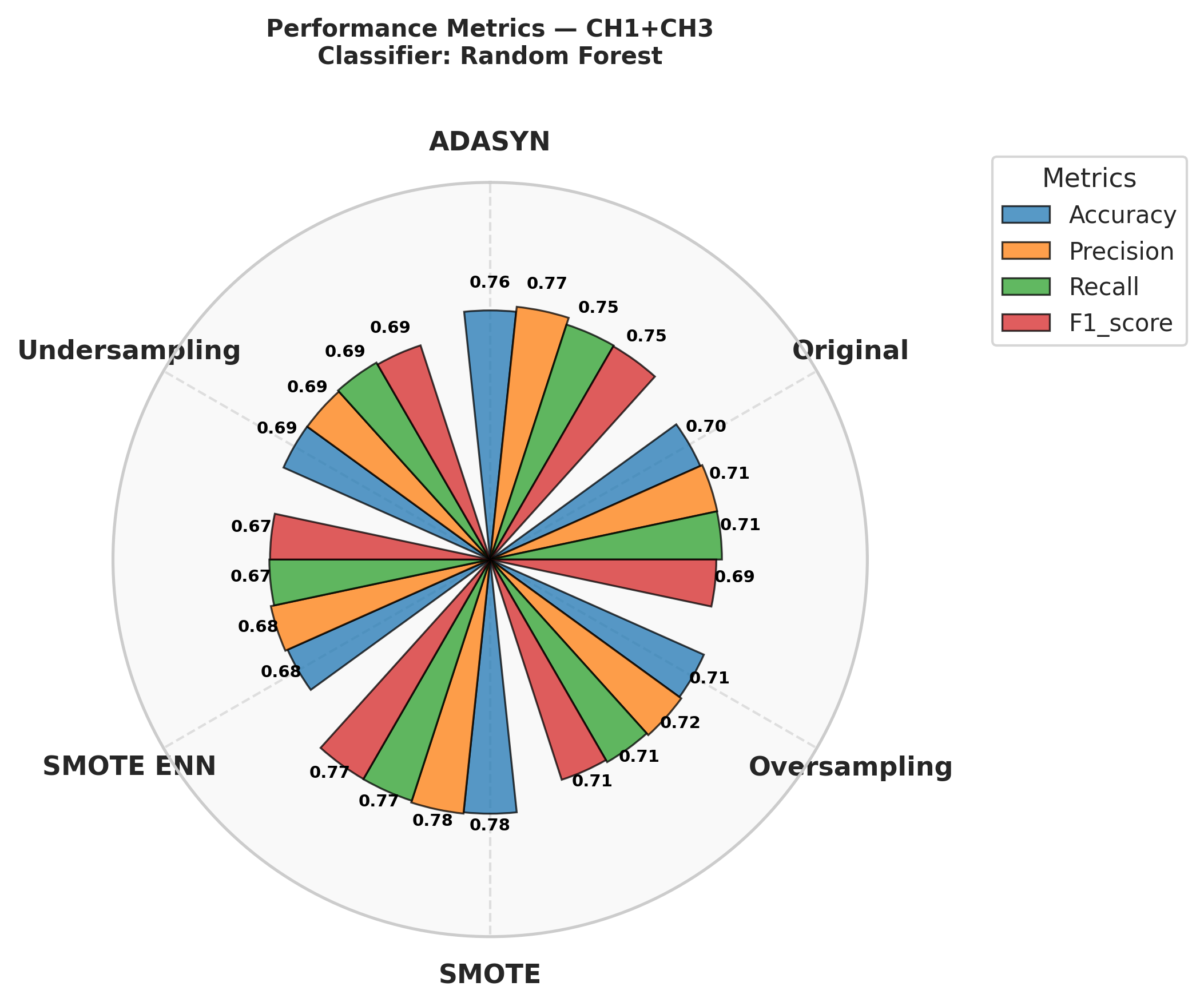

Supplement: Supplementary file 1 [file bioengineering-13-00787-s001.zip › Supplementary Material - Performance Metrics/CH1+CH3_Random Forest_polar.png]

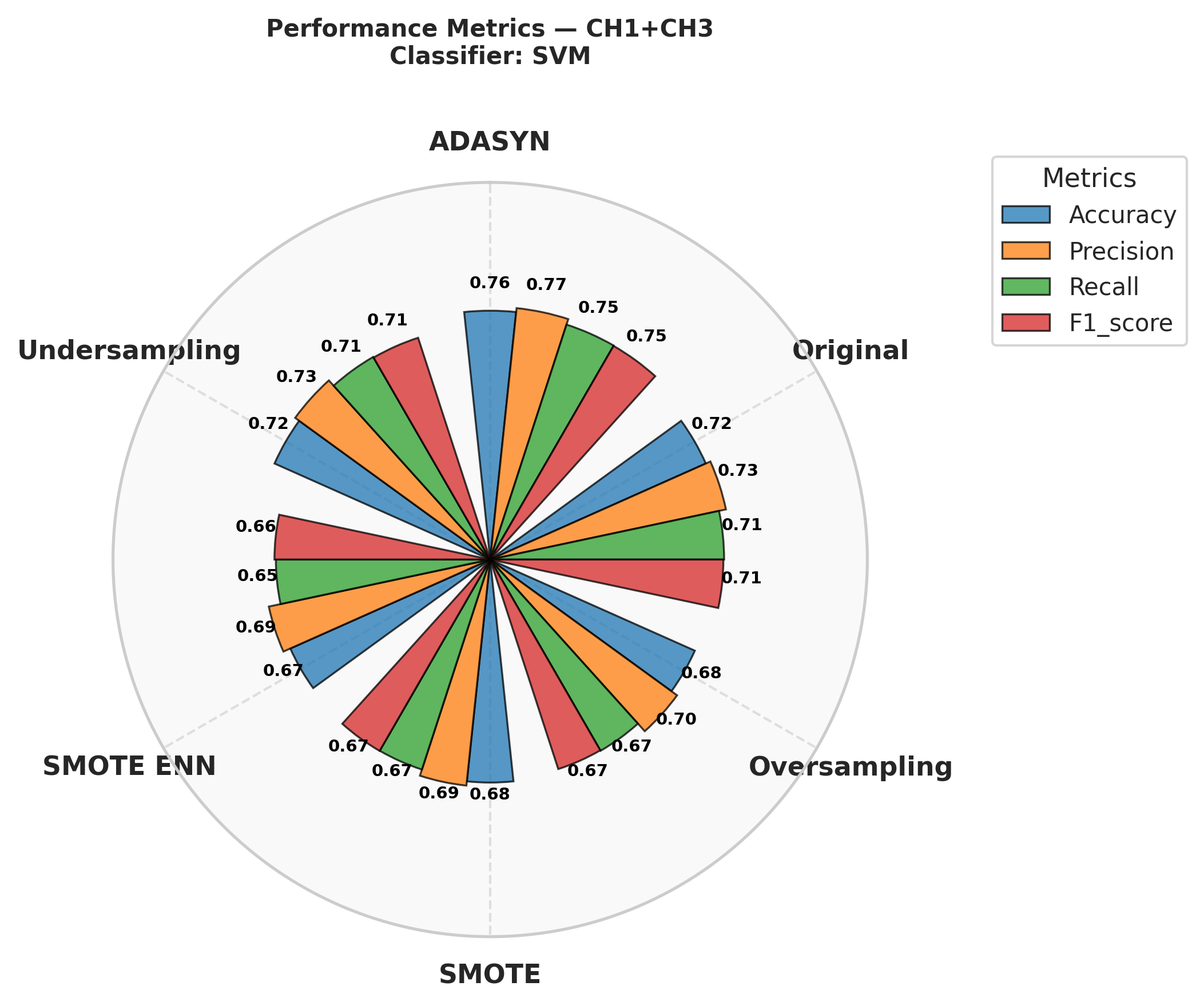

Supplement: Supplementary file 1 [file bioengineering-13-00787-s001.zip › Supplementary Material - Performance Metrics/CH1+CH3_SVM_polar.png]

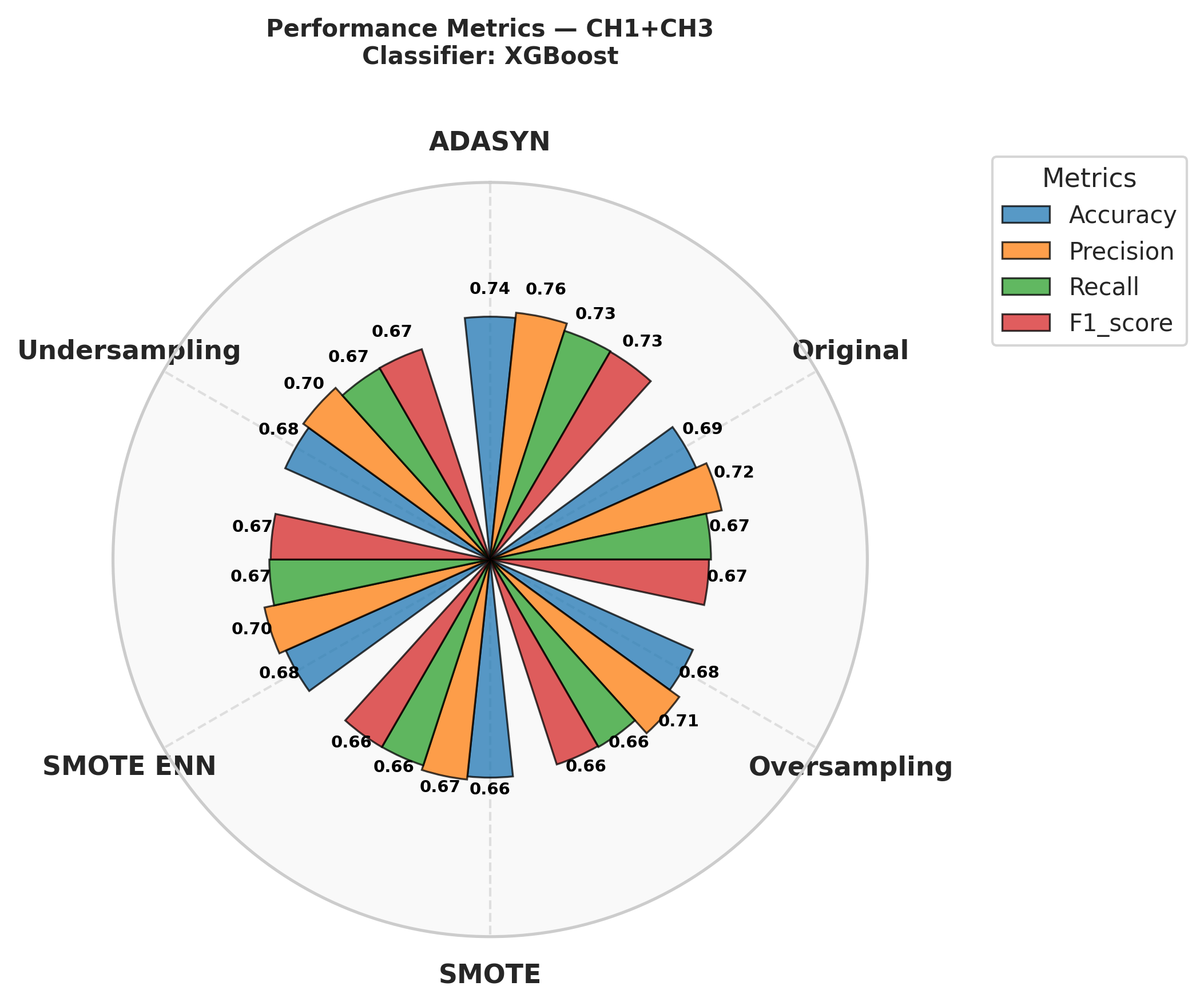

Supplement: Supplementary file 1 [file bioengineering-13-00787-s001.zip › Supplementary Material - Performance Metrics/CH1+CH3_XGBoost_polar.png]

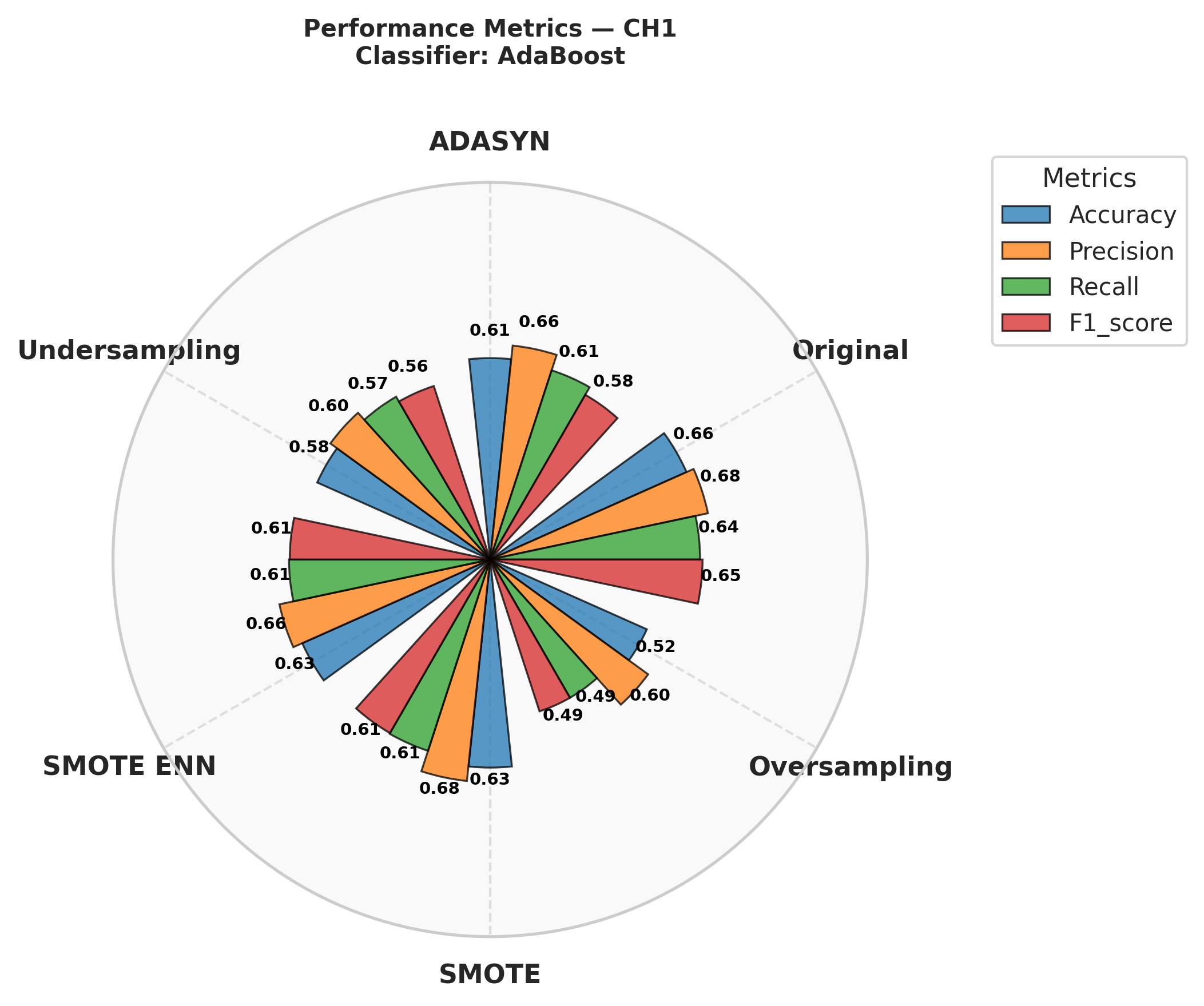

Supplement: Supplementary file 1 [file bioengineering-13-00787-s001.zip › Supplementary Material - Performance Metrics/CH1_AdaBoost_polar.png]

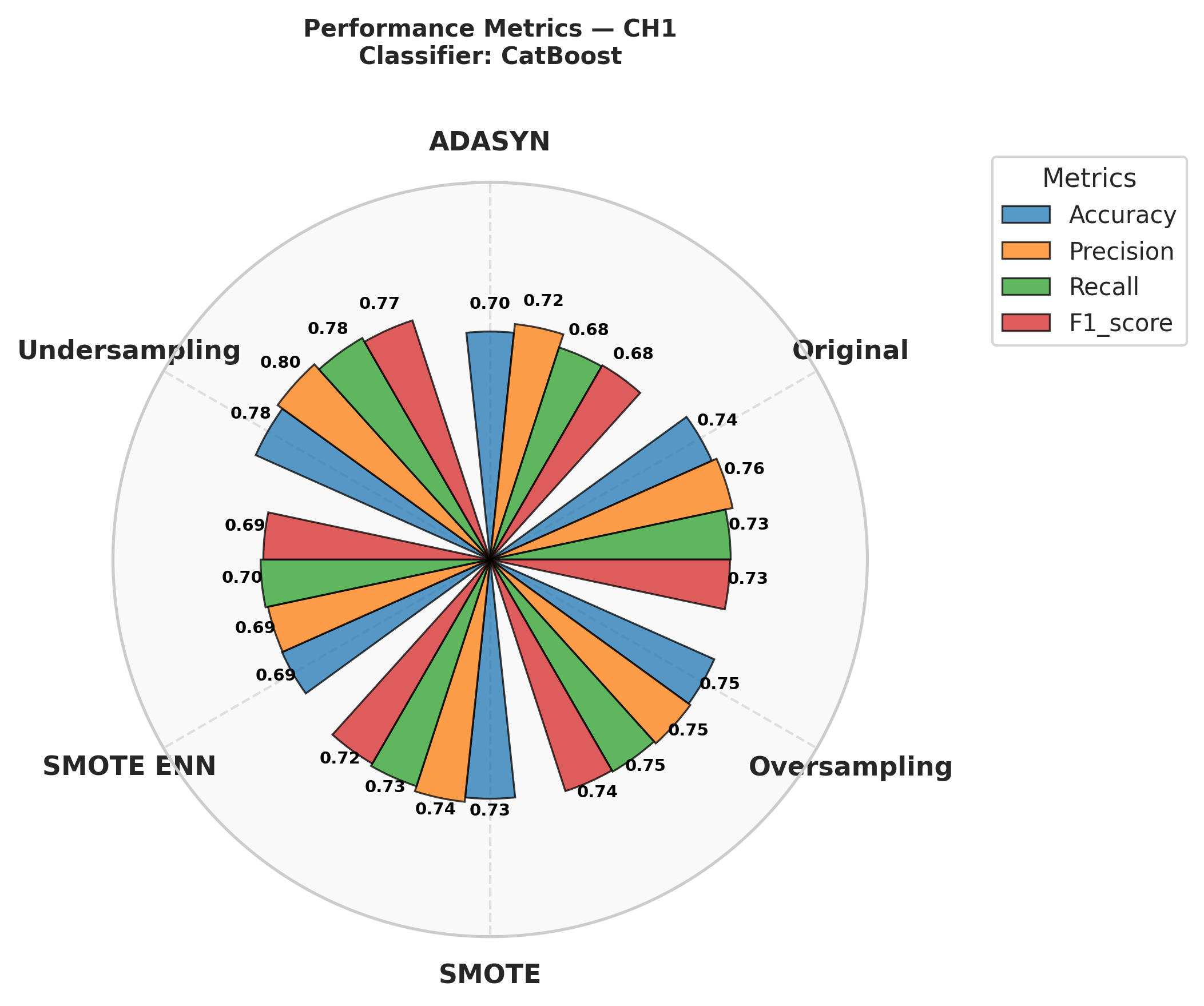

Supplement: Supplementary file 1 [file bioengineering-13-00787-s001.zip › Supplementary Material - Performance Metrics/CH1_CatBoost_polar.png]

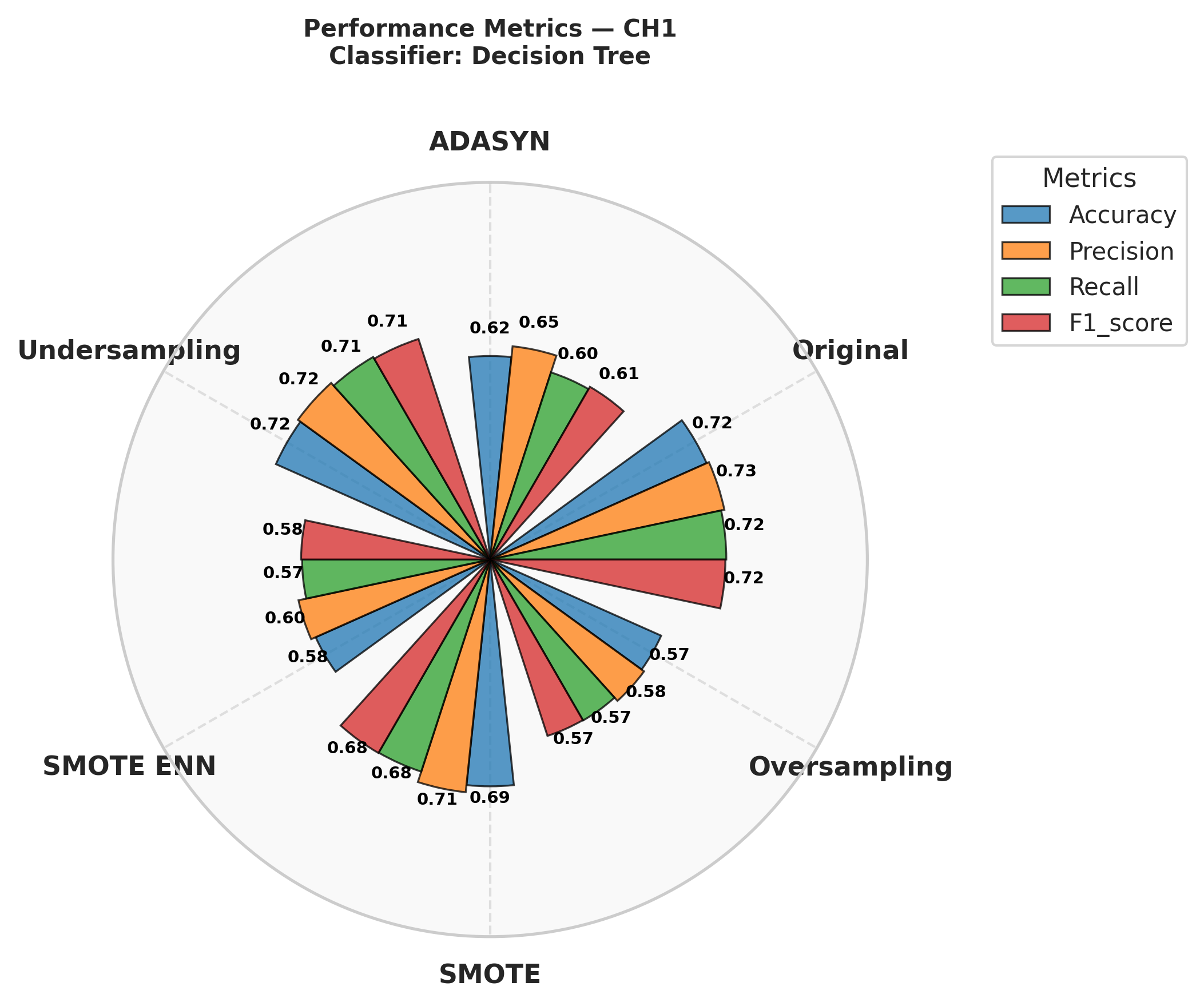

Supplement: Supplementary file 1 [file bioengineering-13-00787-s001.zip › Supplementary Material - Performance Metrics/CH1_Decision Tree_polar.png]

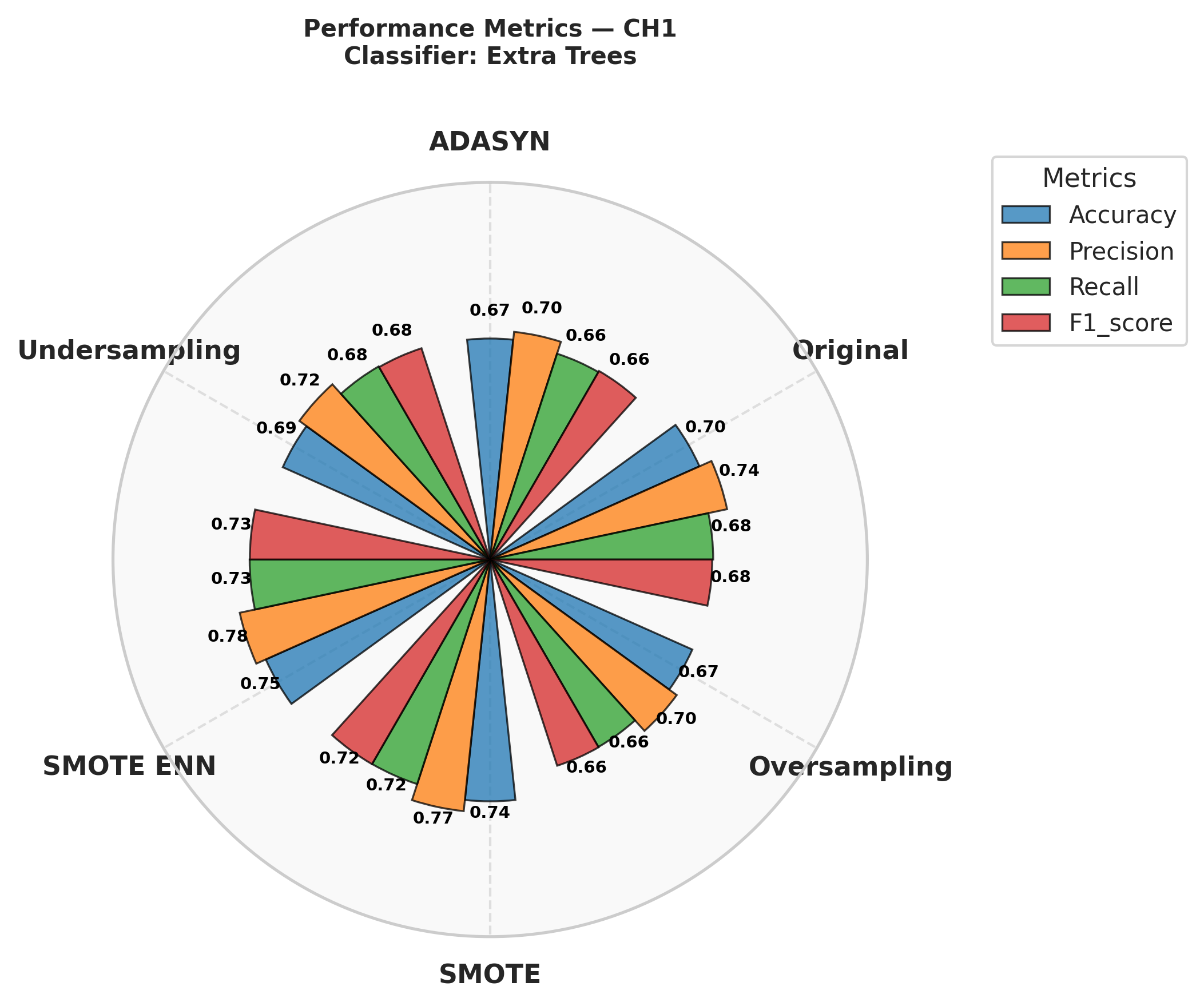

Supplement: Supplementary file 1 [file bioengineering-13-00787-s001.zip › Supplementary Material - Performance Metrics/CH1_Extra Trees_polar.png]

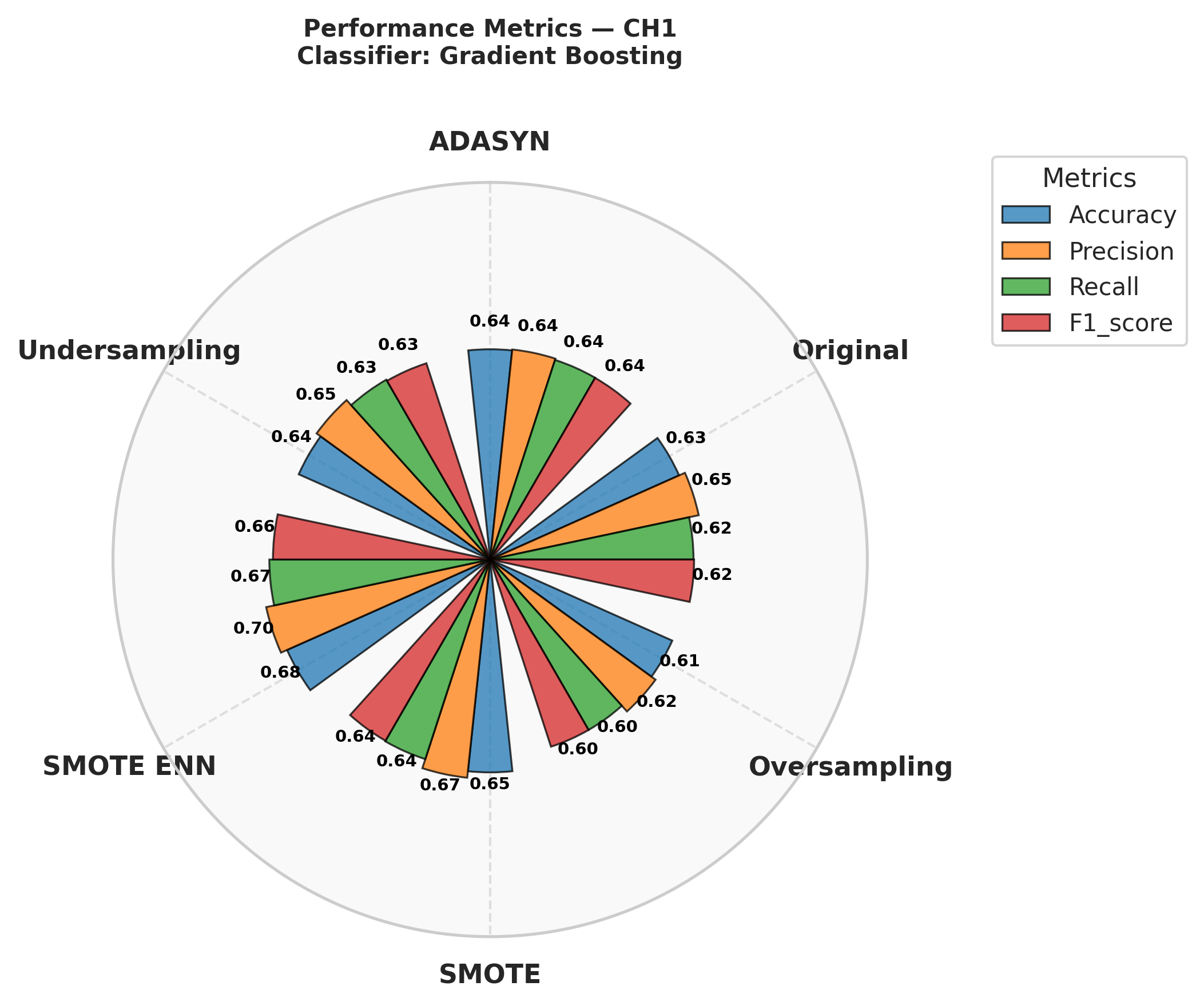

Supplement: Supplementary file 1 [file bioengineering-13-00787-s001.zip › Supplementary Material - Performance Metrics/CH1_Gradient Boosting_polar.png]

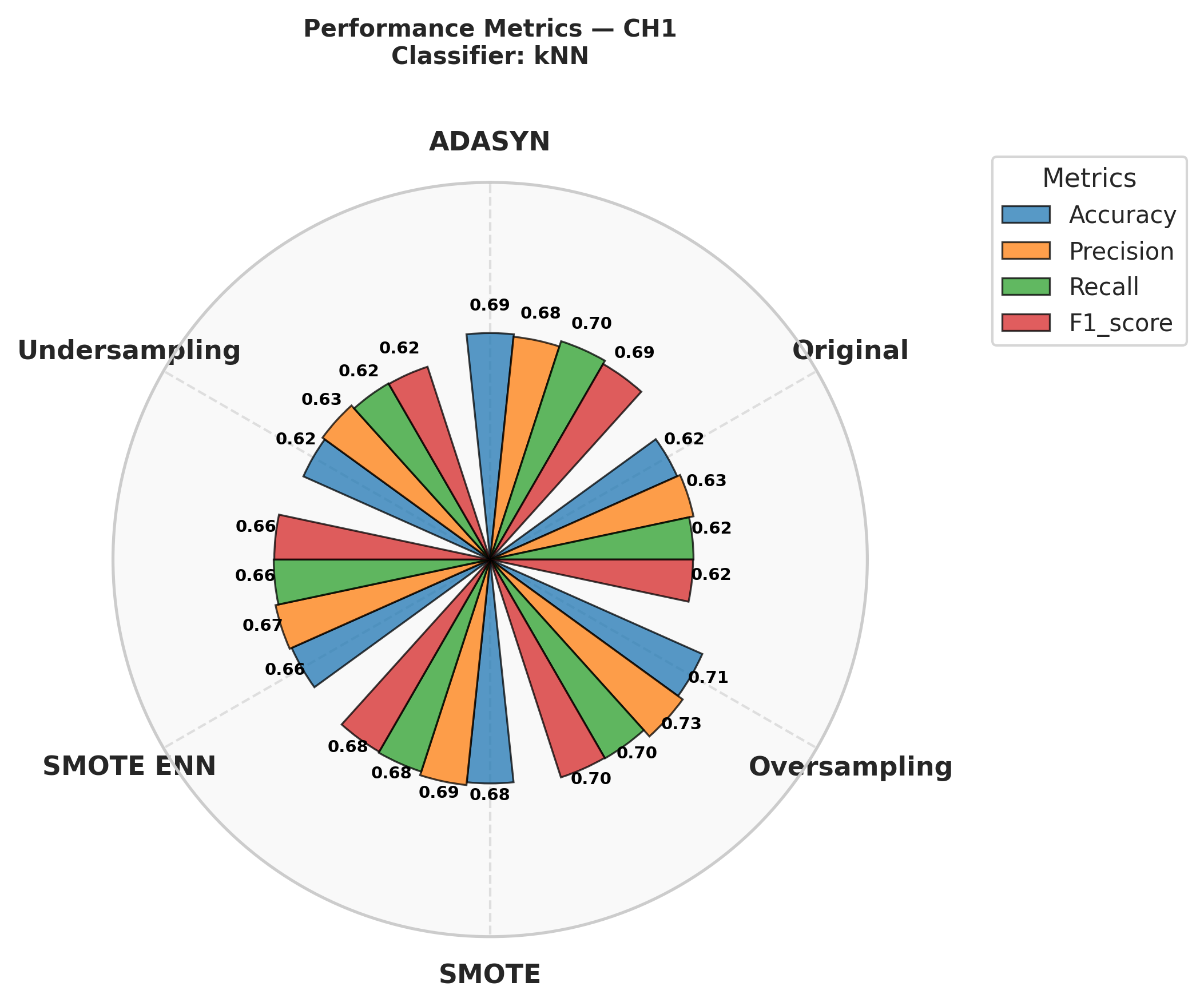

Supplement: Supplementary file 1 [file bioengineering-13-00787-s001.zip › Supplementary Material - Performance Metrics/CH1_kNN_polar.png]

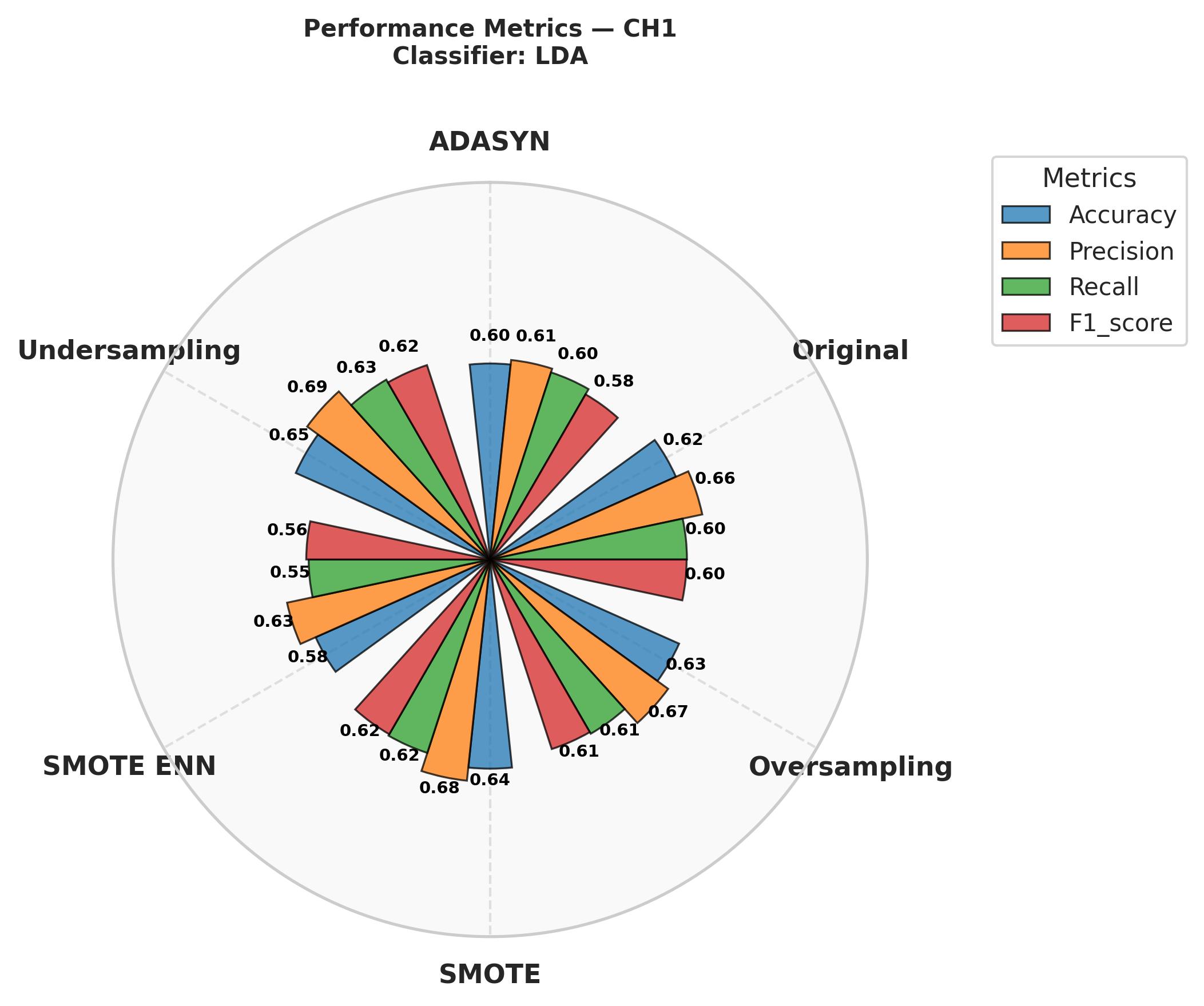

Supplement: Supplementary file 1 [file bioengineering-13-00787-s001.zip › Supplementary Material - Performance Metrics/CH1_LDA_polar.png]

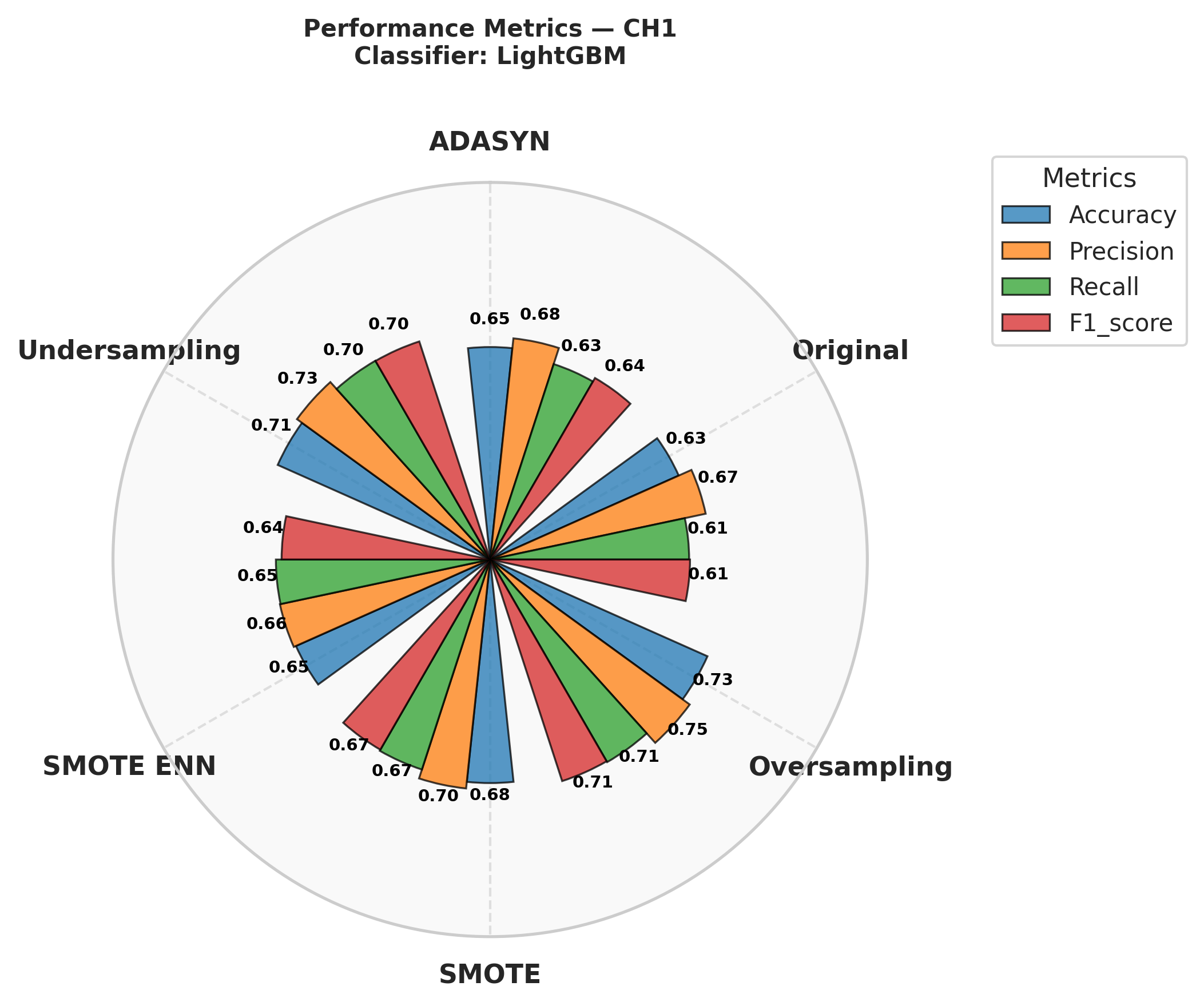

Supplement: Supplementary file 1 [file bioengineering-13-00787-s001.zip › Supplementary Material - Performance Metrics/CH1_LightGBM_polar.png]

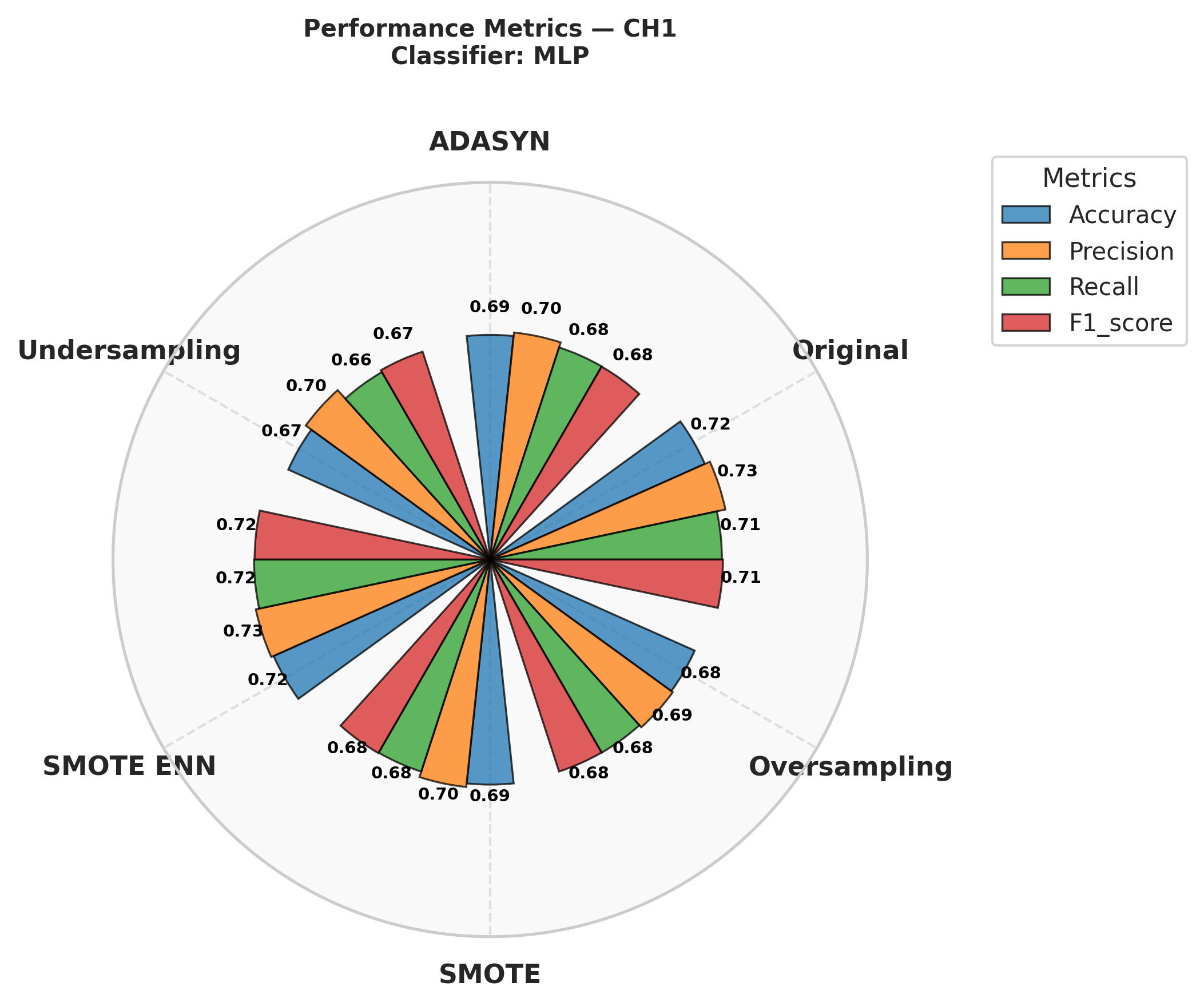

Supplement: Supplementary file 1 [file bioengineering-13-00787-s001.zip › Supplementary Material - Performance Metrics/CH1_MLP_polar.png]

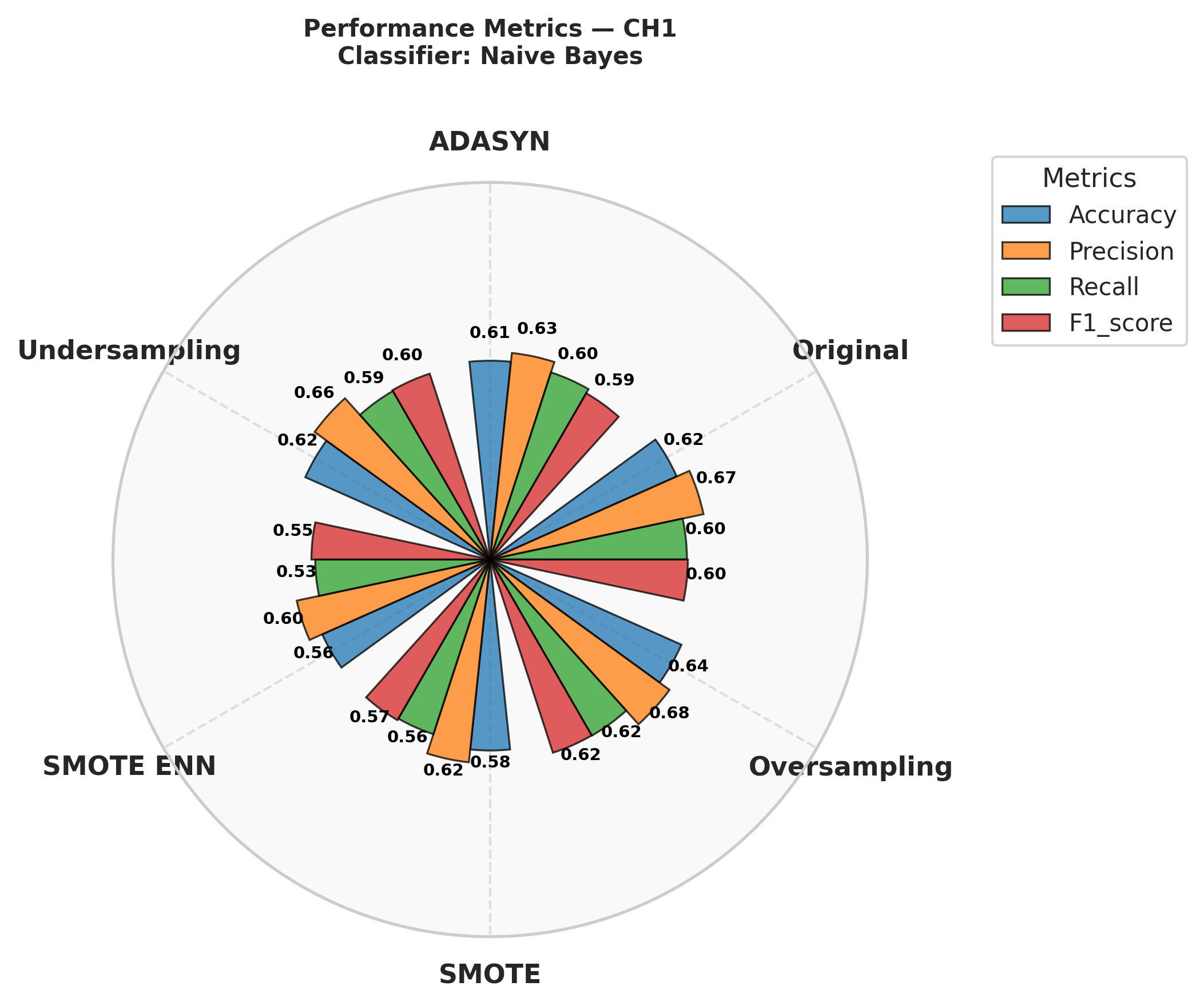

Supplement: Supplementary file 1 [file bioengineering-13-00787-s001.zip › Supplementary Material - Performance Metrics/CH1_Naive Bayes_polar.png]

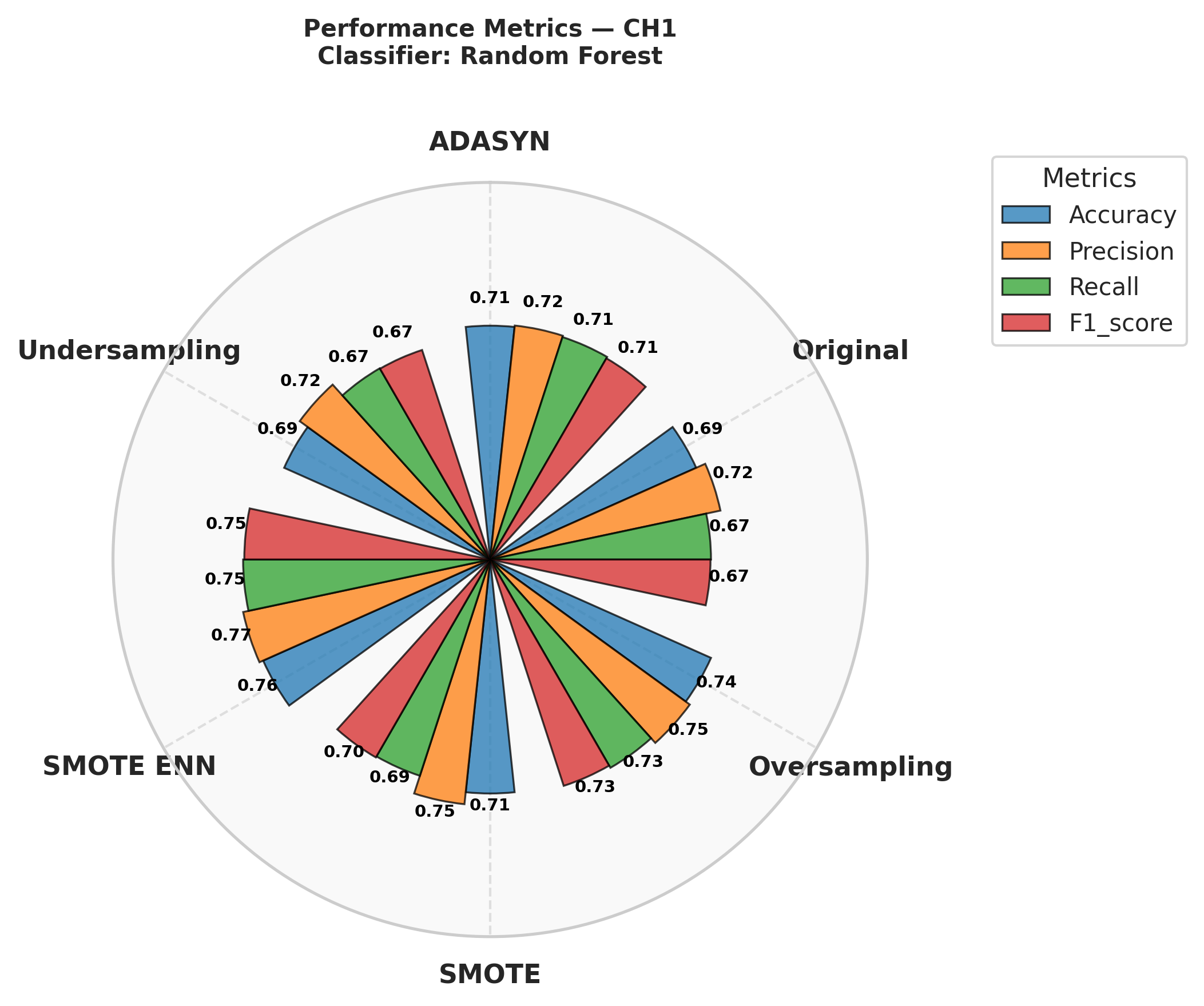

Supplement: Supplementary file 1 [file bioengineering-13-00787-s001.zip › Supplementary Material - Performance Metrics/CH1_Random Forest_polar.png]

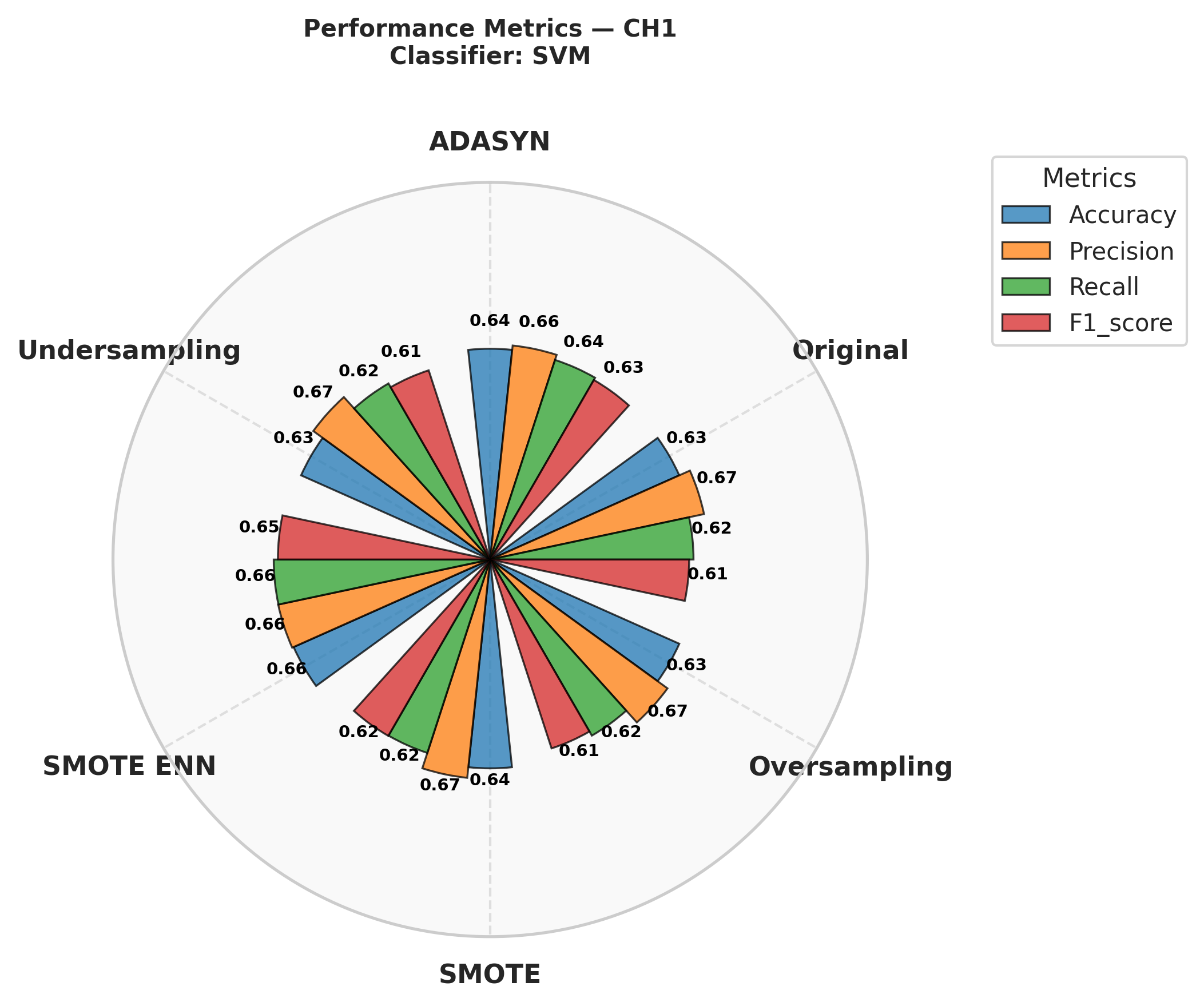

Supplement: Supplementary file 1 [file bioengineering-13-00787-s001.zip › Supplementary Material - Performance Metrics/CH1_SVM_polar.png]

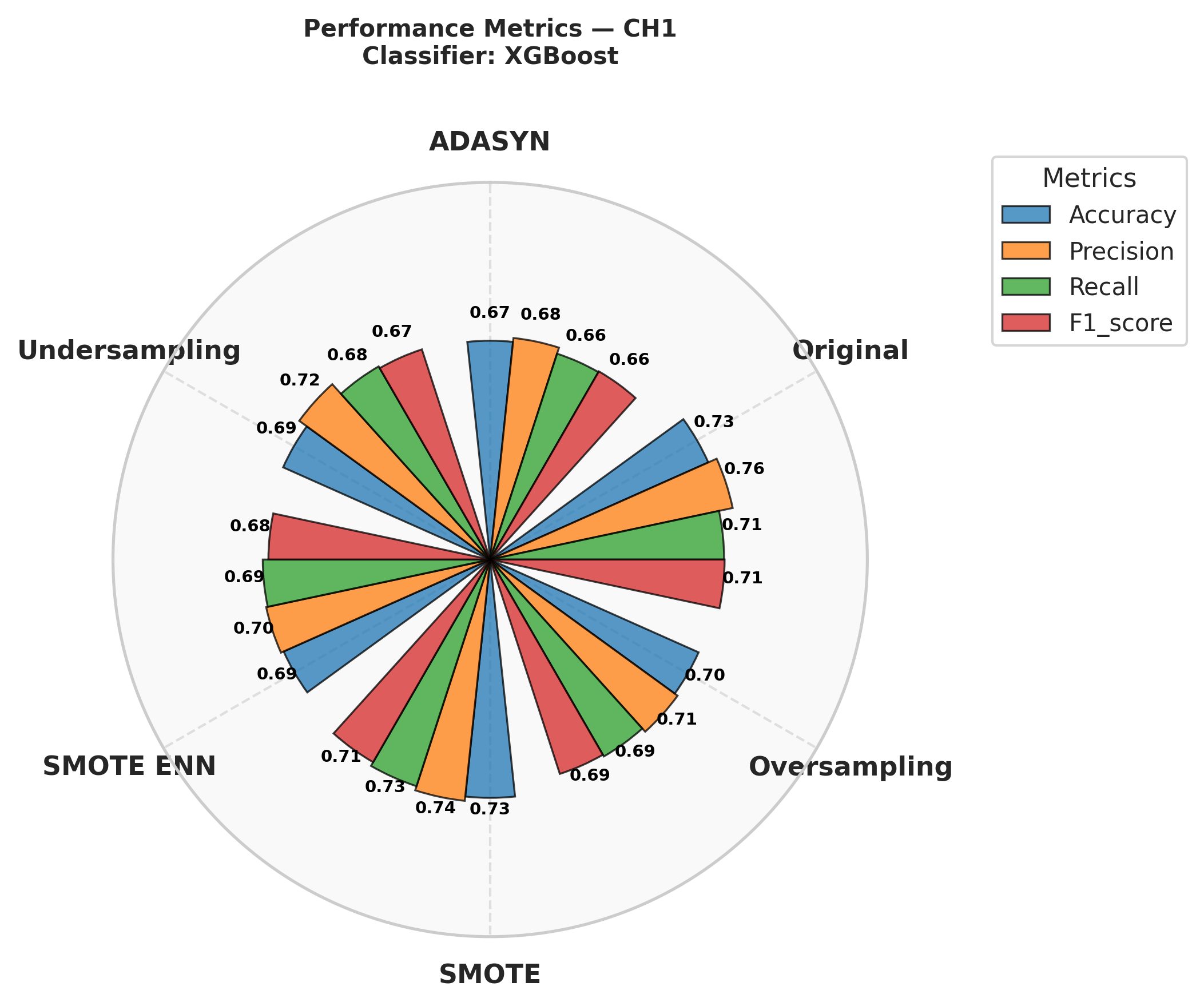

Supplement: Supplementary file 1 [file bioengineering-13-00787-s001.zip › Supplementary Material - Performance Metrics/CH1_XGBoost_polar.png]

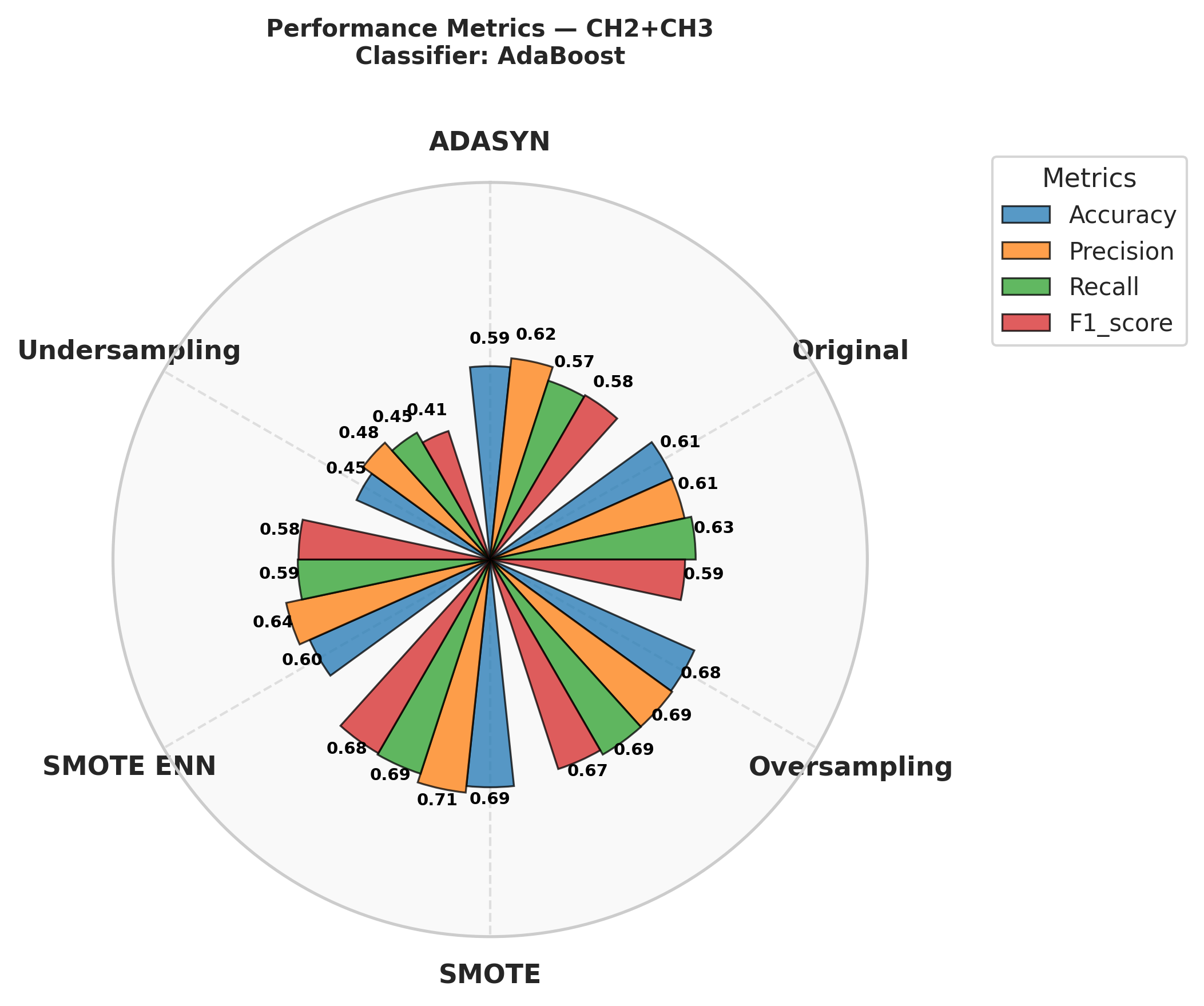

Supplement: Supplementary file 1 [file bioengineering-13-00787-s001.zip › Supplementary Material - Performance Metrics/CH2+CH3_AdaBoost_polar.png]

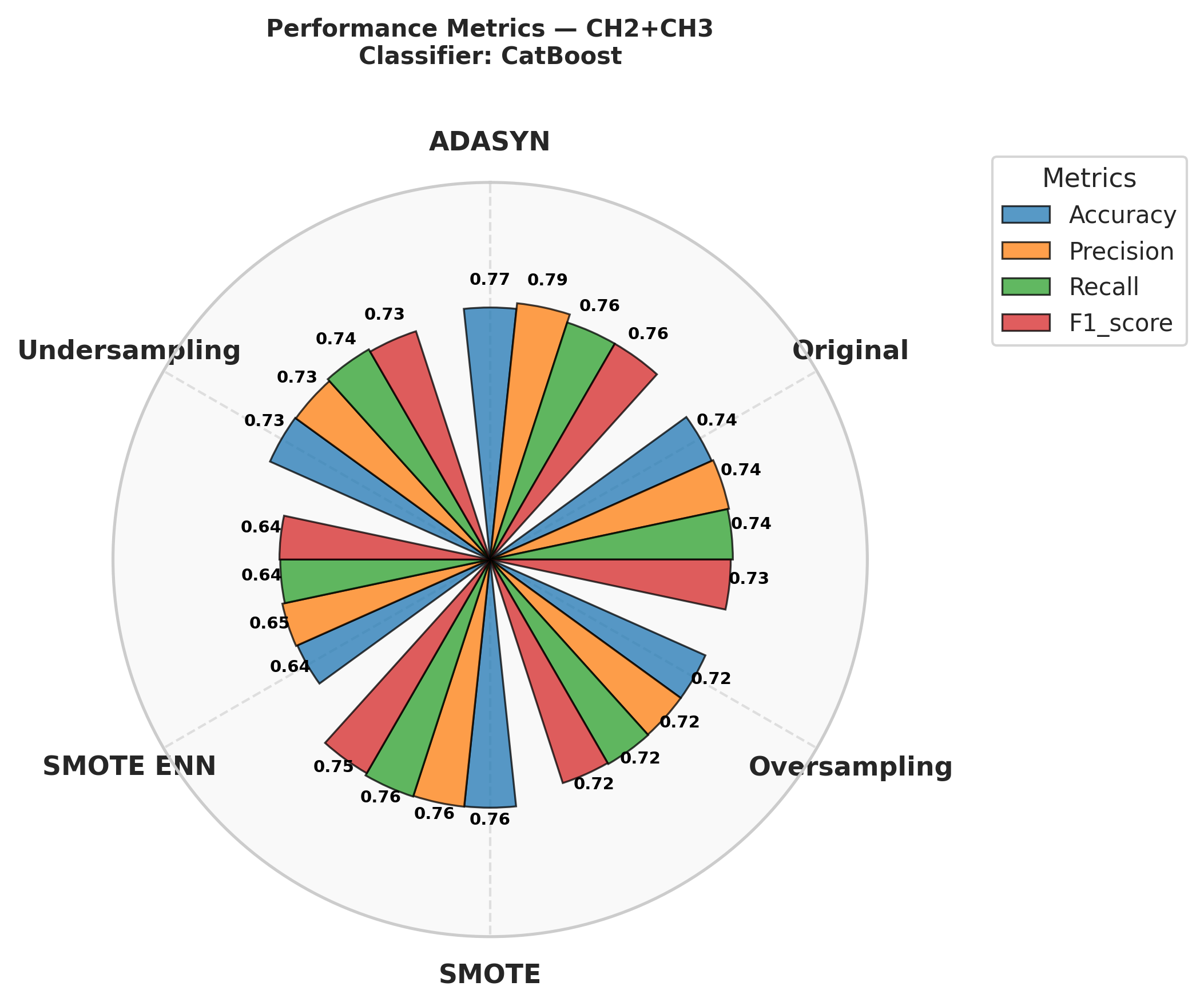

Supplement: Supplementary file 1 [file bioengineering-13-00787-s001.zip › Supplementary Material - Performance Metrics/CH2+CH3_CatBoost_polar.png]

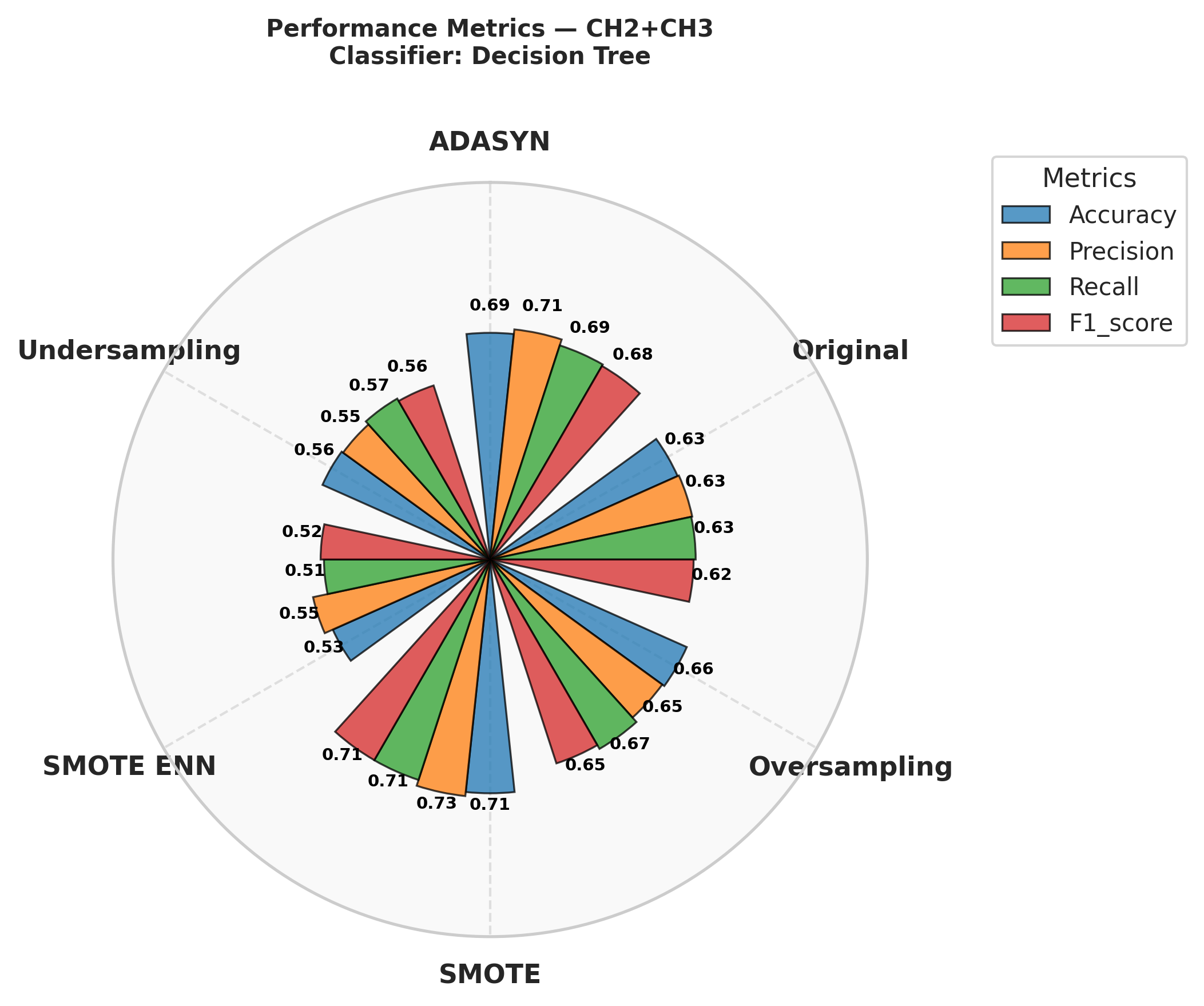

Supplement: Supplementary file 1 [file bioengineering-13-00787-s001.zip › Supplementary Material - Performance Metrics/CH2+CH3_Decision Tree_polar.png]

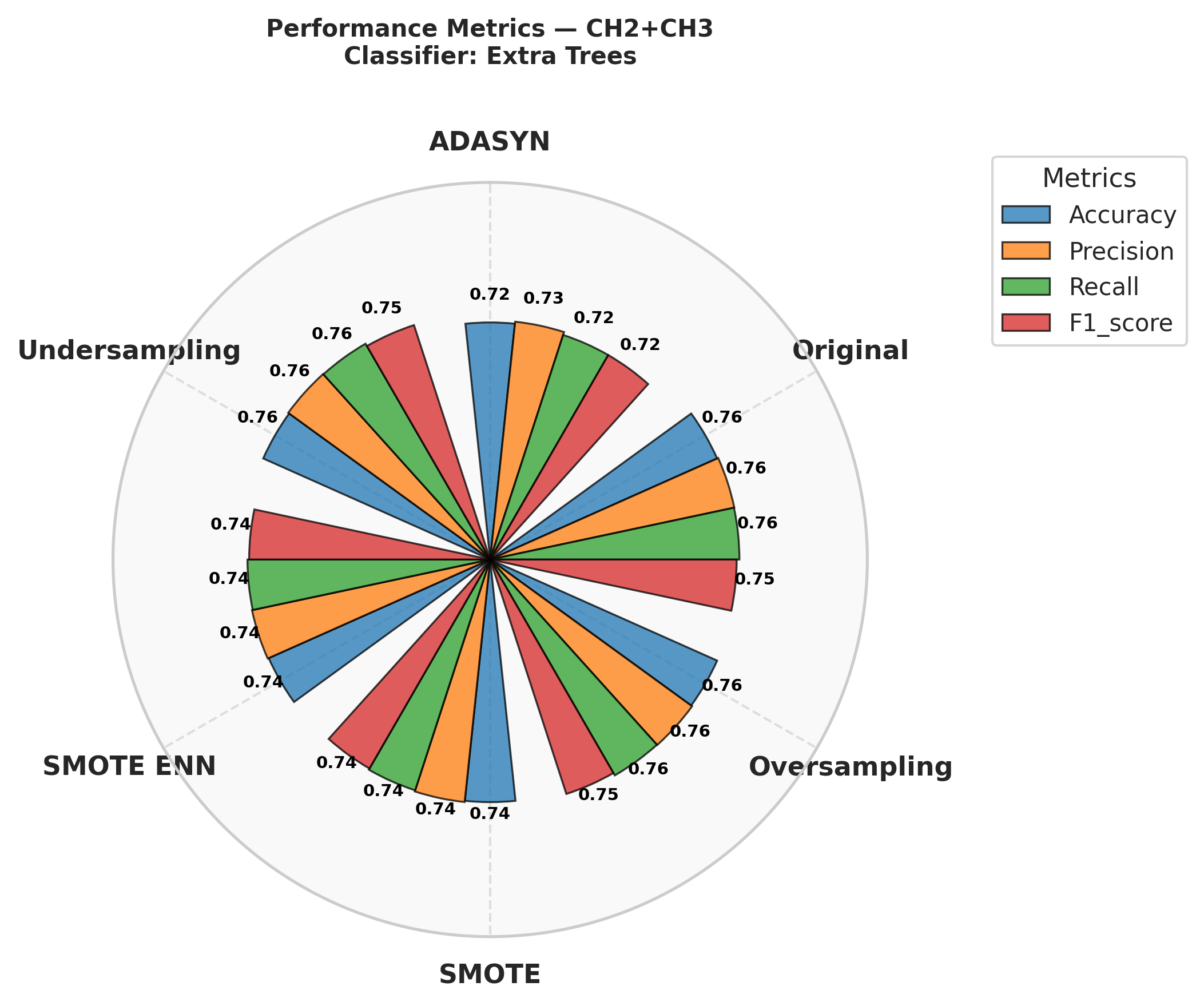

Supplement: Supplementary file 1 [file bioengineering-13-00787-s001.zip › Supplementary Material - Performance Metrics/CH2+CH3_Extra Trees_polar.png]

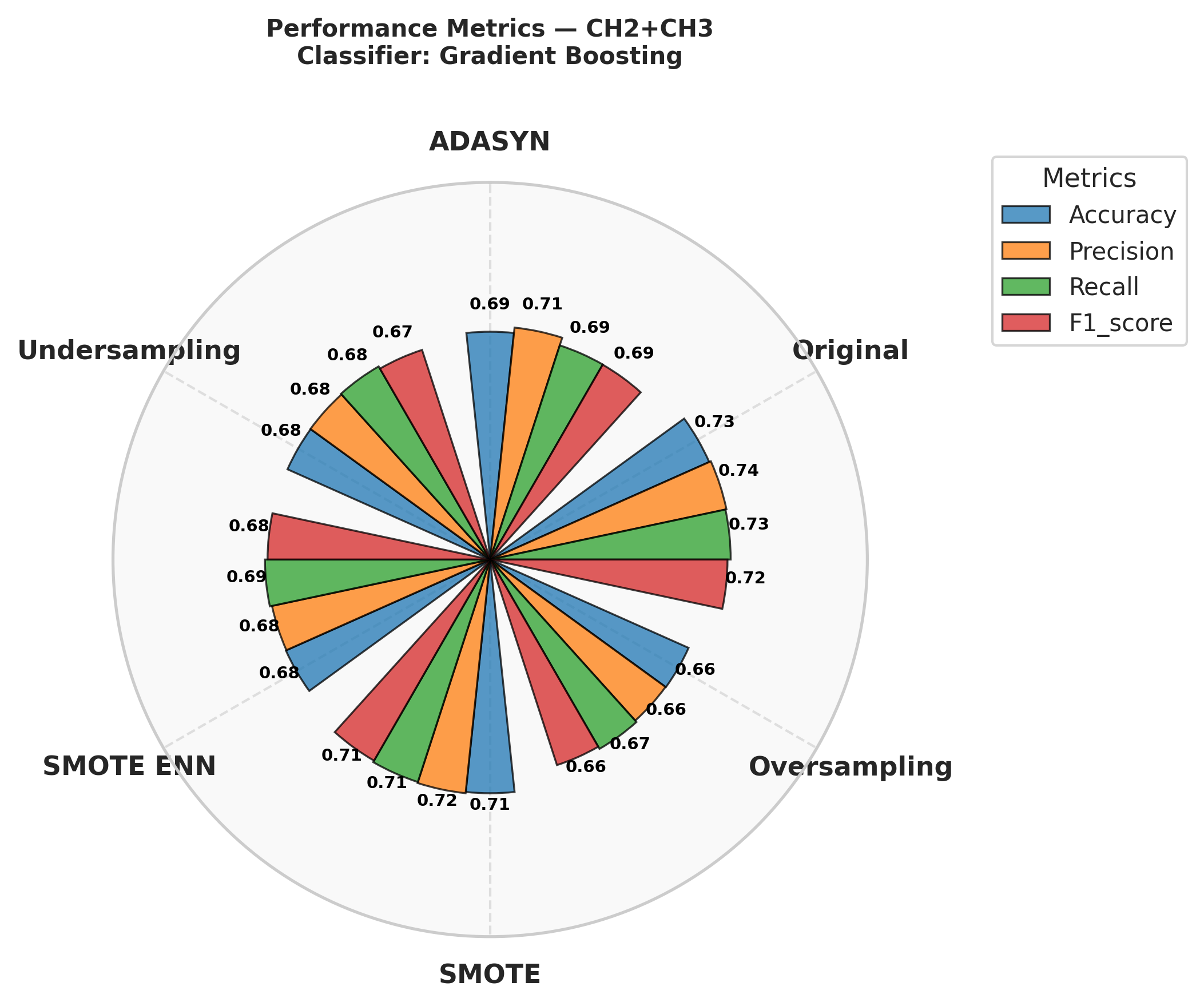

Supplement: Supplementary file 1 [file bioengineering-13-00787-s001.zip › Supplementary Material - Performance Metrics/CH2+CH3_Gradient Boosting_polar.png]

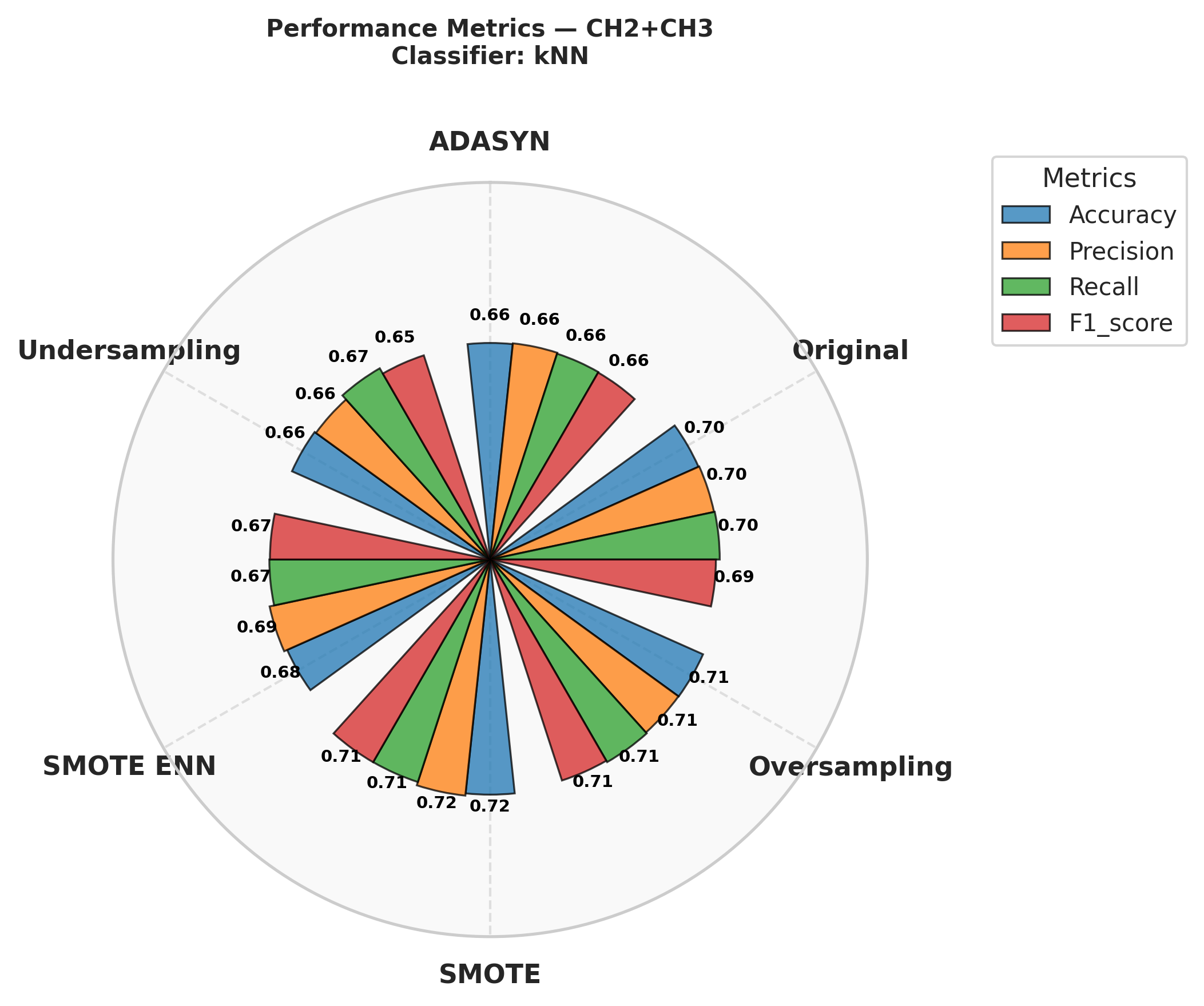

Supplement: Supplementary file 1 [file bioengineering-13-00787-s001.zip › Supplementary Material - Performance Metrics/CH2+CH3_kNN_polar.png]

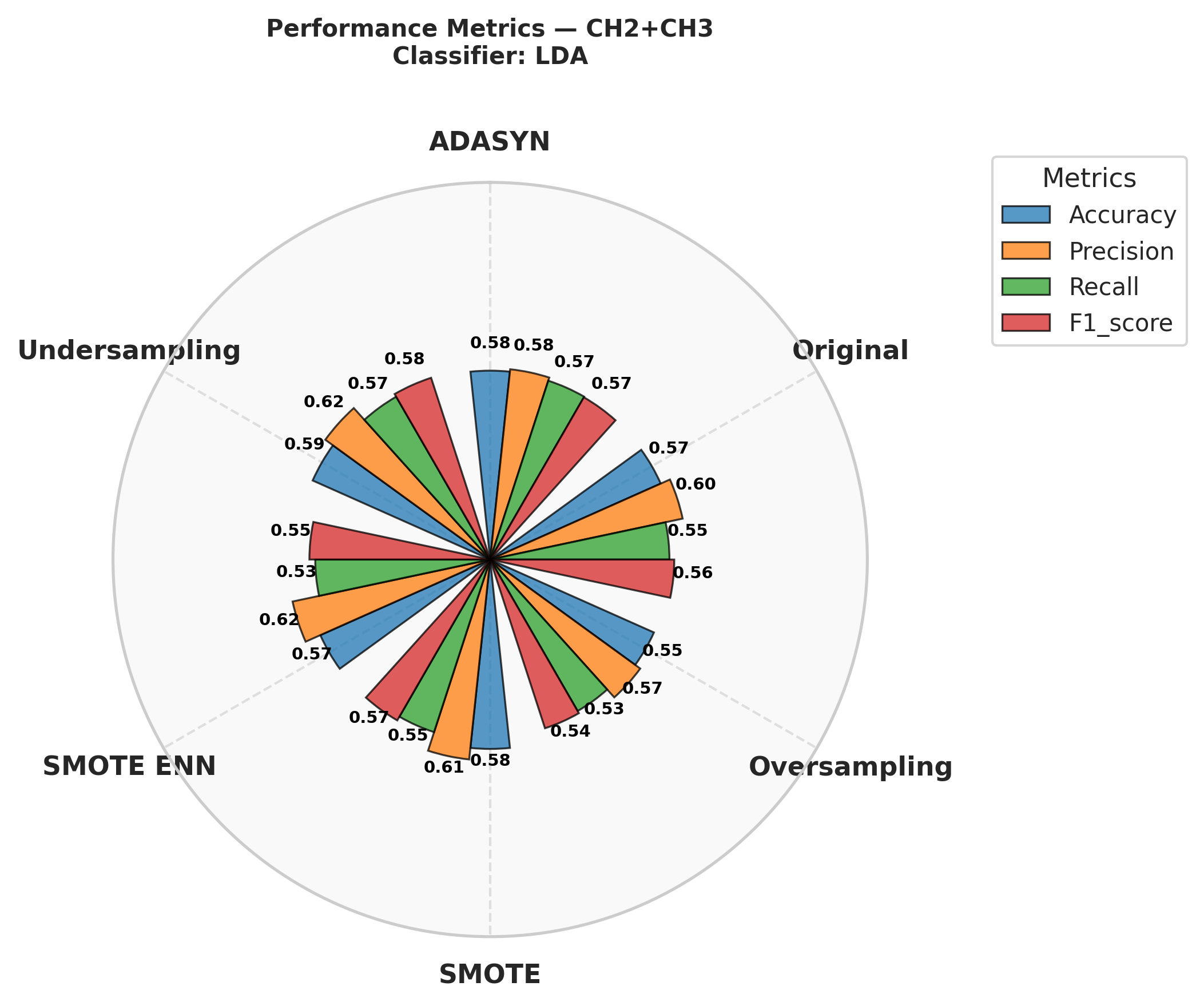

Supplement: Supplementary file 1 [file bioengineering-13-00787-s001.zip › Supplementary Material - Performance Metrics/CH2+CH3_LDA_polar.png]

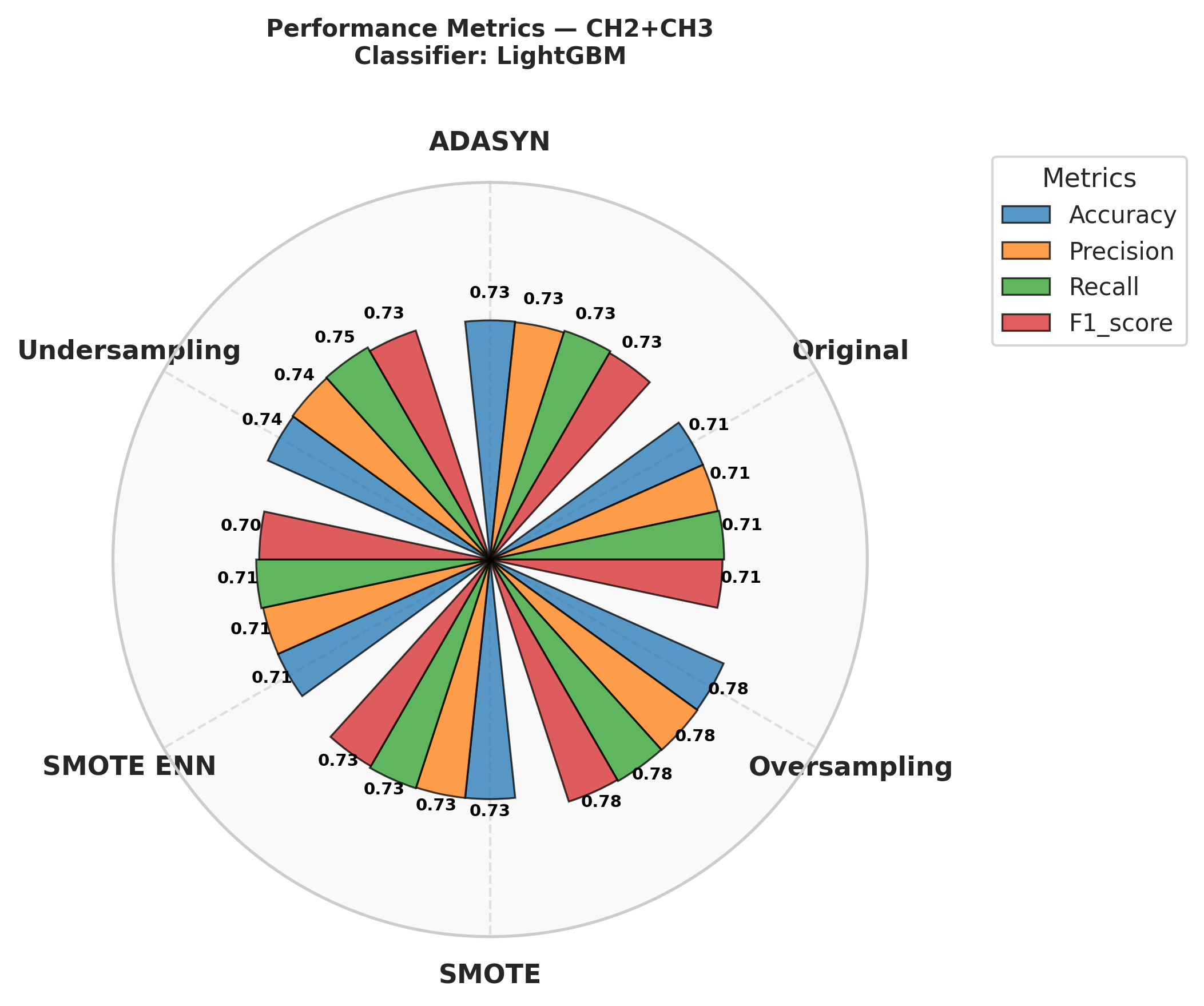

Supplement: Supplementary file 1 [file bioengineering-13-00787-s001.zip › Supplementary Material - Performance Metrics/CH2+CH3_LightGBM_polar.png]

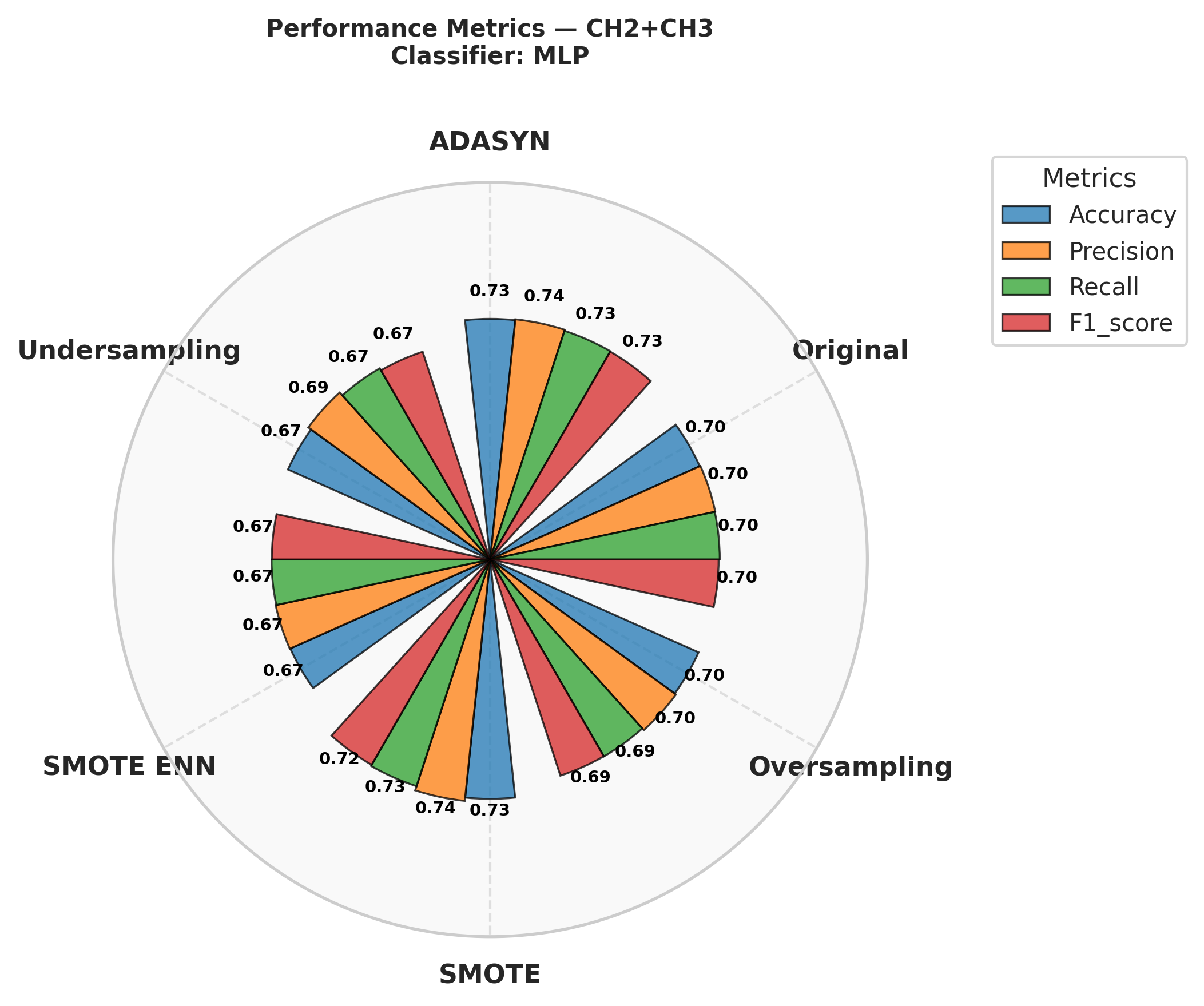

Supplement: Supplementary file 1 [file bioengineering-13-00787-s001.zip › Supplementary Material - Performance Metrics/CH2+CH3_MLP_polar.png]

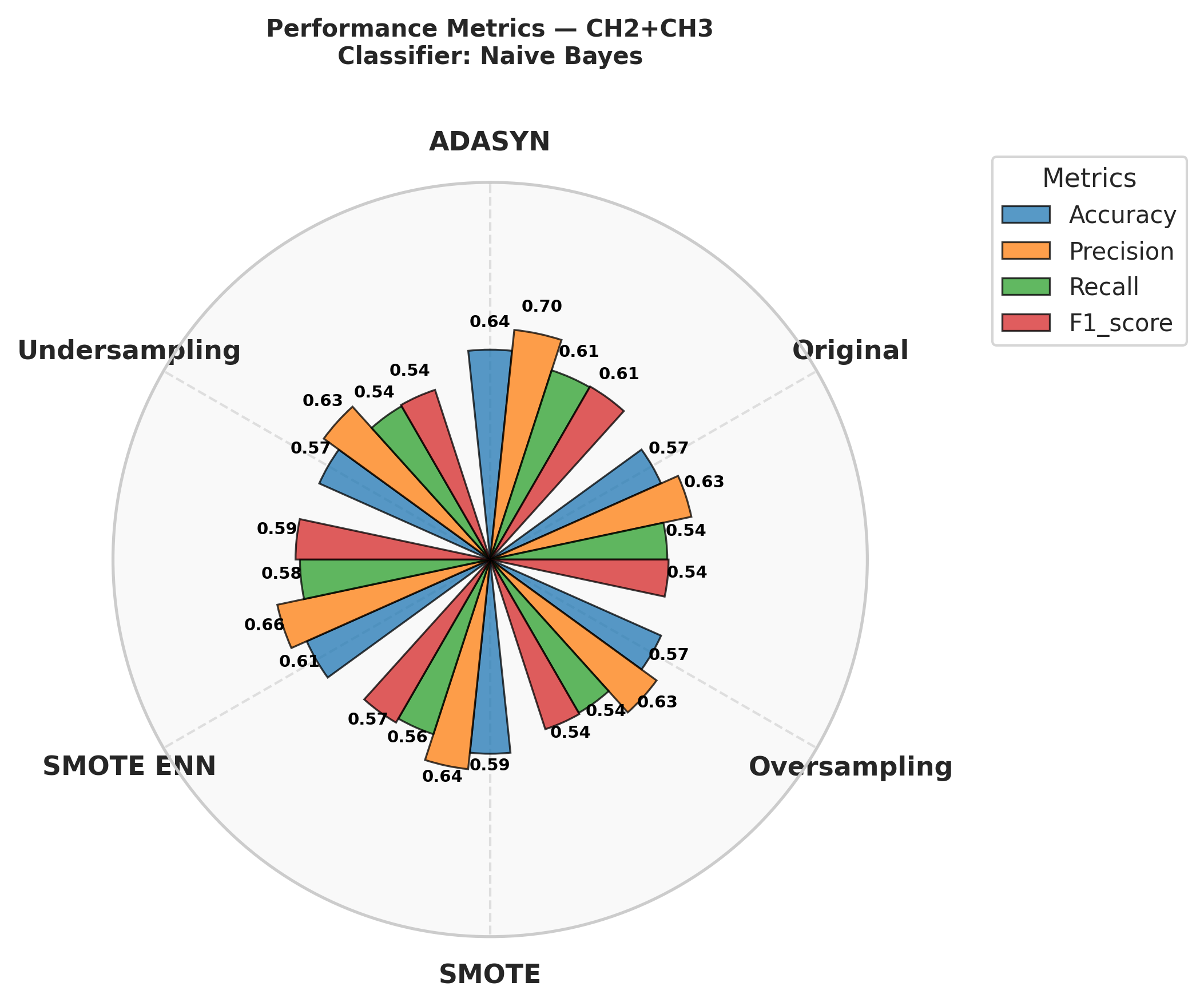

Supplement: Supplementary file 1 [file bioengineering-13-00787-s001.zip › Supplementary Material - Performance Metrics/CH2+CH3_Naive Bayes_polar.png]

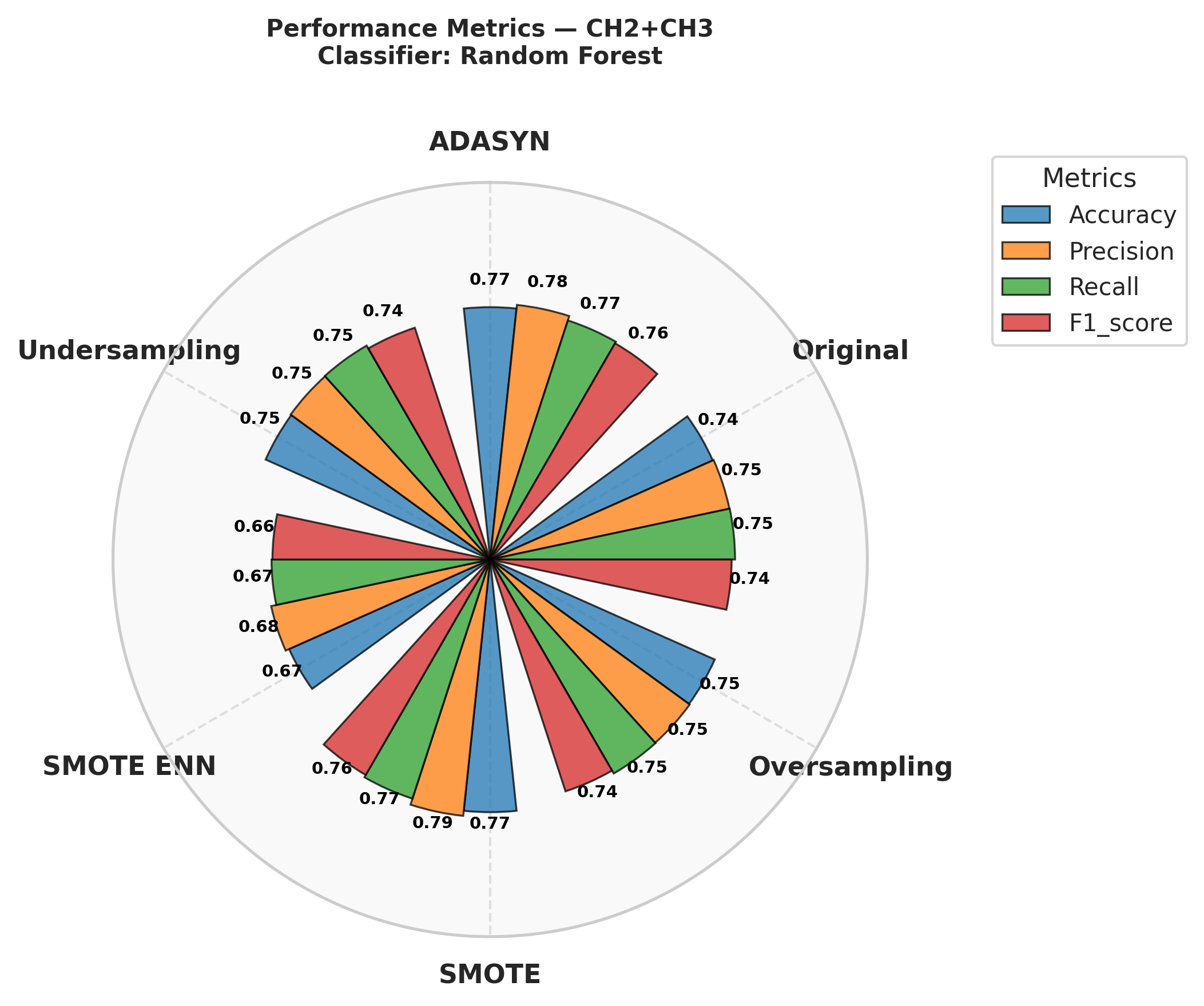

Supplement: Supplementary file 1 [file bioengineering-13-00787-s001.zip › Supplementary Material - Performance Metrics/CH2+CH3_Random Forest_polar.png]

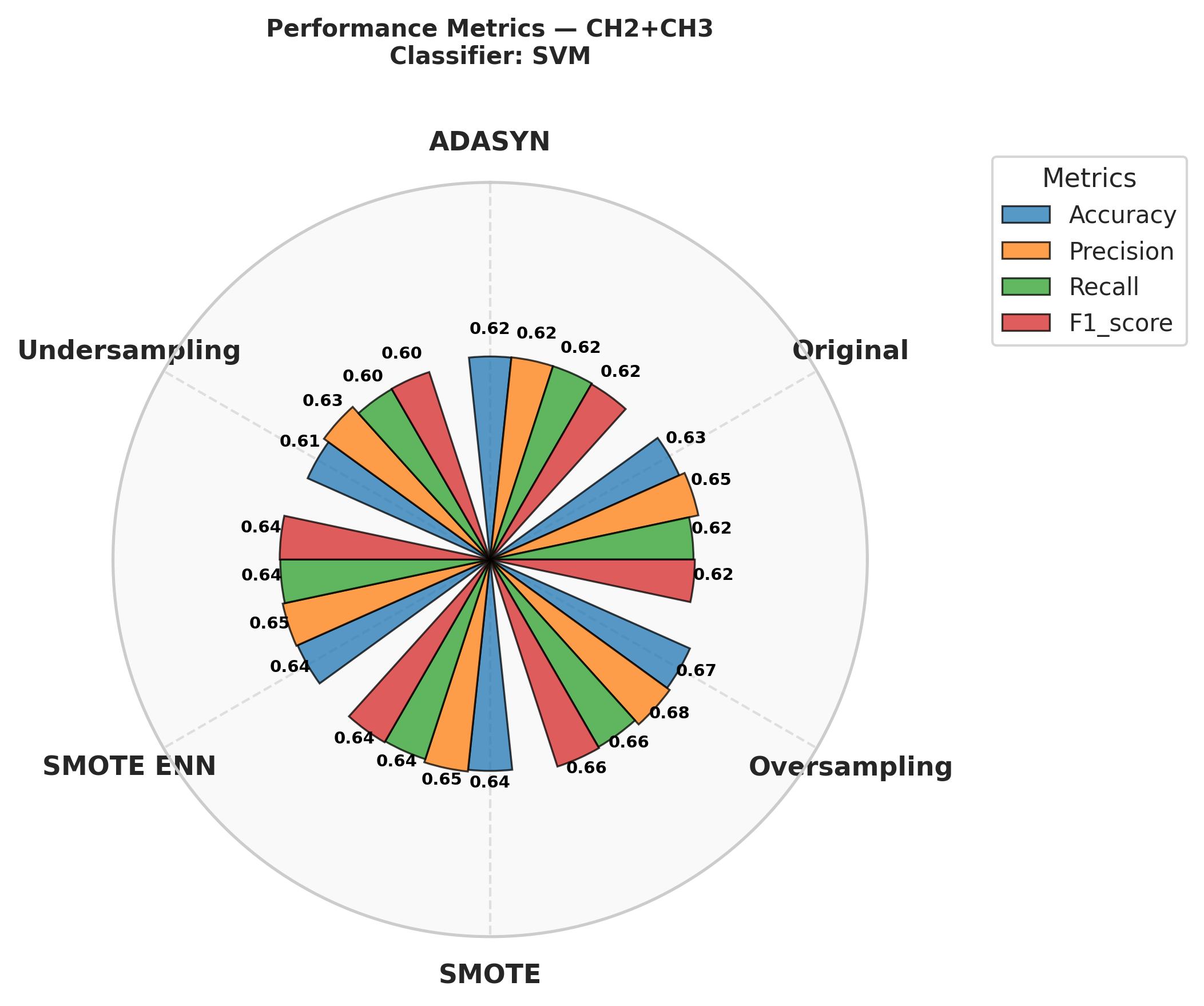

Supplement: Supplementary file 1 [file bioengineering-13-00787-s001.zip › Supplementary Material - Performance Metrics/CH2+CH3_SVM_polar.png]

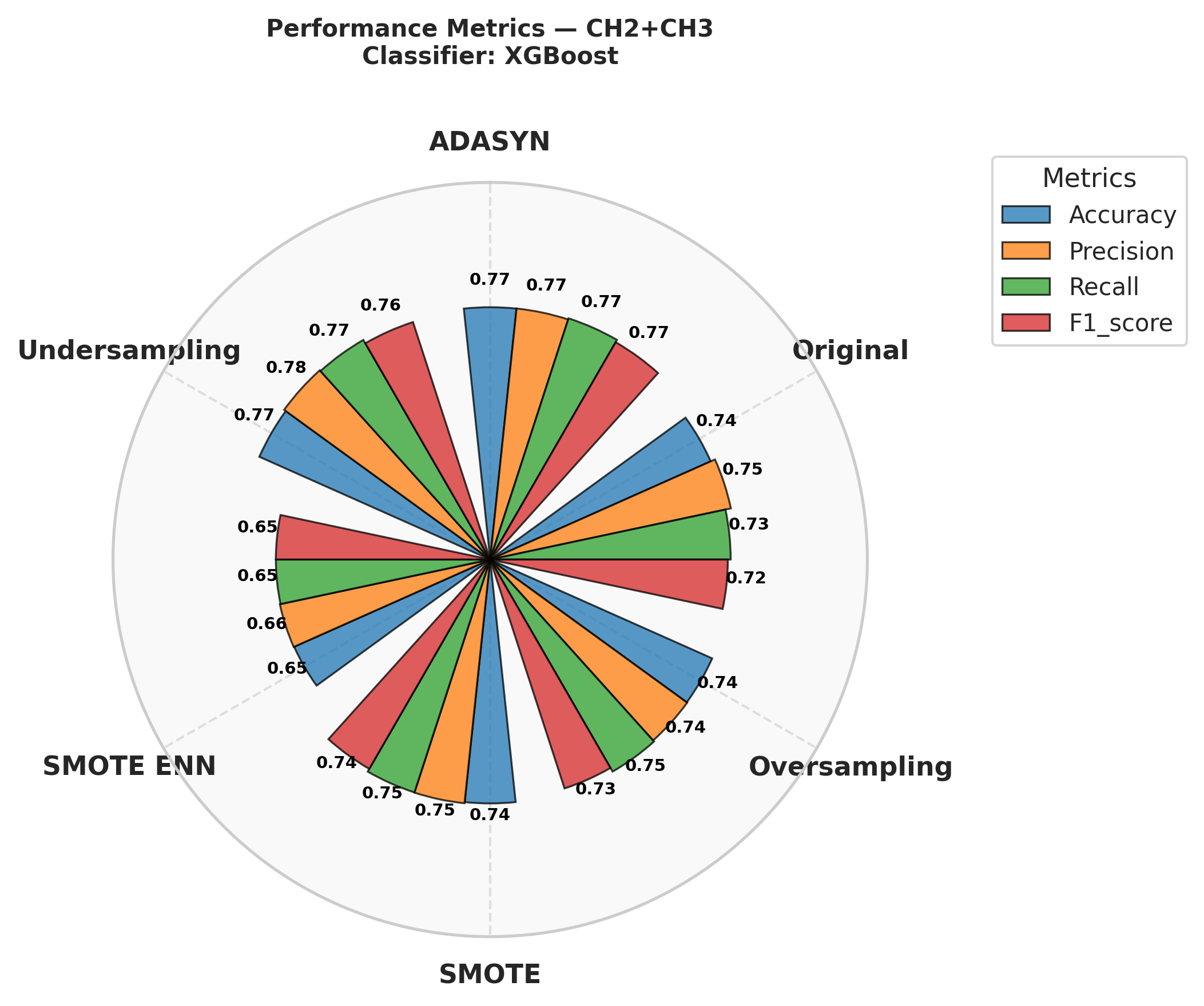

Supplement: Supplementary file 1 [file bioengineering-13-00787-s001.zip › Supplementary Material - Performance Metrics/CH2+CH3_XGBoost_polar.png]

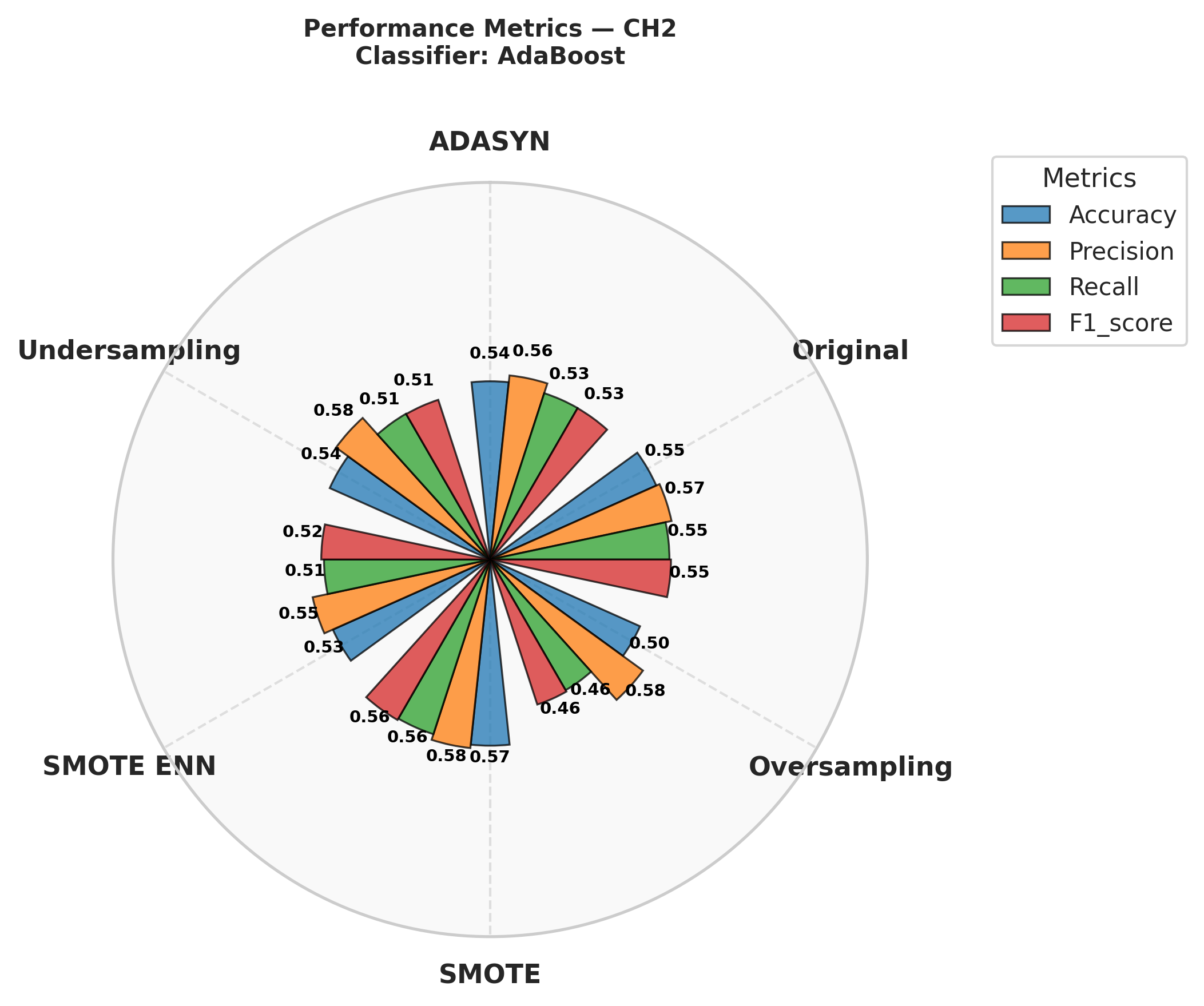

Supplement: Supplementary file 1 [file bioengineering-13-00787-s001.zip › Supplementary Material - Performance Metrics/CH2_AdaBoost_polar.png]

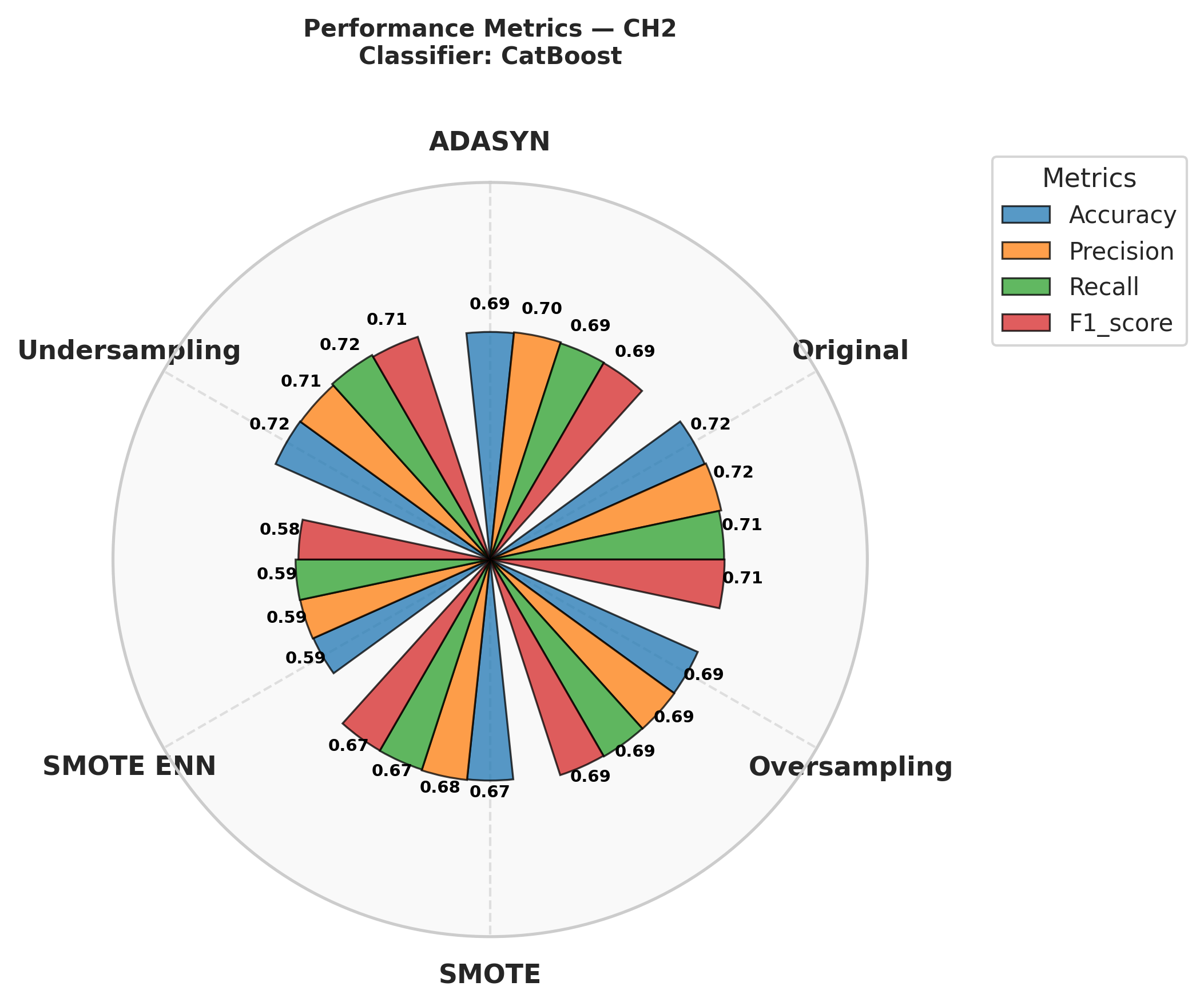

Supplement: Supplementary file 1 [file bioengineering-13-00787-s001.zip › Supplementary Material - Performance Metrics/CH2_CatBoost_polar.png]

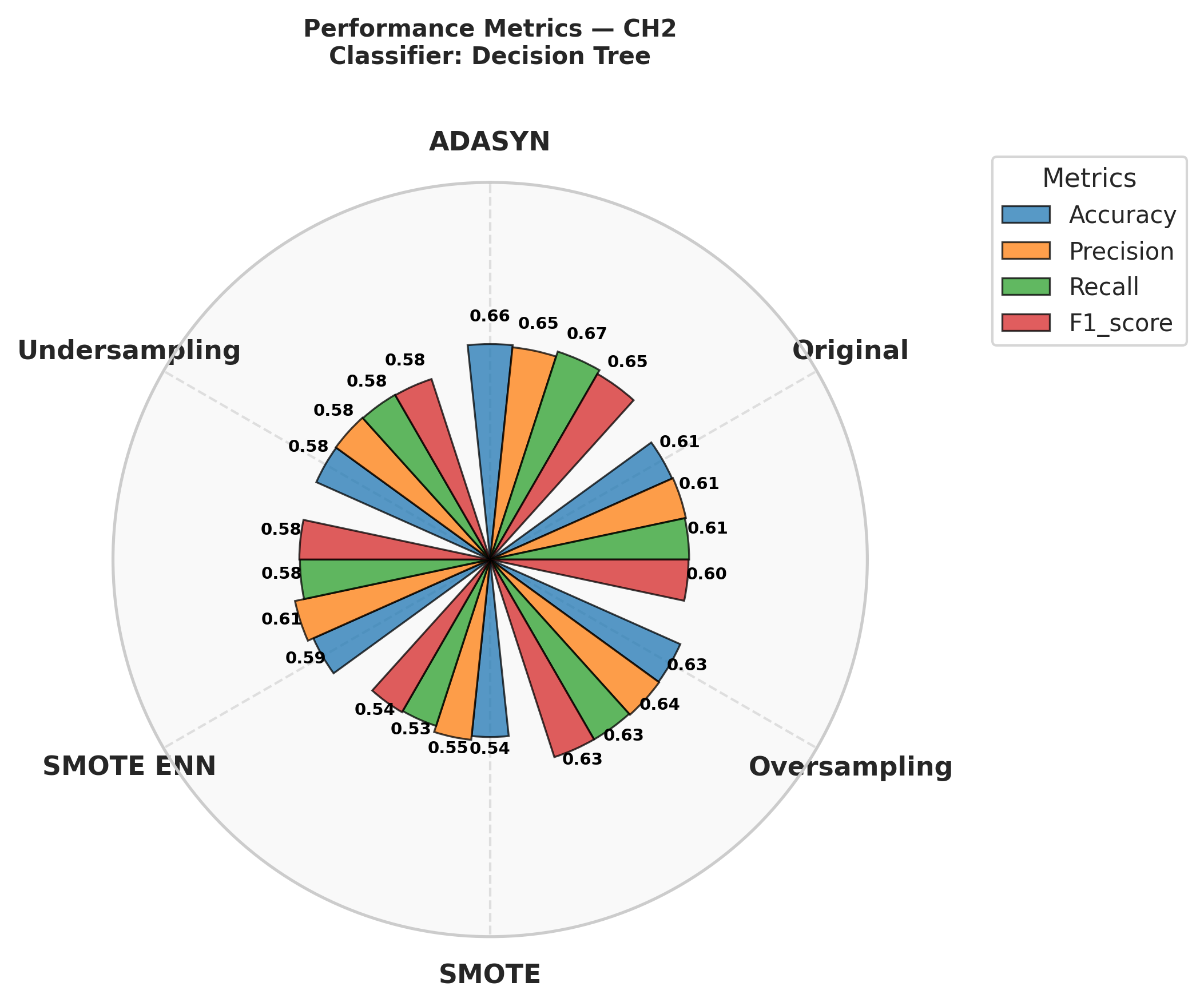

Supplement: Supplementary file 1 [file bioengineering-13-00787-s001.zip › Supplementary Material - Performance Metrics/CH2_Decision Tree_polar.png]

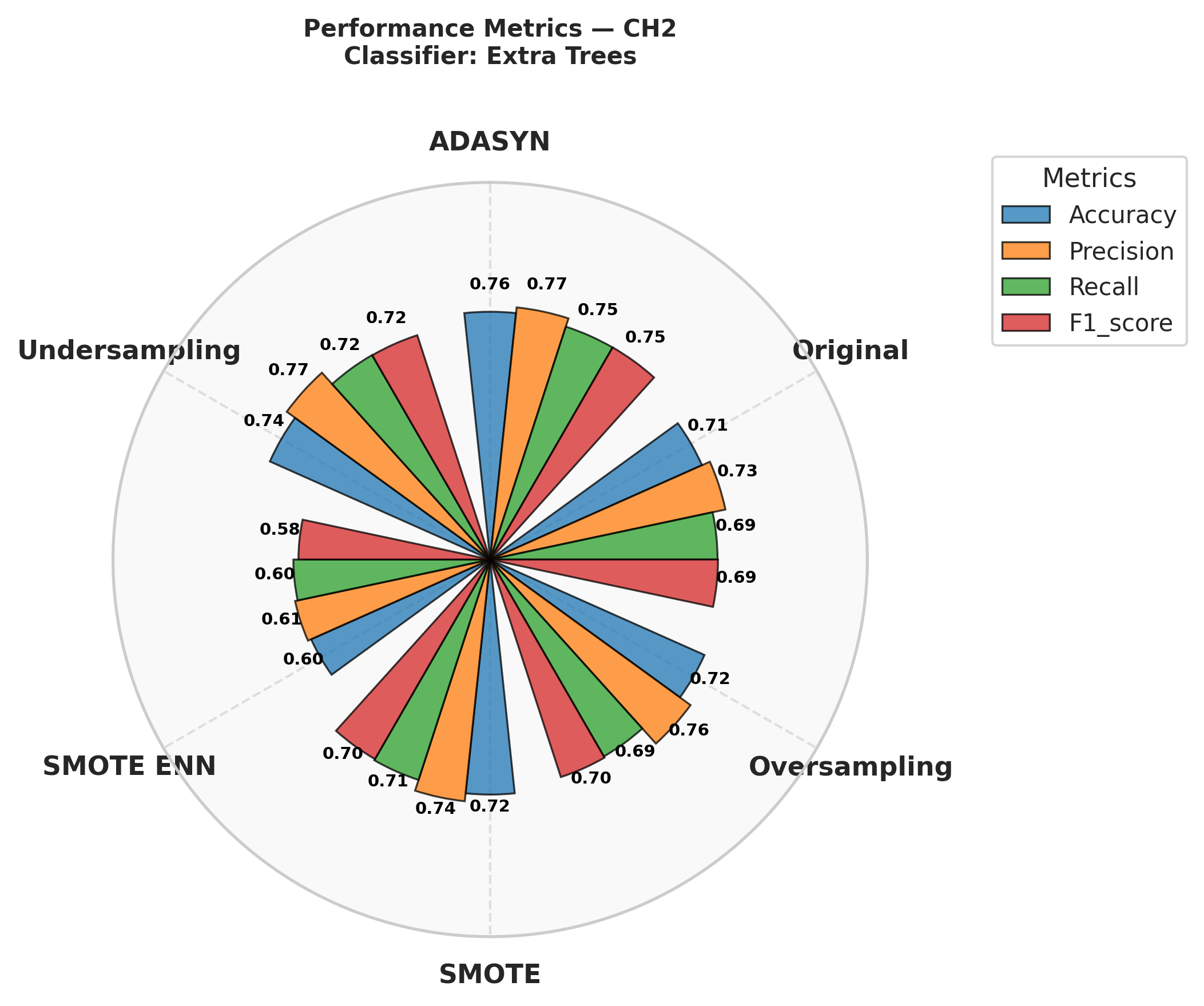

Supplement: Supplementary file 1 [file bioengineering-13-00787-s001.zip › Supplementary Material - Performance Metrics/CH2_Extra Trees_polar.png]

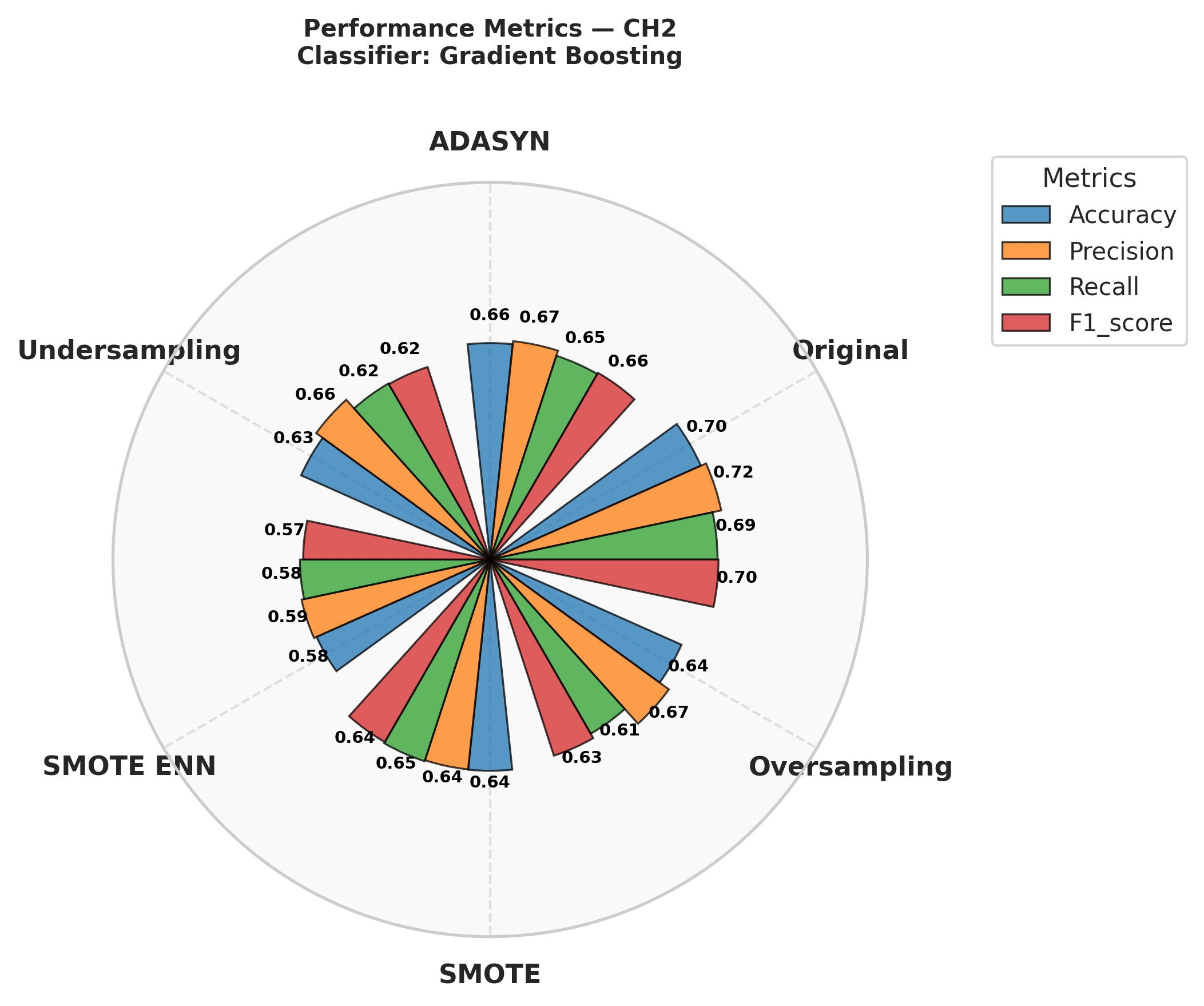

Supplement: Supplementary file 1 [file bioengineering-13-00787-s001.zip › Supplementary Material - Performance Metrics/CH2_Gradient Boosting_polar.png]

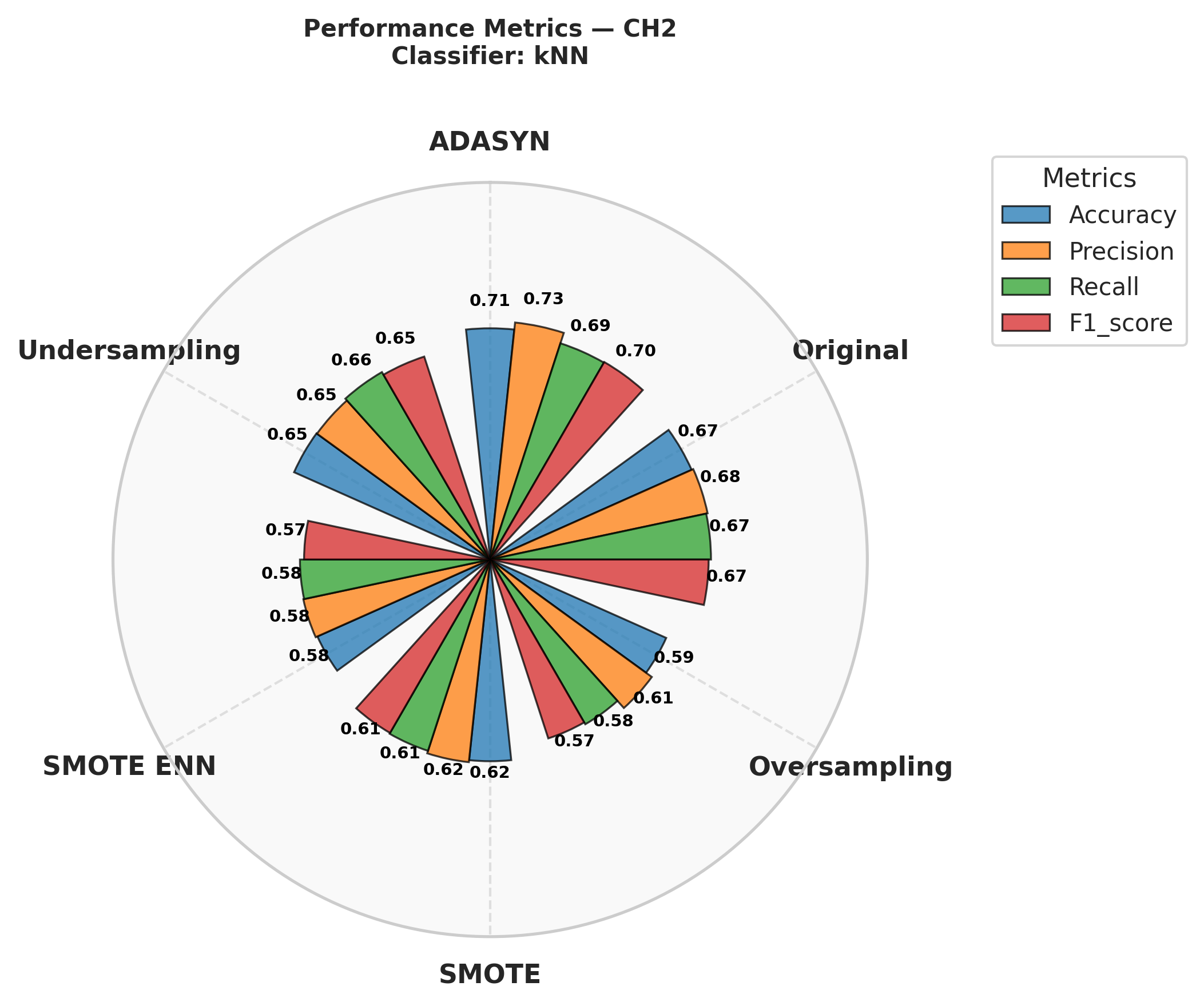

Supplement: Supplementary file 1 [file bioengineering-13-00787-s001.zip › Supplementary Material - Performance Metrics/CH2_kNN_polar.png]

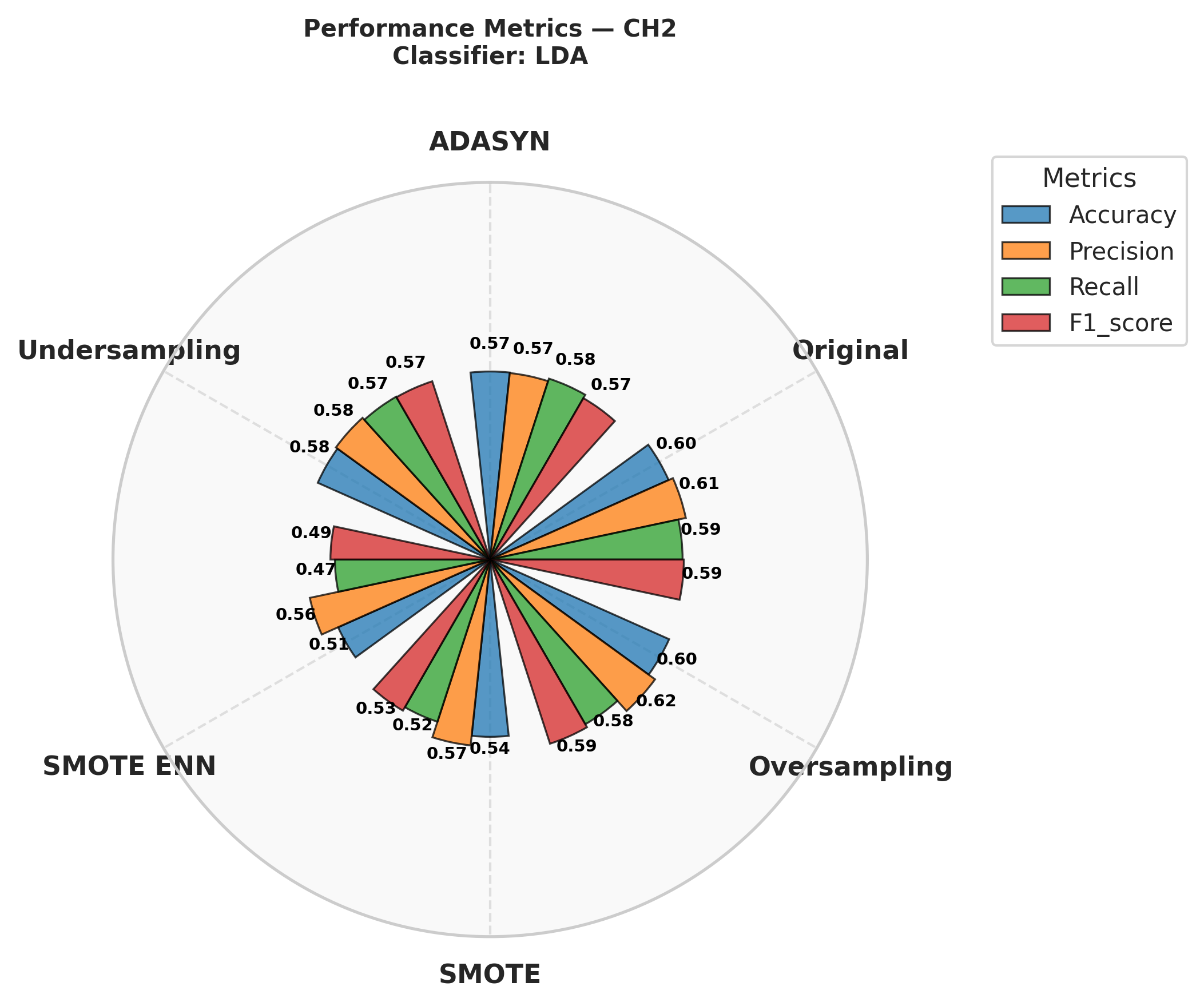

Supplement: Supplementary file 1 [file bioengineering-13-00787-s001.zip › Supplementary Material - Performance Metrics/CH2_LDA_polar.png]

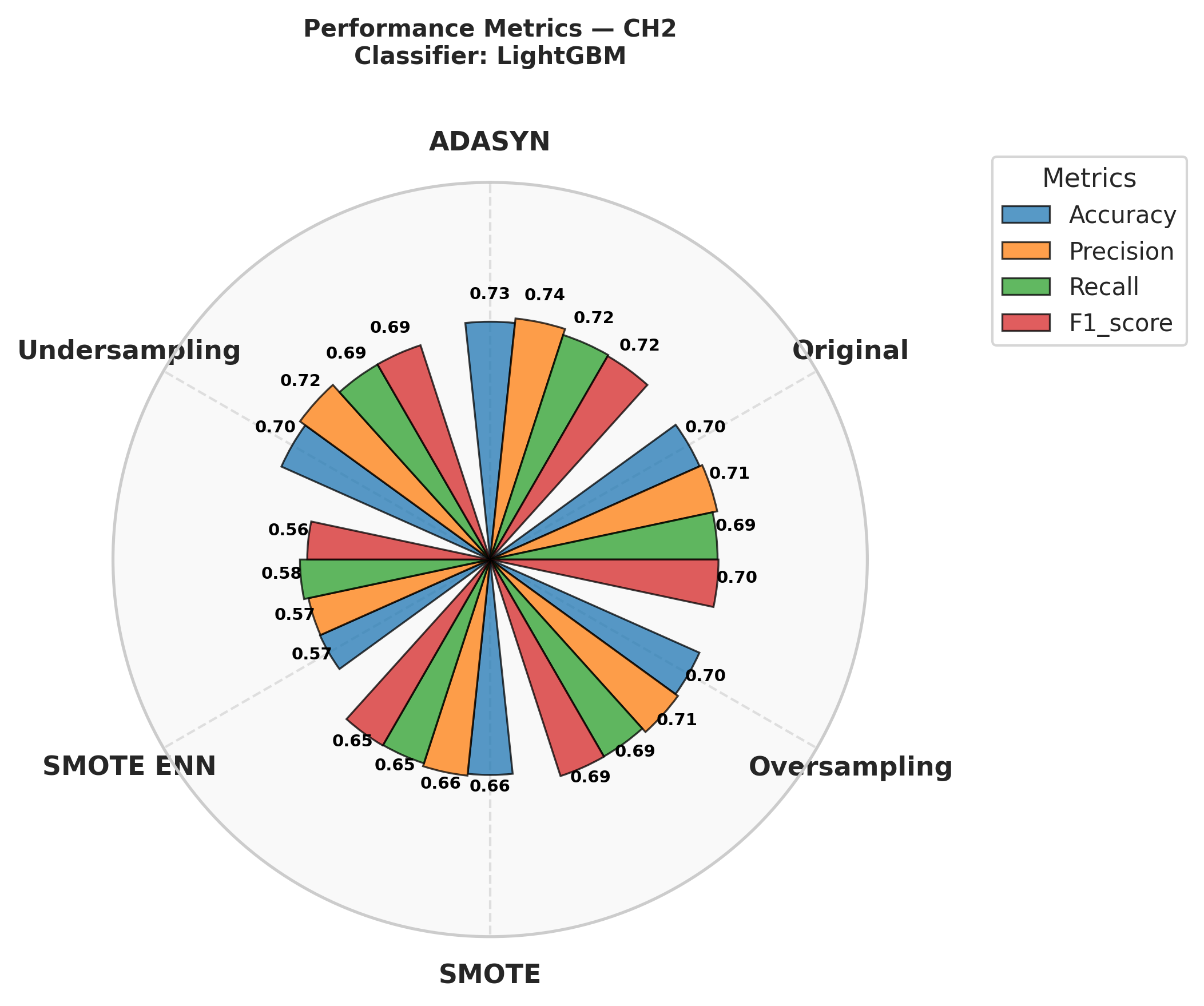

Supplement: Supplementary file 1 [file bioengineering-13-00787-s001.zip › Supplementary Material - Performance Metrics/CH2_LightGBM_polar.png]

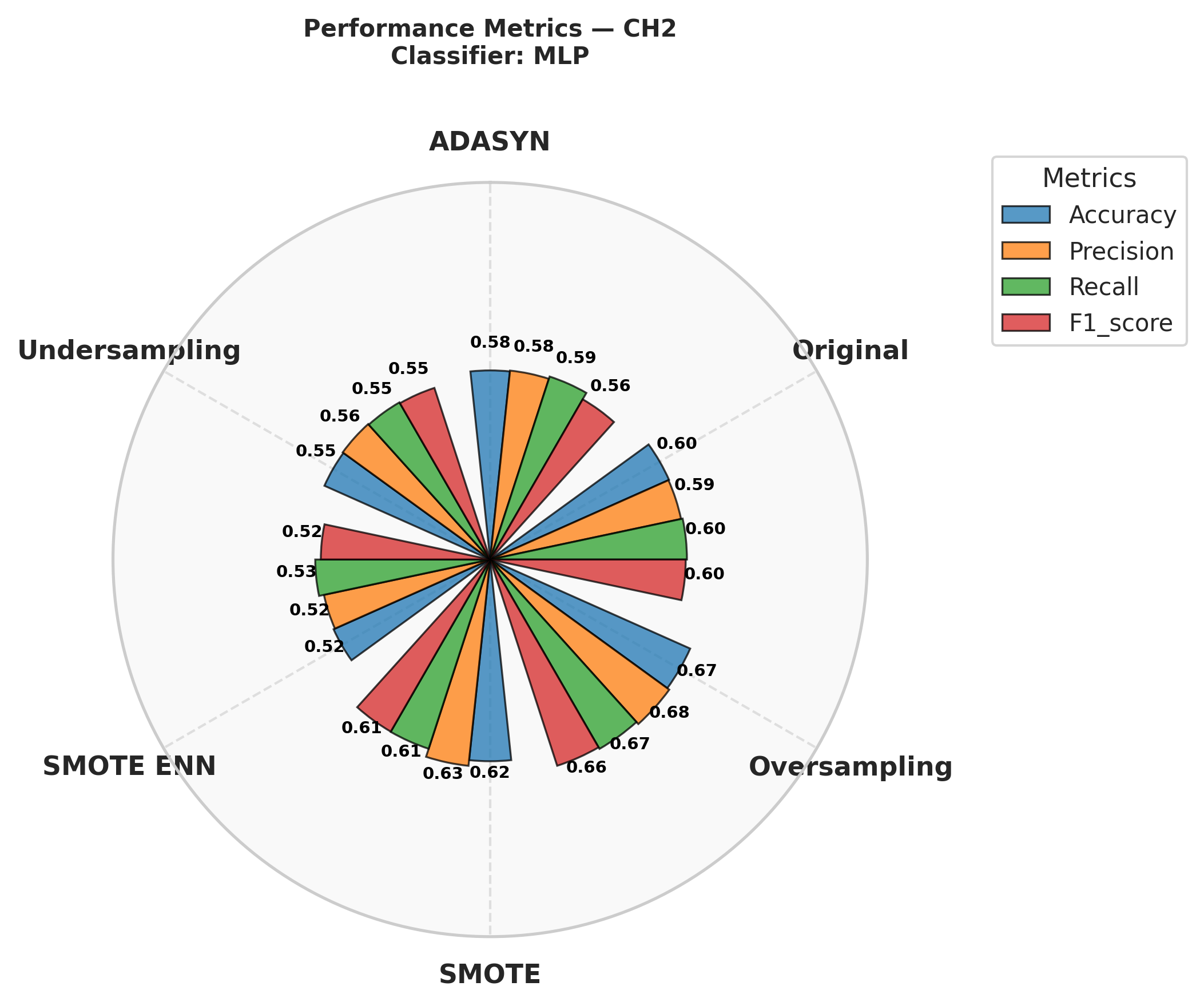

Supplement: Supplementary file 1 [file bioengineering-13-00787-s001.zip › Supplementary Material - Performance Metrics/CH2_MLP_polar.png]

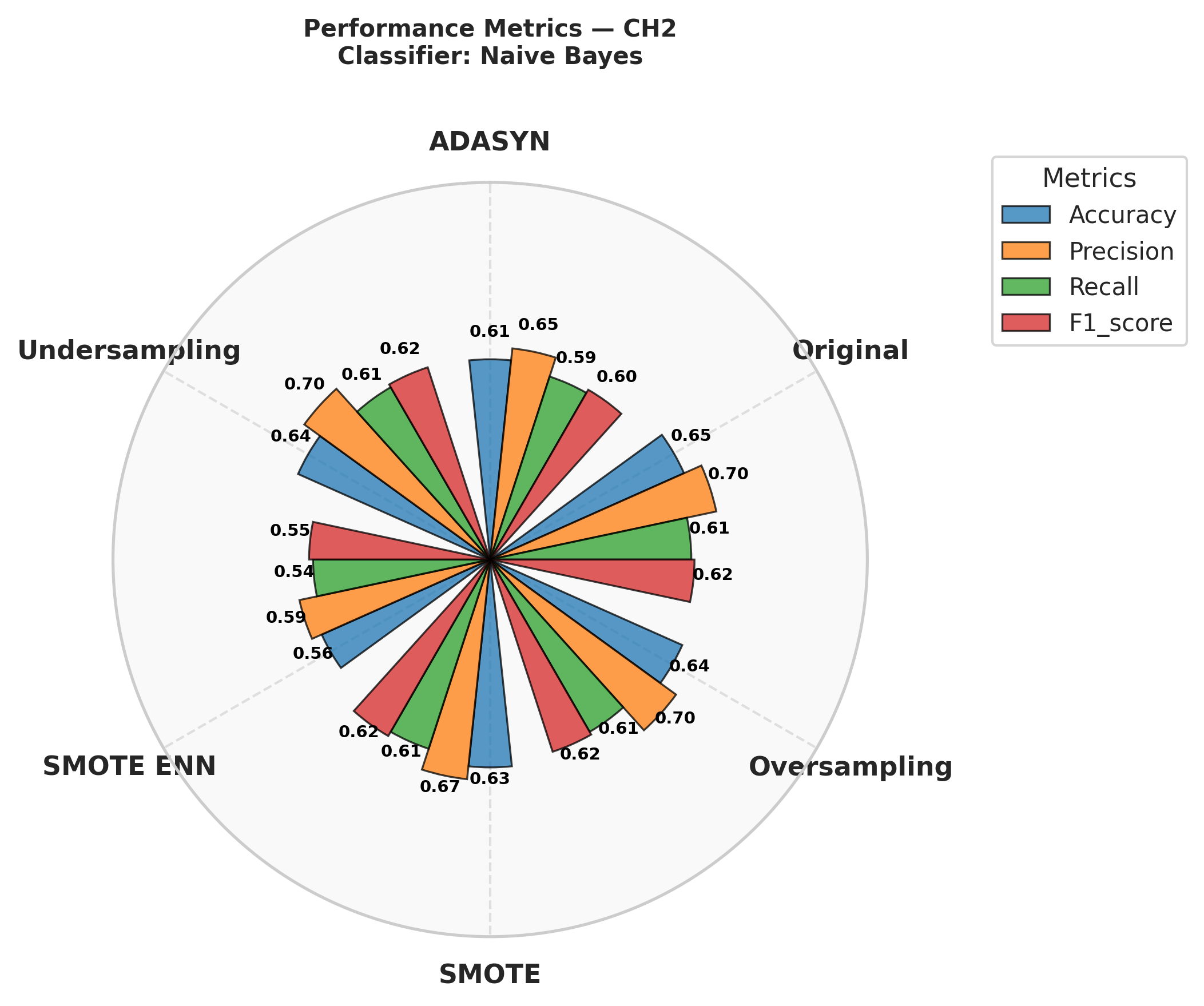

Supplement: Supplementary file 1 [file bioengineering-13-00787-s001.zip › Supplementary Material - Performance Metrics/CH2_Naive Bayes_polar.png]

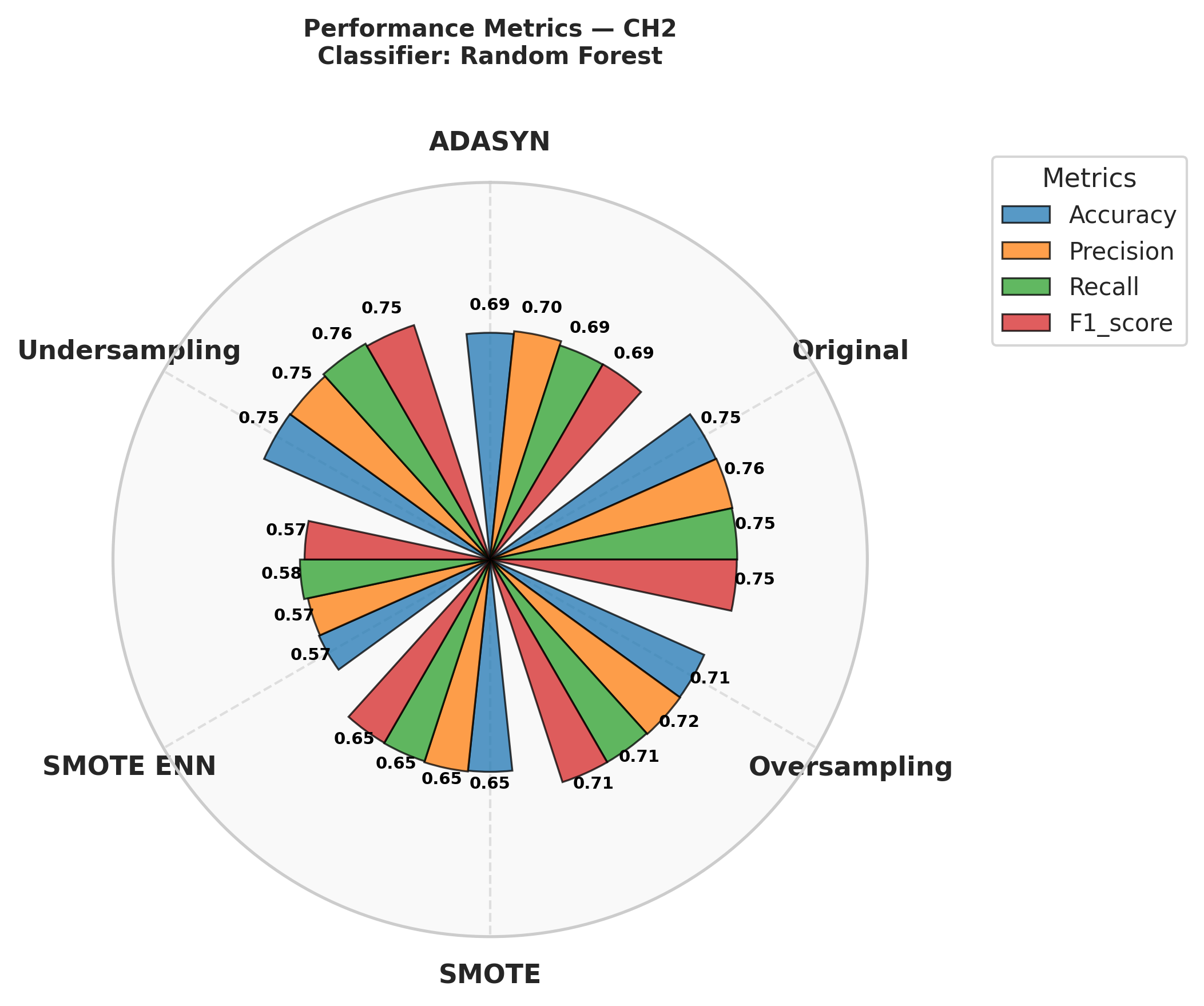

Supplement: Supplementary file 1 [file bioengineering-13-00787-s001.zip › Supplementary Material - Performance Metrics/CH2_Random Forest_polar.png]

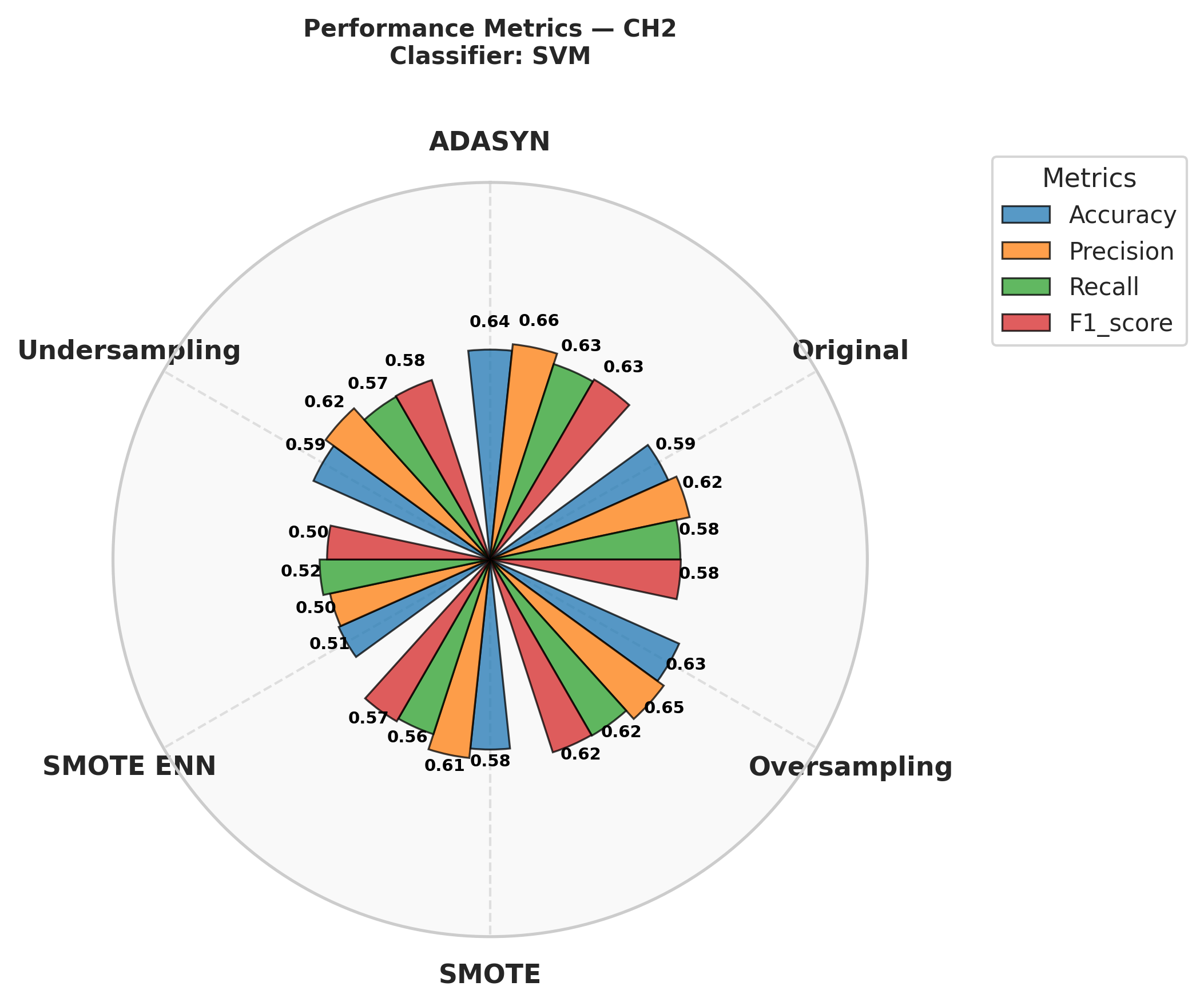

Supplement: Supplementary file 1 [file bioengineering-13-00787-s001.zip › Supplementary Material - Performance Metrics/CH2_SVM_polar.png]

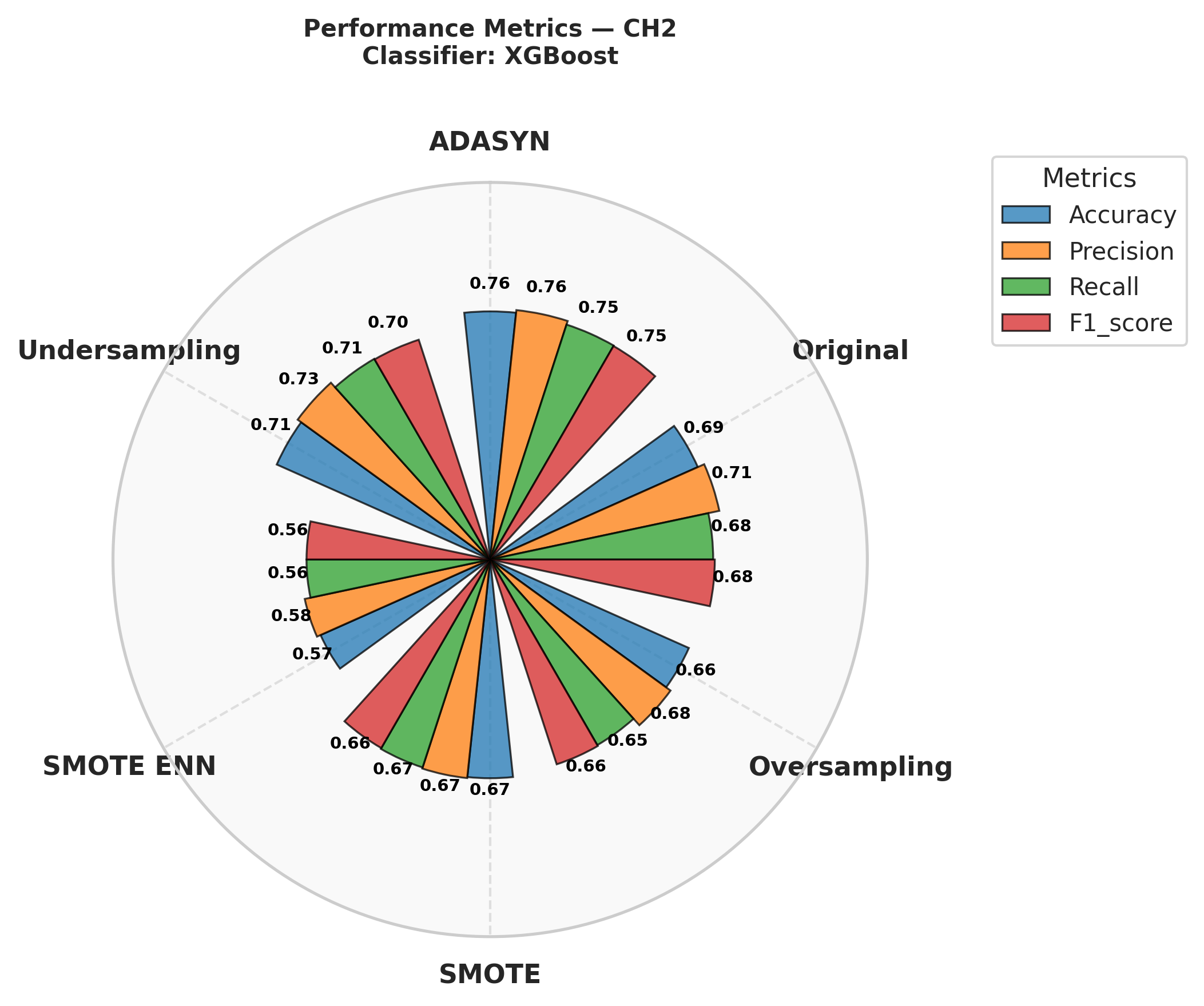

Supplement: Supplementary file 1 [file bioengineering-13-00787-s001.zip › Supplementary Material - Performance Metrics/CH2_XGBoost_polar.png]

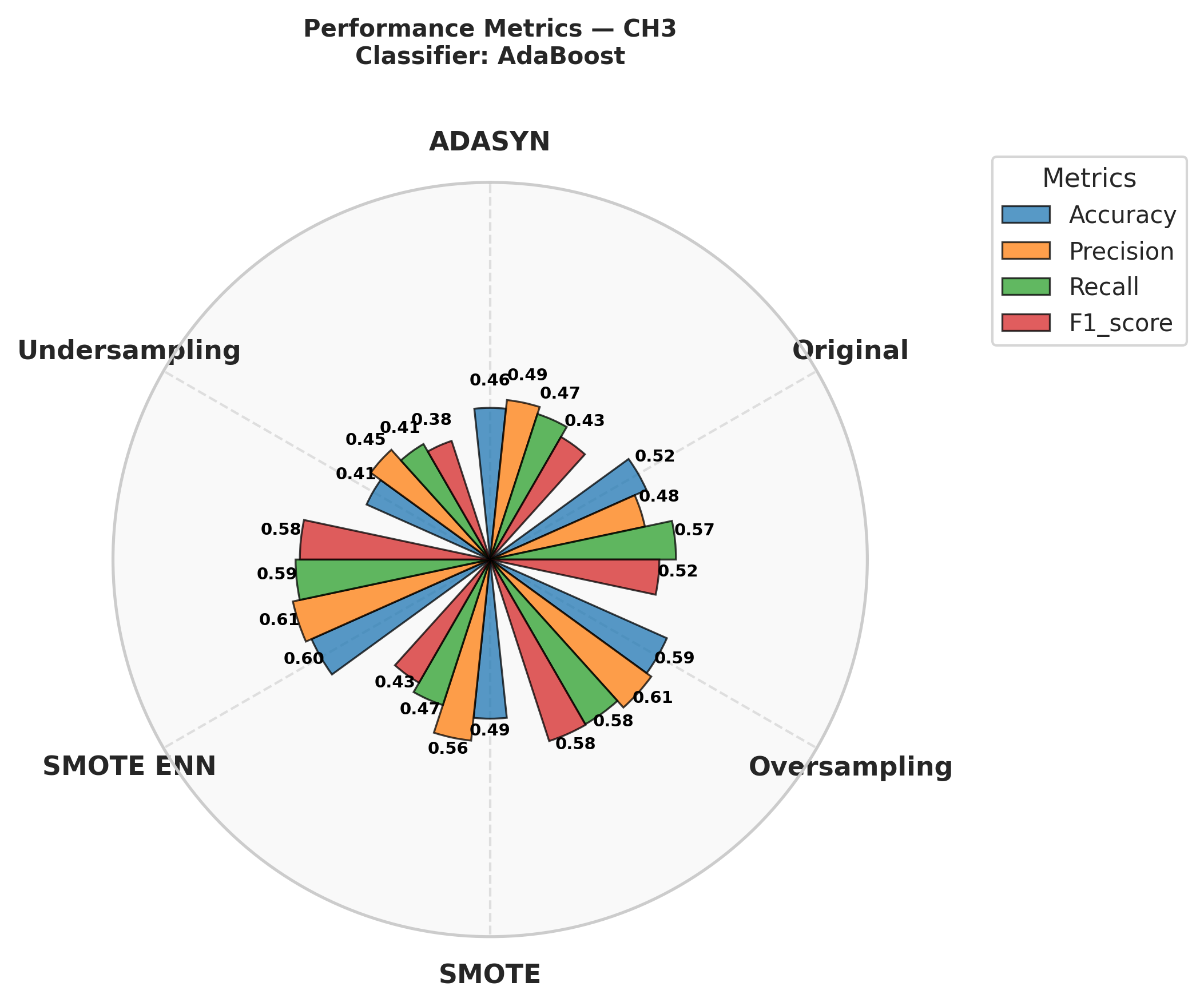

Supplement: Supplementary file 1 [file bioengineering-13-00787-s001.zip › Supplementary Material - Performance Metrics/CH3_AdaBoost_polar.png]

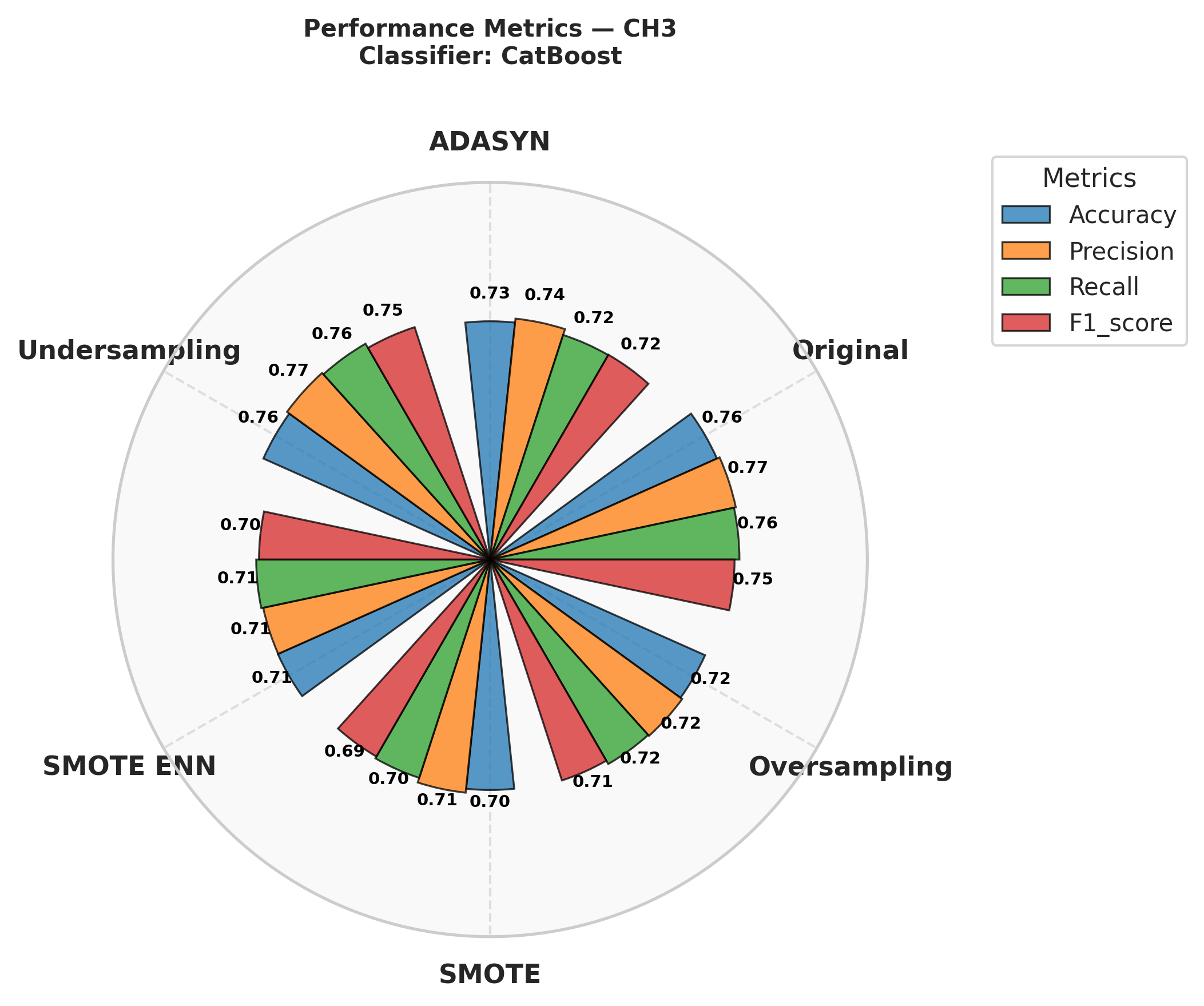

Supplement: Supplementary file 1 [file bioengineering-13-00787-s001.zip › Supplementary Material - Performance Metrics/CH3_CatBoost_polar.png]

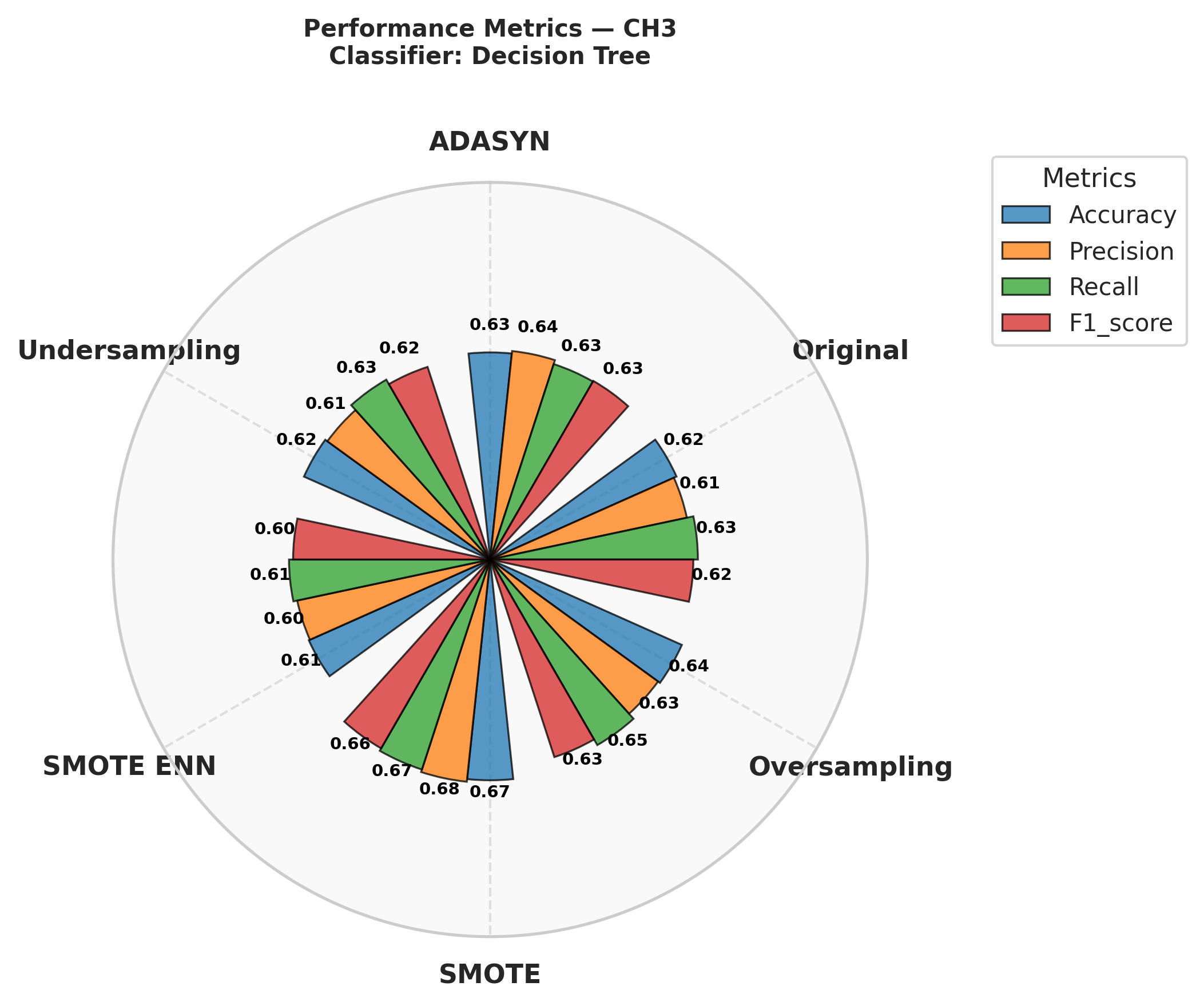

Supplement: Supplementary file 1 [file bioengineering-13-00787-s001.zip › Supplementary Material - Performance Metrics/CH3_Decision Tree_polar.png]

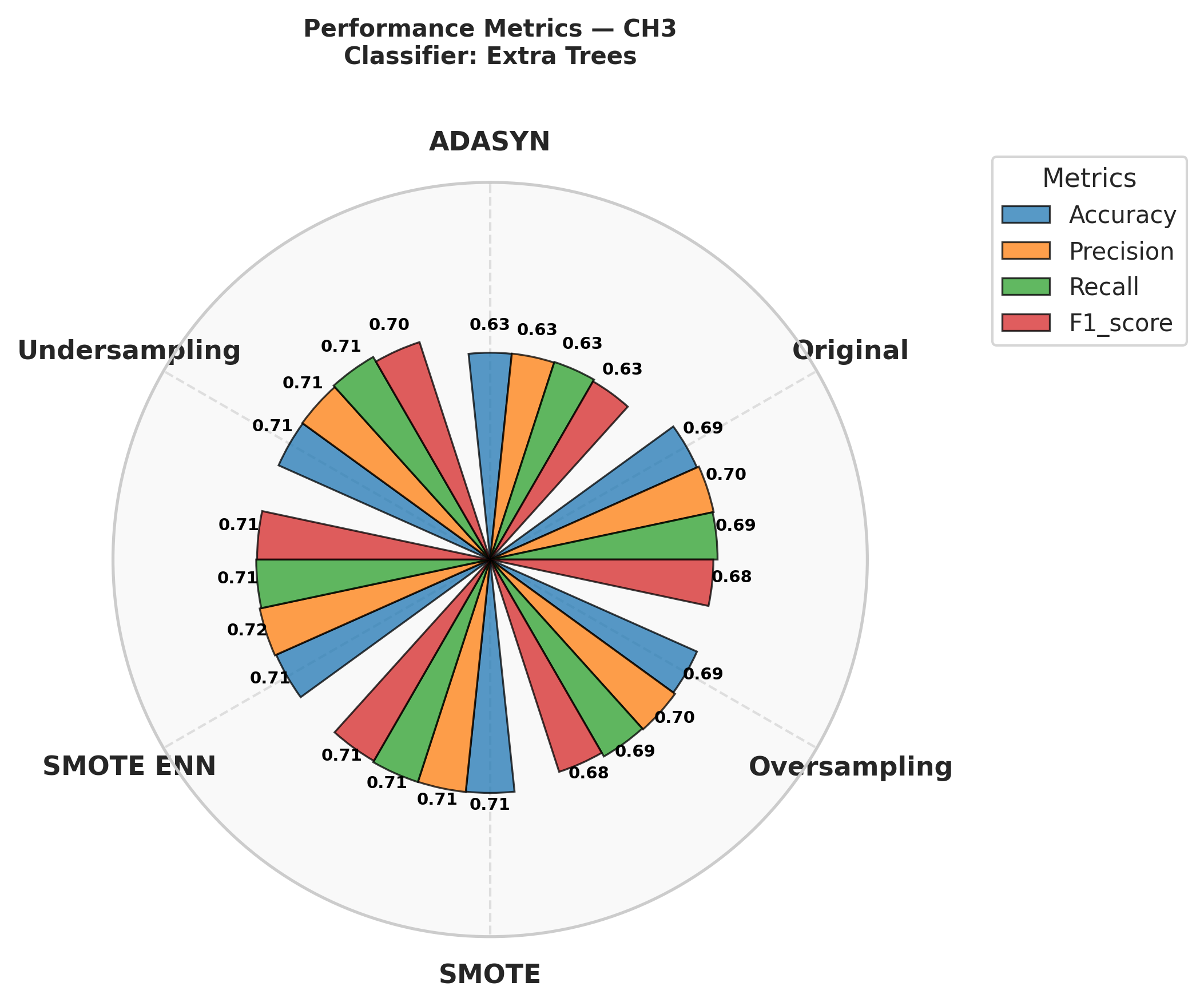

Supplement: Supplementary file 1 [file bioengineering-13-00787-s001.zip › Supplementary Material - Performance Metrics/CH3_Extra Trees_polar.png]

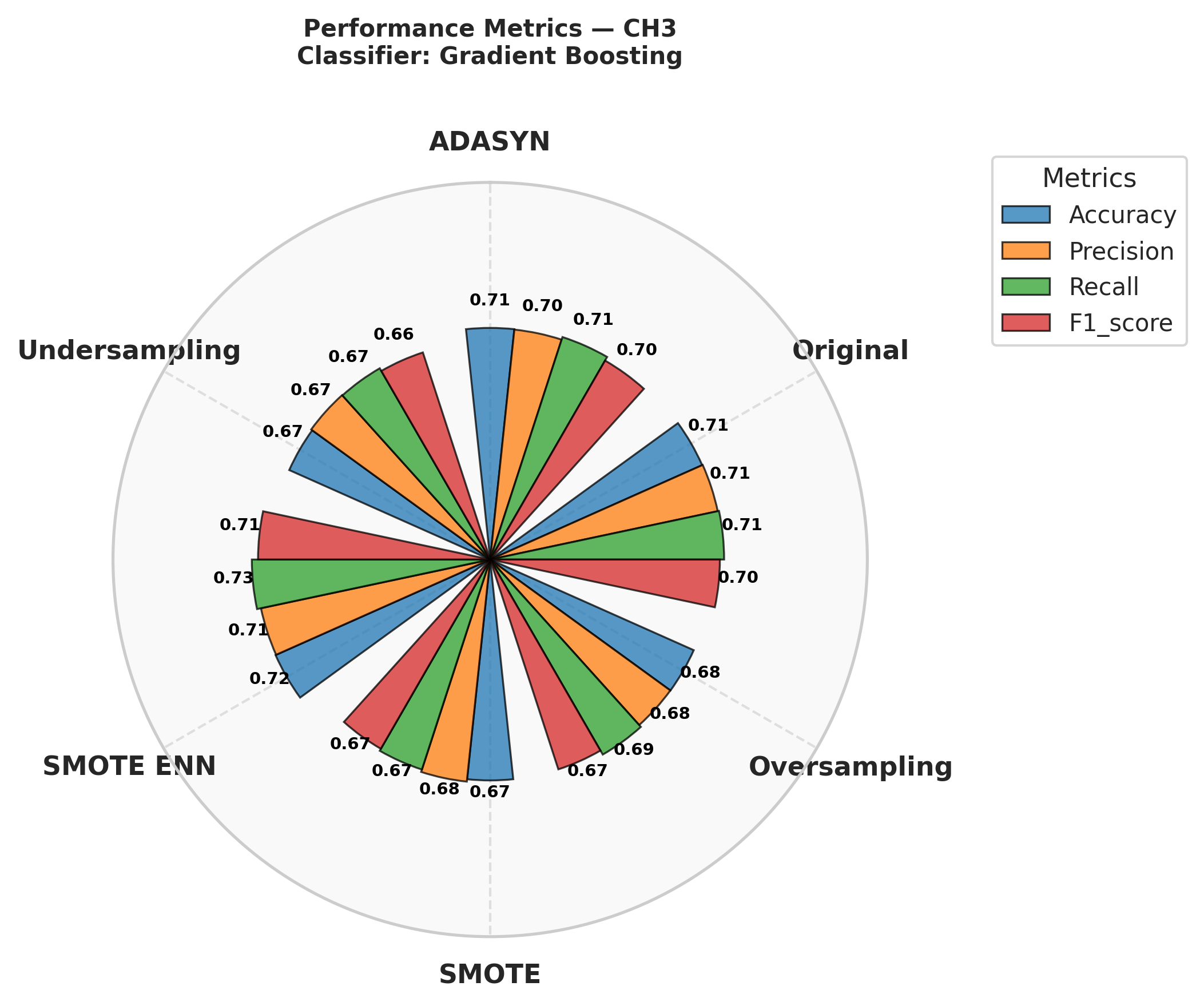

Supplement: Supplementary file 1 [file bioengineering-13-00787-s001.zip › Supplementary Material - Performance Metrics/CH3_Gradient Boosting_polar.png]

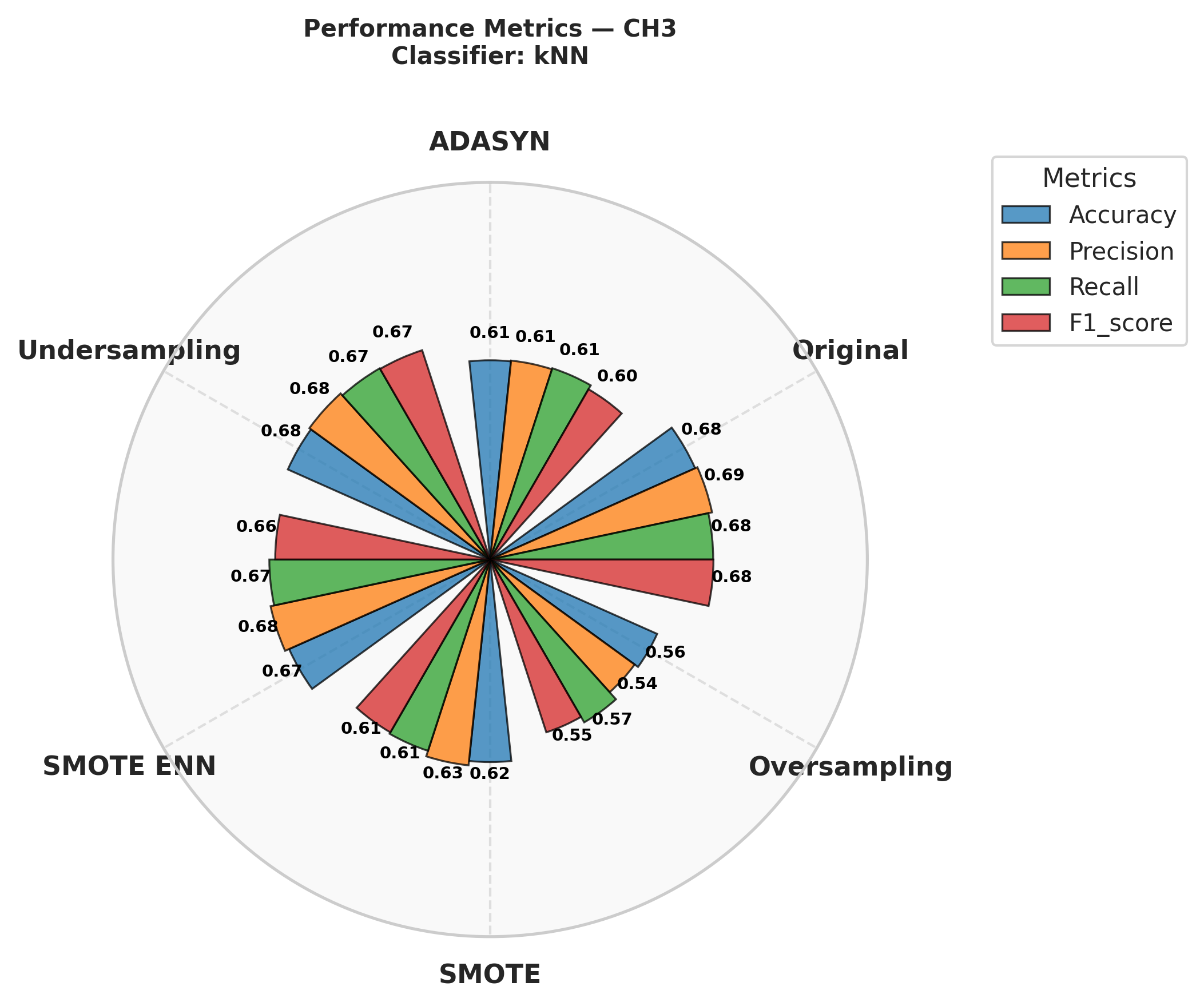

Supplement: Supplementary file 1 [file bioengineering-13-00787-s001.zip › Supplementary Material - Performance Metrics/CH3_kNN_polar.png]

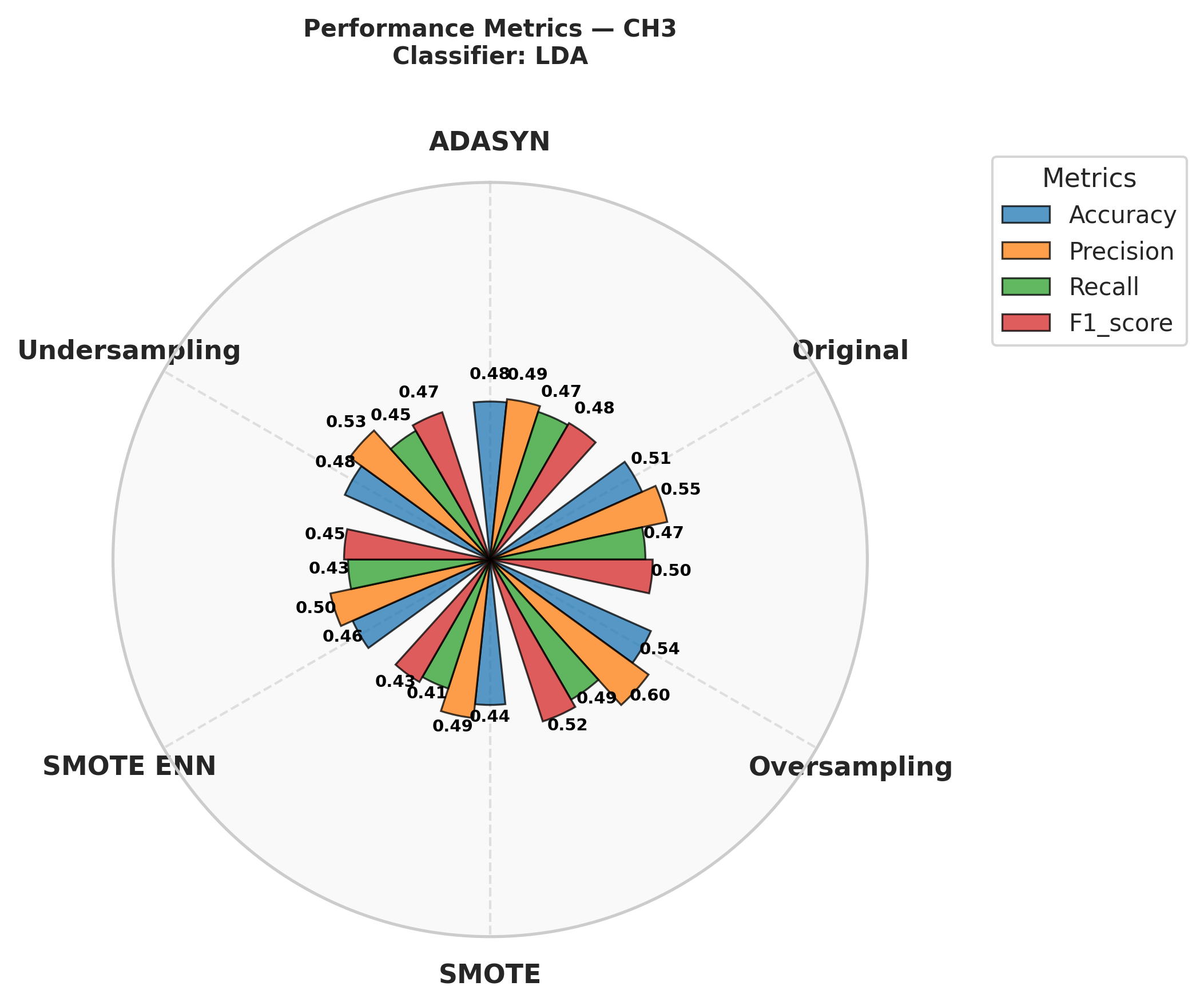

Supplement: Supplementary file 1 [file bioengineering-13-00787-s001.zip › Supplementary Material - Performance Metrics/CH3_LDA_polar.png]

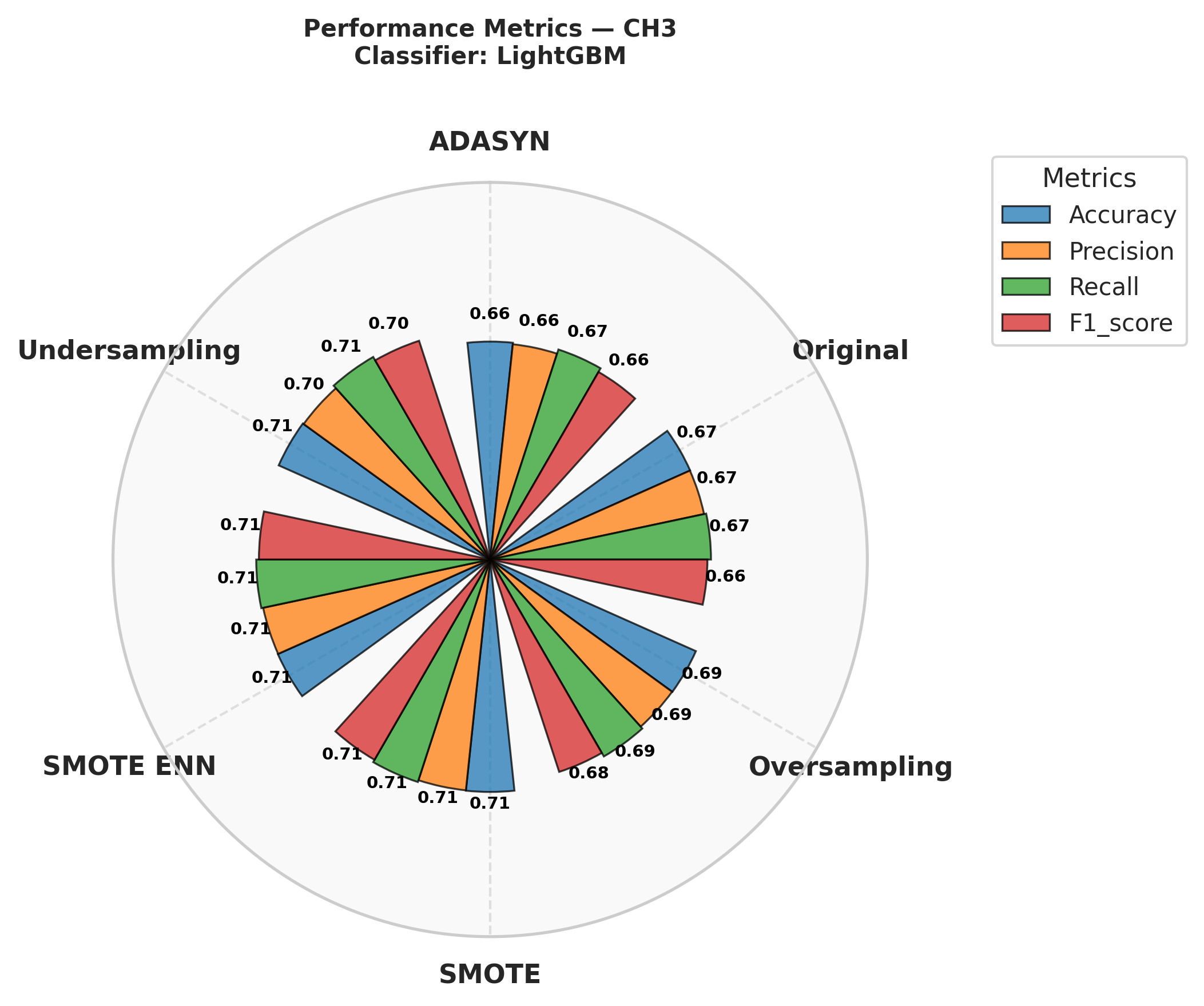

Supplement: Supplementary file 1 [file bioengineering-13-00787-s001.zip › Supplementary Material - Performance Metrics/CH3_LightGBM_polar.png]

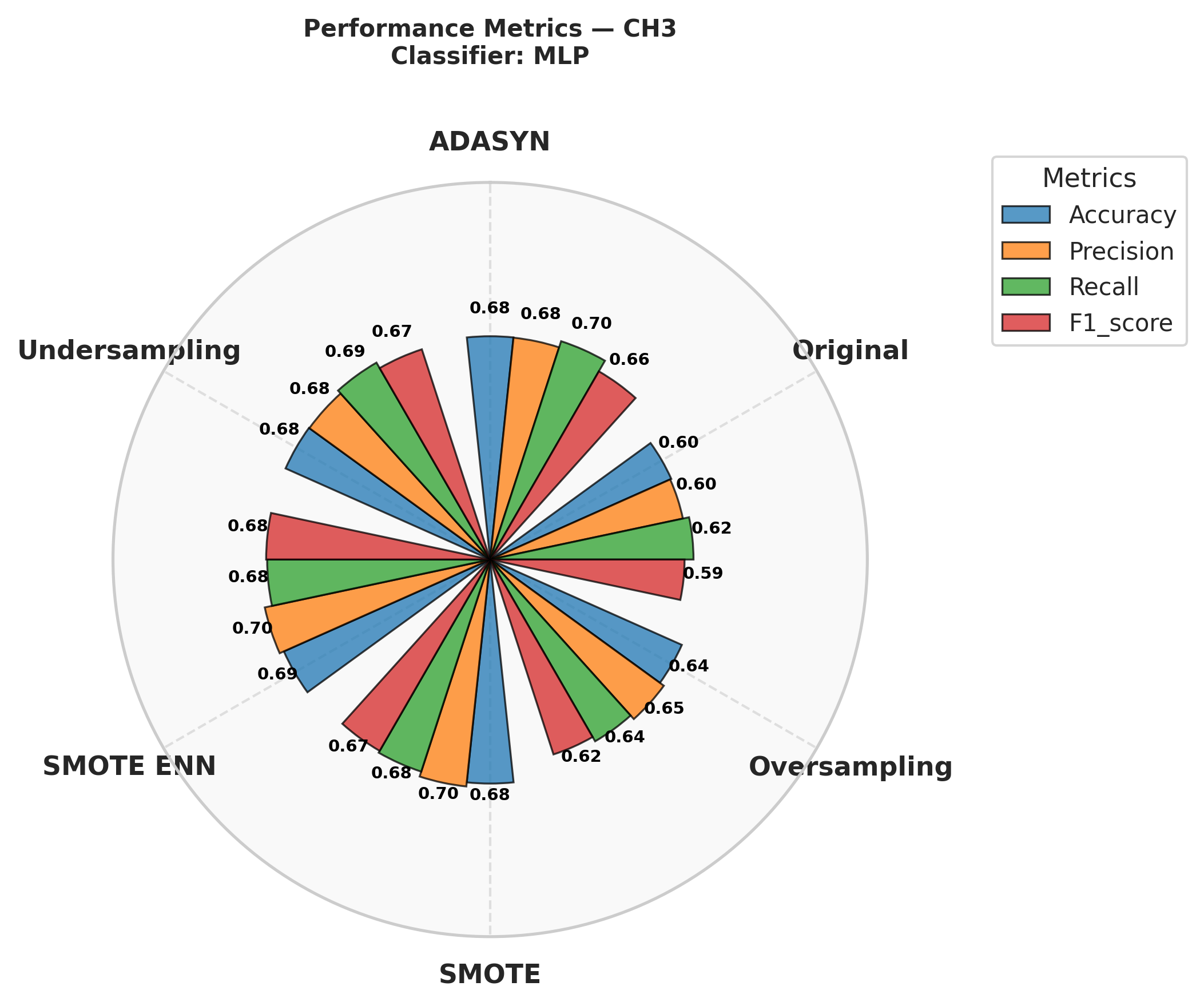

Supplement: Supplementary file 1 [file bioengineering-13-00787-s001.zip › Supplementary Material - Performance Metrics/CH3_MLP_polar.png]

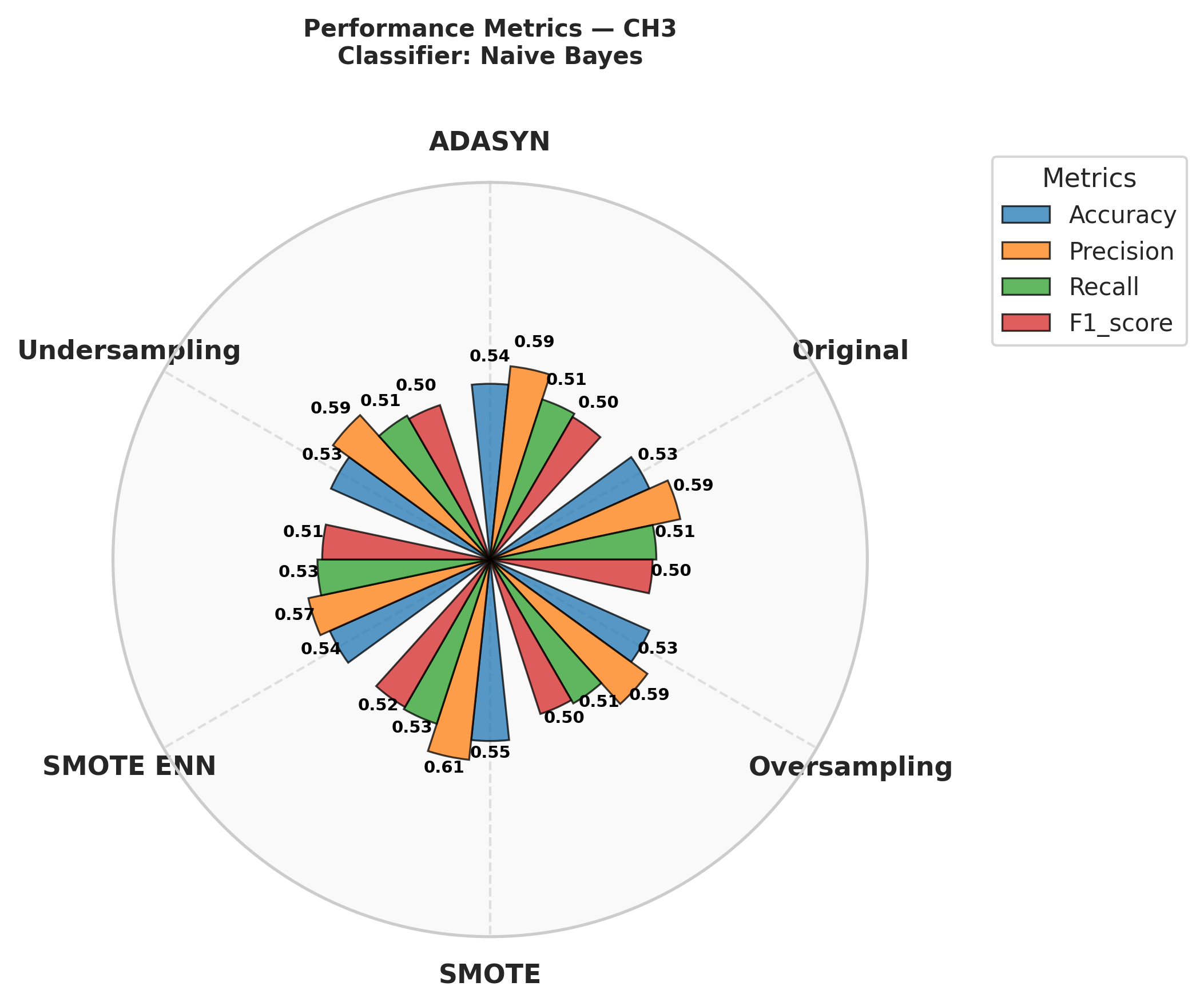

Supplement: Supplementary file 1 [file bioengineering-13-00787-s001.zip › Supplementary Material - Performance Metrics/CH3_Naive Bayes_polar.png]

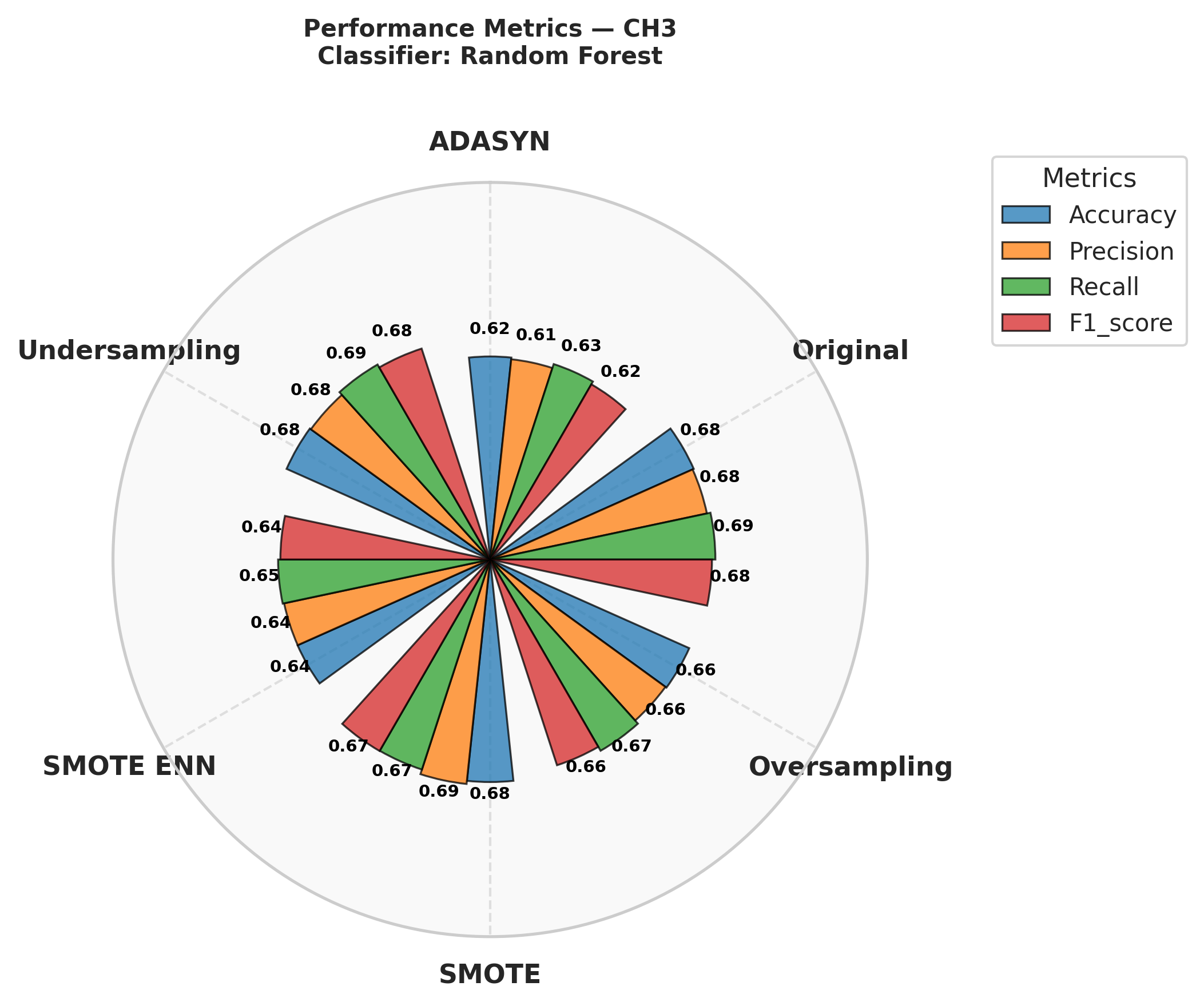

Supplement: Supplementary file 1 [file bioengineering-13-00787-s001.zip › Supplementary Material - Performance Metrics/CH3_Random Forest_polar.png]

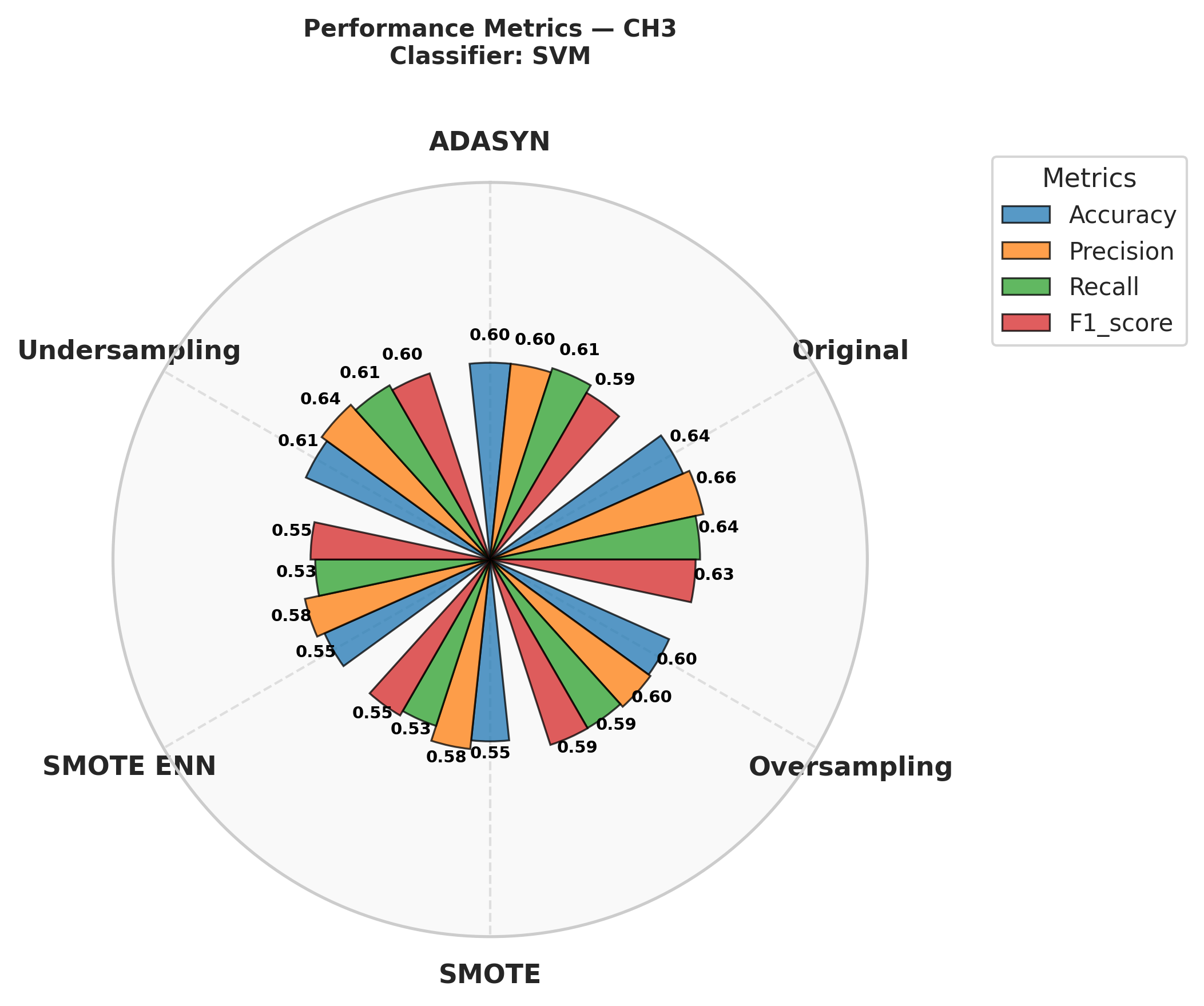

Supplement: Supplementary file 1 [file bioengineering-13-00787-s001.zip › Supplementary Material - Performance Metrics/CH3_SVM_polar.png]

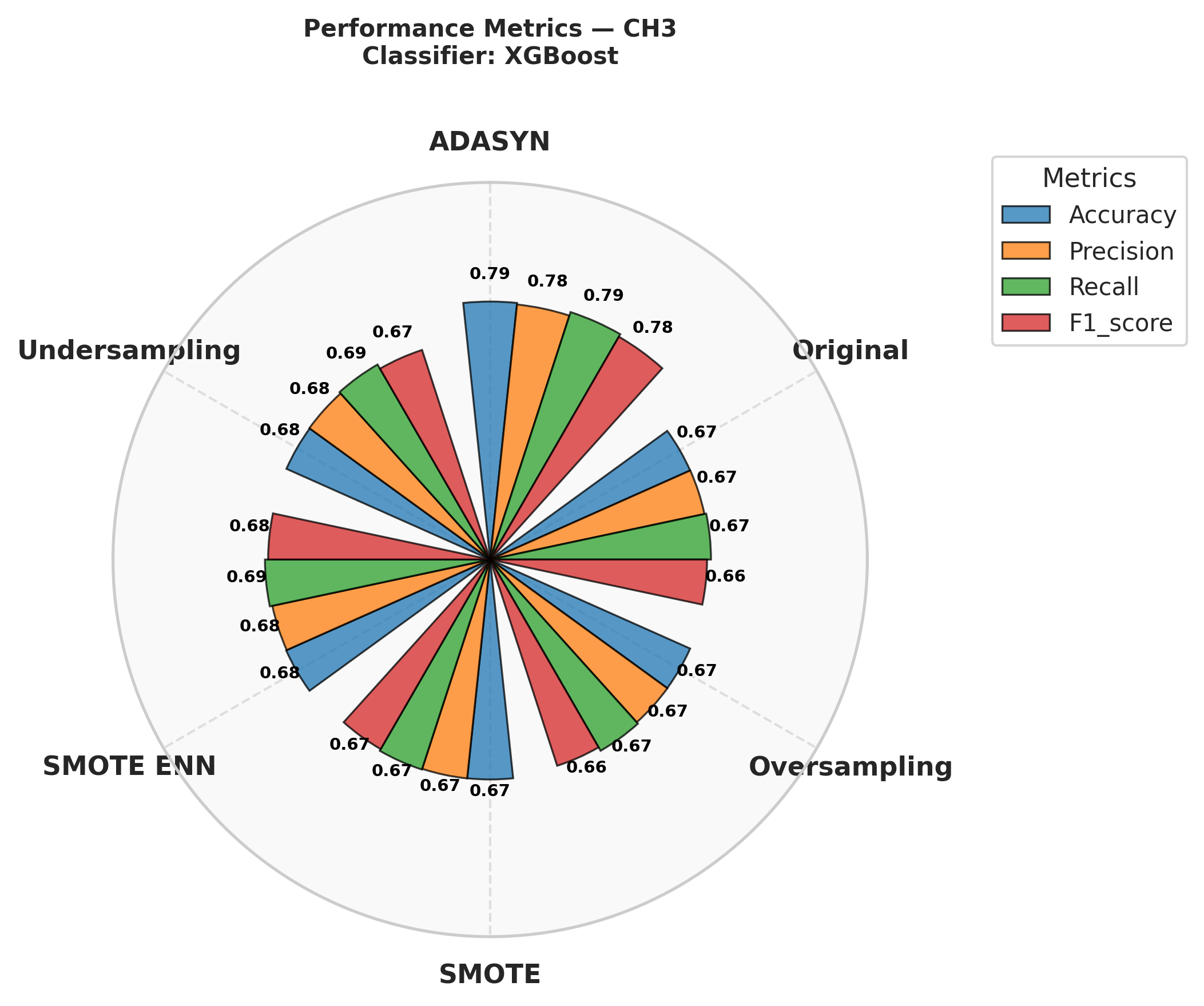

Supplement: Supplementary file 1 [file bioengineering-13-00787-s001.zip › Supplementary Material - Performance Metrics/CH3_XGBoost_polar.png]

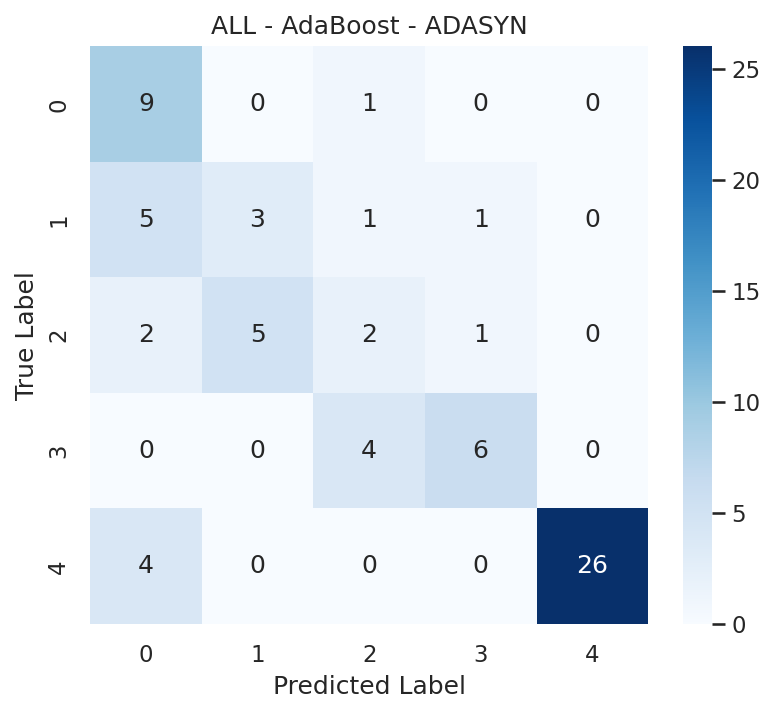

Supplement: Supplementary file 1 [file bioengineering-13-00787-s001.zip › Supplementary Material - Performance Metrics/cm_ALL_AdaBoost_ADASYN.png]

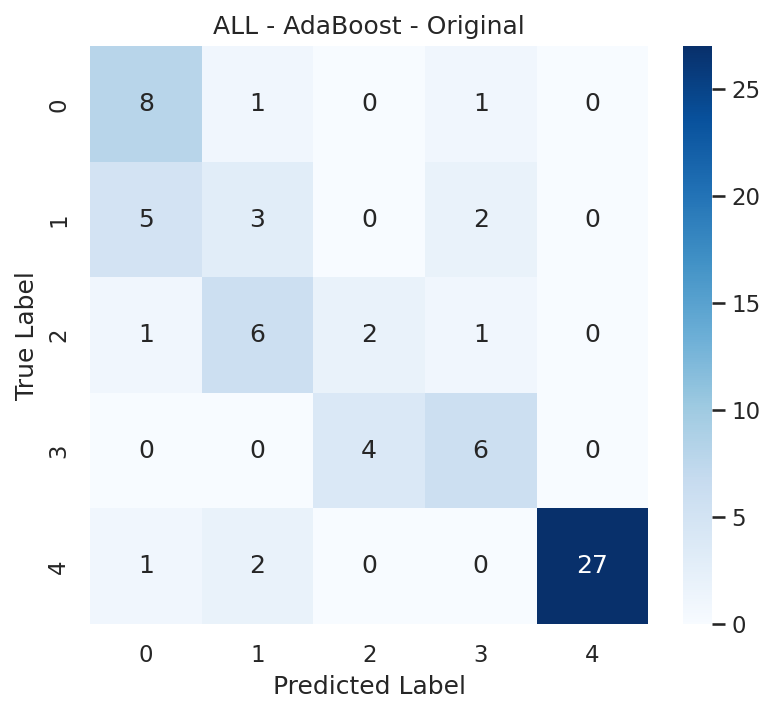

Supplement: Supplementary file 1 [file bioengineering-13-00787-s001.zip › Supplementary Material - Performance Metrics/cm_ALL_AdaBoost_Original.png]

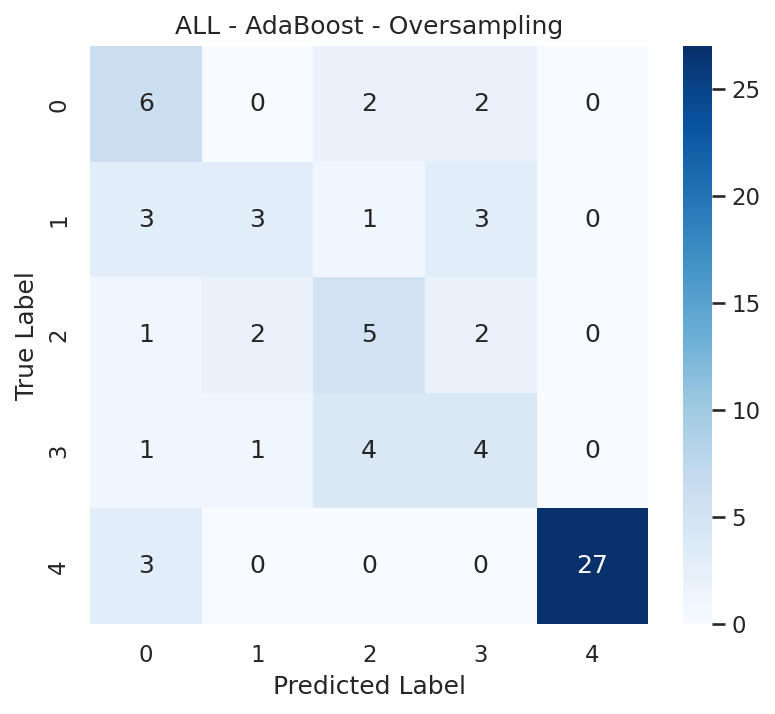

Supplement: Supplementary file 1 [file bioengineering-13-00787-s001.zip › Supplementary Material - Performance Metrics/cm_ALL_AdaBoost_Oversampling.png]

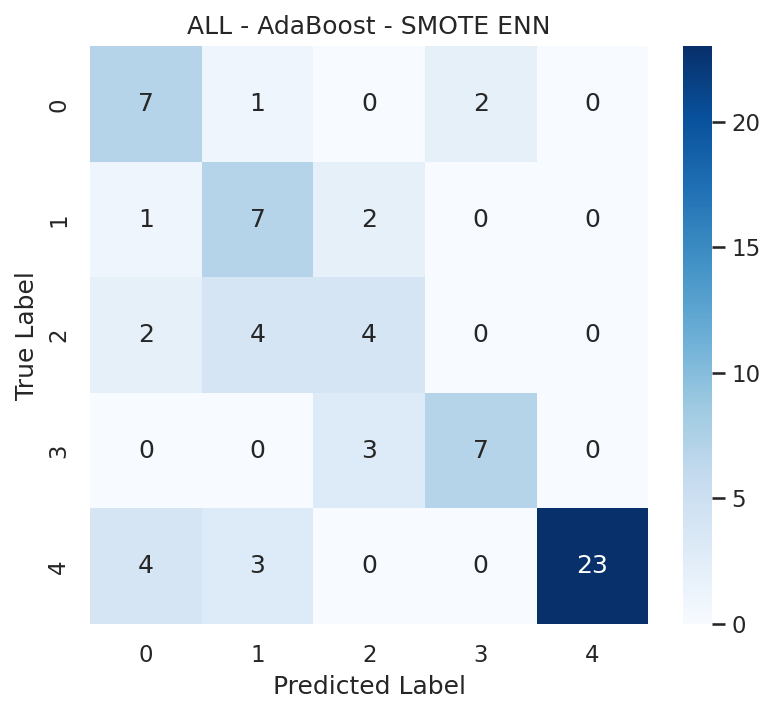

Supplement: Supplementary file 1 [file bioengineering-13-00787-s001.zip › Supplementary Material - Performance Metrics/cm_ALL_AdaBoost_SMOTE ENN.png]

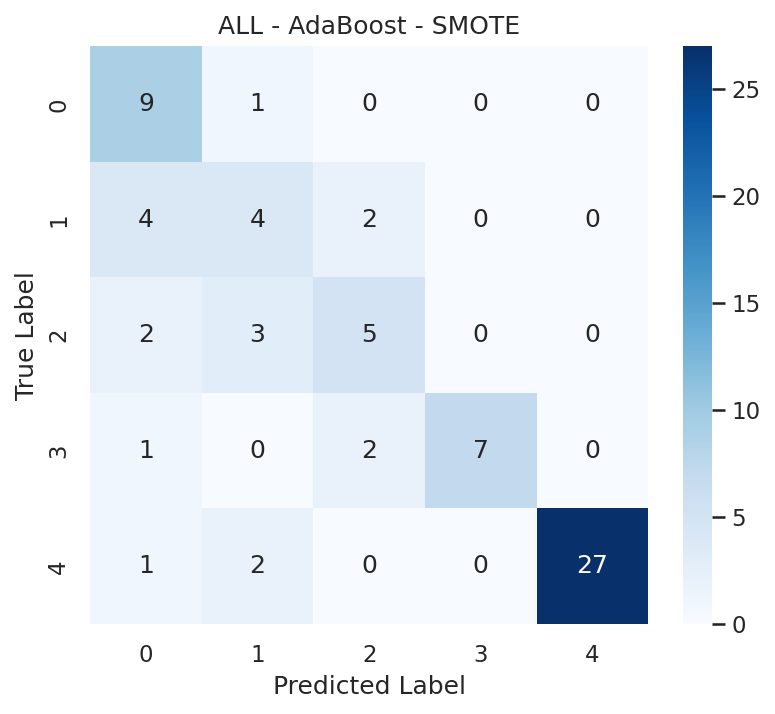

Supplement: Supplementary file 1 [file bioengineering-13-00787-s001.zip › Supplementary Material - Performance Metrics/cm_ALL_AdaBoost_SMOTE.png]

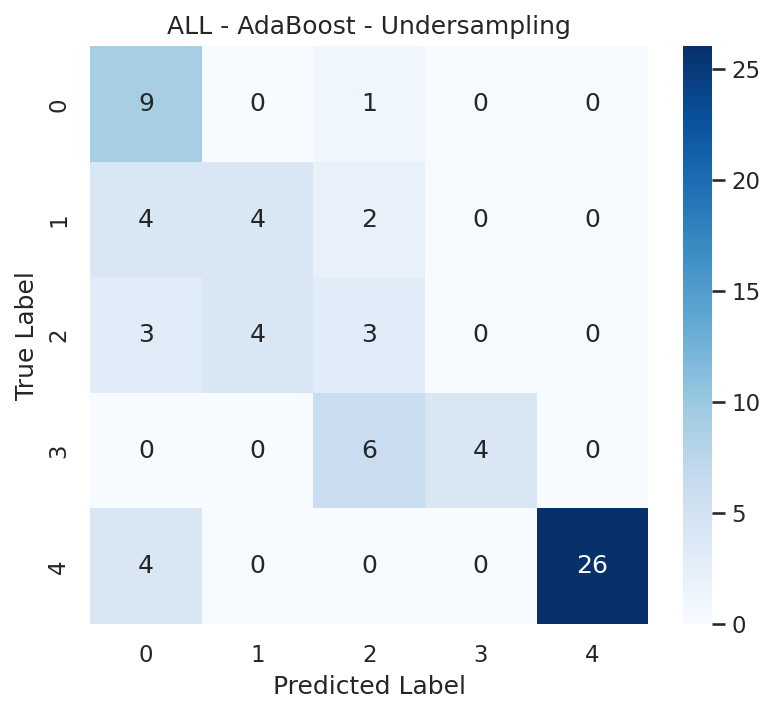

Supplement: Supplementary file 1 [file bioengineering-13-00787-s001.zip › Supplementary Material - Performance Metrics/cm_ALL_AdaBoost_Undersampling.png]

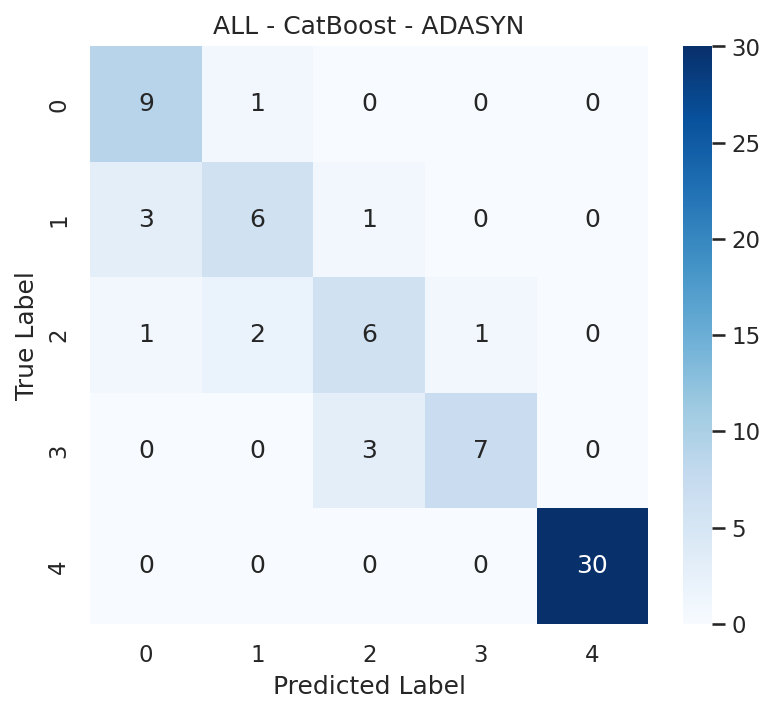

Supplement: Supplementary file 1 [file bioengineering-13-00787-s001.zip › Supplementary Material - Performance Metrics/cm_ALL_CatBoost_ADASYN.png]

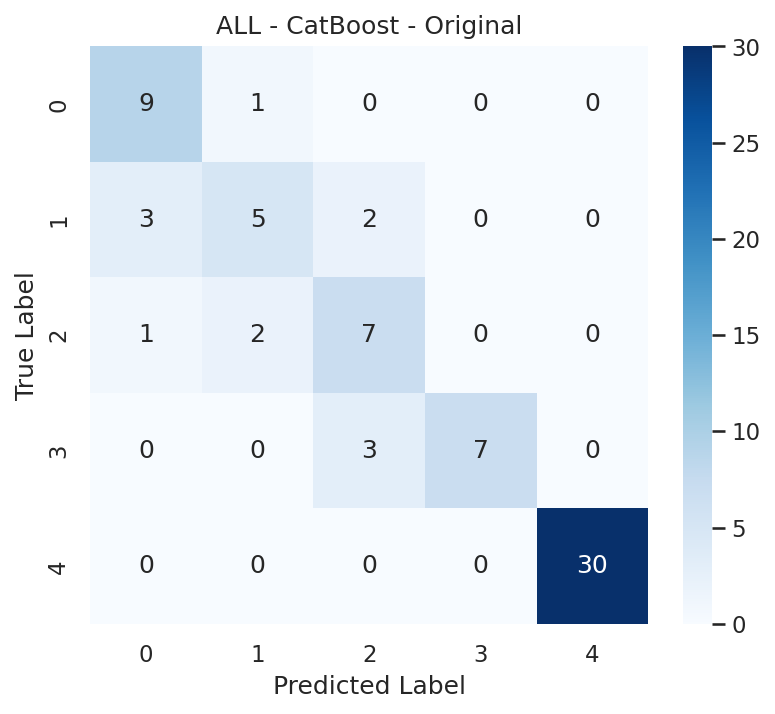

Supplement: Supplementary file 1 [file bioengineering-13-00787-s001.zip › Supplementary Material - Performance Metrics/cm_ALL_CatBoost_Original.png]

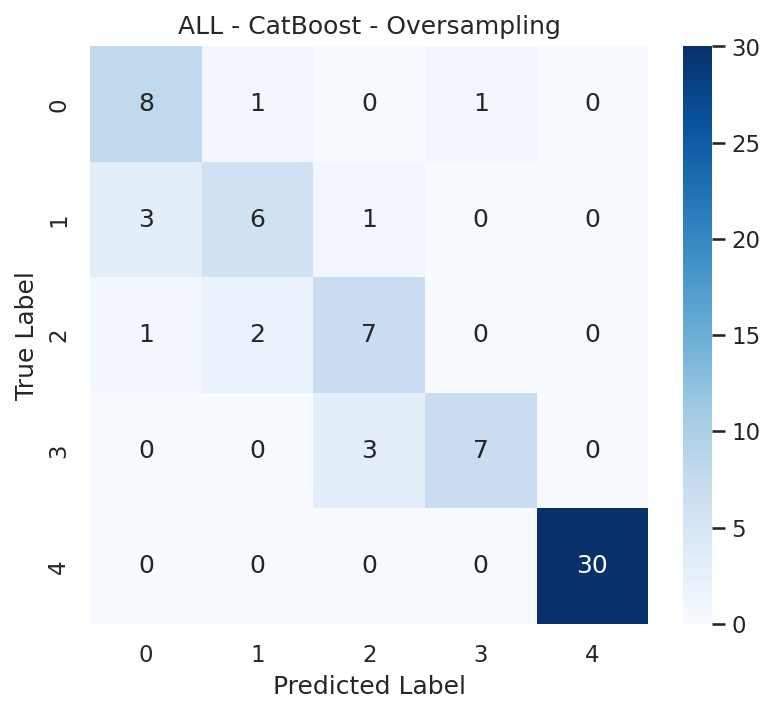

Supplement: Supplementary file 1 [file bioengineering-13-00787-s001.zip › Supplementary Material - Performance Metrics/cm_ALL_CatBoost_Oversampling.png]
